# Supplementary material for: Preliminary bone histological analysis of Lystrosaurus (Therapsida: Dicynodontia) from the Lower Triassic of North China, and its implication for lifestyle and environments after the end-Permian extinction
Source: PLoS One. 2021 Mar 18;16(3):e0248681. doi: 10.1371/journal.pone.0248681 (PMC7971864; doi:10.1371/journal.pone.0248681)

S1 Fig. The accurate sampled positions of all specimens in this study. A, IVPP V26543, arrow denotes the sampled position of the tibia; (**B, C)** IVPP V26544. B, the sampled positions of the tibia and fibula; **C**, the sampled position of the humerus; **D**, IVPP V26542, the distal end of the femur that sampled; **(E-G)** IVPP V26545. **E**, The sampled position of the radius; **F**, the sampled position of the fibula; **G**. The sampled position of the rib; **H**. IVPP V26547, arrow denotes the sampled position of the femur; **I**, IVPP V26546, arrow denotes the sampled position of the femur; **J**. IVPP V26548, arrow denotes the sampled position of the rib.

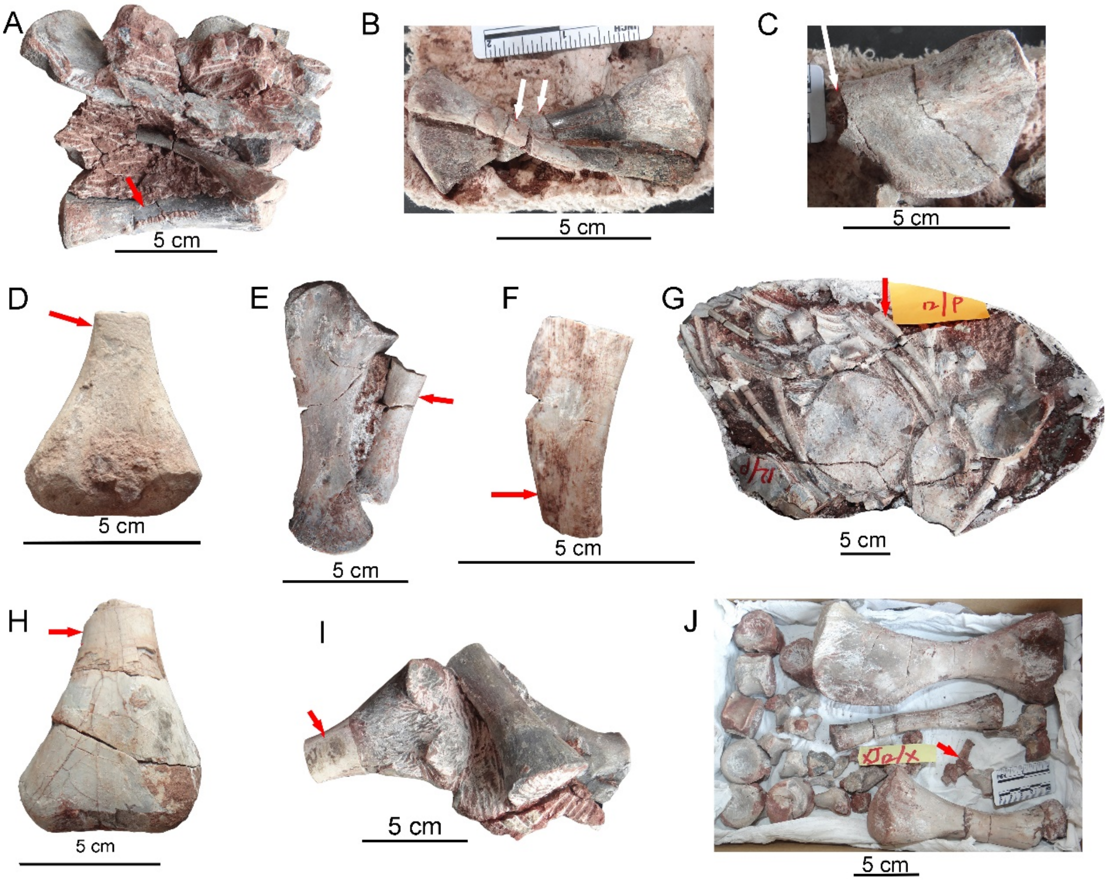

Supplement: S1 Fig — A, IVPP V26543, arrow denotes the sampled position of the right tibia in lateral view; (B, C) IVPP V26544. B, the sampled positions of the right tibia and fibula in anterior view; C, the sampled position of the left humerus in ventral view; D, IVPP V26542, the distal end of the femur in ventral view; (E-G) IVPP V26545. E, the sampled position of the left radius (and ulna) in anterior view; F, the sampled position of the fibula; G, the sampled position of the rib; H, IVPP V26547, arrow denotes the sampled position of the femur in dorsal view; I, IVPP V26546, arrow denotes the sampled position of the femur in ventral view; J, IVPP V26548, arrow denotes the sampled position of the rib. (PDF) [file pone.0248681.s001.pdf]
