# Supplementary material for: Preliminary bone histological analysis of Lystrosaurus (Therapsida: Dicynodontia) from the Lower Triassic of North China, and its implication for lifestyle and environments after the end-Permian extinction
Source: PLoS One. 2021 Mar 18;16(3):e0248681. doi: 10.1371/journal.pone.0248681 (PMC7971864; doi:10.1371/journal.pone.0248681)

Fig. S2. Measurements of the cortical thickness in all thin sections. A, IVPP V26543, tibia; **B**, IVPP V26544 tibia; **C**, IVPP V26544, humerus; **D**, IVPP V26544, fibula; **E**, IVPP V26542, femur; **F**, IVPP V26545, fibula; **G**, IVPP V26545, radius; **H**, IVPP V26546, femur; **I**, IVPP V26547, femur. The yellow lines denote measurements of the cortical thickness. The red lines denote measurements of cross-sectional diameters, and the green circles denote outlines of the medullary cavities.

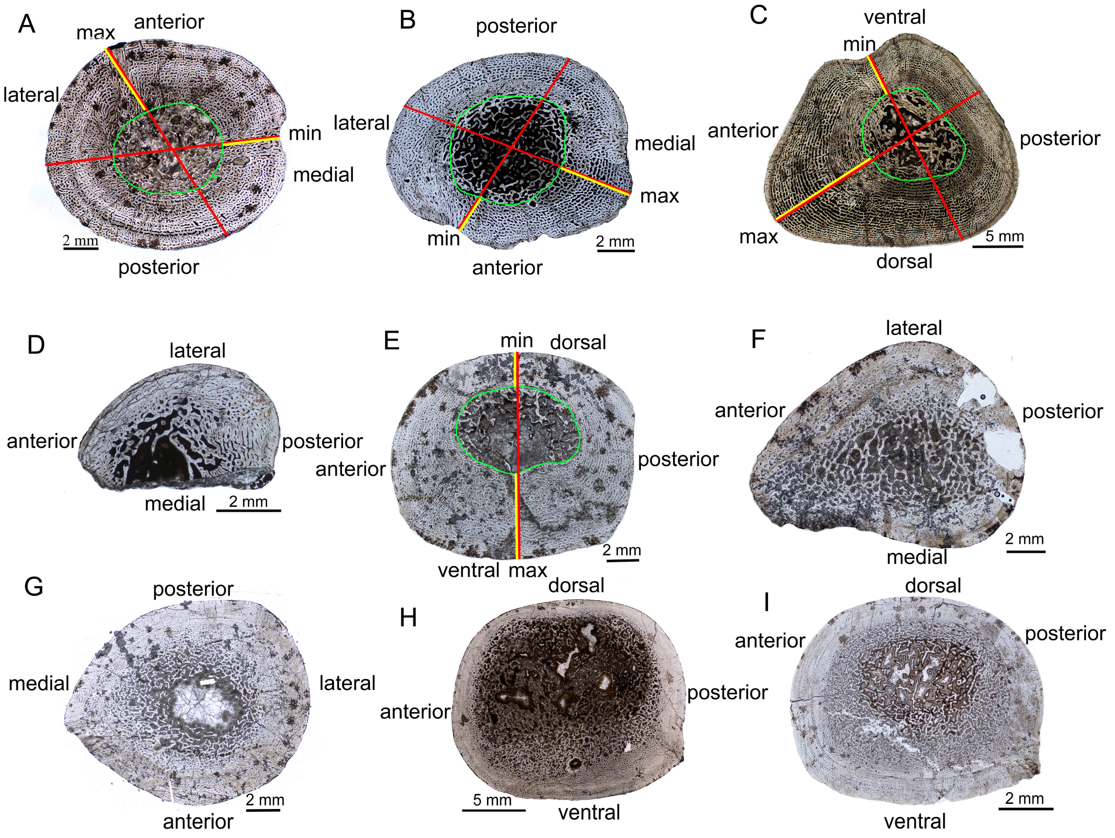

Supplement: S2 Fig — A, IVPP V26543, tibia; B, IVPP V26544 tibia; C, IVPP V26544, humerus; D, IVPP V26544, fibula; E, IVPP V26542, femur; F, IVPP V26545, fibula; G, IVPP V26545, radius; H, IVPP V26546, femur; I, IVPP V26547, femur. The yellow lines denote measurements of the cortical thickness. The red lines denote measurements of cross-sectional diameters, and the green circles denote outlines of the medullary cavities. (PDF) [file pone.0248681.s002.pdf]
