# Supplementary material for: Preliminary bone histological analysis of Lystrosaurus (Therapsida: Dicynodontia) from the Lower Triassic of North China, and its implication for lifestyle and environments after the end-Permian extinction
Source: PLoS One. 2021 Mar 18;16(3):e0248681. doi: 10.1371/journal.pone.0248681 (PMC7971864; doi:10.1371/journal.pone.0248681)
Supplement: S3 Fig — (PDF) [file pone.0248681.s003.pdf]

S3 Fig. Bone microstructure and the sampled areas for calculating cortical porosity in all thin sections

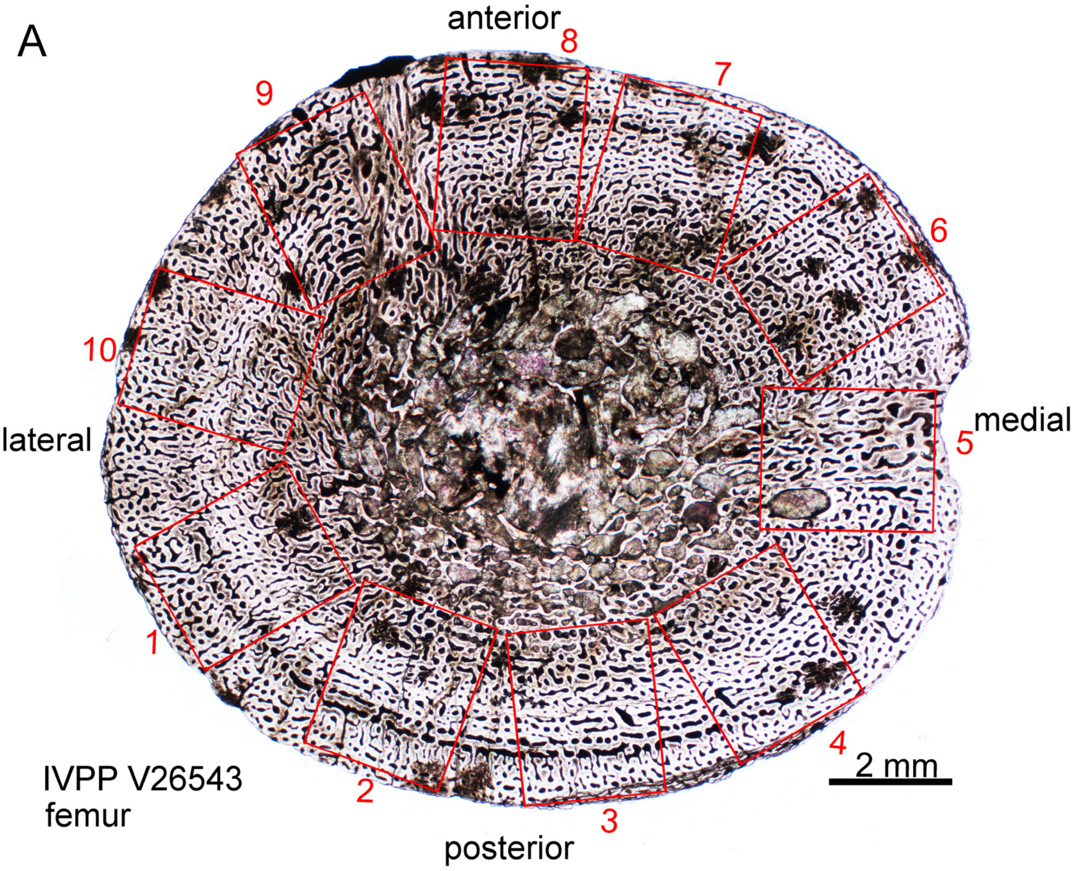

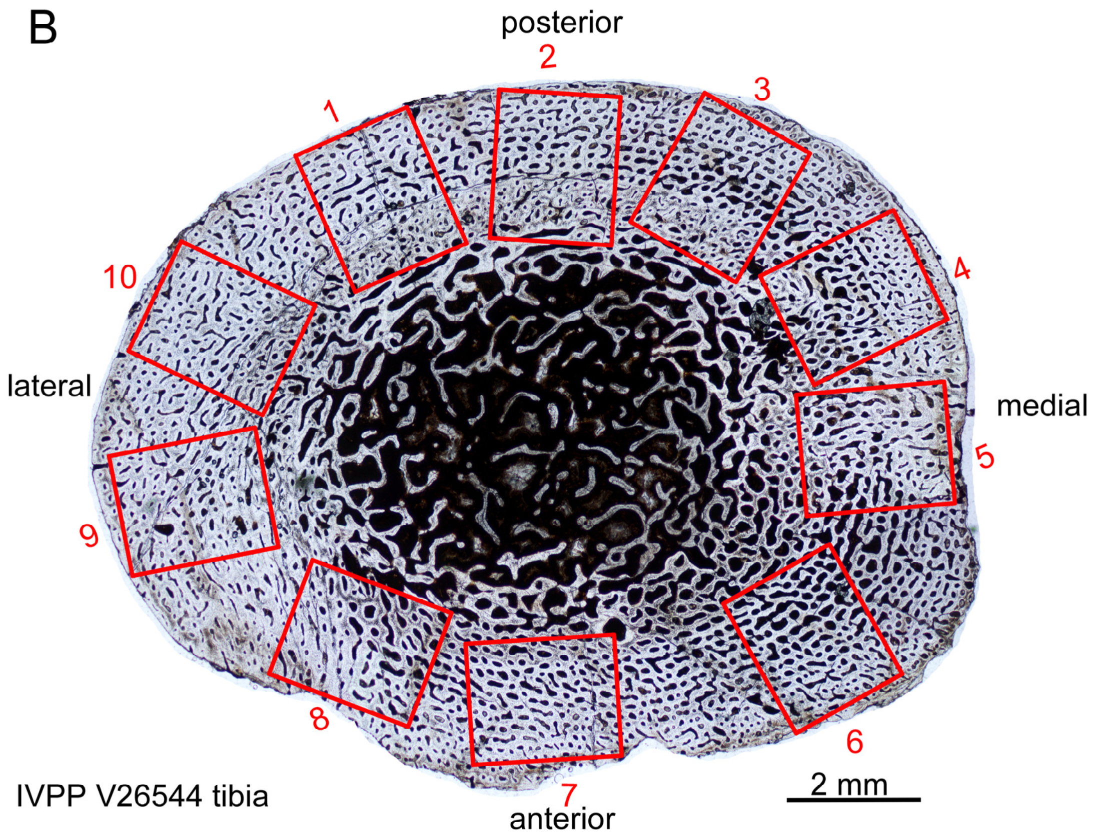
B
posterior
1
2
3
4
5
6
7
8
9
10
lateral
medial
anterior
2 mm
IVPP V26544 tibia

C

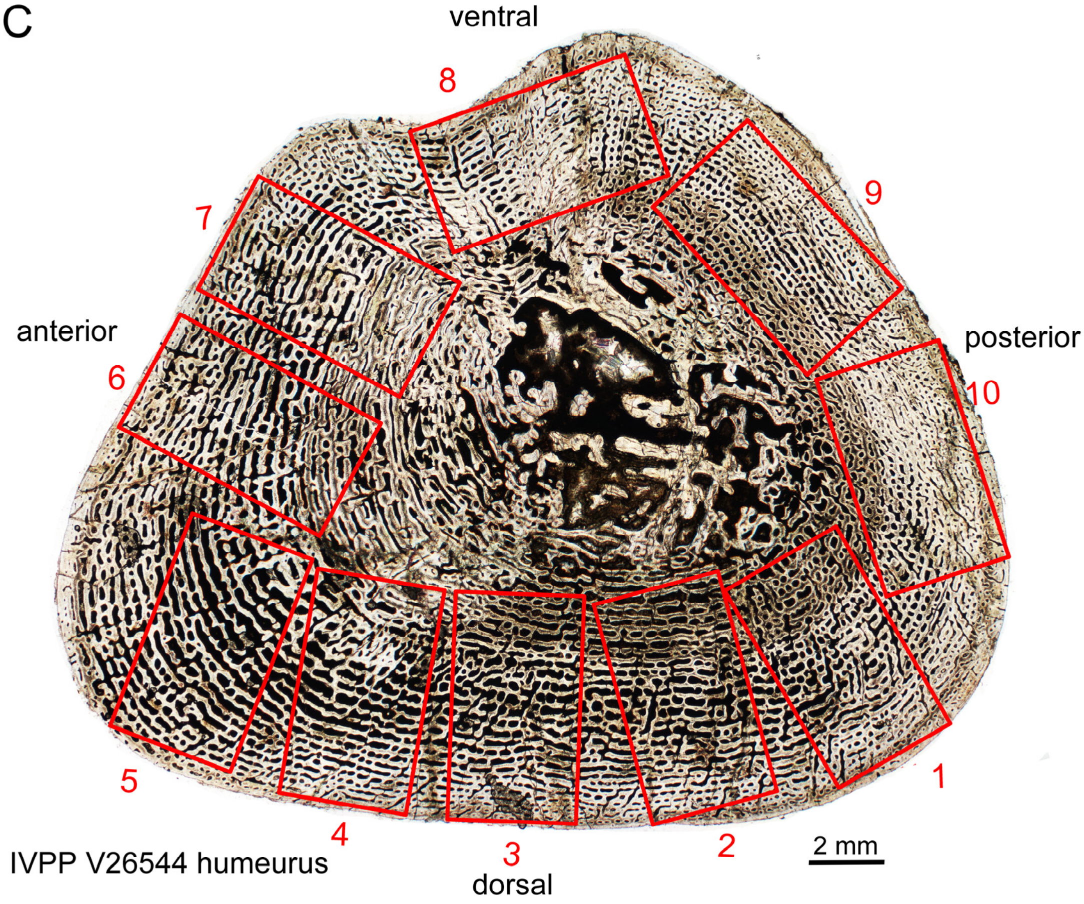
ventral
anterior
posterior
dorsal
8
7
9
6
10
5
4
3
2
1
IVPP V26544 humeurus
2 mm

D

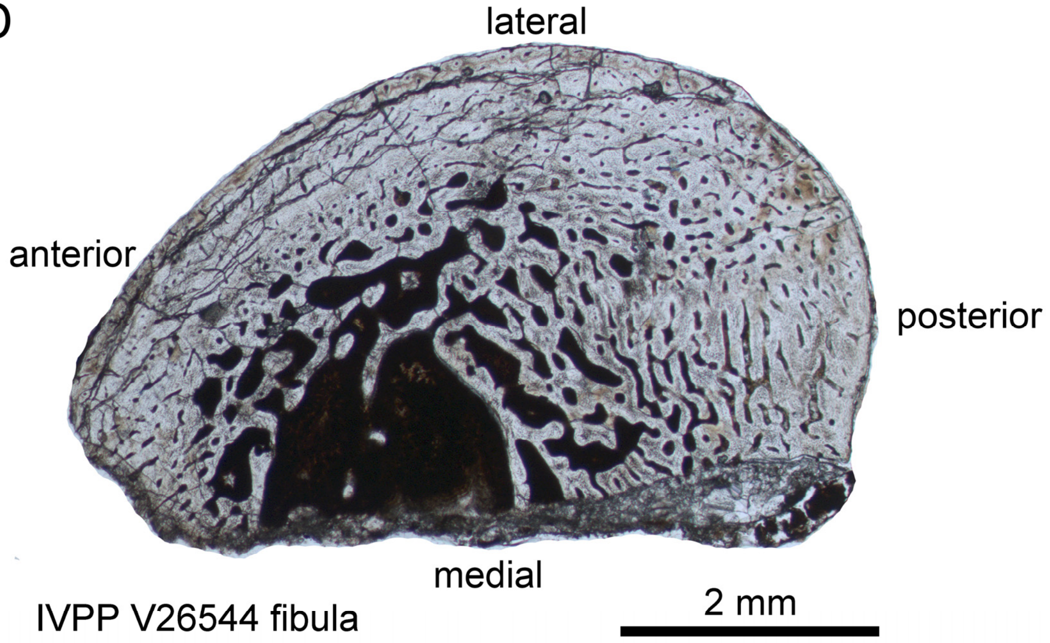
lateral
anterior
posterior
medial
IVPP V26544 fibula
2 mm

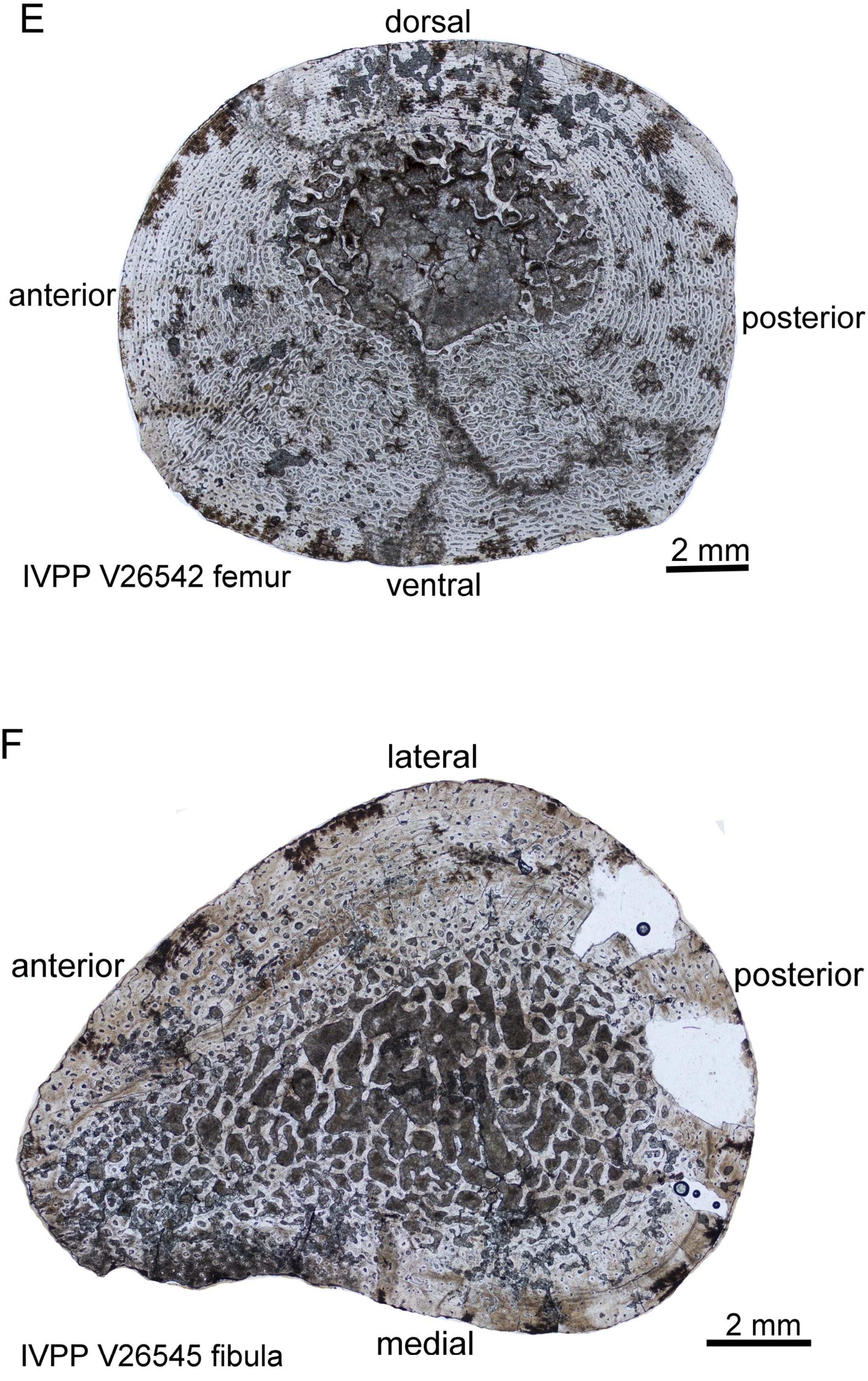
E
dorsal
anterior
posterior
2 mm
IVPP V26542 femur
ventral
F
lateral
anterior
posterior
2 mm
IVPP V26545 fibula
medial

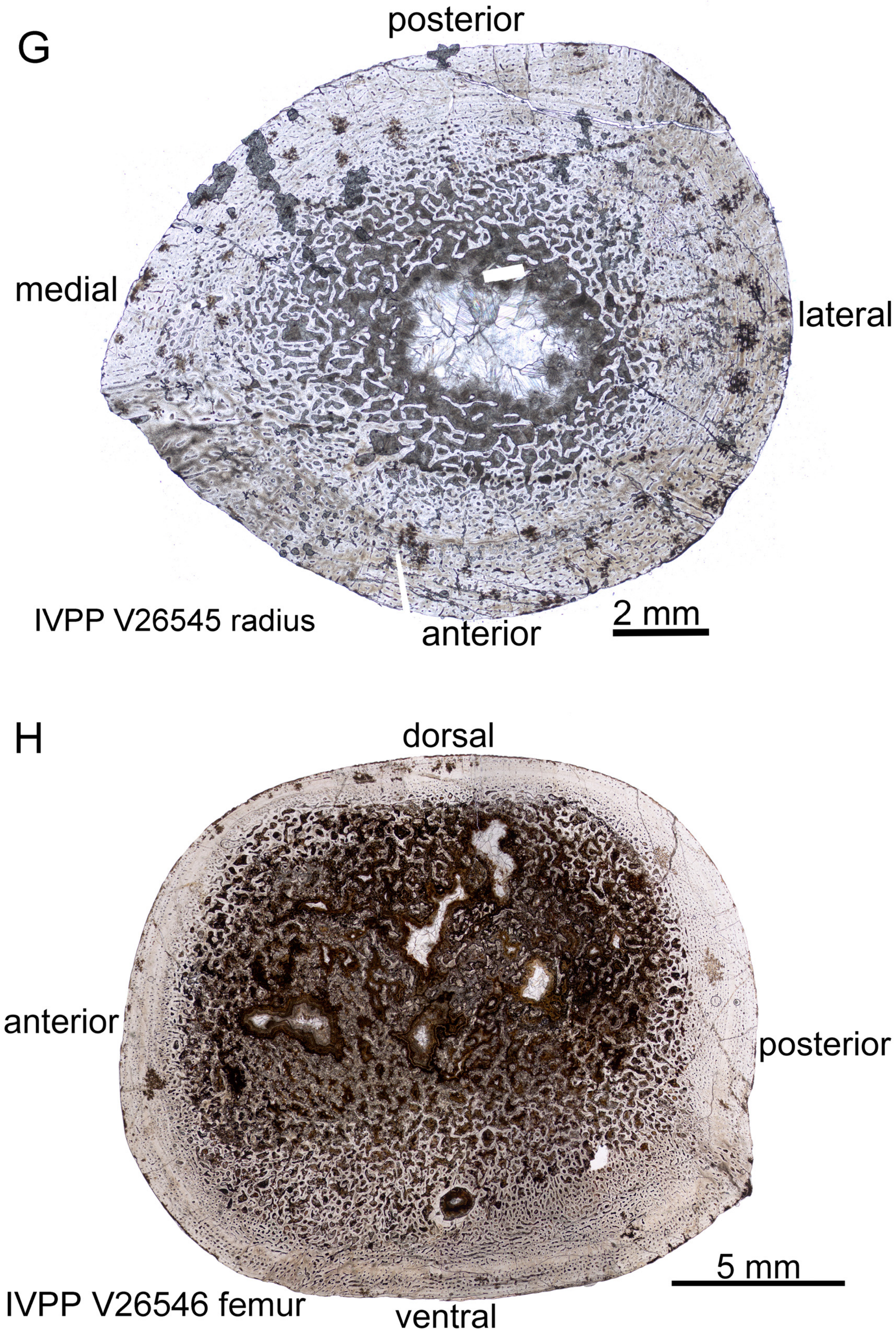
G
posterior
medial
lateral
IVPP V26545 radius
anterior
2 mm
H
dorsal
anterior
posterior
IVPP V26546 femur
ventral
5 mm

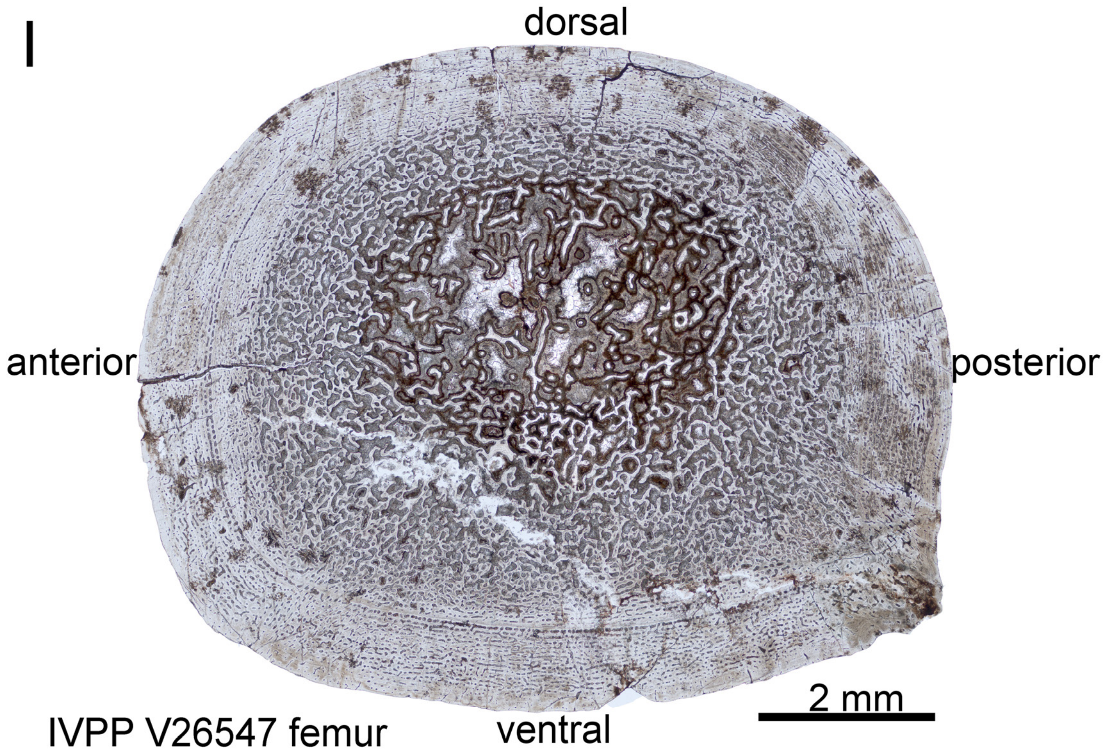
I
dorsal
anterior
posterior
ventral
2 mm
IVPP V26547 femur
