# Supplementary material for: Preliminary bone histological analysis of Lystrosaurus (Therapsida: Dicynodontia) from the Lower Triassic of North China, and its implication for lifestyle and environments after the end-Permian extinction
Source: PLoS One. 2021 Mar 18;16(3):e0248681. doi: 10.1371/journal.pone.0248681 (PMC7971864; doi:10.1371/journal.pone.0248681)
Supplement: S4 Fig — (PDF) [file pone.0248681.s004.pdf]

S4 Fig. Comparison of the humeral length between Chinese and South Africa *Lystrosaurus*

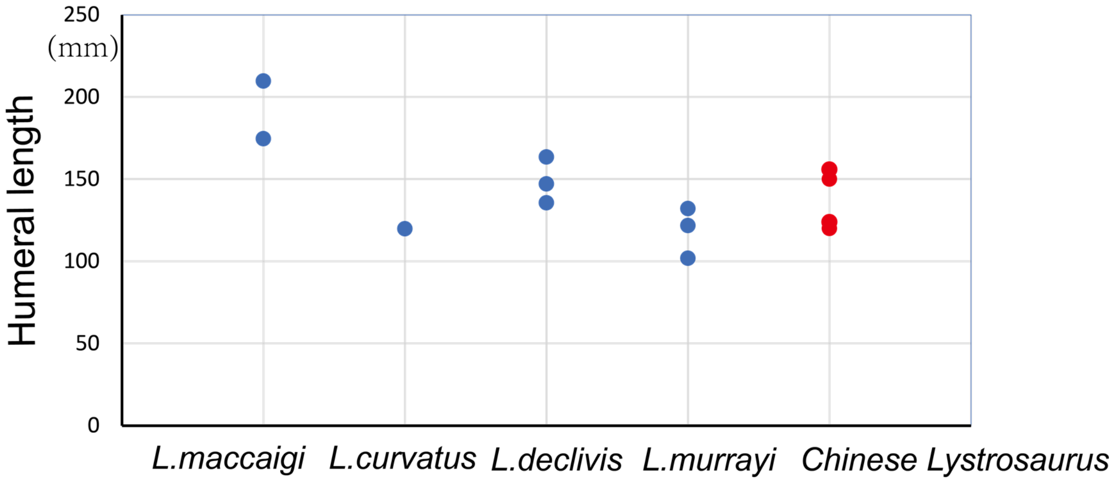

Taxa	Specimen number	Elements	Length (mm)	LAG number
L. maccaigi	NMQR 3922c	humerus	174.71	2
L. maccaigi	NMQR 3663a	humerus	209.8	7
L. curvatus	NMQR 3922a	humerus	119.87	2
L. declivis	SAM-PK-K8013a	humerus	135.7	1
L. declivis	NMQR 3181	humerus	147.24	1
L. declivis	NMQR 1485	humerus	163.49	1
L. murrayi	SAM-PK-8991a	humerus	101.88	1
L. murrayi	NMQR 3963	humerus	121.84	1
L. murrayi	BP/1/3236	humerus	132.18	1
Chinese *Lystrosaurus*	IVPP V26545	humerus	150	1
Chinese *Lystrosaurus*	IVPP V26546	humerus	120	3
Chinese *Lystrosaurus*	IVPP V26547	humerus	124	4
Chinese *Lystrosaurus*	IVPP V26548	humerus	156	>2
