# Supplementary material for: Preliminary bone histological analysis of Lystrosaurus (Therapsida: Dicynodontia) from the Lower Triassic of North China, and its implication for lifestyle and environments after the end-Permian extinction
Source: PLoS One. 2021 Mar 18;16(3):e0248681. doi: 10.1371/journal.pone.0248681 (PMC7971864; doi:10.1371/journal.pone.0248681)

PLOS ONE

Bone histology of Lystrosaurus (Therapsida: Dicynodontia) from the Lower Triassic of North China, and its implication for lifestyle and environments after the end-Permian extinction

--Manuscript Draft--

**Manuscript Number:**	PONE-D-20-17684
**Article Type:**	Research Article
**Full Title:**	Bone histology of Lystrosaurus (Therapsida: Dicynodontia) from the Lower Triassic of North China, and its implication for lifestyle and environments after the end-Permian extinction
**Short Title:**	Bone histology of Lystrosaurus from North China
**Corresponding Author:**	Fenglu Han, Ph.D<br>China University of Geosciences (Wuhan)<br>Wuhan, CHINA
**Keywords:**	bone histology; Lystrosaurus; Early Triassic; China
**Abstract:**	Lystrosaurus represents one of the most successful dicynodonts that survived the end-Permian mass extinction and was abundant in the Early Triassic, but its life strategy is still controversial. The bone histology of Lystrosaurus from South Africa and India has provided important information on the growth strategy and lifestyle, but the bone histology of Lystrosaurus from China has still not been studied. Here, we report on the bone microstructure of Lystrosaurus from the Lower Triassic of Xinjiang, China, based on seven individuals for the first time. Our samples suggest that the bone microstructure of Lystrosaurus from China consists of fibrolamellar bone tissue similar to those from South Africa and India. Three ontogenetic stages were identified: juvenile, subadult, and adult based on Line of arrested growth (LAG) and bone tissue changes. The bone histology supports a rapid growth strategy for Lystrosaurus during early ontogeny. Unlike the bone microstructure of the Early Triassic Lystrosaurus from South Africa, lines of arrested growth are common in our specimens, which suggests the growth interruption in Chinese Lystrosaurus . The distinct bone histology in Lystrosaurus from South Africa and China may indicate different environmental conditions in these two regions.
**Order of Authors:**	Fenglu Han, Ph.D
	Qi Zhao
	Jun Liu
**Additional Information:**	
**Question**	**Response**
**Financial Disclosure**<br><br>Enter a financial disclosure statement that describes the sources of funding for the work included in this submission. Review the submission guidelines for detailed requirements. View published research articles from *PLOS ONE* for specific examples.<br><br>This statement is required for submission and **will appear in the published article** if the submission is accepted. Please make sure it is accurate.	This study was supported by Strategic Priority Research Program of Chinese Academy of Sciences (XDB26000000) to JL, the National Natural Science Foundation of China (www.nsfc.gov.cn; 41530104, 41661134047) to FH and JL respectively. The funders had no role in study design, data collection and analysis, decision to publish, or preparation of the manuscript.

Powered by Editorial Manager® and ProduXion Manager® from Aries Systems Corporation

**Unfunded studies**<br>Enter: *The author(s) received no specific funding for this work.*<br><br>**Funded studies**<br>Enter a statement with the following details:<br>• Initials of the authors who received each award<br>• Grant numbers awarded to each author<br>• The full name of each funder<br>• URL of each funder website<br>• Did the sponsors or funders play any role in the study design, data collection and analysis, decision to publish, or preparation of the manuscript?<br>• **NO** - Include this sentence at the end of your statement: *The funders had no role in study design, data collection and analysis, decision to publish, or preparation of the manuscript.*<br>• **YES** - Specify the role(s) played.<br><br>* typeset	
**Competing Interests**<br><br>Use the instructions below to enter a competing interest statement for this submission. On behalf of all authors, disclose any competing interests that could be perceived to bias this work—acknowledging all financial support and any other relevant financial or non-financial competing interests.<br><br>This statement **will appear in the published article** if the submission is accepted. Please make sure it is accurate. View published research articles from *PLOS ONE* for specific examples.	The authors have declared that no competing interests exist.

Powered by Editorial Manager® and ProduXion Manager® from Aries Systems Corporation

**NO authors have competing interests**<br><br>Enter: *The authors have declared that no competing interests exist.*<br><br>**Authors with competing interests**<br><br>Enter competing interest details beginning with this statement:<br><br>*I have read the journal's policy and the authors of this manuscript have the following competing interests: [insert competing interests here]*<br><br>* typeset	
**Ethics Statement**<br><br>Enter an ethics statement for this submission. This statement is required if the study involved:<br><br>• Human participants<br>• Human specimens or tissue<br>• Vertebrate animals or cephalopods<br>• Vertebrate embryos or tissues<br>• Field research<br><br>Write "N/A" if the submission does not require an ethics statement.<br><br>General guidance is provided below. Consult the submission guidelines for detailed instructions. **Make sure that all information entered here is included in the Methods section of the manuscript.**	N/A

Powered by Editorial Manager® and ProduXion Manager® from Aries Systems Corporation

**Format for specific study types**<br><br>**Human Subject Research (involving human participants and/or tissue)**<br>• Give the name of the institutional review board or ethics committee that approved the study<br>• Include the approval number and/or a statement indicating approval of this research<br>• Indicate the form of consent obtained (written/oral) or the reason that consent was not obtained (e.g. the data were analyzed anonymously)<br><br>**Animal Research (involving vertebrate animals, embryos or tissues)**<br>• Provide the name of the Institutional Animal Care and Use Committee (IACUC) or other relevant ethics board that reviewed the study protocol, and indicate whether they approved this research or granted a formal waiver of ethical approval<br>• Include an approval number if one was obtained<br>• If the study involved *non-human primates*, add *additional details* about animal welfare and steps taken to ameliorate suffering<br>• If anesthesia, euthanasia, or any kind of animal sacrifice is part of the study, include briefly which substances and/or methods were applied<br><br>**Field Research**<br><br>Include the following details if this study involves the collection of plant, animal, or other materials from a natural setting:<br>• Field permit number<br>• Name of the institution or relevant body that granted permission	
**Data Availability**<br><br>Authors are required to make all data underlying the findings described fully available, without restriction, and from the time of publication. PLOS allows rare exceptions to address legal and ethical concerns. See the PLOS Data Policy and FAQ for detailed information.	Yes - all data are fully available without restriction

A Data Availability Statement describing where the data can be found is required at submission. Your answers to this question constitute the Data Availability Statement and **will be published in the article**, if accepted.<br><br>**Important:** Stating 'data available on request from the author' is not sufficient. If your data are only available upon request, select 'No' for the first question and explain your exceptional situation in the text box.<br><br>Do the authors confirm that all data underlying the findings described in their manuscript are fully available without restriction?	
**Describe where the data may be found in full sentences. If you are copying our sample text, replace any instances of XXX with the appropriate details.**<br><br>• If the data are **held or will be held in a public repository**, include URLs, accession numbers or DOIs. If this information will only be available after acceptance, indicate this by ticking the box below. For example: *All XXX files are available from the XXX database (accession number(s) XXX, XXX.).*<br>• If the data are all contained **within the manuscript and/or Supporting Information files**, enter the following: *All relevant data are within the manuscript and its Supporting Information files.*<br>• If neither of these applies but you are able to provide **details of access elsewhere**, with or without limitations, please do so. For example:<br><br>*Data cannot be shared publicly because of [XXX]. Data are available from the XXX Institutional Data Access / Ethics Committee (contact via XXX) for researchers who meet the criteria for access to confidential data.*<br><br>*The data underlying the results presented in the study are available from (include the name of the third party*	All relevant data are within the manuscript and its Supporting Information files.

and contact information or URL).<br>• This text is appropriate if the data are owned by a third party and authors do not have permission to share the data.<br><br>* typeset	
Additional data availability information:	

Click here to download Manuscript Hanetal manuscript.docx

Bone histology of *Lystrosaurus* (Therapsida: Dicynodontia) from the Lower Triassic of North China, and its implication for lifestyle and environments after the end-Permian extinction

Fenglu Han[1*], Qi Zhao[2], Jun Liu[2,3, 4*]

[1] School of Earth Sciences, China University of Geosciences (Wuhan), Wuhan, China

[2] Key Laboratory of Vertebrate Evolution and Human Origins of Chinese Academy of Sciences, Institute of Vertebrate Paleontology and Paleoanthropology, Chinese Academy of Sciences, Beijing, China

3 CAS Center for Excellence in Life and Paleoenvironment, Beijing 100044, China;

4 College of Earth and Planetary Sciences, University of Chinese Academy of Sciences Beijing 100049

* Corresponding author

Email: hanfl@cug.edu.cn (FH); liujun@ivpp.ac.cn (JL)

Abstract

Lystrosaurus represents one of the most successful dicynodonts that survived the end-Permian mass extinction and was abundant in the Early Triassic, but its life strategy is still controversial. The bone histology of *Lystrosaurus* from South Africa and India has provided important information on the growth strategy and lifestyle, but the bone histology of *Lystrosaurus* from China has still not been studied. Here, we report on the bone microstructure of *Lystrosaurus* from the Lower Triassic of Xinjiang, China, based on seven individuals for the first time. Our samples suggest that the bone microstructure of *Lystrosaurus* from China consists of fibrolamellar bone tissue similar to those from South Africa and India. Three ontogenetic stages were identified: juvenile, subadult, and adult based on line of arrested growth (LAG) and bone tissue changes. The bone histology supports a rapid growth strategy for *Lystrosaurus* during early ontogeny. Unlike the bone microstructure of the Early Triassic *Lystrosaurus* from South Africa, lines of arrested growth are common in our specimens, which suggests the growth interruption in Chinese *Lystrosaurus*. The distinct bone histology in *Lystrosaurus* from South Africa and China may indicate different environmental conditions in these two regions.

Introduction

The extinction at the end of the Permian is the greatest biological extinction event since the Cambrian explosion. About 80% of all marine species and 50-70% of terrestrial families went extinct [1-3]. Recent studies suggest that the Permian-Triassic transition occurred in a hot and arid environment, which is unsuitable for life [4-8]. However, some organisms evolved new survival strategies, such as miniaturization or rapid propagation [9-11].

Lystrosaurus is ~~a type~~ of medium to large-sized dicynodont therapsid with a strongly anteroventrally curved snout. It represents one of the most successful vertebrates that survived ~~passed through~~ the end-Permian mass extinction with a cosmopolitan distribution during the Early Triassic [12-16], and their survival strategies are still controversial [17, 18]. The bone histology of *Lystrosaurus* from South Africa suggests it may be aquatic or at least semi-aquatic, which may have helped them to escape the harsh terrestrial environment [19, 20]. However, there is also evidence that *Lystrosaurus* was a borrow maker [21, 22], and burrowing can help an organism survive from ~~the~~ harsh environmental conditions ~~as the temperature and humidity are more stable and comfortable in a cave than aboveground~~ [23, 24]. Also, underground burrows can provide better protection for young organisms, which is helpful for breeding [25].

Interestingly, the body size of *Lystrosaurus* from South Africa is smaller in the Early Triassic than in the late Permian [17], suggesting the miniaturization, but the [illegible]Gs are more common in Permian *Lystrosaurus* indicating a subadult or adult stage, whereas they are very rare from Early Triassic *Lystrosaurus*, which suggests that they were possibly breeding young to compensate for dying at an early age [18].

In summary, bone histology can provide important information on the survival strategies of *Lystrosaurus*. However, many studies have focused only on the *Lystrosaurus* from South Africa and India [19, 20, 26, 27]. *Lystrosaurus* is also very abundant in the Juggar and Turpan basins of Xinjiang, China, as well as in South Africa. In China, seven species have been erected since 1935, but the validity of most species needs to be tested [28, 29], and it is possible that only two species, *L.hedeni* and *L. youngi*, are valid [30]. Here, we present the bone histology of *Lystrosaurus* from the Lower Triassic Turpan Basin, Xinjiang Province, China for the first time. Our ~~purpose here is~~

~~to~~ study describes the bone histology of *Lystrosaurus* from the Early Triassic of China ~~and~~ to deduce its survival strategy.

Material and methods

Seven specimens of *Lystrosaurus* from the collections of the Institute of Vertebrate Paleontology and Paleoanthropology (IVPP) were selected for histological analysis. All ~~these~~ materials come from the Lower Triassic Jiucaiyuan Formation of Taodonggou area (Taoshuyuan), Turpan Basin, Xinjiang, China [31, 32]. The age of Jiucaiyuan Formation in Taodonggou area is middle-upper Indian based on previous chronostratigraphy [31, 33]. The identification to species level is currently impossible ~~right now~~ due to poor preservation or incomplete preparation. The bone histology of the four *Lystrosaurus* species from the Lower Triassic of South Africa is quite similar in morphology [27, 34], although more LAGs are present in the *Lystrosaurus* from the late Permian, which may be caused by a distinct environmental condition [18]. Therefore, here we suppose that *Lystrosaurus* from the Taodonggou area of Xinjiang China should have identical morphology on bone histology even in different species. However, they may have distinct bone histological information from other places due to different environmental conditions [20].

Sections were sampled from the mid-diaphysis of limb bones including the tibia, femur, humerus, ulna, and radius because they are the primary centers of ossification, and should preserve the most completed record of growth, and also diminish the potential for secondary remodeling to destroy the growth record of the cortical bones (Fig S1; Table 1) [35, 36]. The sections of ribs in some individuals were also sampled for comparison. The preparation of the histological sections was carried out at the IVPP. The selected bones were processed using the Exakt-Cutting-Grinding

System [37]. Sections were embedded in polyester resin, cut by EXAKT-300CP, and were grinded to a thickness of about 50 μm with an EXAKT 400CS grinding machine. The polished sections were then observed under the transmitted and polarized light. The thin sections were observed using a Leica DM2700P microscope and photographed using a Leica DMC4500 camera. Nomenclature and definitions of structures follow [38, 39]. Growth marks and LAGs both indicate growth lines here, but the growth marks are identified to be LAGs only when the marked lines are traced around the full circumference of the bone [35].

Table 1 Skeletal elements of the specimens examined for histology from the Early Triassic *Lystrosaurus*, Xinjiang, China.

Specimen number	Elements examined	Humeral length (cm)	Radial length (cm)	Femoral length (cm)	Tibial length (cm)	Length of fibulae (cm)	Growth stages
IVPP V26543	Tibia	——	——	12.5	9	——	juvenile
IVPP V26544	Humerus, Tibia, Fibula	11	7.5	——	9	9	juvenile
IVPP V26542	Femur	11	7.5	——	——	——	subadult

IVPP V26545	Fibula, Radius	15.5	11[e]	——	——	11[e]	subadult
IVPP V26546	Femur	——	——	——	——	12.5	adult
IVPP V26547	Femur	——	12[e]	17	12	12	adult
IVPP V26548	Rib	——	14	19	14	13.5	adult

[illegible]note estimated values .

Cortical porosity was measured using the analyze particles tool in ImageJ 1.52a (https://imagej.nih.gov/ij/). It was difficult to obtain the cortical porosity of a whole cross-section for each specimen. Therefore, we chose as many as te[illegible]as from the inner to the outermost cortex to calculate its average vascular porosity. High-resolution images were acquired using the Leica DMC4500 camera. ~~Then all the images were converted to an 8-bit file, and the brightness/contrast was adjusted to make them clear. After that, we use “threshold” tools in ImageJ to choose all the vascular canals. Finally, the cortical porosity was produced using “analyze particles” tools in ImageJ.~~ The average cortical thickness was calculated as the ratio of the cortex to the cross-sectional diameter. ~~The medullary cavity is usually round or elliptical.~~ First, we choose two points to draw a line, which is the longest within the medullary cavity. And then we choose the midpoint of the line and draw another line that is perpendicular to the first line. Along these two lines, we measured the

cortical thickness and was divided by the whole diameters. This value represents the minimum cortical thickness of each element.

Results

The bone histology of *Lystrosaurus* from North China shows a predominance of fibrolamellar bone tissue and a high cortical porosity (Figs 1-6), as in *Lystrosaurus* from South Africa and India [19]. The primary osteons are predominantly longitudinal, forming laminar or plexiform bone. Radially oriented primary osteons are also present in some parts of cortex. Secondary remodeling is common in the inner and mid-cortical bone in subadult and adult stages, resulting in enlarged channels and a thin periosteal bone. The free medullary cavity is small and filled with bony trabeculae. Lines of arrested growth (LAGs) are common in the outer cortex of larger individuals.

All these elements could be divided into juvenile, subadult and adult stages based on bone tissue type and the presence of LAGs (**Table 2**). They are generally similar to the growth stages of *Lystrosaurus* from South Africa and India, but also have some features that differ from the latter.

Table 2. Bone histological information of *Lystrosaurus* from China in each specimen.

Specimen number	Skeletal elements	Cortical thickness /porosity (%)	Bone tissue type	Secondary reconstruction	Growth rings	Medullary cavity	Stages

IVPP V26543	Tibia	28/29	plexiform and reticular	inner enlarged channels	0	a small free cavity	juvenile
IVPP V26544	Humerus	30/32	plexiform	a few secondary osteons	0	a small free cavity	juvenile
	Tibia	24/27	laminar and reticular	a few secondary osteons	0	full of trabeculae	juvenile
	Fibula	25/22	laminar	a few secondary osteons	0	a large free cavity	juvenile
IVPP V26542	femur	27/38	laminar and plexiform	extensive secondary reconstruction	1	a free medullary cavity	subadult
IVPP V26545	fibula	25/18	laminar	a few secondary osteons	1	full of trabeculae	subadult
	Radius	26/20	laminar	a few secondary osteons	1	full of trabeculae	subadult
IVPP	femur	15/24	laminar	extensive	3	full of	adult

V26546				secondary reconstrution		trabeculae	
IVPP V26547	Femur	25/22	laminar	extensive secondary reconstruction	4	full of trabeculae	adult
IVPP V26548	rib	/	laminar	extensive secondary reconstruction	>3	full of trabeculae	adult

Juvenile

The juvenile stage was represented by two samples: IVPP V 26543 and IVPP V 26544. All the cortices composed of a woven-fibered bone matrix, a high number of vascular canals, abundant globular osteocyte lacunae, and no LAGs, suggesting a rapid, constant growth rate. The mid-diaphyseal tibia of IVPP V 26543 is elliptical in cross-section, with a diameter of 12.5×14.3 mm and an average cortical thickness of about 28% (Fig 1). The free medullary cavity is small with a diameter of about 4 mm. The entire cortex is comprised of woven fibered bone matrix and dense vascular canals (Fig 1). The cortex has a high cortical porosity (about 29%). The canals are predominantly longitudinal. A few irregularly shaped canals are shown in the inner and mid cortex (Fig 1A). Radial anastomoses form two narrow regions throughout the cortex, and one region has a concave periosteal surface (Fig 1B, C). Anastomosing canals are found in the outer cortex

(Fig 1D) and ~~link four to five canals forming long canals~~ in the mid and inner cortex (Fig 1A).

of the vascular channels show thin centripetal osteonal deposits forming primary osteons (Fig 1E, F). The primary osteons mainly show laminar and plexiform patterns in the outer and deeper cortex, and a row of wide-open canals are shown near the periphery (periosteal surface) in some areas (Fig 1D). Osteocyte density is consistently high throughout the cortex. Erosionally enlarged resorption cavities are present in the inner cortex (Fig 1A, C). There are no secondary osteons, LAGs, and endosteal bone. The medullary region contains ~~a~~ cancellous bone, ~~and a free small cavity is present.~~

Fig 1. Bone microstructures in juvenile *Lystrosaurus* IVPP V26543.

(A) partial cross-section showing fibrolamellar bone. (B) whole cross-section of the tibia. (C) partial cross-section showing radial vascular canals. (D) the outermost cortex under normal light. (E) enlargement of the outer cortex showing primary osteons under normal light. (F) bone tissue of E under polarized light. **Abbreviations**: **rc**, radial canals; **wc**, wide-open canals.

In IVPP V26544, the tibia, humerus, and fibula were all sectioned (Fig 2). The cross-section of the tibia is elliptical in outline, with a diameter of 15×12 mm (Fig 2A). The cortical thickness is about 24%. There is no free medullary cavity. The cortex mainly consists of fibrolamellar bone tissues (Fig 2B), but parallel-fibered bone tissue is present in the medial region of the cortex articulated with the fibula (Fig 2C). Vascular canals are predominantly longitudinal. There are a few anastomoses that link two to five canals forming long straight and irregular canals. Most of the vascular canals are primary osteons forming a laminar pattern (Fig 2B). A thin layer (1 mm) of secondary osteons is present in the inner region of the compact bone (Fig 2D). The cortex has a high porosity of 27% (Fig 2A), but the diameter of vascular canals is slightly reduced near the

periphery. Enlarged vascular canals are present in the inner cortex due to secondary remodeling. No LAG/annulus was detected in the cortex. The medullary region is full of trabeculae.

Fig 2. Bone microstructures in juvenile *Lystrosaurus* IVPP V26544.

(A-D) bone microstructure in the tibia. (A) mid-diaphyseal cross-section. (B) partial cross-section under normal light. (C) partial cross-section showing the parallel-fibered bone under polarized light. (D) enlargement inner cortex of B under polarized light showing dense secondary osteons. (E-J) bone microstructure in the humerus. (E) bone microstructure under normal light. (F) the same region as E under polarized light. (G) outermost cortex under normal light showing a growth mark (arrow). (I) the same region as G under polarized light showing parallel-fibered bone. (H) inner cortex showing secondary osteons under normal light. (J) the same region as H under polarized light. (K-O) bone microstructure in the fibula. (K) bone microstructure under normal light. (L) bone microstructure under polarized light showing parallel-fibered bone. (M) enlargement of L showing the organized osteocyte lacunae. (N) the outermost cortex showing secondary osteons under normal light. (O) the innermost cortex showing endosteal bone under polarized light. **Abbreviations**: **end**, endosteal bone; **so**, secondary osteons.

The humerus is subtriangular in cross-section (midshaft diameter: 21×25 mm) and more robust than the tibia (Fig 2E, F). It has a thick compact bone (at least 7 mm) with an average cortical thickness of about 30%, and the free medullary cavity is extremely small. The cortex is comprised of fibrolamellar bone tissue, but a thin layer of parallel-fibered bone tissue is present near the periphery (Fig 2F-I). The bone porosity has a value of about 32%, which is higher than the tibia.

The primary osteons are predominantly plexiform and are longitudinally-oriented in the outer cortex. They are strongly reduced in size in the outer cortex. Parallel-fibered bone tissue and growth marks are present in the outermost region of the cortex (Fig 2G, I), suggesting slower growth rates when the individual died, but the osteocyte lacunae are still abundant. Many secondary osteons are seen in the inner cortex (Fig 2H-J). A thin layer of lamellar endosteal bone is deposited along the edges of the trabeculae in the medullary region (Fig 2F).

The fibula is much narrower than the tibia. The cross-section is oval in outline (midshaft diameter 6.5×4 mm) with a cortical thickness of about 25%. The medullary cavity is small with a diameter of about 2 mm. The cortex mainly consists of both fibrolamellar and parallel-fibered bone tissue (Fig 2K, L) and is less vascularized than the tibia and humerus, resulting in a lower bone porosity (22%). Osteocyte lacunae are abundant but are flattened and organized in the parallel-fibered bone (Fig 2M). The primary osteons are predominantly longitudinal and gradually decrease in size towards the periphery. Secondary osteons are mainly shown in the inner region, but there are also a few ones near the periphery (Fig 2N). A thin layer of endosteal bone is deposited in the innermost cortex (Fig 2O).

Subadult

The subadult stage was represented by IVPP V26542 and IVPP V26545. The cross-section of the femur of IVPP V26542 is elliptical with a diameter of 15×13 mm and an average cortical thickness of about 27% (Fig 3). The medullary cavity is large (the diameter is about 5 mm). The thickness of the cortex differs around the bone. The bone porosity is about 38% and the thickened region of the cortex has a higher porosity than that of the narrow region (Fig 3B). The

cortex mainly consists of fibrolamellar bone and is highly vascularized (Fig. 3B). The primary osteons show a laminar pattern that are mainly longitudinally oriented, but radially-oriented channels are found in a narrow channel in some places (Fig 3A). The number and diameter of the vascular canals include the porosity of the narrow region is significantly reduced near the periphery (Fig. 3B). The outer cortex consists of parallel-fibered bone matrix, and a LAG is present near the periphery (Fig 3C, D), suggesting reduced growth rates before it died. The secondary osteons are restricted in the innermost cortex (Fig 3E, F). Endosteal bone is present along the trabecular in the medullary cavity (Fig 3G).

Fig 3. Bone microstructures of the femur in subadult *Lystrosaurus* IVPP V26542.

(A) mid-diaphyseal transverse section of the femur. (B) enlargement of A showing highly vascularized cortical porosity. (C) enlargement of the outer cortex showing the LAG (arrow) under normal light. (D) the same region as C, showing parallel-fibered bone under polarized light. (E) the inner cortex showing secondary osteons under normal light. (F) the same region as E under polarized light. (G) the medullary region showing endosteal bone. **Abbreviations**: **end**, endosteal bone; **rc**, radial-oriented canals; **so**, secondary osteons

The cross-sections of the fibula, radius and rib of IVPP V26545 were sampled for observation. The cross-section of the fibula is subtriangular in outline (diameter: 12.4×9 mm) with an average cortical thickness of about 25% (Fig 4A, B). The cortex consists of a fibrolamellar bone. Most of the vascular canals are longitudinally arranged circumferentially around the bone, forming primary osteons. They are abundant in the inner region, but the number and size decrease from the

mid-cortex to the periphery, resulting in a low cortical porosity (18%). Secondary osteons are restricted to the inner cortex (Fig 4B), which consists of compacted coarse cancellous bone (CCCB). A distinct LAG comprised of several growth marks is present near the mid-region of the cortex (Fig 4B). The medullary region is filled with trabeculae (Fig 4A).

Fig 4. Bone microstructure in subadult *Lystrosaurus* IVPP V26545.

(A-B) bone microstructure of the fibula. (A) partial mid-diaphyseal transverse section under normal light. The arrow denotes a LAG. (B) the outermost cortex showing the LAG consisting of several growth marks (arrows). (C-E) bone microstructure of the radius. (C) partial outer cortex of the radius under normal light. (D) mid-cortex showing the LAG consisting of several growth marks under polarized light (arrows). (E), the same region as D under normal light. (F-G) the rib. (F) whole cross-section under normal light. (G) enlargement of the outer cortex showing a few vascular canals and a LAG. The arrows indicate LAGs. **Abbreviation**: **so**, secondary osteons.

The radius is more robust than the fibula with an average cortical thickness of about 26%. The cross-section of the radius is elliptical with a diameter of about 6×5 mm (Fig 4C). The cortex mainly consists of woven fibered bone and has a cortical porosity of about 20%. The vascular canals are primarily longitudinal but there are also many irregular canals. ~~Most of the vascular canals have formed primary osteons.~~ Secondary osteons and erosional cavities are present in the inner cortex. A clear LAG, as well as growth marks, are present at the mid-region as in the fibula (Fig 4D, E). Periosteally from the LAG the density of vascular canals is strongly reduced but are still abundant (Fig 4C). The medullary cavity is large with a diameter of about one third that of the whole 14

cross-section.

The cross-section of the rib is teardrop-shaped with a diameter of about 13×7 mm (Fig 4F, G). The compact bone is very thin (the average thickness is about 0.6 mm) due to a highly remodeled inner and mid-cortex. The medullary cavity is filled with trabeculae. The cortex consists of parallel-fibered bone tissues with only a few vascular canals (Fig 4G). The osteocyte lacunae are still abundant and disorganized. A clear LAG is present as in the fibula and radius (Fig. 4G).

Adult

The bone tissue of the femora of IVPP V26546 and IVPP V26547 shows adult tissues. In IVPP V26547, the midshaft of the femoral cortex is elliptical in outline with a diameter of 25×20 mm (Fig 5). The cortical thickness is about 25%. The cortical porosity is about 22% throughout, and the inner region is about 38% but is strongly reduced in the outer cortex (about 18.5%). There is no free medullary cavity. The cortex is composed of fibrolamellar bone throughout, and the outer cortex consists of parallel-fibered bone matrix with a thickness of about 1 mm (Fig 5E). Primary osteons are predominantly longitudinal and abundant throughout the cortex except for the region near the mid-cortex LAGs and the outer cortex. Secondary osteons are found

in the inner and mid-cortex regions. Two prominent LAGs are present in the mid-cortex (Fig 5D, E). The distance between these two LAGs is narrow (about 0.5 mm), and a thin layer of wide-open canals are present around the whole cross-section (Fig 5A, D). In the outermost cortex, the vascular canals are rare and nearly absent (Fig 5F), and two LAGs as well as growth marks are present near the periphery (Fig 5F-H). The osteocyte lacunae are flattened and organized along the growth lines (Fig 5H). The bone histology suggests that this individual grew very slowly

before it died, although it has no external fundamental system (EFS), which is present in modern mammals and some dinosaurs [36, 40].

Fig 5. Bone microstructure of the femur in adult *Lystrosaurus* IVPP V26547.

(A) mid-diaphyseal cross-section. (B) partial cross-section under normal light. (C) the same cortex as B under polarized light. (D) Enlargement of B under normal light showing the LAGs and parallel-fibered bone in the outer cortex. (E) the same cortex as D under polarized light showing LAGs and parallel-fibered bone. (F) the outermost cortex under normal light showing LAGs and vascular canals. (G) the same region as F showing parallel-fibered bone. (H) enlargement of F showing LAGs, grow mark, osteocyte lacunae and primary osteons. The arrows denote LAGs. **Abbreviations: gm,** growth mark; **wc**, wide-open canals.

In IVPP V26546, the femoral cortex is elliptical in cross-section. It has a thin compact bone with an average cortical thickness of about 15% and a large medullary region (Fig 6A). The cortex consists of fibrolamellar bone matrix except for the periphery. The vascular canals are relatively small and longitudinally-oriented in the outer cortex (Fig 6B). The cortical porosity is about 24% and strongly reduced in the outer cortex. Secondary osteons are common throughout the cortex and are also shown near the periphery (Fig 6E, F). A LAG with a narrow avascular layer of annuli is present in the mid-cortex (Fig 6B, C, G, H), and there are only a few vascular canals around. The osteocyte lacunae are flattened and organized within the annuli (Fig 6G, H). At least two LAGs are well present near the periphery (Fig 6C, D). The parallel-fibered bone matrix with LAGs and rare vascular canals suggests that it grew slowly before it died.

Fig 6. Bone microstructure in adult *Lystrosaurus* IVPP V26546 and IVPP V26548.

(A-H) bone microstructure of the femur of IVPP V26546. (A) the whole mid-diaphyseal cross-section. (B) partial transverse section under normal light. (C) the outermost cortex under polarized light showing parallel-fibered bone and LAGs near the periphery. (D) enlargement of the outermost cortex showing LAGs. (E) the outermost cortex showing secondary osteons under normal light. (F) the same region as E under polarized light. (G) enlargement of the annuli region showing the organized osteocyte lacunae. (H) the same region as G under polarized light. (I-J) the sectioned rib of IVPP V26548. (I) bone microstructure of the dorsal rib under normal light. (J) enlargement of the outermost cortex under normal light showing at least three LAGs. The arrows denote LAGs. **Abbreviations**: **anu**, annuli; **so**, secondary osteons.

In the largest specimen IVPP V26548, only one dorsal rib was sampled (Fig 6I). The cortex is strongly remodeled in the inner and mid-region, and even in the outermost cortex in some region. A thin layer of compact bone is shown in the outermost region. It is nearly avascularized with at least three LAGs near the periphery, which is similar to an EFS. However, these lines did not extend to the outermost region, and there are still several open vascular canals, suggesting this individual was still growing when died (Fig 6J).

Discussion

The bone tissues of *Lystrosaurus* is predominantly fibrolamellar bone with large channels as those from Africa and India [19, 27], and three growth stages were identified: a fast-growing

juvenile stage, a slower-growing subadult and an adult that grew very slowly. The fast growth rates may help them to adapt to the environment quickly [27]. The late juvenile was characterized by uninterrupted fibrolamellar bone tissue and the subadult was primarily fibrolamellar bone tissue interrupted by LAGs. The adult is nearly fully grown, which is characterized by the presence of parallel-fibered bone and closely spaced LAGs in the outermost cortex. Additionally, the bone tissue type of *Lystrosaurus* is not only related to the growth stages but also differs between the various limb bones, including the femur, tibia, fibula and radius. The humerus, femur and tibia show a mainly laminar to plexiform vascular arrangement with a high cortical porosity in all stages, whereas the vascular canals of the fibula and radius are most longitudinally oriented and reduced in density, resulting in a low cortical porosity. In addition, our samples support than the humerus and tibia have higher cortical porosity than that of other limb bones, but the parallel-fibered bone with growth marks near the periphery appeared earlier in humerus than in other elements (IVPP V26544).

Ray et al. (2005) found a parallel-fibered bone in the outer cortex of a dorsal rib and a thin layer of lamellar bone in a vertebra at the adult stage, but they did not find a typical EFS with closely spaced LAGs near the periphery. In our samples, the parallel-fibered bone in the outer cortex appeared in the dorsal rib and other elements, representing the subadult stage here (Fig 4). Although this is no typical EFS detected, the presence of more LAGs and parallel-fibered bone tissue near the periphery suggests that it grew very slowly before it died. Considering other dicynodonts have ~~detected~~ an EFS, such as t~~he~~ *Placerias* [41] and *Kannemeyeria* [42], *Lystrosaurus* may also have an EFS. Botha et al. (2016) found that all Early Triassic *Lystrosaurus* from South Africa have no more than one observed LAG and no parallel-fibered bone in the outermost cortex, which suggests that they ~~all~~ did not reach skeletal maturity [18]. However, LAGs seem to be more commonly distributed

in *Lystrosaurus* from the Early Triassic of China. Within the seven individuals, three have more than one LAG and two have one LAG for each. The adult stage of *Lystrosaurus* from China has a femur of about 15 cm and tibia about 12 cm, which is larger than all the samples taken from Ray et al. (2005). The presence of LAGs and the large size of *Lystrosaurus* from China suggest that many *Lystrosaurus* reached the ~~sub~~adult stage, unlike Triassic *Lystrosaurus* from Africa where ~~sub~~adults are rare. This difference may be caused by differing environmental conditions in these areas. The Karoo Basin and North China were geographically distant in the Early Triassic ~~and were supposed to be~~ located in ~~the Mid-high latitude in~~ the South and North hemisphere, respectively [43]. The Karoo Basin is thought to have been very arid in the Early Triassic based on sedimentological evidence [6, 7, 34]. The harsh environmental condition may have resulted in high mortality rates of *Lystrosaurus* and many individuals died early in ontogeny [18]. By comparison, the climate is thought to have been semiarid-subhumid in the Early Triassic of North China [31, 44, 45], which may have provided more suitable environmental conditions than that in South Africa.

Another important issue is whether the body size of *Lystrosaurus* was reduced in Early Triassic due to the harsh environment such as arid weather. Body size reduction has been shown in nonmammalian eutheriodont therapsids during the end-Permian mass extinction [10], and the reduction is also observed in *Lystrosaurus* from the Karoo Basin of South Africa, but this may be caused by the immaturity of the body based on bone microstructure [18]. Interestingly, the body size of *Lystrosaurus* from the lower section of the Guodikeng Formation such as *L. youngi* is relatively smaller than the upper section of Jiucaiyuan Formation, contrasting the condition in South Africa [29]. This may be because the material of *L.youngi* represents immature individuals, whereas most *Lystrosaurus* from Early Triassic ~~have~~ reached ~~the~~ subadult or adult stage. However, the bone

microstructure of *Lystrosaurus* from Guodikeng Formation is poorly preserved, and more material needs to be assessed in the future.

The lifestyle of *Lystrosaurus* was said to be aquatic/semiaquatic [19], fully terrestrial or burrowing [46, 47]. In recent decades, the controversy has concentrated on whether it is aquatic or borrowing, which could benefit its survival in the Early Triassic. The aquatic/semiaquatic lifestyle was supported by the bone microstructure showing a thick cortex and porous vascular canals [19], and ~~the~~ linear discriminant analysis based on the compactness profile and body size parameters in extant animals [48, 49]. The cortical thicknesses of all samples are between 15-30% here, which is similar to *Lystrosaurus* from South Africa [19]. However, the dorsal ribs have dense erosional cavities in the medial and mid-cortex and a thin layer of compact outer cortex (Figs. 4, 6), which differs from thick cortex (27-37%) in *Lystrosaurus* from Africa [19]. Therefore, the bone histology of *Lystrosaurus* described here provides weak evidence for an amphibious lifestyle. In addition, a thick cortex has also been reported in extant burrowing animals and nonmammalian therapsids [39]. Finally, juvenile *Lystrosaurus* has been found inside burrow casts indicating that they were active burrowers [21, 22]. The lifestyle of *Lystrosaurus* could be further elucidated by analyzing stable isotopes in the future. Previous studies suggest that aquatic and semi-aquatic vertebrates have $\delta^{18}O$ values significantly lower than those of coexisting terrestrial animals [50-52]. Oxygen and calcium isotopes support the hypothesis that spinosaurids were aquatic or semiaquatic like modern crocodiles [53, 54], and they could also provide more evidence for the lifestyle of *Lystrosaurus*.

Conclusion

Here we have studied the bone histology of *Lystrosaurus* from the Early Triassic of China

based on thin sections of seven individuals. The results indicate that *Lystrosaurus* grew rapidly in the early stages as previously shown in *Lystrosaurus* from South Africa and India. However, the presence of LAGs in the largest individual suggests that the growth of *Lystrosaurus* was interrupted in the late stage. ~~The number of~~ LAGs are rare in *Lystrosaurus* from South Africa but they are more abundant in the *Lystrosaurus* from Xinjiang, China. We propose that most *Lystrosaurus* from Xinjiang reached subadult and adult stages, whereas those from South Africa died during their juvenile stage. This distinction may be caused by different environmental conditions, but more samples from the Early Triassic of China are needed to test this hypothesis in the future.

Acknowledgment

We thank Zhang Shukang and Wu Rui for helping to make bone sections, Chu Daoliang for his helpful discussion. We thank Jennifer Botha, Megan Whitney, and Zoe Kulik for their comments on a previous edition of this paper.

References

1. Maxwell WD. Permian and Early Triassic extinction of non-marine tetrapods. Palaeontology. 1992;35(3):571-83.

2. Erwin DH. The Permo-Triassic extinction. Nature. 1994;367(6460):231-5.

3. Stanley SM. Estimates of the magnitudes of major marine mass extinctions in earth history. Proc Natl Acad SCI. 2016;113(42):E6325-E34. doi: 10.1073/pnas.1613094113

4. Retallack GJ. Permian and Triassic greenhouse crises. Gondwana Res. 2013;24(1):90-103. doi: org/10.1016/j.gr.2012.03.003.

5. Sun Y, Joachimski MM, Wignall PB, Yan C, Chen Y, Jiang H, et al. Lethally hot temperatures during the Early Triassic greenhouse. Science. 2012;338(6105):366-70. doi: 10.1126/science.1224126.

6. Smith RMH, Botha-Brink J. Anatomy of a mass extinction: Sedimentological and taphonomic evidence for drought-induced die-offs at the Permo-Triassic boundary in the main Karoo Basin, South Africa. Palaeogeogr Palaeoclimatol Palaeoecol. 2014;396(3):99–118.

7. MacLeod KG, Quinton PC, Bassett DJ. Warming and increased aridity during the earliest Triassic in the Karoo Basin, South Africa. Geology. 2017;45(6):483-6. doi: 10.1130/G38957.1

8. Bernardi M, Petti FM, Benton MJ. Tetrapod distribution and temperature rise during the Permian-Triassic mass extinction. Proc R Soc Lond B Biol Sci. 2018;285(1870):20172331. doi: doi:10.1098/rspb.2017.2331.

9. Song H, Tong J. Mass extinction and survival during the Permian-Triassic crisis. Earth Sci. 2016;41(6):901-18. (in chinese with English abstract)

10. Huttenlocker AK. Body size reductions in nonmammalian eutheriodont therapsids (Synapsida) during the end-Permian mass extinction. PLoS One. 2014;9(2):e87553. doi: 10.1371/journal.pone.0087553.

11. Chu D, Tong J, Song H, Benton MJ, Song H, Yu J, et al. Lilliput effect in freshwater ostracods during the Permian–Triassic extinction. Palaeogeogr Palaeoclimatol Palaeoecol. 2015;435:38-52. doi: https://doi.org/10.1016/j.palaeo.2015.06.003.

12. Smith RM, Ward PD. Pattern of vertebrate extinctions across an event bed at the Permian-Triassic boundary in the Karoo Basin of South Africa. Geology. 2001;29(12):1147-50. doi: org/10.1130/0091-7613(2001)029<1147:POVEAA>2.0.CO;2.

13. Smith R, Botha J. The recovery of terrestrial vertebrate diversity in the South African Karoo Basin

after the end-Permian extinction. C R Palevol. 2005;4(6-7):623-36. doi: org/10.1016/j.crpv.2005.07.005.

14. Colbert EH. The distribution of *Lystrosaurus* in Pangaea and its implications. Geobios. 1982;15:375-83. doi: org/10.1016/S0016-6995(82)80126-5.

15. Cosgriff JW, Hammer WR, Ryan WJ. The Pangaean Reptile, *Lystrosaurus maccaigi*, in the Lower Triassic of Antarctica. J Paleontol. 1982;56(2):371-85.

16. King GM, Jenkins I. The dicynodont *Lystrosaurus* from the Upper Permian of Zambia: Evolutionary and stratigraphical implications. Palaeontology. 1997;40(1):149-56.

17. Botha J, Smith RMH. *Lystrosaurus* species composition across the Permo-Triassic boundary in the Karoo Basin of South Africa: *Lystrosaurus* across the PTB. Lethaia. 2007;40(2):125-37. doi: org/10.1111/j.1502-3931.2007.00011.x.

18. Botha-Brink J, Codron D, Huttenlocker AK, Angielczyk KD, Ruta M. Breeding young as a survival strategy during earth's greatest mass extinction. Sci Rep. 2016;6(1):24053. doi: org/10.1038/srep24053.

19. Ray S, Chinsamy A, Bandyopadhyay S. *Lystrosaurus murrayi* (therapsida, dicynodontia): bone histology, growth and lifestyle adaptations. Palaeontology. 2005;48(6):1169–85. doi: org/10.1111/j.1475-4983.2005.00513.x.

20. Ray S, Mukherjee D, Bandyopadhyay S. Growth patterns of fossil vertebrates as deduced from bone microstructure: case studies from India. J Biosci. 2009;34(5):661-72. doi: org/10.1007/s12038-009-0055-x.

21. Botha-Brink J. Burrowing in *Lystrosaurus*: preadaptation to a postextinction environment? J Vert Paleontol. 2017;37(5):e1365080. doi: 10.1080/02724634.2017.1365080.

22. Modesto SP, Botha-Brink J. A burrow cast with *Lystrosaurus skeletal* remains from the Lower Triassic of South Africa. Palaios. 2010;25(4):274-81. doi: org/10.2110/palo.2009.p09-077r.

23. Tałanda M, DzięCioł S, Sulej T, NiedźWiedzki G. Vertebrate burrow system from the Upper Triassic of Poland. Palaios. 2011;26(2):99-105. doi: org/10.2110/palo.2010.p10-024r.

24. Hasiotis ST, Wellner RW, Martin AJ, Demko TM. Vertebrate Burrows from Triassic and Jurassic Continental Deposits of North America and Antarctica: Their Paleoenvironmental and Paleoecological Significance. Ichnos. 2004;11(1-2):103-24. doi: org/10.1080/10420940490428760.

25. Özkurt Ö, Yiğit N, Çolak E, Sözen M, Gharkheloo M. Observations on the Ecology, Reproduction and Behavior of *Spermophilus* Bennett, 1835 (Mammalia: Rodentia) in Turkey. Turk J Zool. 2005;29:91-9.

26. Chinsamy A, Rubidge BS. Dicynodont (Therapsida) bone histology: phylogenetic and physiological implications. Palaeontol Afr. 1993;30:97-102.

27. Botha-Brink J, Angielczyk KD. Do extraordinarily high growth rates in Permo-Triassic dicynodonts (Therapsida, Anomodontia) explain their success before and after the end-Permian extinction? Zool J Linn Soc. 2010;160(2):341-65. doi: org/10.1111/j.1096-3642.2009.00601.x.

28. Li J-L. *Lystrosaurus* of China. Vertebr Palasiat. 1988;26(4):241-9. (in Chinese with English abstract)

29. Liu J, Li J, Cheng Z. The *Lystrosaurus* fossils from Xinjiang and their bearing on the terrestrial Permian Triassic boundary. Vertebr Palasiat. 2002;40(4):267-75. (in Chinese with English abstract)

30. Camp J, and Liu J. 2011. The taxonomy and cranial morphology of Chinese *Lystrosaurus*. [Abstract] Supplement to online Journal of Vertebrate Paleontology, 71st Annual Meeting Society of Vertebrate Paleontology: 82

31. Yang W, Feng Q, Liu Y, Tabor N, Miggins D, Crowley JL, et al. Depositional environments and cyclo- and chronostratigraphy of uppermost Carboniferous–Lower Triassic fluvial–lacustrine deposits, southern Bogda Mountains, NW China — A terrestrial paleoclimatic record of mid-latitude NE Pangea.

Global Planet Change. 2010;73(1):15-113. doi: org/10.1016/j.gloplacha.2010.03.008.

32. Thomas SG, Tabor NJ, Yang W, Myers TS, Yang Y, Wang D. Palaeosol stratigraphy across the Permian–Triassic boundary, Bogda Mountains, NW China: Implications for palaeoenvironmental transition through earth's largest mass extinction. Palaeogeogr Palaeoclimatol Palaeoecol. 2011;308(1):41-64. doi: org/10.1016/j.palaeo.2010.10.037.

33. Wan M, Yang W, Wang JJ. A new *Protophyllocladoxylon* wood from the Induan (Lower Triassic) Jiucaiyuan Formation in the Turpan–Hami Basin, southern Bogda Mountains, Rev Palaeobot Palynol. northwestern China. 2019;267:62-72.

34. Botha J, Huttenlocker AK, Smith RMH, Prevec R, Viglietti P, Modesto SP. New geochemical and palaeontological data from the Permian-Triassic boundary in the South African Karoo Basin test the synchronicity of terrestrial and marine extinctions. Palaeogeogr Palaeoclimatol Palaeoecol. 2020;540:109467. doi: org/10.1016/j.palaeo.2019.109467.

35. Horner JR, de Ricqlès A, Padian K. Variation in dinosaur skeletochronology indicators: implications for age assessment and physiology. Paleobiology. 1999;25(3): 295-304. doi: org/10.1017/S0094837300021308.

36. Chinsamy-Turan A. The microstructure of dinosaur bone. Baltimore: Johns Hopkins University Press; 2005. 195 p.

37. Donath K, Rohrer M. Bone Sectioning Using the Exakt System. In: An YH, Martin KL, editors. Handbook of Histology Methods for Bone and Cartilage: Springer; 2003. pp. 243-52.

38. Francillon-Viellot H, de Buffrénil V, Castanet J, Géraudie J, Meunier FJ, Dire JY, et al. Microstructure and mineralization of vertebrate skeletal tissues. In: Carter JG, editor. Skeletal biomineralization: patterns, processes and evolutionary trends. New York: Van Nostrand Reinhold; 1990.

p. 471-530.

39. Chinsamy-Turan A. The microstructure of bones and teeth of nonmammalian therapsids. In: Chinsamy-Turan A, editor. Forerunners of mammals: radiation, histology, biology. Bloomington, Indiana: Indiana University Press; 2012. p. 65-90.

40. Horner JR, De Ricqles A, Padian K. Long bone histology of the hadrosaurid dinosaur *Maiasaura peeblesorum*: Growth dynamics and physiology based on an ontogenetic series of skeletal elements. J Vert Paleontol. 2000;20(1):115-29. doi: org/10.1671/0272-4634(2000)020[0115:LBHOTH]2.0.CO;2.

41. Green JL, Schweitzer MH, Lamm ET. Limb bone histology and growth in *Placerias hesternus* (Therapsida: Anomodontia) from the Upper Triassic of North America. Palaeontology. 2010;53(2): 347-64. doi: org/10.1111/j.1475-4983.2010.00944.x.

42. Ray S, Botha-Brink J, Chinsamy-Turan AJ. Dicynodont growth dynamics and lifestyle adaptations. In: Chinsamy-Turan A, editor. The forerunners of mammals: radiation, histology and biology. Bloomington: Indiana University Press; 2012. p. 121-48.

43. Muttoni G, Gaetani M, Kent DV, Sciunnach D, Angiolini L, Berra F, et al. Opening of the Neo-Tethys Ocean and the Pangea B to Pangea A transformation during the Permian. GeoArabia. 2009;14(4):17-48.

44. Liu D, Zhang C, Yang D, Pan Z, Kong X, Huang Z, et al. Petrography and geochemistry of the Lopingian (Upper Permian)-Lower Triassic strata in the southern Junggar and Turpan basins, NW China: implications for weathering, provenance, and palaeogeography. Int Geol Rev. 2019;61(8):1016-36. doi: org/10.1080/00206814.2018.1490932.

45. Tabor NJ, Sidor CA, Smith RMH, Nesbitt SJ, Angielczyk KD. Paleosols of the Permian-Triassic: proxies for rainfall, climate change and major changes in terrestrial tetrapod diversity. J Vert Paleontol.

2017;37(sup1):240-53. doi: org/10.1080/02724634.2017.1415211.

46. King GM, Cluver MA. The aquatic *Lystrosaurus*: An alternative lifestyle. Hist Biol. 1991;4(3-4):323-41. doi: org/10.1080/08912969009386548.

47. King GM. The aquatic *Lystrosaurus*: a palaeontological myth. Hist Biol. 1991;4(3-4):285-321.

48. Germain D, Laurin M. Microanatomy of the radius and lifestyle in amniotes (Vertebrata, Tetrapoda). Zool Scr. 2005;34(4):335-50. doi: org/10.1111/j.1463-6409.2005.00198.x.

49. Canoville A, Laurin M. Evolution of humeral microanatomy and lifestyle in amniotes, and some comments on palaeobiological inferences. Biol J Linn Soc. 2010;100(2):384-406. doi: org/10.1111/j.1095-8312.2010.01431.x.

50. Bocherens H, Koch PL, Mariotti A, Geraads D, Jaeger J-J. Isotopic biogeochemistry (^{13}C, ^{18}O) of mammalian enamel from African Pleistocene hominid sites. Palaios. 1996;11(4):306-18.

51. Fricke HC, Rogers RR. Multiple taxon–multiple locality approach to providing oxygen isotope evidence for warm-blooded theropod dinosaurs. Geology. 2000;28(9):799-802.

52. Clementz MT, Holroyd PA, Koch PL. Identifying aquatic habits of herbivorous mammals through stable isotope analysis. Palaios. 2008;23(9):574-85.

53. Hassler A, Martin J, Amiot R, Tacail T, Godet FA, Allain R, et al. Calcium isotopes offer clues on resource partitioning among Cretaceous predatory dinosaurs. Proc R Soc Lond B Biol Sci. 2018;285(1876):20180197. doi: org/10.1098/rspb.2018.0197.

54. Amiot R, Buffetaut E, Lécuyer C, Wang X, Boudad L, Ding Z, et al. Oxygen isotope evidence for semi-aquatic habits among spinosaurid theropods. Geology. 2010;38(2):139-42. doi: org/10.1130/G30402.1.

Supporting information

S1 Fig. The accurate sampled positions of all specimens in this study. A. IVPP V26543, arrow denotes the sampled position of the tibia; **B, C**. IVPP V26544. B, the sampled positions of the tibia and fibula; C, the sampled position of the humerus; **D**, IVPP V26542, the distal end of the femur that sampled; **E-G.** IVPP V26545. E, The sampled position of the radius; F, the sampled position of the fibula; G. The sampled position of the rib; H. IVPP V26547, arrow denotes the sampled position of the femur; **I**, IVPP V26546, arrow denotes the sampled position of the femur; **J**. IVPP V26548, arrow denotes the sampled position of the rib.

Figure 1

Click here to download Figure Figure 1.tif

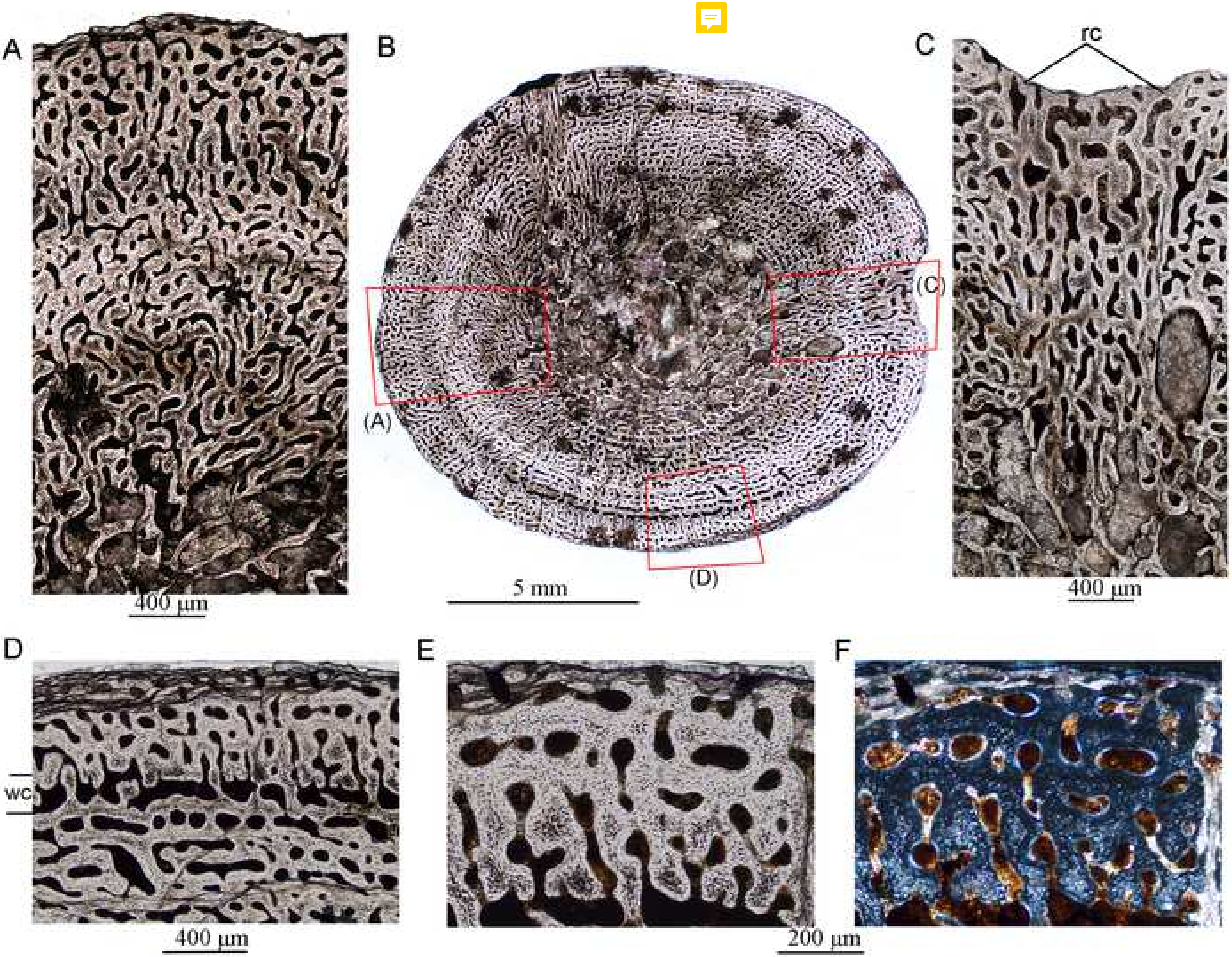

Figure 2

Click here to download Figure Figure 2.tif

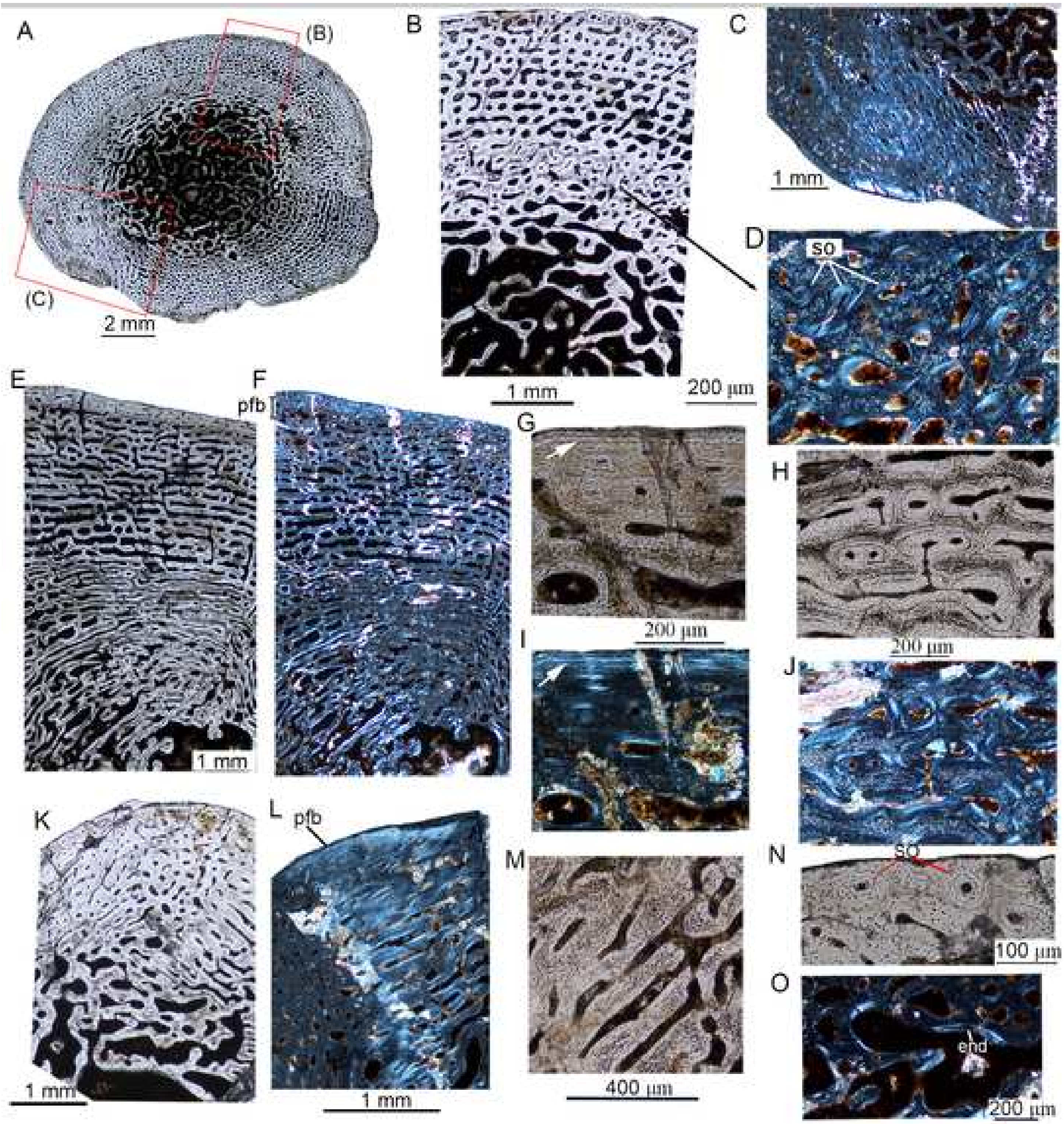

Figure 3

Click here to download Figure Figure 3.tif

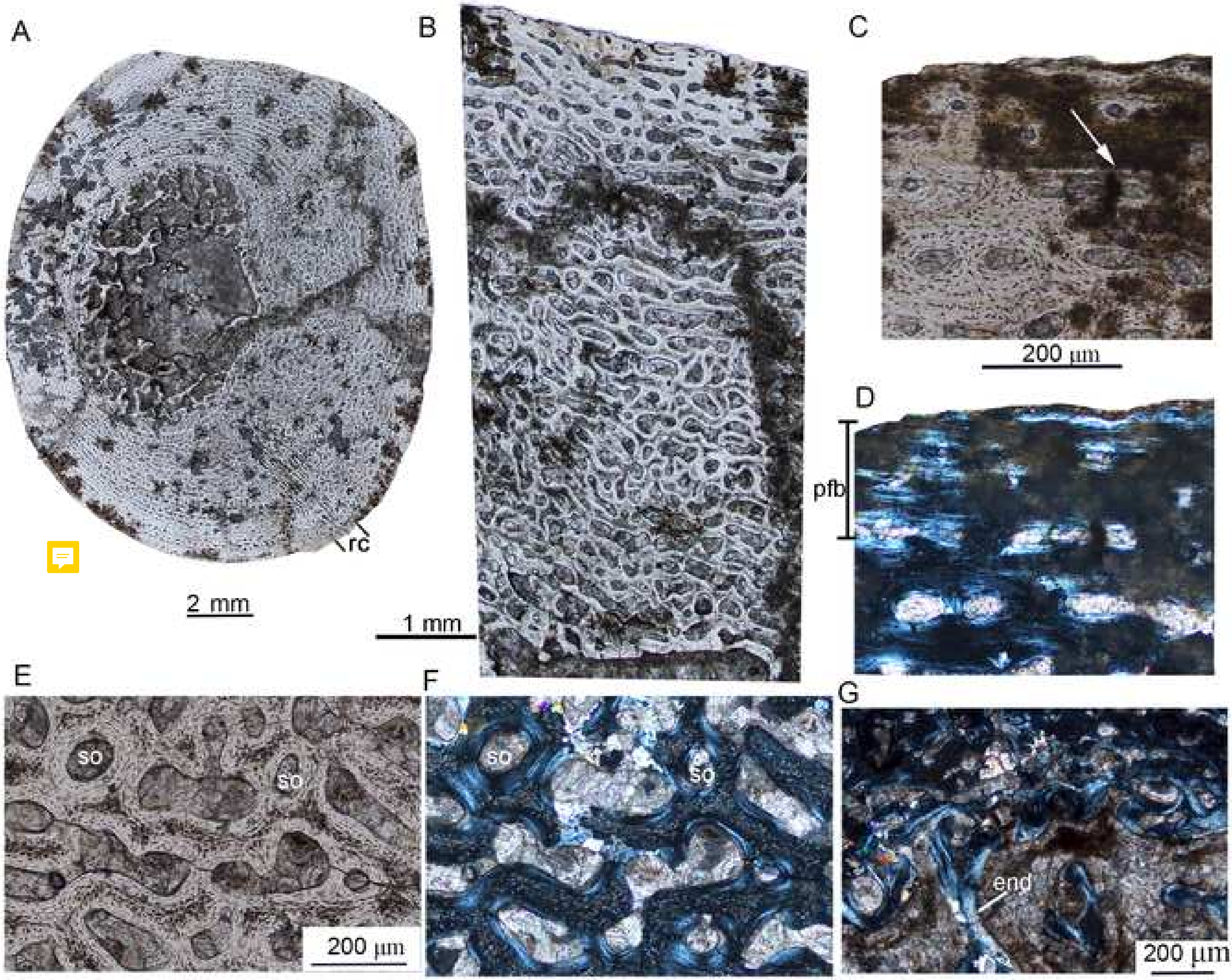

Figure 4

Click here to download Figure Figure 4.tif

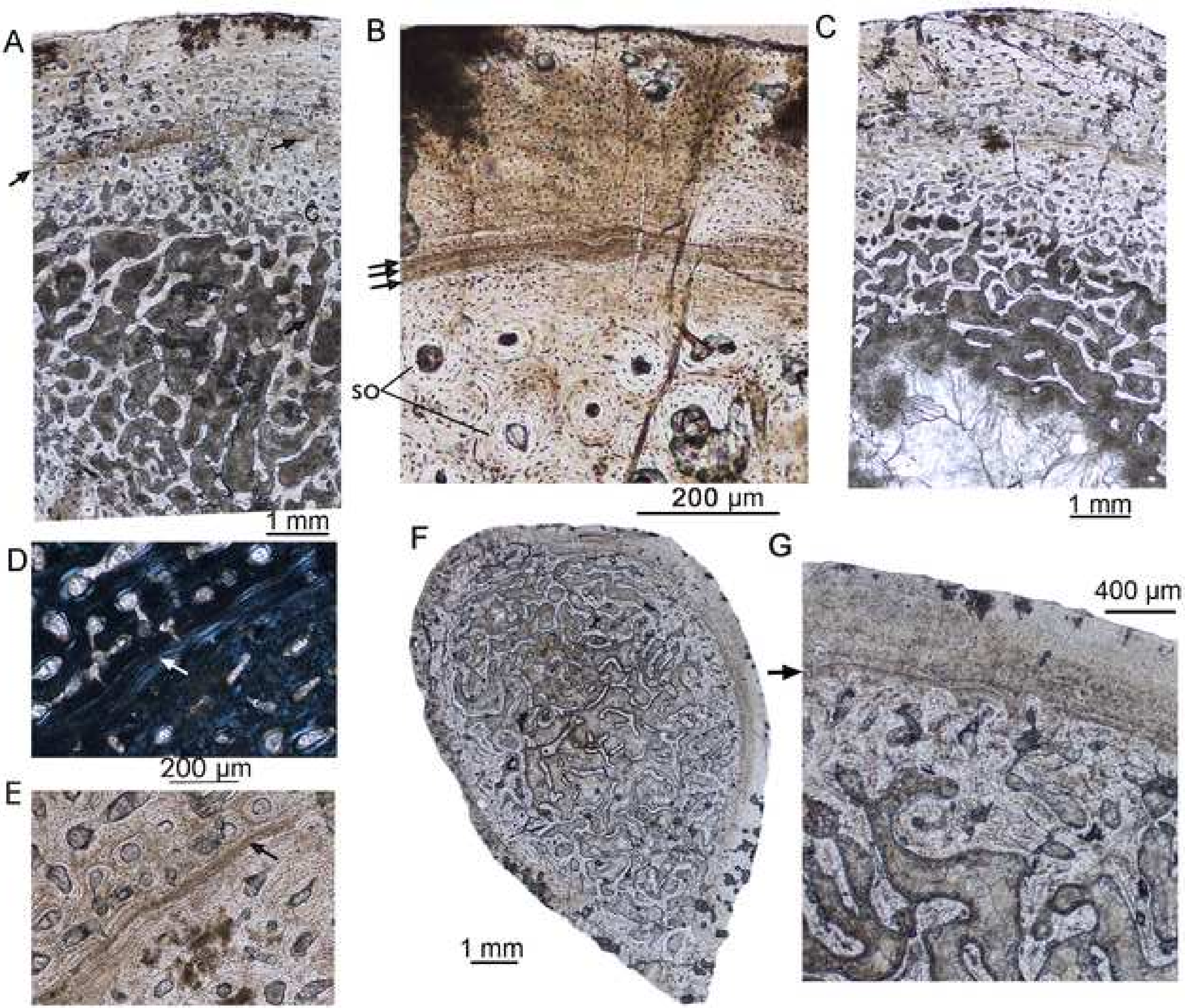

Figure 5

Click here to download Figure Figure 5.tif

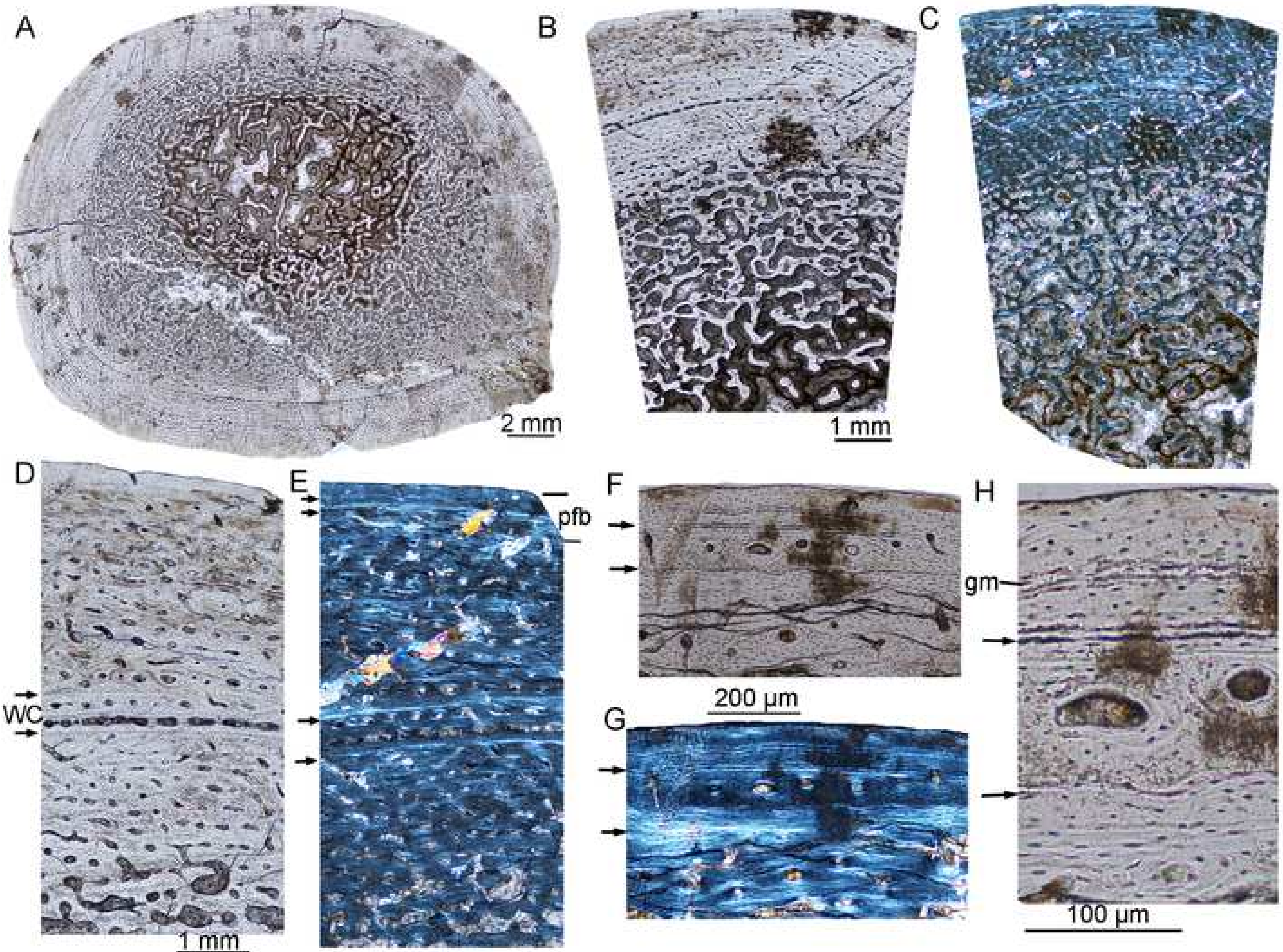

Figure 6

Click here to download Figure Figure 6.tif

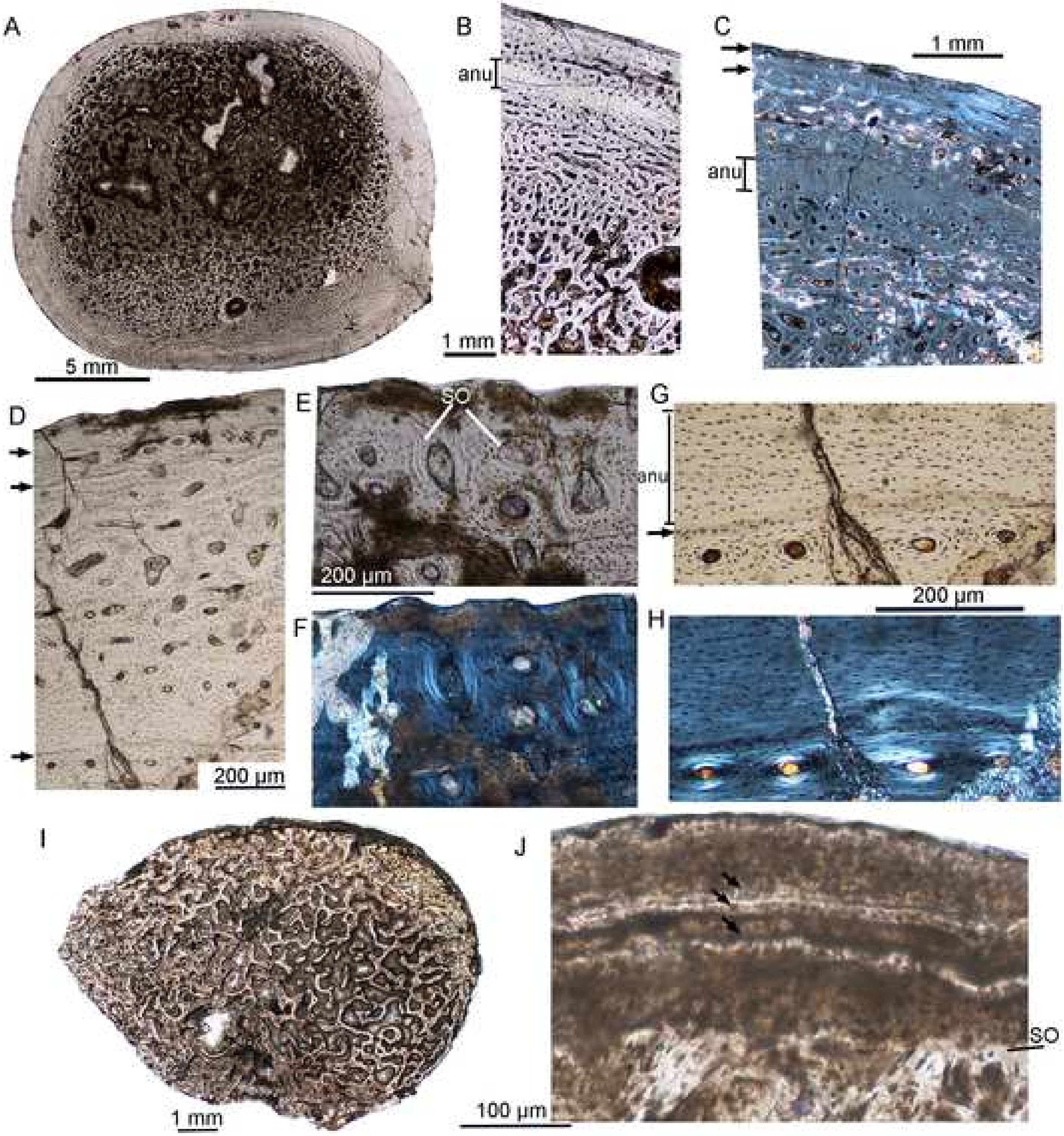

Supplement: Attachment — Submitted filename: PONE-D-20-17684_COMMENTS.pdf [file pone.0248681.s007.pdf]
