# Supplementary material for: Preliminary bone histological analysis of Lystrosaurus (Therapsida: Dicynodontia) from the Lower Triassic of North China, and its implication for lifestyle and environments after the end-Permian extinction
Source: PLoS One. 2021 Mar 18;16(3):e0248681. doi: 10.1371/journal.pone.0248681 (PMC7971864; doi:10.1371/journal.pone.0248681)
Supplement: Attachment — Submitted filename: PONE-D-20-17684_R1_reviewed.pdf [file pone.0248681.s009.pdf]

PLOS ONE

Bone histology of Lystrosaurus (Therapsida: Dicynodontia) from the Lower Triassic of North China, and its implication for lifestyle and environments after the end-Permian extinction

--Manuscript Draft--

**Manuscript Number:**	PONE-D-20-17684R1
**Article Type:**	Research Article
**Full Title:**	Bone histology of Lystrosaurus (Therapsida: Dicynodontia) from the Lower Triassic of North China, and its implication for lifestyle and environments after the end-Permian extinction
**Short Title:**	Bone histology of Lystrosaurus from North China
**Corresponding Author:**	Fenglu Han, Ph.D<br>China University of Geosciences (Wuhan)<br>Wuhan, CHINA
**Keywords:**	Bone Histology; Lystrosaurus; Early Triassic; China
**Abstract:**	Lystrosaurus represents one of the most successful dicynodonts, a survivor of the end-Permian mass extinction that remained abundant in the Early Triassic, but many aspects of its paleobiology are still controversial. The bone histology of Lystrosaurus species from South Africa and India has provided important information on their growth strategy and lifestyle, but until recently no data was available on the bone histology of Lystrosaurus from China. Here, we report on the bone microstructure of seven Lystrosaurus individuals from the Lower Triassic of Xinjiang, providing the first such data for the Chinese Lystrosaurus species. Our samples indicate that the microstructure of Lystrosaurus limb bones from China is characterized by fibrolamellar bone tissue similar to those from South Africa and India. Three ontogenetic stages were identified: juvenile, subadult, and adult based on lines of arrested growth (LAGs) and bone tissue changes. Bone histology supports a rapid growth strategy for Lystrosaurus during early ontogeny. Unlike Early Triassic Lystrosaurus from South Africa, lines of arrested growth are common in our specimens, suggesting interruption in growth in Lystrosaurus from China. The differences in bone histology between Lystrosaurus from South Africa and China may indicate different environmental conditions in these two regions.
**Order of Authors:**	Fenglu Han, Ph.D
	Qi Zhao
	Jun Liu
**Response to Reviewers:**	Dear Editor and Reviewers:<br>Thank you for your letter and for the reviewers' comments concerning our manuscript entitled "Bone histology of Lystrosaurus (Therapsida: Dicynodontia) from the Lower Triassic of North China, and its implication for lifestyle and environments after the end-Permian extinction" (ID: PONE-D-20-17684). Those comments are all valuable and very helpful for revising and improving our paper, as well as important for highlighting the significance of our work. We have studied the comments carefully and made a number of corrections to address them, which we hope to meet with approval. The revised portions of the MS were marked by red colors. The main corrections to the paper and the responses to the editor and reviewer's comments are as following:<br>Editor comments:<br>Please pay detailed attention to the comments made by ALL reviewers but specifically to the comments and criticism of Reviewer 4. For this manuscript to be acceptable for publication in Plos One, it is essential that you provide additional comparative data with regards to South African Lystrosaurus specimens as well as reconsider the interpretation of your own data, specifically with respect to the evidence for an external fundamental system (EFS), which is rather unconvincing based on the currently provided data.<br><br>REPLY: We have modified our MS carefully according to all reviewers' comments. We

Powered by Editorial Manager® and ProduXion Manager® from Aries Systems Corporation

have also added more comparisons between Chinese Lystrosaurus and South African Lystrosaurus, including both bone microstructure and body size. In addition, We have deleted the statement of the possible presence of EFS in Lystrosaurus based on current evidence, and we just mentioned that "there is still no typical EFS found in Lystrosaurus".

Reviewer1
introduction line 4: It might be good to note that there is some debate about this, at least for the extinction in the Karoo Basin, such as Gastaldo et al. (2020; Journal of Sedimentary Research) and references cited therein

REPLY: We have added the debate and related references in the MS.

page 3 line 2: instead of curve, to would recommend using downturned, since the shape is more angular than curved in most Lystrosaurus species.

REPLY: Corrected.

page 3 line 3: The report of Lystrosaurus in Zambia is incorrect (See Angielczyk et al. 2014; Early Evolutionary History of the Synapsida book), so I recommend not citing the King and Jenkins paper. Frobisch (2009; Earth-Science Reviews) would be a more comprehensive paper to cite here (although, it still includes the incorrect Zambian record).

REPLY: We have changed the citation as suggested.

page 3 line 6: It might be worth noting that the semi-aquatic ecology of Lystrosaurus has been disputed including using bone histology data (e.g., King 1991; King and Cluver 1991, both Historical Biology; Botha-Brink and Angielczyk 2010, ZJLS).

REPLY: We have added more citations here.

page 3, paragraph 2: A couple comments here. 1) remove 'the' before minaturization. 2) I suggest a little re-phrasing of the results from the Botha-Brink et al. (2016) paper. That paper did indicate that LAGs were more common in Permian specimens, which was interpreted as evidence that the Permian species had a more extended ontogeny that extended over several years. This contrasts with the prevalence of small individuals with few or no LAGs in the Triassic species, which was part of the evidence suggesting breeding young (the other part of the evidence were the demographic simulations that suggested that breeding young could be an effective survival strategy when life expectancy was low).

REPLY: Modified as suggested.

material and methods line 6: I'm not sure if the specimens you sectioned had associated skulls. If they do not, I recommend saying that ID to species level is currently not possible because Chinese Lystrosaurus taxonomy (and Lystrosaurus taxonomy in general) is currently based on skull characters. If they have skulls, you might say that you didn't try to ID them to species level given the uncertainties about Chinese Lystrosaurus species-level taxonomy that you mentioned above.

REPLY: Thank you for your suggestion. Our specimens only include postcranial materials. Modified as suggested.

material and methods line 8: Morphology refers to macroscopic features, whereas histology refers to microscopic tissue structure. So you should say: "...South Africa is quite similar, although more LAGs..." You should also make this change in the next sentence when you make your predictions about the Chinese species.

REPLY: Corrected. Furthermore, we moved this part to the discussion as suggested by another reviewer.

material and methods line 10: Identical histology might be too strong. Similar is

probably a better prediction, assuming there aren't differences due to environmental conditions (as you note).
REPLY: Modified as suggested.

Materials and methods, paragraph 2, line 3: complete instead of completed; also at the end of this sentence, change bones to bone. It would be good to note whether and how the specimens were documented before sectioning (e.g., e.g., if photographs or measurments were taken, if molds/casts were made, etc.).

REPLY: We have added this information in paragraph 2.

Table 1: How did you assign your specimens to growth stages? Is this something that was done a priori, and if so what was it based on (Size? Degree of ossification and development of muscle scars and articular surfaces? Something else?). You should include this information in the methods section.

REPLY: Growth stages were identified based on bone histological information, and we have added this information in the footnotes of Table 1.

Page 6, paragraph 1: This paragraph uses a mixture of present tense and past tense. Please revise it so it consistently uses past tense.

REPLY: Corrected.

Page 6, line 2-3: You should state why it was difficult to measure cortical porosity for a whole cross section. Was it because of breakage, differential preservation of the bone tissue in different areas, something else? Also, It might be good to elaborate a bit on how you chose areas to measure when that was necessary, maybe the average number of areas you needed to use (in addition to the maximum you report), and whether using different numbers of areas had any effect on the results.

REPLY: We have added this information and modified the sentence as "It was difficult to obtain the cortical porosity of a whole cross-section for each specimen because of partially breakage or poor preservation in some areas of these sections."

Cortical porosity is usually reduced from the inner region to outside, but they are usually (not always) similar on all sides (medial, lateral, anterior, posterior). We subsampled ten areas from the inner to the outermost cortex in all sections (except IVPP V26546 was selected 12 areas) to calculate average vascular porosity. The sampled areas were chosen from the inner region to the periphery on different parts of the whole cross-sections, so they could reflect the value of cortical porosity. However, some bone sections show unclear bone histology in some regions, which may affect the results. Cortical porosities of those poorly preserved bone sections were labeled to be "estimated values". We have provided the figures showing the sampled area (S3 Fig).

Fig. 1: I recommend reorganizing this figure slightly so that the overview of the specimen showing the focal areas is in panel A (similar to Fig. 2).

REPLY: Modified as suggested.

juvenile line 2: change to: All the corticies are composed of a woven-fibered bone matrix, with a…

REPLY: Corrected. We have modified this sentence as "All the cortices are composed of fibrolamellar tissue, with a high number of vascular canals, abundant globular osteocyte lacunae, and no LAGs, suggesting a rapid, constant growth rate."

juvenile, line 5: when you mention cortical thickness and porosity values here and elsewhere in the text, you should call out Table 1. It would also be good if you can be more consistent in mentioning those values for the different elements in the text.

REPLY: We have added this information throughout the manuscript.

Fig. 2 and Fig. 2 caption: H and I should be switched in the figure in the caption given that you discuss what is currently I (but should be H) first. Also, panel O is a little dark. Can you adjust the brightness a bit on the image?

REPLY: Modified as suggested.

Fig. 3: Please add boxes to the overview image in panel A to show where the higher magnification images are located.

REPLY: Added.

Page 13, line 3: I think you mean: "The number and diameters of the vascular canals in the narrow region..."

REPLY: Thank you for pointing out. Corrected.

Fig. 5 : Please add boxes to the overview image in panel A to show where the higher magnification images are located.

REPLY: Added.

Page 16, line 2: An EFS was reported in the dicynodont Placerias by Green et al. (2010; Palaeontology).

REPLY: We have added more references here.

Fig. 6 : Please add boxes to the overview image in panel A to show where the higher magnification images are located.

REPLY: We have added a box in panel A, showing the position of B, and also a box in panel B, showing the position of C.

page 18, first full paragraph, line 7: Bone histology has been looked in a pretty wide range of dicynodonts now, and an EFS has only been observed in a couple of instances. Therefore, it seems odd to suggest that it may be present in Lystrosaurus, especially when you didn't find any evidence of one in any of your specimens.

REPLY: We have modified this paragraph and conclusion here. The modified statement is "There is still no typical EFS detected in Lystrosaurus."

Page 19, line 9: As noted above, some research has suggested wetter conditions in the Karoo at this time. It would be good to note that possibility here as well.

REPLY: we have added new references on wetter conditions.

Page 19, last line: It might be good to qualify this a bit by stating that most collected Early Triassic specimens are at least subadults. My sense is that Lystrosaurus hasn't been documented in a comprehensive enough way to have an unbiased sense of the relative abundances of different size classes.

REPLY: Modified as suggested.

Page 20, first full paragraph: The other thing that's important to note here is that the bone microstructure of Lystrosaurus doesn't seem to differ all that much from other dicynodonts (e.g., Botha-Brink and Angielczyk 2010). So either that would imply that most dicynodonts were semi-aquatic or that Lystrosaurus was terrestrial like other dicynodonts (with dicynodonts in general showing some fairly consistent differences in microstructure to modern terrestrial tetrapods).

REPLY: Our samples support Lystrosaurus has a thick cortex, as in Lystrosaurus of Africa. But thick cortex is not only seen in aquatic animals but also shown in extant burrowing animals. Therefore, thick cortex could not provide confident evidence of the

Powered by Editorial Manager® and ProduXion Manager® from Aries Systems Corporation

aquatic or semiaquatic lifestyle of Lystrosaurus, so we did not discuss the lifestyle of other dicynodonts. We have provided more discussion on Lystrosaurus lifestyle and how to solve this problem.

Page 20, first full paragraph, next to last sentence: It would be good to cite Rey et al. (2020; Palaeo3) here since it is a study that uses stable isotope data to make inferences about habitat preferences of a diynodont.
REPLY: Thank you for the information. Cited.

Reviewer 2.
There are some critical flaws that I think could only be addressed with an adequate comparative dataset of Permian Lystrosaurus specimens, and it's unclear to me whether the research team has adequately surveyed Permo-Triassic therapsid histology at all. Based on the abundant material of Permo-Triassic Lystrosaurus that I've thin-sectioned and seen firsthand, the South African material is nearly identical to the Chinese specimens. This study is hindered by a lack of any comparative specimens, and would further benefit from a more quantitative scientific approach.

REPLY: Thank you for your suggestions. We have added more comparison to Lystrosaurus from South Africa on both bone histology and body sizes. We admit that our samples are limited, but they do show some interesting and clear features that unlike those of Lystrosaurus from South Africa, and we will provide more data on bone histology and other parts of Chinese Lystrosaurus in the future.

Reviewer #3: The frequent presence of LAGs in the Chinese specimens is interesting and well worth publishing. I have made a number of copy edits and comments on some unclear parts of the manuscript in the attached file, which should be addressed before this contribution can be accepted.

REPLY: Thank you for your comments and detailed corrections for the grammar in the MS. We have checked the whole text and corrected all these things. Here are our replies to the main comments.

Page 4 Line 72. “Therefore, here we suppose that Lystrosaurus from the Taodonggou area of Xinjiang China should have identical morphology on bone histology even in different species. However, they may have distinct bone histological information from other places due to different environmental conditions”
Comment: This statement is unclear. Why should they have identical morphology?

REPLY: we have changed the word “identical” to “similar”. And moved this part to the discussion section. Here we mean that the previous work on Africa Lystrosaurus shows similar bone microstructure in different species, and Chinese Lystrosaurus may also have similar bone microstructure even in different species.

Page 6 Line 98. “Therefore, we subsampled areas (as many as ten) from the inner to the outermost cortex to calculate average vascular porosity.”
Comment: What is the minimum?

REPLY: We subsampled ten areas in all the sections. We have provided the sampled areas on photographs in the new supplementary files (S3 Fig).

Page 11. Line 206 The number and diameter of the vascular canals in the narrow region is significantly reduced near the periphery.
COMMENT: This does not make sense to me.

REPLY: Thank you for pointing out. We have modified this sentence to “The number and diameter of the vascular canals are significantly reduced near the periphery.”

Page 12. Line 210 “Trabeculae? In the trabecular region?”
REPLY: Yes. We have corrected this word.

Page 19 Recent papers by Kevin Rey on South African oxygen isotope work in dicynodonts would be worth citing and possibly discussing here.

Powered by Editorial Manager® and ProduXion Manager® from Aries Systems Corporation

REPLY: Thank you for your useful information. We have added this new reference here.

Reviewer #4:
This study describes the bone histology of seven Early Triassic Lystrosaurus from Xinjiang, China. The authors describe an ontogenetic series of rapid growth, with some periods of interrupted growth, and eventually a slowed, adult-stage growth. The authors compare these findings to those from the bone histology of South African Lystrosaurus and detail that although signals of arrested growth are rarely reported from South Africa, they were found in their sample. This suggests that perhaps the samples from South Africa are still in the juvenile/subadult stage. The authors suggest that this finding could be explained by environmental conditions that are thought to have been more extreme in South Africa than in China during the Early Triassic resulting in a greater die-off of individuals before skeletal maturity in South Africa. The authors also discuss their findings as it relates to the lifestyle of Lystrosaurus, refuting previous hypotheses that the genus was aquatic/semiaquatic.

I have reviewed a previous version of this manuscript and found the grammar and organization to be much improved. There were still some line edits of which I have added to my marked version of the manuscript. I have also added comments that are both specific and general to this document, but in addition, I have a few general comments about the manuscript. I think the figures are incredibly well presented and it is important this data is published. However, I think there are very major revisions in the discussion points/conclusions as well as some methodology that requires attention prior to publication.

Generally, I think the authors need to make a more compelling argument that their sample represents an ontogenetic series, independent of the histology. Table 1 I believe is supposed to address this, but the organization is difficult for the reader to follow, especially when I can't compare a single element across the sample. To address this I think the authors, using more complete specimens and previously established ratios between elements, could come up with estimates of a single element for each individual in their sample. Alternatively, a single figure that images all of the samples at the same scale would help the reader confirm the authors' designations of ontogenetic stages. I believe there is a supplementary figure, however, this figure was not included in my review document so I am unable to say if it would help to understand the relative sizes of these individuals. This is the only reason why I answered that not all data was available. In such a figure, I would also include images of the largest South African and Indian Lystrosaurus elements so that the reader can see just how much larger the Chinese specimens are.

REPLY: Thank you for your suggestions. Sorry for unmodified this part last time. The postcranial skeleton of Lystrosaurus was not well studied, and we did not know the variation between different species and ontogeny, so we did not estimate values previously. We have rechecked more Lystrosaurus materials and provided estimated values based on element ratios of one complete skeleton (IVPP RV35012), please see the footnotes of Table 1 for detail information.

In addition, sorry that we forgot to upload figure S1. In this figure, we have provided the accurate sampled positions of each element.

The methodology still requires significant revision/clarity. I would review previous histological papers that measure cortical thickness. I think for cortical thickness, presenting a range is more important that an average, especially in some specimens that are asymmetrically arranged. Cortical porosity also needs to be addressed. The description as it stands does not seem systematic and completely subjective. Subsampling vascularity is ok, but the locations (medially, laterally, posterior, anterior, endosteally, mid-cortex, periosteally) need to be systematically sampled similarly across the entire sample. Without this, it is difficult to review the values reported.

REPLY: We have provided ranges for all cortical thickness and labeled the measurement positions in S2 Fig.

We have added more descriptions of how to measure cortical porosity. We choose ten

areas that cover all the cortex. The sampled areas were from the inner region to the periphery on different parts of the whole cross-sections, and it indicates the average cortical porosity of the whole cross-section. Some bone sections show unclear bone histology in some regions, which may affect the results. Cortical porosities of those poorly preserved bone sections were labeled to be “estimated values”. We have also provided the figures showing the areas that we choose. Please see S3 Fig.

In the descriptions differences in cortical porosity need to be explicitly stated. There are many instances where the percentage for one region (e.g. endosteal) of the cortex is reported and then the authors state there is a reduction in vascularity towards the periosteal surface, but no percentage is reported. Consistency is important in interpreting the descriptions.

REPLY: We have added more percentages to make it clear.

It is important that the authors describe the anatomical context for the images (i.e. medial, lateral sides etc.). This can be done in the image itself (e.g. compass-style arrows) or in the figure caption.

REPLY: We checked all the specimens again and have added the directions for all the figures.

Finally, I do not think the data here is sufficient to refute the hypothesis that Lystrosaurus was aquatic. I agree entirely that it is unlikely Lystrosaurus was not aquatic, however, I don’t believe the data as currently presented in this manuscript supports this. In fact, the thick cortical walls appear very similar to aquatic taxa, which is why previous studies have suggested an aquatic lifestyle. However, there are studies that demonstrate this is not always a reliable proxy for lifestyle (e.g. Houssaye et al. 2016). I would recommend one of two courses of action. Either remove the discussion of the aquatic lifestyle from this manuscript OR discuss how cortical thickness does not always accurately predict habitat.

REPLY: Sorry for the confusion. We did not refute the hypothesis that Lystrosaurus was aquatic. We prefer to keep this paragraph and have added more discussion here. Most of our samples support Lystrosaurus has a thick cortex, as in Lystrosaurus of Africa. But thick cortex is not only seen in aquatic animals but also shown in extant burrowing animals. Therefore, thick cortex could not provide confident evidence of the aquatic or semiaquatic lifestyle of Lystrosaurus. The lifestyle of Lystrosaurus is still controversial, but we provide more data for this issue.

Other comments in the text
Page 4 Line 9. Comment: This is an important discussion point, but should not be in the materials and methods section. Perhaps move to the discussion when reviewing the variation.

REPLY: We have moved this paragraph to the discussion part.

Page 5 Line 5. Comment: This is a confusing sentence. I think to simplify and to set up the descriptions nicely just describe the histological criteria used to assign a growth line (especially annuli, since you use that term later) vs. LAG.

REPLY: We have modified this sentence as “LAGs are growth marked lines that can be traced around the full circumference. The annuli correspond to periods of slow growth by the presence of parallel-fibered bone and flattened osteons”.

Page 5 Table 1. Comment: I think this table is really confusing. It's hard to compare the sizes between specimens since there are different elements. See overall notes for suggestions.

REPLY: We have added the estimated values as suggested.

Page 5 Table 1. Comment: If you are going to estimate values you have to describe how those estimations were made.

REPLY: We have added an explanation of how to estimate values in the footnotes.

Page 7 line 7. Comment: This is very confusing and I'm not convinced it properly measures cortical thickness. Generally, I don't actually think the "diameter" measurements given in each of the description sections are properly stated either. I would suggest reporting mediolateral and anteroposterior widths (which is basically what I think the "diameter" measurements are?). I believe given the variability in cortical thickness around the bone, reporting the average is not helpful. Instead, maybe report a max and min %?

REPLY: We have modified the MS as suggested.

P10 L4 comment: Label these in the figure.

REPLY: Labeled as suggested.

P10L5 There are no secondary osteons.
Comment: "It looks like there is in fact a massive one in 1C"

REPLY: we assigned it to be an enlarged resorption cavity rather than secondary osteons for its extremely large size. No Haversian system was presented around the erosion cavity.

P10 L6. This has already been described earlier in the paragraph

REPLY: we have deleted it.

P10 L11.
I feel that wide-open is too casual of a term. Instead I would go with something like "enlarged canals". You could also just highlight the band of expanded vascularity with a bracket like: |-----|

REPLY: "enlarged canals" usually indicate secondary remodeling in our manuscript. But here it should be the primary bone. Therefore, we just described its shape here. We have added a bracket as suggested.

P11 Line 16 The humerus is subtriangular in cross-section (midshaft diameter: 21×25 mm) and more robust than the tibia.
Comment: I don't know what this means. In what way is it more robust? Greater cortical thickness?

REPLY: "subtriangular" means triangular but less angled. We have shown more figures in supplementary files (S3 Fig). "robust" means the humerus bone has a larger cross-section. Modified as suggested.

Page 12 Line 9
Where within the cortex are these tissues found? How are they distributed throughout the cortex?

REPLY: We have modified this sentence as "The cortex mainly consists of fibrolamellar bone tissue, but parallel-fibered bone tissue is shown in the posterior cortex".

Page 12 subadult line 3. "The medullary cavity is large (the diameter is about 5 mm)" comment: but not relative to the rest of the cross section...it may be large compared to the juvenile...but this is relative. It appears quite small to me in the figure.

REPLY: We have modified this sentence to "The medullary cavity is relatively large (the diameter is about 5 mm) compared to those of juvenile individuals"

P13. Line 2. What side of the bone is this on? It's a cool structure so some anatomical context would be appreciated.

REPLY: Added as suggested.

P14 Line 1. Also include mid-cortex and endosteal values. See general notes for more on this.

REPLY: We have added these values in the revised MS.

P14 Fig. 4 Where are D and E from in C? See general notes for further comments on this.

REPLY: we have added a box in C to show the position of D and E.

P14. Line 16. The vascular canals are primarily longitudinal but there are also many irregular canals
comments: Where? Label them in figure.

REPLY: We have added the labels in figure 4C.

P14 Line 18. Label these in C as well so there is context for D and E

REPLY: Added.

P15. Comments: The distinction between growth marks and LAGs should be clearly established in the methods section

REPLY: We have added their differences in the methods section. Growth marks and LAGs both form lines, but growth marks could not be traced on the whole circumference cortex bone.

P16 figure 5. comment: Indicate from where in A. See general notes for more details

REPLY: We have added a box in A showing the position of B.

P16 figure 5 wide-open canals.

REPLY: enlarged canals usually referred to secondary remodeling. Here we just describe its shape. It should represent primary bone.

P16 paragraph 3 line 3. Compared to what?

REPLY: We have modified this sentence to make it more clear." The vascular canals are longitudinally-oriented and relatively small in the outer cortex than in the mid and inner cortices".

P16 paragraph 3 line 5 Stay consistent in reporting percentages and report for all regions.

REPLY: We have added more percentages of cortical porosity in different parts of the cortex.

P16 paragraph 3 line 6 Also mention the LARGE one to the bottom of Fig. 6A.

REPLY: The large cavity to the bottom is a large erosional cavity surrounded by endosteal bone rather than secondary osteon because its size is much larger than other secondary osteons (See fig. S8). We have added the description of this large cavity.

P17 line 1.
Is this section truly from the mid-diaphysis? It looks to me like it's slightly towards the proximal or distal end which is fine, but do report it if that's the case. If it is in fact truly from the mid-diaphysis, the medullary cavity is much larger than in the other adult specimen and this difference should be discussed.

REPLY: Thank you for pointing out. The femur of IVPP V26546 that sampled only preserved the distal part. We rechecked this specimen and found that you are correct.

Powered by Editorial Manager® and ProduXion Manager® from Aries Systems Corporation

The measurement of the preserved region is 5 cm, and the estimated whole length is about 14.6 cm. So this section was sampled near the distal region (See figure S1). We have added this information in the method and description part of the paper.

P19 Line 6.
Cite studies such as Köhler et al. 2012 that show LAGs are deposited even in fast growing animals in stressful enivornmental conditions.

REPLY: Cited as suggested.

P19 line13:
There are two discussion points missing from here I think. First, wouldn't the LAGs in the mid-cortex mean that Chinese Lystrosaurus was experiencing some kind of stressful season? Of anything, there is more indicators of stress in specimens from China than those from South Africa. I think if there is an argument to be made about the environment being more stressful in South Africa, you need more clarity on the size differences. If you can show that the South African specimens were smaller than your subadult specimens with LAGs then it would get around this issue. Second, I would move the section where species differences in bone tissue is discussed here and expand on differences that may be related to species-level differences.

REPLY: we have added more comparison on body size between Africa and Chinese Lystrosaurus, and pointed out that our individuals are larger than sampled Africa Lystrosaurus from Early Triassic. We have also added species differences discussion here, although only a little could be clarified here.

Page 19. Paragraph 2. Line5
Do you mean to say something more like, "an artifact of sampling only young individuals from South Africa"? This is not clearly written as is.

REPLY: Here we mean that Lystrosaurus from Early Triassic may die at a young age (indicated by fewer LAGs), so they are smaller than those from late Permian (should be at least subadult based on more LAGs). We have modified this sentence to “but this may the result of the young stage (indicates by rare LAGs) of the body in the Early Triassic based on bone microstructure”.

Page 19. Paragraph 2 line 6.
This formation has not been introduced. Describe it's age here for audience reference.

REPLY: Added as suggested.

Page 20 line 10.
Do you mean in the mid-cortex? This means something else in reference to the gross anatomy so I would avoid using it.

REPLY: We have modified this sentence to “---in the inner and mid cortex”

Page 20. Line 11. This jump is not logically laid out. You state a thick cortex and abundant canals suggests aquatic lifestyle. Then you state the cortical thickness (which I think needs clarification or remeasuring) but do not state whether that is thick or thin compared to other animals. You state that the ribs are different. And then you state that because of this, you can't say it's aquatic/semiaquatic. See general comments for further discussion.

REPLY: we have deleted this sentence and modified this paragraph properly to make it more clear. Most of our samples support Lystrosaurus has a thick cortex, as in Lystrosaurus of Africa. But thick cortex is not only seen in aquatic animals but also shown in extant burrowing animals. Therefore, thick cortex could not provide confident evidence of the aquatic or semiaquatic lifestyle of Lystrosaurus. The lifestyle of Lystrosaurus could not be certain right now, but we provided more methods to solve this problem.

PAGE 28

Powered by Editorial Manager® and ProduXion Manager® from Aries Systems Corporation

	S1 Fig. I did not get this figure in my review document.<br><br>REPLY: Sorry. We have unloaded this figure in the revised version.<br><br>Figure 1. The guides in B to show were zoomed in photos are from are really helpful and necessary. I would suggest adding similar boxes to the rest of the figures for clarity.<br><br>REPLY: We have added boxes in other figures as suggested.<br><br>I would add anatomical directions (i.e. medial, lateral, posterior, anterior) in either the figure or the caption (e.g. medial to right, lateral to left, etc.). I would do this for all of the histology figures.<br><br>REPLY: Thank you for your suggestions. We have added anatomical directions in all the figures.<br><br>Sincerely,<br>Fenglu Han
**Additional Information:**	
**Question**	**Response**
**Financial Disclosure**<br><br>Enter a financial disclosure statement that describes the sources of funding for the work included in this submission. Review the submission guidelines for detailed requirements. View published research articles from *PLOS ONE* for specific examples.<br><br>This statement is required for submission and **will appear in the published article** if the submission is accepted. Please make sure it is accurate.	This study was supported by Strategic Priority Research Program of Chinese Academy of Sciences (XDB26000000) to JL, the National Natural Science Foundation of China (www.nsfc.gov.cn; 41530104, 41661134047) to FH and JL respectively. The funders had no role in study design, data collection and analysis, decision to publish, or preparation of the manuscript.

Powered by Editorial Manager® and ProduXion Manager® from Aries Systems Corporation

**Unfunded studies**<br>Enter: *The author(s) received no specific funding for this work.*<br><br>**Funded studies**<br>Enter a statement with the following details:<br>• Initials of the authors who received each award<br>• Grant numbers awarded to each author<br>• The full name of each funder<br>• URL of each funder website<br>• Did the sponsors or funders play any role in the study design, data collection and analysis, decision to publish, or preparation of the manuscript?<br>• **NO** - Include this sentence at the end of your statement: *The funders had no role in study design, data collection and analysis, decision to publish, or preparation of the manuscript.*<br>• **YES** - Specify the role(s) played.<br><br>* typeset	
**Competing Interests**<br><br>Use the instructions below to enter a competing interest statement for this submission. On behalf of all authors, disclose any competing interests that could be perceived to bias this work—acknowledging all financial support and any other relevant financial or non-financial competing interests.<br><br>This statement **will appear in the published article** if the submission is accepted. Please make sure it is accurate. View published research articles from *PLOS ONE* for specific examples.	The authors have declared that no competing interests exist.

Powered by Editorial Manager® and ProduXion Manager® from Aries Systems Corporation

**NO authors have competing interests**<br><br>Enter: *The authors have declared that no competing interests exist.*<br><br>**Authors with competing interests**<br><br>Enter competing interest details beginning with this statement:<br><br>*I have read the journal's policy and the authors of this manuscript have the following competing interests: [insert competing interests here]*<br><br>* typeset	
**Ethics Statement**<br><br>Enter an ethics statement for this submission. This statement is required if the study involved:<br><br>• Human participants<br>• Human specimens or tissue<br>• Vertebrate animals or cephalopods<br>• Vertebrate embryos or tissues<br>• Field research<br><br>Write "N/A" if the submission does not require an ethics statement.<br><br>General guidance is provided below. Consult the submission guidelines for detailed instructions. **Make sure that all information entered here is included in the Methods section of the manuscript.**	N/A

Powered by Editorial Manager® and ProduXion Manager® from Aries Systems Corporation

**Format for specific study types**<br><br>**Human Subject Research (involving human participants and/or tissue)**<br>• Give the name of the institutional review board or ethics committee that approved the study<br>• Include the approval number and/or a statement indicating approval of this research<br>• Indicate the form of consent obtained (written/oral) or the reason that consent was not obtained (e.g. the data were analyzed anonymously)<br><br>**Animal Research (involving vertebrate animals, embryos or tissues)**<br>• Provide the name of the Institutional Animal Care and Use Committee (IACUC) or other relevant ethics board that reviewed the study protocol, and indicate whether they approved this research or granted a formal waiver of ethical approval<br>• Include an approval number if one was obtained<br>• If the study involved *non-human primates*, add *additional details* about animal welfare and steps taken to ameliorate suffering<br>• If anesthesia, euthanasia, or any kind of animal sacrifice is part of the study, include briefly which substances and/or methods were applied<br><br>**Field Research**<br><br>Include the following details if this study involves the collection of plant, animal, or other materials from a natural setting:<br>• Field permit number<br>• Name of the institution or relevant body that granted permission	
**Data Availability**<br><br>Authors are required to make all data underlying the findings described fully available, without restriction, and from the time of publication. PLOS allows rare exceptions to address legal and ethical concerns. See the PLOS Data Policy and FAQ for detailed information.	Yes - all data are fully available without restriction

A Data Availability Statement describing where the data can be found is required at submission. Your answers to this question constitute the Data Availability Statement and **will be published in the article**, if accepted.<br><br>**Important:** Stating 'data available on request from the author' is not sufficient. If your data are only available upon request, select 'No' for the first question and explain your exceptional situation in the text box.<br><br>Do the authors confirm that all data underlying the findings described in their manuscript are fully available without restriction?	
**Describe where the data may be found in full sentences. If you are copying our sample text, replace any instances of XXX with the appropriate details.**<br><br>• If the data are **held or will be held in a public repository**, include URLs, accession numbers or DOIs. If this information will only be available after acceptance, indicate this by ticking the box below. For example: *All XXX files are available from the XXX database (accession number(s) XXX, XXX.)*.<br>• If the data are all contained **within the manuscript and/or Supporting Information files**, enter the following: *All relevant data are within the manuscript and its Supporting Information files.*<br>• If neither of these applies but you are able to provide **details of access elsewhere**, with or without limitations, please do so. For example:<br><br>*Data cannot be shared publicly because of [XXX]. Data are available from the XXX Institutional Data Access / Ethics Committee (contact via XXX) for researchers who meet the criteria for access to confidential data.*<br><br>*The data underlying the results presented in the study are available from (include the name of the third party*	All relevant data are within the manuscript and its Supporting Information files.

and contact information or URL).<br>• This text is appropriate if the data are owned by a third party and authors do not have permission to share the data.<br><br>* typeset	
Additional data availability information:	

Powered by Editorial Manager® and ProduXion Manager® from Aries Systems Corporation

Manuscript

Click here to access/download;Manuscript;Han et al manucript R1.docx

Bone histology of *Lystrosaurus* (Therapsida: Dicynodontia) from the Lower Triassic of North China, and its implication for lifestyle and environments after the end-Permian extinction

Fenglu Han[1*], Qi Zhao[2], Jun Liu[2,3, 4*]

[1] School of Earth Sciences, China University of Geosciences (Wuhan), Wuhan, China

[2] Key Laboratory of Vertebrate Evolution and Human Origins of Chinese Academy of Sciences, Institute of Vertebrate Paleontology and Paleoanthropology, Chinese Academy of Sciences, Beijing, China

3 CAS Center for Excellence in Life and Paleoenvironment, Beijing 100044, China;

4 College of Earth and Planetary Sciences, University of Chinese Academy of Sciences Beijing 100049

* Corresponding author

Email: hanfl@cug.edu.cn (FH); liujun@ivpp.ac.cn (JL)

Abstract

Lystrosaurus represents one of the most successful dicynodonts, a survivor of the end-Permian mass extinction that remained abundant in the Early Triassic, but many aspects of its paleobiology are still controversial. The bone histology of *Lystrosaurus* species from South Africa and India has provided important information on their growth strategy and lifestyle, but until recently no data was available on the bone histology of *Lystrosaurus* from China. Here, we report on the bone microstructure of seven *Lystrosaurus* individuals from the Lower Triassic of Xinjiang, providing the first such data for the Chinese *Lystrosaurus* species. Our samples indicate that the microstructure of *Lystrosaurus* limb bones from China is characterized by fibrolamellar bone tissue similar to those from South Africa and India. Three ontogenetic stages were identified: juvenile, subadult, and adult based on lines of arrested growth (LAGs) and bone tissue changes. Bone histology supports a rapid growth strategy for *Lystrosaurus* during early ontogeny. Unlike Early Triassic *Lystrosaurus* from South Africa, lines of arrested growth are common in our specimens, suggesting interruption in growth in *Lystrosaurus* from China. The differences in bone histology between *Lystrosaurus* from South Africa and China may indicate different environmental conditions in these two regions.

Introduction

The extinction at the end of the Permian was the most influential event in animal history since the Cambrian explosion. About 80% of all marine species and 50-70% of terrestrial families went extinct [1-3]. Recent studies suggest that the Permian-Triassic transition occurred in an increasingly hot environment which was disruptive to life [4-8], although it is still controversial

whether the environment is arid [6, 7] or humid [9]. However, some organisms evolved new survival strategies, such as miniaturization or rapid propagation [10-12].

Lystrosaurus is a medium to large-sized dicynodont therapsid with a strongly anteroventrally downturned snout. It represents one of the most successful vertebrates that survived the end-Permian mass extinction, showing a cosmopolitan distribution during the Early Triassic [13-17]. However, the survival strategies of *Lystrosaurus* during the extinction are still controversial [18, 19]. The lifestyle of *Lystrosaurus* was supposed to be aquatic or semi-aquatic, which could have helped it to escape the harsh terrestrial environment [20, 21], and was supported by histological studies [22, 23]. There is also evidence that *Lystrosaurus* was a burrow maker [24, 25], and burrowing can help an organism survive harsh environmental conditions, as temperature and humidity are generally more stable underground than on the surface [26, 27]. Also, underground burrows can provide better protection for the young, which helps propagate the species [28].

Interestingly, the body size of *Lystrosaurus* from South Africa is smaller in the Early Triassic than in the late Permian [18], suggesting miniaturization. Furthermore, LAGs (lines of arrested growth) are more common in Permian *Lystrosaurus*, which was interpreted as evidence that the Permian species has a more extended ontogeny that extending over several years [19]. This contrasts with the prevalence of small individuals with few or no LAGs in the Triassic species, which was part of the evidence suggesting breeding young to compensate for dying at an early age [19].

Bone histology can provide important information on the life history of *Lystrosaurus*, which may bear on its survival during the end-Permian mass extinction. Previous studies have focused on the *Lystrosaurus* record from South Africa and India [22, 23, 29, 30]. However,

Lystrosaurus seems to have been cosmopolitan in distribution [14], and is also very abundant in the Juggar and Turpan basins of Xinjiang, China. In China, seven *Lystrosaurus* species have been named since 1935, but the validity of most of these need re-evaluation [31, 32], and it is possible that only two species (*L. hedini* and *L. youngi*) are valid [33]. Here, we present data on the bone histology of *Lystrosaurus* from the Lower Triassic Turpan Basin, Xinjiang Province, China for the first time, using this data to comment on the survival strategy of this taxon.

Material and methods

Seven specimens of *Lystrosaurus* from the collections of the Institute of Vertebrate Paleontology and Paleoanthropology (IVPP) were selected for histological analysis. All materials come from the Lower Triassic Jiucaiyuan Formation of the Taodonggou area (Taoshuyuan), Turpan Basin, Xinjiang, China [34, 35]. The age of the Jiucaiyuan Formation in this area is middle-upper Induan based on previous chronostratigraphy [34, 36]. Identification of these specimens to species level is currently not possible because the Chinese *Lystrosaurus* taxonomy is currently based on skull characters.

Sections were sampled from the mid-diaphysis of limb bones including the tibia, femur, humerus, ulna, and radius (S1 Fig). However, IVPP V26546 was sampled near the distal end (the distance between the sampled position and the distal end is about 5 cm) of the femur due to lack of mid-region. Limb diaphyseal sections were chosen because they are the primary centers of ossification and should preserve the most complete record of growth, and also minimize the potential of secondary remodeling overwriting the growth record (S1 Fig; Table 1) [37, 38]. Rib sections in some individuals were also sampled for comparison. All these materials were

photographed and measured before sectioning (S1 Fig; Table 1). The preparation of the histological sections was carried out at the IVPP. The selected bones were processed using the Exakt-Cutting-Grinding System [39]. Sections were embedded in polyester resin, cut by EXAKT-300CP, and were ground to a thickness of about 50 μm with an EXAKT 400CS grinding machine. The polished sections were then observed under transmitted and polarized light. The thin sections were observed using a Leica DM2700P microscope and photographed using a Leica DMC4500 camera. The whole cross-sections are large and bone microstructure is varied in different areas around the cortex, and we have labeled all the sampled directions for all the figures that are easy for comparison. Nomenclature and definitions of structures follow Francillon-Viellot and Chinsamy-Turan [40, 41]. 'LAGs' are growth marked lines that can be traced around the full circumference. The annuli correspond to periods of slow growth by the presence of parallel-fibered bone and flattened osteons [40].

Table 1 Skeletal elements of the *Lystrosaurus* specimens examined for histological sampling from the Early Triassic of Xinjiang, China.

Specimen number	Elements	Humeral length (cm)	Ulna length	Radial length (cm)	Femoral length (cm)	Tibial length (cm)	Length of fibulae (cm)	Growth stages
IVPP V26543	Tibia	9.9[e]	8.3[e]	7.4[e]	12.5	9.32	9.0	juvenile
IVPP V26544	Humerus, Tibia, Fibula	10.1	7.8[e]	7.0[e]	11.8	9.17	9.0	juvenile
IVPP V26542	Femur	10.3	8.3[e]	6.52	12.5[e]	9.8[e]	9.5[e]	subadult
IVPP V26545	Fibula, Radius	15.0	11.0	10.8[e]	18[e]	14.2[e]	13.8[e]	subadult
IVPP V26546	Femur	12.0	9.7[e]	8.7[e]	14.6[e]	12.5	11.5	adult
IVPP	Femur	12.4 [e]	10[e]	9[e]	15.1	12.1	11.3	adult

V26547								
IVPP V26548	Rib	15.6 [e]	12.6[e]	11.3[e]	19.0	14.0	13.5	adult
IVPP RV35012	None	11.5	9.3	8.3	14.0	10.9	10.6	unknown

[e] denotes estimated values based on the comparison of the ratios between elements of the *Lystrosaurus* IVPP RV35012. The estimated values in each element are all compared to the femur, whereas there is no femur preserved, the estimated values are compared to the humerus. "Growth stages" were based on bone histological information.

The cortical thickness was calculated as the ratio of the cortex to the cross-sectional diameter of the bone [22]. However, they usually vary in different parts of the whole cross-sections, and we have provided a range of cortical thickness in each section (Table 2). The measurement positions of the maximum and minimum of the cortical thickness in all sections were shown in S2 Fig. Cortical porosity was measured using the software ImageJ 1.52a (https://imagej.nih.gov/ij/). It was difficult to obtain the cortical porosity of a whole cross-section for each specimen because of partially breakage or poor preservation in some areas of these sections. Therefore, we subsampled ten areas from the inner to the outermost cortex in all sections (except IVPP V26546 was selected 12 areas) to calculate average vascular porosity. The sampled areas were chosen from the inner region to the periphery on different parts of the whole cross-sections (S3 Fig), so they could reflect the value of cortical porosity. However, some bone sections show unclear bone histology in some regions, which may affect the results. Cortical porosities of those poorly preserved bone sections were labeled to be "estimated values". High-resolution images were acquired using a Leica DMC4500 camera. All the images were converted to 8-bit files, and the brightness/contrast was

adjusted to clarify the structure. After that, we used "threshold" tools in ImageJ to select all the vascular canals. Finally, the cortical porosity was determined using "analyze particles" tools in ImageJ.

Results

The bone histology of *Lystrosaurus* from North China shows a predominance of fibrolamellar bone tissue and a high cortical porosity (Figs 1-6), as in *Lystrosaurus* from South Africa and India [22]. The primary osteons are predominantly longitudinal, forming laminar or plexiform bone. Radially oriented primary osteons are also present in some parts of the cortex. Secondary remodeling is common in the inner and mid-cortical bone in subadult and adult stages, resulting in enlarged channels and thin periosteal bone. The medullary cavity is small and filled with bony trabeculae. LAGs are common in the outer cortex of larger individuals.

All these elements could be divided into juvenile, subadult and adult stages based on bone tissue type and the presence of LAGs (**Table 2**). They are generally similar to the growth stages of *Lystrosaurus* from South Africa and India, but also have some features that differ from the latter.

Table 2. Bone histological information of *Lystrosaurus* from China in each specimen

Specimen number	Skeletal elements	Cortical thickness /porosity (%)	Bone tissue type	Secondary reconstruction	Growth rings	Medullary cavity
IVPP V26543	Tibia	24-32/29	plexiform and reticular	inner enlarged channels	0	a small free cavity
IVPP V26544	Humerus	21-45/32	plexiform	a few secondary osteons	0	a small free cavity

IVPP V26544	Tibia	19-30/27	laminar and reticular	a few secondary osteons	0	full of trabeculae
IVPP V26544	Fibula	31-40/22	laminar	a few secondary osteons	0	a large free cavity
IVPP V26542	Femur	16-41/38[e]	laminar and plexiform	extensive secondary reconstruction	1	a free medullary cavity
IVPP V26545	Fibula	17-35/—	laminar	a few secondary osteons	1	full of trabeculae
IVPP V26545	Radius	22-30/20	laminar	a few secondary osteons	1	full of trabeculae
IVPP V26546	femur	9-24/24	laminar	extensive secondary reconstruction	3	full of trabeculae
IVPP V26547	Femur	18-34/22	laminar	extensive secondary reconstruction	4	full of trabeculae
IVPP V26548	rib	/	laminar	extensive secondary reconstruction	>3	full of trabeculae

[e] denotes estimated values.

Juvenile

The juvenile stage was represented by two samples: IVPP V 26543 and IVPP V 26544. All the cortices are composed of fibrolamellar tissue, with a high number of vascular canals, abundant globular osteocyte lacunae, and no LAGs, suggesting a rapid, constant growth rate. The mid-diaphyseal tibia of IVPP V 26543 is elliptical in cross-section, with a diameter of 13.5 (ML, mediolateral width)×12.0 (AP, anteroposterior width) mm and a cortical thickness ranging from 24-32% (Fig 1; Table 2). The free medullary cavity is small, with a diameter of about 4 mm. The entire

cortex is comprised of woven fibered bone matrix and dense vascular canals (Fig 1). The cortex has a high cortical porosity (about 29%, Table 2). The canals are predominantly longitudinal. A few irregularly shaped canals are shown in the inner and mid cortex (Fig 1B). Radial anastomoses are shown in the medial and anterolateral regions of the cortex, and the medial region has a concave periosteal surface (Fig 1A, C). The anastomoses join two or three adjacent canals in the outer cortex (Fig 1D) and link four to five canals forming long canals in the mid and inner cortex (Fig 1B). Most of the vascular channels show thin centripetal osteonal deposits forming primary osteons (Fig 1E, F). The primary osteons mainly show laminar and plexiform patterns in the outer and deeper cortex, and a row of wide-open canals are shown near the periphery (periosteal surface) in some areas (Fig 1D). Osteocyte density is consistently high throughout the cortex. Erosionally enlarged resorption cavities are present in the inner cortex (Fig 1B, C). There are no secondary osteons, LAGs, or endosteal bone. The medullary region contains cancellous bone.

Fig 1. Bone microstructures in juvenile *Lystrosaurus* IVPP V26543. A, whole cross-section of the tibia; **B**, partial cross-section showing fibrolamellar bone; **C**, partial cross-section showing radial vascular canals; **D**, the outer cortex under normal light; **E**, enlargement of the outer cortex showing primary osteons under normal light; **F**, bone tissue of E under polarized light. **Abbreviation**: **ec**, enlarge resorption cavity; **rc**, radial canals; **wc**, wide-open canals

In IVPP V26544, the tibia, humerus, and fibula were all sectioned (Fig 2). The cross-section of the tibia is elliptical in outline, with a diameter of 12.5 (ML) ×10.0 (AP) mm (Fig 2A). The cortical thickness is ranging from 19% to 30% (Table 2). There is no free medullary cavity. The

cortex mainly consists of fibrolamellar bone tissues (Fig 2B), but parallel-fibered bone tissue is present in the medial region of the cortex articulated with the fibula (Fig 2C). Vascular canals are predominantly longitudinal. There are a few anastomoses that link two to five canals forming long straight and irregular canals. Most of the vascular canals are primary osteons forming a laminar pattern (Fig 2B). A thin layer (1 mm) of secondary osteons is present in the inner region of the compact bone (Fig 2D). The cortex has a high porosity of 27% (Fig 2A, Table 2), but the diameter of vascular canals is slightly reduced near the periphery. Enlarged vascular canals are present in the inner cortex due to secondary remodeling. No LAG/annulus was detected in the cortex. The medullary region is full of trabeculae.

Fig 2. Bone microstructures in juvenile *Lystrosaurus* IVPP V26544. (A-D) tibia. **A**, mid-diaphyseal cross-section of the tibia. **B**, bone microstructure of the tibia; **C**, partial cross-section of the tibia showing the parallel-fibered bone under polarized light; **D**, enlargement inner region of B under crossed plane-polarized light showing secondary osteons; (E-J) humerus. **E**, bone microstructure of the humerus under normal light; **F**. bone microstructure of the humerus under polarized light; **G**, the outermost cortex under normal light showing a growth mark (arrow); **H**, the same region as G under polarized light showing parallel-fibered bone; **I**, the inner cortex showing secondary osteons under normal light; **J**, the same region as H under polarized light; (K-O) fibula. **K**, bone microstructure of the fibula under normal light; **L**, bone microstructure of the fibula under polarized light showing parallel-fibered bone; **M**, enlargement of L showing the organized osteocyte lacunae; **N**, the outermost cortex showing secondary osteons under normal light; **O**, the innermost region showing endosteal bone under polarized light. **Abbreviation**: **end**, endosteal bone; **so**,

secondary osteons.

The humerus is subtriangular in cross-section with a midshaft diameter of 18.5 (AP) ×15.5 (DV, dorsoventral width) mm and has a greater cortical thickness than the tibia (Fig 2E, F and S3 Fig). It has a thick compact bone (at least 7 mm) with a cortical thickness ranging from 21% to 45% (Table 2), and the free medullary cavity is extremely small. The cortex is comprised of fibrolamellar bone tissue, but a thin layer of parallel-fibered bone tissue is present near the periphery (Fig 2F-H). The bone porosity has a value of about 32% (Table 2), which is higher than the tibia. The primary osteons are predominantly plexiform and are longitudinally-oriented in the outer cortex. They are strongly reduced in size in the outer cortex. Parallel-fibered bone tissue and growth marks are present in the outermost region of the cortex (Fig 2G, H), suggesting slower growth rates when the individual died, but the osteocyte lacunae are still abundant. Many secondary osteons are seen in the inner cortex (Fig 2I-J). A thin layer of lamellar endosteal bone is deposited along the edges of the trabeculae in the medullary region (Fig 2F).

The cross-section of the fibula is oval in outline (midshaft diameter 6.5×4 mm, S3 Fig) with a midshaft diameter of 6.3 (AP) × 3.8 (ML) mm and a cortical thickness ranging from 31% to 40% (Table 2). The medullary cavity is small with a diameter of about 2 mm. The cortex mainly consists of fibrolamellar bone tissue, but parallel-fibered bone tissue is shown in the posterior cortex (Fig 2K, L). The cortex is less vascularized than the tibia and humerus, resulting in a lower bone porosity (22%, Table 2). Osteocyte lacunae are abundant but are flattened and organized in parallel-fibered bone (Fig 2M). The primary osteons are predominantly longitudinal and gradually decrease in size towards the periphery. Secondary osteons are mainly in the inner region, but there are also a

few ones near the periphery (Fig 2N). A thin layer of endosteal bone is deposited in the innermost cortex (Fig 2O).

Subadult

The subadult stage was represented by IVPP V26542 and IVPP V26545. The cross-section of the femur of IVPP V26542 is elliptical with a diameter of 15.0 (AP) ×12.5 (DV) mm and a cortical thickness ranging from 16% to 41% (Fig 3). The estimated bone porosity of the whole thin section is about 38%. The medullary cavity is relatively large (the diameter is about 5 mm) compared to those of juvenile individuals. The thickness of the cortex differs around the bone, and the dorsal cortex is much thinner than the ventral cortex (Fig. 3). Furthermore, the thickened ventral cortex has a higher porosity (about 40%) than that of the narrow dorsal cortex (about 17%). The cortex mainly consists of fibrolamellar bone and is highly vascularized. The primary osteons show a laminar pattern that is mainly longitudinally oriented, but radially-oriented channels are found in a narrow channel near the anteroventral cortex (Fig 3A). The number and diameter of the vascular canals are significantly reduced near the periphery. The outer cortex consists of parallel-fibered bone matrix, and a LAG is present near the periphery (Fig 3C, D), suggesting reduced growth rates before it died. The secondary osteons are restricted in the innermost cortex (Fig 3E, F). Endosteal bone is present along the trabeculae in the medullary cavity (Fig 3G).

Fig 3. Bone microstructures of the femur in subadult *Lystrosaurus* IVPP V26542. **A**, mid-diaphyseal transverse section of the femur; **B**, enlargement of A showing highly vascularized cortical porosity; **C**, enlargement of the outer cortex showing the LAG (arrow) under normal light;

D, the same region as C, showing a LAG and parallel-fibered bone under polarized light; **E**, the inner cortex showing secondary osteons under normal light; **F**, the same region as E under polarized light; **G**, the medullary region showing endosteal bone. **Abbreviation**: **end**, endosteal bone; **rc**, radial canals; **so**, secondary osteons.

The cross-sections of the fibula, radius, and rib of IVPP V26545 were sampled for observation. The cross-section of the fibula is subtriangular in outline with a diameter of 13.0 (AP) × 9.8 (ML) mm and a cortical thickness ranging from 17% to 35% (Fig 4A, B; Table 2). The cortex consists of fibrolamellar bone. Most of the vascular canals are longitudinally arranged circumferentially around the bone, forming primary osteons. They are abundant in the inner region with a cortical porosity of about 35% (measured in the lateral cortex), but the number and size decrease from the mid-cortex (cortical porosity about 22%) to the periphery (cortical porosity about 10%). Secondary osteons are restricted to the inner cortex (Fig 4B), which consists of compact coarse cancellous bone (CCCB). A distinct LAG comprised of several growth marks is present near the mid-region of the cortex (Fig 4B). The medullary region is filled with trabeculae (Fig 4A).

Fig 4. Bone microstructure in subadult *Lystrosaurus* IVPP V26545. A, mid-diaphyseal transverse section of the fibula under normal light. The arrow denotes a LAG. **B**, partial outer cortex of the fibula showing the LAG consists of several growth marks (arrows). **C**, partial outer cortex of the radius under normal light. **D**, mid-cortex showing the LAG consists of several growth marks under polarized light (arrows); **E**, the same region as (D) under normal light. **F**, the whole cross-section of rib under normal light; **G,** enlargement of the outer cortex of the rib. The black arrow

indicates LAGs. **Abbreviation**: **irc**, irregular canals; **so**, secondary osteons.

The radius is more robust than the fibula, with a diameter of 17.5 (ML) × 14.9 (AP) mm and a cortical thickness ranging from 22% to 30% (Fig 4C and S3 Fig, Table 2). The cortex mainly consists of woven fibered bone and has a cortical porosity of about 20% (Table 2). The vascular canals are primarily longitudinal but there are also some irregular canals in the mid-cortex (Fig. 4C). Secondary osteons and erosional cavities are present in the inner cortex. A clear LAG, as well as growth marks, are present at the mid-region as in the fibula (Fig 4D, E). Periosteally from the LAG the density of vascular canals is strongly reduced but are still abundant (Fig 4C). The medullary cavity is large with a diameter of about one-third that of the whole cross-section.

The cross-section of the rib is teardrop-shaped, with a diameter of about 11.0 (AP) ×6.5 (ML) mm (Fig 4F, G). The compact bone is very thin (the average thickness is about 0.6 mm) due to a highly remodeled inner and mid-cortex. The medullary cavity is filled with trabeculae. The cortex consists of parallel-fibered bone tissues with only a few vascular canals (Fig 4G). The osteocyte lacunae are still abundant and disorganized. A clear LAG is present, as in the fibula and radius.

Adult

The bone tissue of the femora of IVPP V26546 and IVPP V26547 shows adult tissues. In IVPP V26547, the midshaft of the femoral cortex is elliptical in outline with a diameter of 23.5 (AP) ×19.5 (DV) mm (Fig 5). The cortical thickness is ranging from 18% to 34%, and the cortical porosity is about 22% throughout (Table 2), and the inner region is about 38% but is strongly reduced in the

outer cortex (about 18.5%). There is no free medullary cavity. The cortex is composed of fibrolamellar bone throughout, and the outer cortex consists of parallel-fibered bone matrix with a thickness of about 1 mm (Fig 5E). Primary osteons are predominantly longitudinal and abundant throughout the cortex except for the region near the mid-cortex LAGs and the outer cortex. Secondary osteons are found in the inner and mid-cortex regions. Two prominent LAGs are present in the mid-cortex (Fig 5D, E). The distance between these two LAGs is narrow (about 0.5 mm), and a thin layer of wide-open canals are present around the whole cross-section (Fig 5A, D). In the outermost cortex, the vascular canals are rare and nearly absent (Fig 5F). Two LAGs as well as growth marks are present near the periphery (Fig 5F-H). The osteocyte lacunae are flattened and organized along the growth lines (Fig 5H). The bone histology suggests that this individual grew very slowly before it died, although it has no external fundamental system (EFS), which is present in modern mammals, some dinosaurs, and some dicynodonts [38, 42, 43].

Fig 5. Bone microstructure of the femur in adult *Lystrosaurus* IVPP V26547. A, mid diaphyseal cross-section. **B**, partial cross-section under normal light; **C**, the same cortex as B under crossed plane-polarized light. **D**, enlargement of (B) under normal light, showing LAGs and parallel-fibered bone in the outer cortex. **E**. the same cortex as (C) under polarized light, showing LAGs and parallel-fibered bone. **F**, the outermost cortex under normal light showing LAGs and vascular canals; **G**, the same region as (F) showing parallel-fibered bone; **H**, enlargement of F showing LAGs, osteocyte lacunae and primary osteons. The arrows denote LAGs. **Abbreviation**: **gm,** growth marks; **wc,** wide-open canals

In IVPP V26546, the sampled section near the distal end is elliptical with a diameter of 26.5 (AP) × 23.0 (DV) mm. It has a thin compact bone with a cortical thickness ranging from 9% to 24% and a large medullary region (Fig 6; Table 2). The cortex consists of fibrolamellar bone matrix except for the periphery. The vascular canals are longitudinally-oriented and relatively small in the outer cortex than in the mid and inner cortices (Fig 6B). The average cortical porosity is about 24% (Table 2) and is strongly reduced in the outer cortex (about 10% near the periphery). Secondary osteons are common throughout the cortex and are also shown near the periphery (Fig 6E, F). A large erosional cavity surrounded by endosteal bone was present in the inner region of the ventral cortex (Fig. 6B). A LAG with a narrow avascular layer of annuli is present in the mid-cortex (Fig 6B, C, G, H), and there are only a few vascular canals around. The osteocyte lacunae are flattened and organized within the annuli (Fig 6G, H). At least two LAGs are present near the periphery (Fig 6C, D). The parallel-fibered bone matrix with LAGs and rare vascular canals suggests that it grew slowly before it died.

Fig 6. Bone microstructure in adult *Lystrosaurus* IVPP V26546 and IVPP V26548. (A-H) IVPP V26546. **A**, whole cross-section; **B**, partial transverse section of the femur under normal light; **C**, the outer cortex under polarized light showing parallel-fibered bone and LAGs near the periphery; **D**, enlargement of the outer cortex showing LAGs; **E**, the outermost cortex showing secondary osteons under normal light; **F**, The same region as (E) under polarized light; **G**, enlargement of the annuli region showing the organized osteocyte lacunae; **H**, the same region as G under polarized light; (**I-J**) IVPP V26548. **F**, bone microstructure of the dorsal rib under normal light; **G**, enlargement of the outer region under normal light showing the LAGs. The arrows denote LAGs.

Abbreviation: **anu**, annulus; **ec**, endosteal cavity; **so**, secondary osteons.

In the largest specimen IVPP V26548, only one dorsal rib was sampled (Fig 6I). The cortex is strongly remodeled in the inner and mid-region, and even in the outermost cortex in some region. A thin layer of compact bone is shown in the outermost region. It is nearly avascular, with at least three LAGs near the periphery, which is similar to an EFS. However, these lines did not extend to the outermost region, and there are still several open vascular canals, suggesting this individual was still growing when it died (Fig 6J).

Discussion

The bone tissue of *Lystrosaurus* consists of predominantly fibrolamellar bone with large channels, similar to those from Africa and India [22, 30]. Three growth stages of *Lystrosaurus* described here were identified: a fast-growing juvenile stage, a slower-growing subadult, and an adult that grew very slowly. The fast growth rates may have helped them to adapt to the environment quickly [30]. The late juvenile was characterized by uninterrupted fibrolamellar bone tissue and the subadult was primarily fibrolamellar bone tissue interrupted by LAGs. The adult is nearly fully grown, which is characterized by the presence of parallel-fibered bone and closely spaced LAGs in the outermost cortex. Bone tissue type in *Lystrosaurus* is not only related to the growth stages but also differs between the various limb bones, including the femur, tibia, fibula and radius. The humerus, femur and tibia show a mainly laminar to plexiform vascular arrangement with a high cortical porosity in all stages, whereas the vascular canals of the fibula and radius are mostly longitudinally oriented and reduced in density, resulting in a low cortical porosity. In addition, our

samples indicate that the humerus and tibia have higher cortical porosity than that of other limb bones, but the parallel-fibered bone with growth marks near the periphery appeared earlier in the humerus than in other elements (IVPP V26544).

The adult stage was usually represented by the presence of an EFS [38], which has been identified in some dicynodonts, including *Placerias* [43] and *Kannemeyeria* [44]. There is still no typical EFS detected in *Lystrosaurus*. Ray et al. (2005) found parallel-fibered bone in the outer cortex of a dorsal rib and a thin layer of lamellar bone in a vertebra at the adult stage, but they did not find a typical EFS with closely spaced LAGs near the periphery. In our samples, the parallel-fibered bone in the outer cortex appeared in the dorsal rib and other elements, representing the subadult stage here (Fig 4). Although there is no typical EFS detected, the presence of more LAGs and parallel-fibered bone tissue near the periphery suggests that it grew very slowly before it died.

Botha et al. (2016) found that all Early Triassic *Lystrosaurus* from South Africa have no more than one observed LAG and no parallel-fibered bone in the outermost cortex, which suggests that they did not reach skeletal maturity [19]. However, LAGs seem to be more commonly distributed in *Lystrosaurus* from the Early Triassic of China. Within the seven individuals, three have more than one LAG and two have one LAG for each. The presence of at least one LAG in *Lystrosaurus* from China has a humerus ranging from 10.3 to 15.6 cm, and a femur ranging from 12 to 19 cm, which is much larger than the sampled one LAG *Lystrosaurus* from the Early Triassic of Africa (i.e. SAM-PK-K8, SAM-PK-8991 have a humeral length of about 10 cm) [19, 22, 30]. This suggests that our sampled specimens are larger than the sampled individuals of *Lystrosaurus* from the Early Triassic of Africa.

Bone histology in the four *Lystrosaurus* species from the Lower Triassic of South Africa

is structurally similar [30, 45], which also occurs in our samples. However, IVPP V26545 shows a large humerus (15 cm) but with only one LAG that is supposed to be subadult, but all other individuals with the humerus larger than 12 cm have more than two LAGs to be among adult stage (Table 1). In addition, the ratio of humerus length to ulna length is about 1.4, which is larger than that of IVPP RV3501(1.2). This may indicate a different species of *Lystrosaurus*, which needs to clarify in the future.

The presence of LAGs and the large size of *Lystrosaurus* from China suggest that many *Lystrosaurus* reached at least the subadult stage, unlike Triassic *Lystrosaurus* from Africa where subadults are rare [19]. This difference may be caused by differing environmental conditions in these areas[23, 46]. Although broadly connected on the Pangaean supercontinent, the Karoo Basin and North China were geographically distant in the Early Triassic, being located in mid-to-high latitudes of the southern and northern hemispheres, respectively [47]. Although evidence from calcic vertisols indicates some region of Karoo Basin to a wetting trend towards the *Daptocephalus*-*Lystrosaurus* Assemblage-Zone boundary [9], the hot and arid environment of Karoo Basin in the Early Triassic had been widely accepted [6, 7, 45]. These harsh environmental conditions may have resulted in high mortality rates of *Lystrosaurus*, with most individuals dying during the early stage [19]. By comparison, the climate is inferred to have been semiarid-subhumid in the Early Triassic of North China [34, 48, 49], which may indicate better environmental conditions than that in South Africa.

Another important issue is whether the body size of *Lystrosaurus* was reduced in the Early Triassic due to the harsh environment such as arid weather. Body size reduction has been shown in non-mammalian eutheriodont therapsids during the end-Permian mass extinction [11], and a

reduction is also observed in *Lystrosaurus* from the Karoo Basin of South Africa, but this may the result of the young stage of the body (indicates by rare LAGs) in the Early Triassic based on bone microstructure [19]. Interestingly, the body size of *Lystrosaurus* from the lower section of the Guodikeng Formation (late Permian), such as *L. youngi* is smaller than the upper section of Jiucaiyuan Formation, contrasting with the condition in South Africa [32]. This may indicate that the known materials of *L. youngi* represent immature individuals, whereas later Chinese *Lystrosaurus* from Early Triassic are at least subadult. However, the bone microstructure of *Lystrosaurus* from the Guodikeng Formation is poorly preserved, and more material needs to be assessed in the future to confirm this hypothesis.

The lifestyle of *Lystrosaurus* has been argued to be aquatic/semiaquatic [22], fully terrestrial, or burrowing [20, 21]. In recent decades, this controversy has concentrated on aquatic or borrowing habits, which could benefit its survival in the hot environments of the Early Triassic. The aquatic/semiaquatic lifestyle was supported by the bone microstructure showing a thick cortex and porous vascular canals [22], and linear discriminant analysis based on the compactness profile and body size parameters in extant animals [50, 51]. The cortical thicknesses of most samples described here are between 20% - 40%, which is similar to *Lystrosaurus* from South Africa [22]. And the main difference is the sampled dorsal rib has dense erosional cavities in the inner and mid cortex and a thin layer of compact outer cortex (Figs. 4, 6), which differs from the thick cortex (27-37%) in *Lystrosaurus* from Africa [22]. In sum, our results also show that *Lystrosaurus* has a thick cortex as in extent aquatic animals, such as *Hippopotamus* [52]. However, the thick cortex is not only shown in aquatic animals but also reported in extant burrowing animals and nonmammalian therapsids [41]. Therefore, thick cortex could not provide confident evidence the aquatic or semiaquatic lifestyle of

Lystrosaurus. Moreover, Juvenile *Lystrosaurus* has been found inside burrow casts indicating that they were active burrowers [24, 25].

Whether *Lystrosaurus* had a substantial aquatic component to its lifestyle in addition to burrowing could be elucidated by analyzing stable isotopes in the future. Previous studies suggest that aquatic and semi-aquatic vertebrates have $\delta^{18}O$ values significantly lower than those of coexisting terrestrial animals [53-55]. Oxygen and calcium isotopes all proved spinosaurids to be aquatic or semiaquatic like modern crocodiles [56, 57], and they could also provide more evidence for the lifestyle of *Lystrosaurus*. Furthermore, $\delta^{18}O$ isotopes have been already used in some late Permian dicynodontians, and the result shows that *Endothiodon* may have a semi-aquatic lifestyle, whereas *Tropidostoma* was a terrestrial species [58].

Conclusion

Here we have studied the bone histology of *Lystrosaurus* from the Early Triassic of China based on thin sections of seven individuals. The results indicate that *Lystrosaurus* grew rapidly in the early stages as previously shown in *Lystrosaurus* from South Africa and India. However, the presence of LAGs in the largest individual suggests that the growth of *Lystrosaurus* was interrupted in the late stage. LAGs are rare in *Lystrosaurus* from South Africa but they are more abundant in the *Lystrosaurus* from Xinjiang, China. We propose that most *Lystrosaurus* from Xinjiang reached subadult and adult stages, whereas those from South Africa died during their juvenile stage. This distinction may be caused by different environmental conditions, but more samples from the Early Triassic of China are needed to test this hypothesis in the future.

Acknowledgment

We thank Zhang Shukang and Wu Rui for helping to make bone sections, Chu Daoliang for his helpful discussion. We thank Jennifer Botha, Megan Whitney, and Zoe Kulik for their comments on a previous edition of this paper. Megan Whitney also reviewed this version and have provided more constructive and useful comments. We thank the editor Jörg Fröbisch, and the reviewer Christian F. Kammerer, and two other anonymous reviewers for their very helpful comments on this manuscript.

References

1. Maxwell WD. Permian and Early Triassic extinction of non-marine tetrapods. Palaeontology. 1992;35(3):571-83.

2. Erwin DH. The Permo-Triassic extinction. Nature. 1994;367(6460):231-5.

3. Stanley SM. Estimates of the magnitudes of major marine mass extinctions in earth history. Proc Natl Acad Sci. 2016;113(42):E6325-E34. doi: 10.1073/pnas.1613094113

4. Retallack GJ. Permian and Triassic greenhouse crises. Gondwana Res. 2013;24(1):90-103. doi: org/10.1016/j.gr.2012.03.003.

5. Sun Y, Joachimski MM, Wignall PB, Yan C, Chen Y, Jiang H, et al. Lethally hot temperatures during the Early Triassic greenhouse. Science. 2012;338(6105):366-70. doi: 10.1126/science.1224126.

6. Smith RMH, Botha-Brink J. Anatomy of a mass extinction: Sedimentological and taphonomic evidence for drought-induced die-offs at the Permo-Triassic boundary in the main Karoo Basin, South Africa. Palaeogeogr Palaeoclimatol Palaeoecol. 2014;396(3):99–118.

7. MacLeod KG, Quinton PC, Bassett DJ. Warming and increased aridity during the earliest Triassic in the Karoo Basin, South Africa. Geology. 2017;45(6):483-6. doi: 10.1130/G38957.1

8. Bernardi M, Petti FM, Benton MJ. Tetrapod distribution and temperature rise during the Permian-Triassic mass extinction. Proc R Soc Lond B Biol Sci. 2018;285(1870):20172331. doi: doi:10.1098/rspb.2017.2331.

9. Gastaldo RA, Kus K, Tabor N, Neveling J. Calcic Vertisols in the upper Daptocephalus Assemblage Zone, Balfour Formation, Karoo Basin, South Africa: Implications for Late Permian Climate. J Sediment Res. 2020;90(6):609-28. doi: 10.2110/jsr.2020.32

10. Song H, Tong J. Mass extinction and survival during the Permian-Triassic crisis. Earth Sci. 2016;41(6):901-18. (in chinese with English abstract)

11. Huttenlocker AK. Body size reductions in nonmammalian eutheriodont therapsids (Synapsida) during the end-Permian mass extinction. PLoS One. 2014;9(2):e87553. doi: 10.1371/journal.pone.0087553.

12. Chu D, Tong J, Song H, Benton MJ, Song H, Yu J, et al. Lilliput effect in freshwater ostracods during the Permian–Triassic extinction. Palaeogeogr Palaeoclimatol Palaeoecol. 2015;435:38-52. doi: https://doi.org/10.1016/j.palaeo.2015.06.003.

13. Smith RM, Ward PD. Pattern of vertebrate extinctions across an event bed at the Permian-Triassic boundary in the Karoo Basin of South Africa. Geology. 2001;29(12):1147-50. doi: org/10.1130/0091-7613(2001)029<1147:POVEAA>2.0.CO;2.

14. Smith R, Botha J. The recovery of terrestrial vertebrate diversity in the South African Karoo Basin after the end-Permian extinction. C R Palevol. 2005;4(6-7):623-36. doi: org/10.1016/j.crpv.2005.07.005.

15. Colbert EH. The distribution of *Lystrosaurus* in Pangaea and its implications. Geobios. 1982;15:375-83. doi: org/10.1016/S0016-6995(82)80126-5.

16. Cosgriff JW, Hammer WR, Ryan WJ. The Pangaean Reptile, *Lystrosaurus maccaigi*, in the Lower Triassic of Antarctica. J Paleontol. 1982;56(2):371-85.

17. Fröbisch J. Composition and similarity of global anomodont-bearing tetrapod faunas. Earth-Sci Rev. 2009;95(3):119-57. doi: https://doi.org/10.1016/j.earscirev.2009.04.001.

18. Botha J, Smith RMH. *Lystrosaurus* species composition across the Permo-Triassic boundary in the Karoo Basin of South Africa: *Lystrosaurus* across the PTB. Lethaia. 2007;40(2):125-37. doi: org/10.1111/j.1502-3931.2007.00011.x.

19. Botha-Brink J, Codron D, Huttenlocker AK, Angielczyk KD, Ruta M. Breeding young as a survival strategy during earth's greatest mass extinction. Sci Rep. 2016;6(1):24053. doi: org/10.1038/srep24053.

20. King GM, Cluver MA. The aquatic *Lystrosaurus*: An alternative lifestyle. Hist Biol. 1991;4(3-4):323-41. doi: org/10.1080/08912969009386548.

21. King GM. The aquatic *Lystrosaurus*: a palaeontological myth. Hist Biol. 1991;4(3-4):285-321.

22. Ray S, Chinsamy A, Bandyopadhyay S. *Lystrosaurus murrayi* (therapsida, dicynodontia): bone histology, growth and lifestyle adaptations. Palaeontology. 2005;48(6):1169–85. doi: org/10.1111/j.1475-4983.2005.00513.x.

23. Ray S, Mukherjee D, Bandyopadhyay S. Growth patterns of fossil vertebrates as deduced from bone microstructure: case studies from India. J Biosci. 2009;34(5):661-72. doi: org/10.1007/s12038-009-0055-x.

24. Botha-Brink J. Burrowing in *Lystrosaurus*: preadaptation to a postextinction environment? J

Vert Paleontol. 2017;37(5):e1365080. doi: 10.1080/02724634.2017.1365080.

25. Modesto SP, Botha-Brink J. A burrow cast with *Lystrosaurus skeletal* remains from the Lower Triassic of South Africa. Palaios. 2010;25(4):274-81. doi: org/10.2110/palo.2009.p09-077r.

26. Tałanda M, DzięCioł S, Sulej T, NiedźWiedzki G. Vertebrate burrow system from the Upper Triassic of Poland. Palaios. 2011;26(2):99-105. doi: org/10.2110/palo.2010.p10-024r.

27. Hasiotis ST, Wellner RW, Martin AJ, Demko TM. Vertebrate Burrows from Triassic and Jurassic Continental Deposits of North America and Antarctica: Their Paleoenvironmental and Paleoecological Significance. Ichnos. 2004;11(1-2):103-24. doi: org/10.1080/10420940490428760.

28. Özkurt Ö, Yiğit N, Çolak E, Sözen M, Gharkheloo M. Observations on the Ecology, Reproduction and Behavior of *Spermophilus* Bennett, 1835 (Mammalia: Rodentia) in Turkey. Turk J Zool. 2005;29:91-9.

29. Chinsamy A, Rubidge BS. Dicynodont (Therapsida) bone histology: phylogenetic and physiological implications. Palaeontol Afr. 1993;30:97-102.

30. Botha-Brink J, Angielczyk KD. Do extraordinarily high growth rates in Permo-Triassic dicynodonts (Therapsida, Anomodontia) explain their success before and after the end-Permian extinction? Zool J Linn Soc. 2010;160(2):341-65. doi: org/10.1111/j.1096-3642.2009.00601.x.

31. Li J-L. *Lystrosaurus* of China. Vertebr Palasiat. 1988;26(4):241-9. (in Chinese with English abstract)

32. Liu J, Li J, Cheng Z. The *Lystrosaurus* fossils from Xinjiang and their bearing on the terrestrial Permian Triassic boundary. Vertebr Palasiat. 2002;40(4):267-75. (in Chinese with English abstract)

33. Camp J, and Liu J. 2011. The taxonomy and cranial morphology of Chinese *Lystrosaurus*. [Abstract] Supplement to online Journal of Vertebrate Paleontology, 71st Annual Meeting Society

of Vertebrate Paleontology: 82

34. Yang W, Feng Q, Liu Y, Tabor N, Miggins D, Crowley JL, et al. Depositional environments and cyclo- and chronostratigraphy of uppermost Carboniferous–Lower Triassic fluvial–lacustrine deposits, southern Bogda Mountains, NW China — A terrestrial paleoclimatic record of mid-latitude NE Pangea. Global Planet Change. 2010;73(1):15-113. doi: org/10.1016/j.gloplacha.2010.03.008.

35. Thomas SG, Tabor NJ, Yang W, Myers TS, Yang Y, Wang D. Palaeosol stratigraphy across the Permian–Triassic boundary, Bogda Mountains, NW China: Implications for palaeoenvironmental transition through earth's largest mass extinction. Palaeogeogr Palaeoclimatol Palaeoecol. 2011;308(1):41-64. doi: org/10.1016/j.palaeo.2010.10.037.

36. Wan M, Yang W, Wang JJ. A new *Protophyllocladoxylon* wood from the Induan (Lower Triassic) Jiucaiyuan Formation in the Turpan–Hami Basin, southern Bogda Mountains, Rev Palaeobot Palynol. northwestern China. 2019;267:62-72.

37. Horner JR, de Ricqlès A, Padian K. Variation in dinosaur skeletochronology indicators: implications for age assessment and physiology. Paleobiology. 1999;25(3): 295-304. doi: org/10.1017/S0094837300021308.

38. Chinsamy-Turan A. The microstructure of dinosaur bone. Baltimore: Johns Hopkins University Press; 2005. 195 p.

39. Donath K, Rohrer M. Bone Sectioning Using the Exakt System. In: An YH, Martin KL, editors. Handbook of Histology Methods for Bone and Cartilage: Springer; 2003. pp. 243-52.

40. Francillon-Viellot H, de Buffrénil V, Castanet J, Géraudie J, Meunier FJ, Dire JY, et al. Microstructure and mineralization of vertebrate skeletal tissues. In: Carter JG, editor. Skeletal

biomineralization: patterns, processes and evolutionary trends. New York: Van Nostrand Reinhold; 1990. p. 471-530.

41. Chinsamy-Turan A. The microstructure of bones and teeth of nonmammalian therapsids. In: Chinsamy-Turan A, editor. Forerunners of mammals: radiation, histology, biology. Bloomington, Indiana: Indiana University Press; 2012. p. 65-90.

42. Horner JR, De Ricqles A, Padian K. Long bone histology of the hadrosaurid dinosaur *Maiasaura peeblesorum*: Growth dynamics and physiology based on an ontogenetic series of skeletal elements. J Vert Paleontol. 2000;20(1):115-29. doi: org/10.1671/0272-4634(2000)020[0115:LBHOTH]2.0.CO;2.

43. Green JL, Schweitzer MH, Lamm ET. Limb bone histology and growth in *Placerias hesternus* (Therapsida: Anomodontia) from the Upper Triassic of North America. Palaeontology. 2010;53(2): 347-64. doi: org/10.1111/j.1475-4983.2010.00944.x.

44. Ray S, Botha-Brink J, Chinsamy-Turan AJ. Dicynodont growth dynamics and lifestyle adaptations. In: Chinsamy-Turan A, editor. The forerunners of mammals: radiation, histology and biology. Bloomington: Indiana University Press; 2012. p. 121-48.

45. Botha J, Huttenlocker AK, Smith RMH, Prevec R, Viglietti P, Modesto SP. New geochemical and palaeontological data from the Permian-Triassic boundary in the South African Karoo Basin test the synchronicity of terrestrial and marine extinctions. Palaeogeogr Palaeoclimatol Palaeoecol. 2020;540:109467. doi: org/10.1016/j.palaeo.2019.109467.

46. Köhler M, Marín-Moratalla N, Jordana X, Aanes R. Seasonal bone growth and physiology in endotherms shed light on dinosaur physiology. Nature. 2012;487(7407):358-61. doi: 10.1038/nature11264.

47. Muttoni G, Gaetani M, Kent DV, Sciunnach D, Angiolini L, Berra F, et al. Opening of the Neo-Tethys Ocean and the Pangea B to Pangea A transformation during the Permian. GeoArabia. 2009;14(4):17-48.

48. Liu D, Zhang C, Yang D, Pan Z, Kong X, Huang Z, et al. Petrography and geochemistry of the Lopingian (Upper Permian)-Lower Triassic strata in the southern Junggar and Turpan basins, NW China: implications for weathering, provenance, and palaeogeography. Int Geol Rev. 2019;61(8):1016-36. doi: org/10.1080/00206814.2018.1490932.

49. Tabor NJ, Sidor CA, Smith RMH, Nesbitt SJ, Angielczyk KD. Paleosols of the Permian-Triassic: proxies for rainfall, climate change and major changes in terrestrial tetrapod diversity. J Vert Paleontol. 2017;37(sup1):240-53. doi: org/10.1080/02724634.2017.1415211.

50. Germain D, Laurin M. Microanatomy of the radius and lifestyle in amniotes (Vertebrata, Tetrapoda). Zool Scr. 2005;34(4):335-50. doi: org/10.1111/j.1463-6409.2005.00198.x.

51. Canoville A, Laurin M. Evolution of humeral microanatomy and lifestyle in amniotes, and some comments on palaeobiological inferences. Biol J Linn Soc. 2010;100(2):384-406. doi: org/10.1111/j.1095-8312.2010.01431.x.

52. Wall WP. The Correlation between High Limb-Bone Density and Aquatic Habits in Recent Mammals. J Paleontol. 1983;57(2):197-207.

53. Bocherens H, Koch PL, Mariotti A, Geraads D, Jaeger J-J. Isotopic biogeochemistry (^{13}C, ^{18}O) of mammalian enamel from African Pleistocene hominid sites. Palaios. 1996;11(4):306-18. doi: org/10.2307/3515241

54. Fricke HC, Rogers RR. Multiple taxon–multiple locality approach to providing oxygen isotope evidence for warm-blooded theropod dinosaurs. Geology. 2000;28(9):799-802. doi:

org/10.1130/0091-7613(2000)28<799:MTLATP>2.0.CO;2

55. Clementz MT, Holroyd PA, Koch PL. Identifying aquatic habits of herbivorous mammals through stable isotope analysis. Palaios. 2008;23(9):574-85. doi: org/10.2110/palo.2007.p07-054r

56. Hassler A, Martin J, Amiot R, Tacail T, Godet FA, Allain R, et al. Calcium isotopes offer clues on resource partitioning among Cretaceous predatory dinosaurs. Proc R Soc Lond B Biol Sci. 2018;285(1876):20180197. doi: org/10.1098/rspb.2018.0197.

57. Amiot R, Buffetaut E, Lécuyer C, Wang X, Boudad L, Ding Z, et al. Oxygen isotope evidence for semi-aquatic habits among spinosaurid theropods. Geology. 2010;38(2):139-42. doi: org/10.1130/G30402.1.

58. Rey K, Day MO, Amiot R, Fourel F, Luyt J, Lécuyer C, et al. Stable isotopes (δ18O and δ13C) give new perspective on the ecology and diet of Endothiodon bathystoma (Therapsida, Dicynodontia) from the late Permian of the South African Karoo Basin. Palaeogeogr Palaeoclimatol Palaeoecol. 2020;556:109882. doi: org/10.1016/j.palaeo.2020.109882.

Supporting information

S1 Fig. The accurate sampled positions of all specimens in this study. **A**, IVPP V26543, arrow denotes the sampled position of the tibia; (**B, C)** IVPP V26544. **B**, the sampled positions of the tibia and fibula; C, the sampled position of the humerus; **D**, IVPP V26542, the distal end of the femur that sampled; (**E-G)** IVPP V26545. **E**, the sampled position of the radius; F, the sampled position of the fibula; **G**, the sampled position of the rib; **H**, IVPP V26547, arrow denotes the sampled position of the femur; **I**, IVPP V26546, arrow denotes the sampled position of the femur; **J**, IVPP V26548, arrow denotes the sampled position of the rib.

S2 Fig. Measurements of the cortical thickness in all thin sections. A, IVPP V26543, tibia; **B**, IVPP V26544 tibia; **C**, IVPP V26544, humerus; **D**, IVPP V26544, fibula; **E**, IVPP V26542, femur; **F**, IVPP V26545, fibula; **G**, IVPP V26545, radius; **H**, IVPP V26546, femur; **I**, IVPP V26547, femur. The yellow lines denote measurements of the cortical thickness. The red lines denote measurements of cross-sectional diameters, and the green circles denote outlines of the medullary cavities.

S3 Fig. Bone microstructure and the sampled areas for calculating cortical porosity in all thin sections.

Figure 1 R1

Click here to access/download;Figure;Figure 1 R1.tif

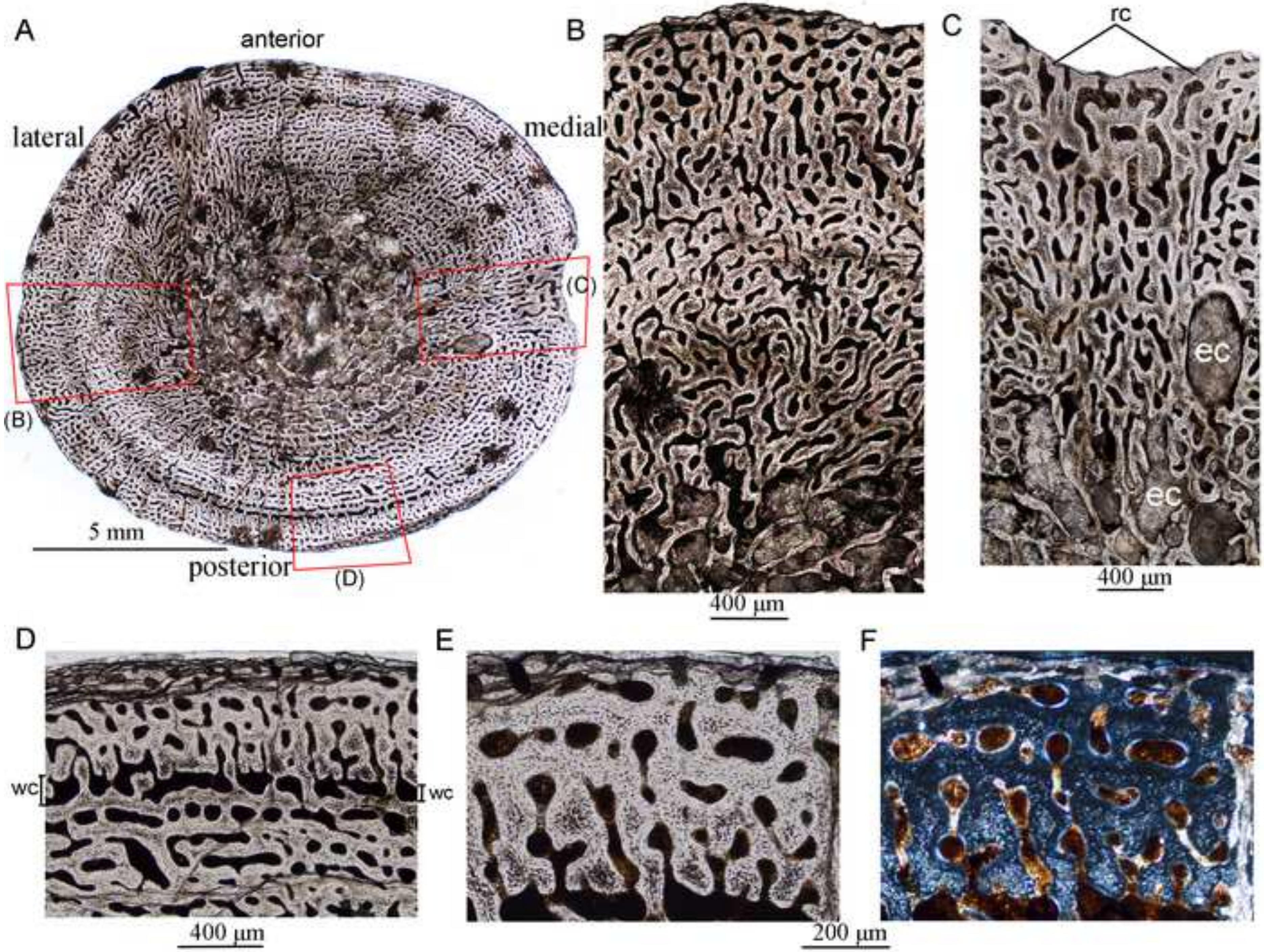

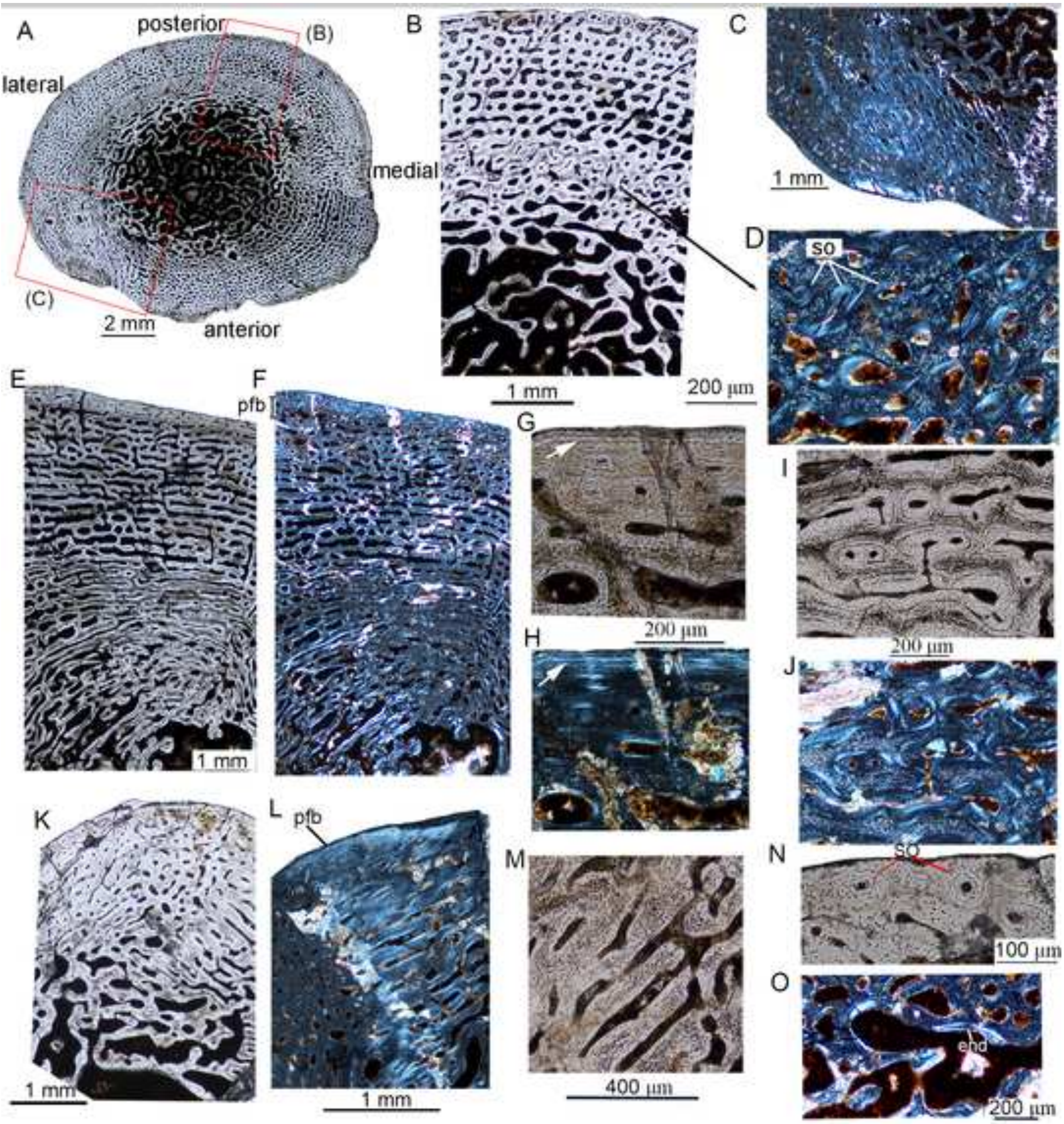
A
posterior
(B)
lateral
medial
(C)
2 mm
anterior
B
1 mm
200 µm
C
1 mm
D
so
E
1 mm
F
pfb
G
200 µm
H
I
200 µm
J
K
1 mm
L
pfb
1 mm
M
400 µm
N
so
100 µm
O
end
200 µm

 Click here to access/download;Figure;Figure 3 R1.tif

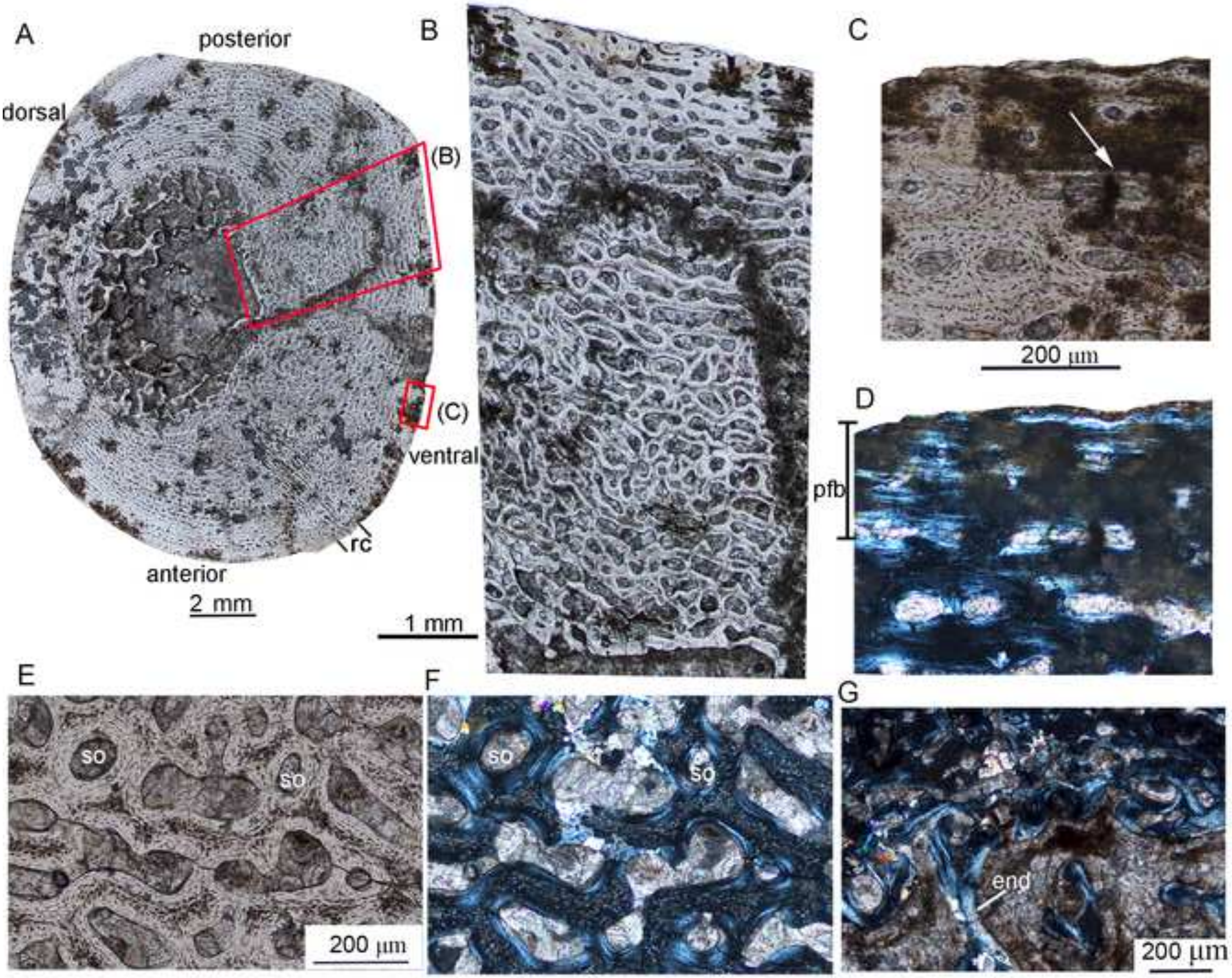

Figure 4 R1 Click here to access/download;Figure;Figure 4 R1.tif

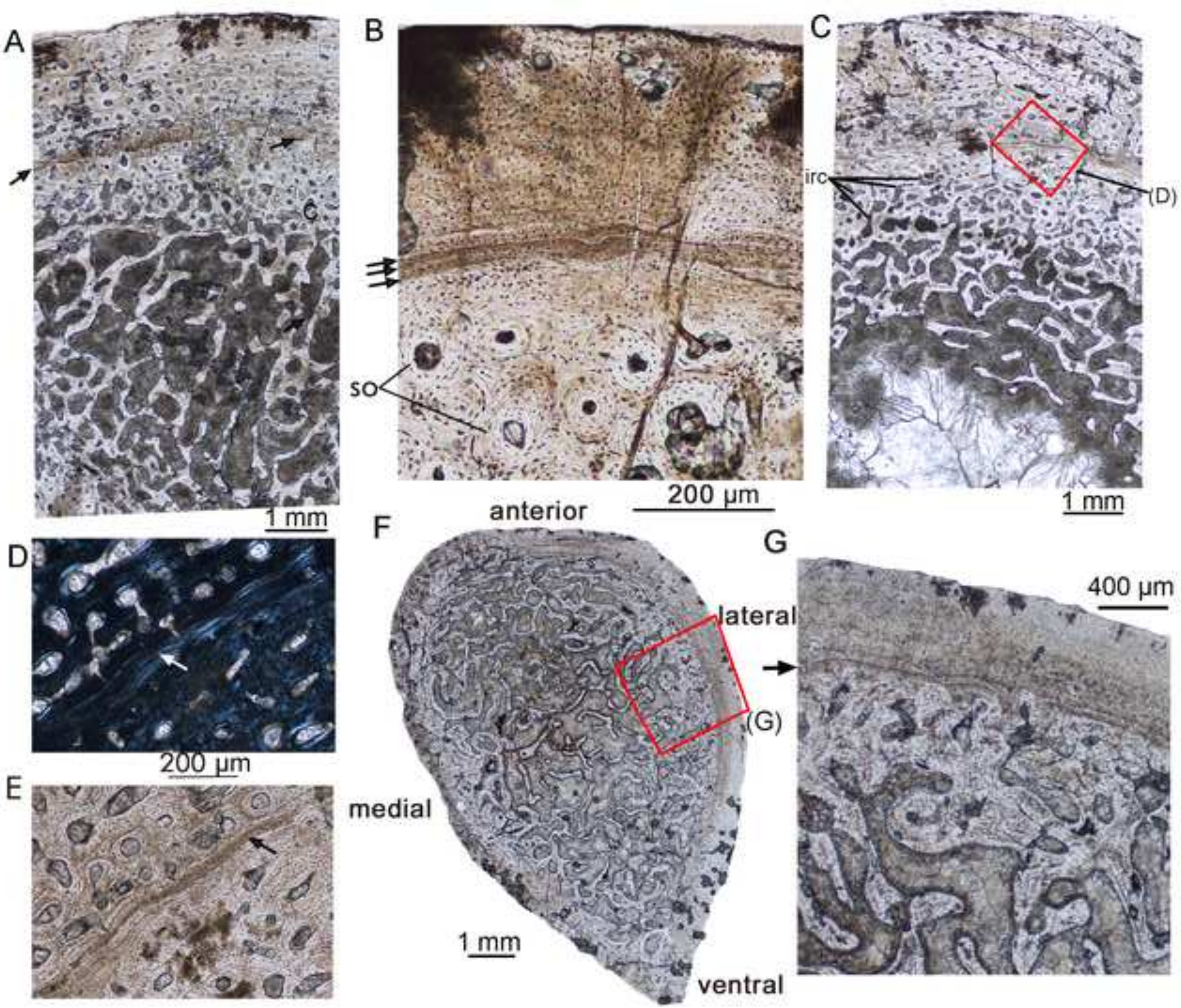

Click here to access/download;Figure;Figure 5 R1.tif

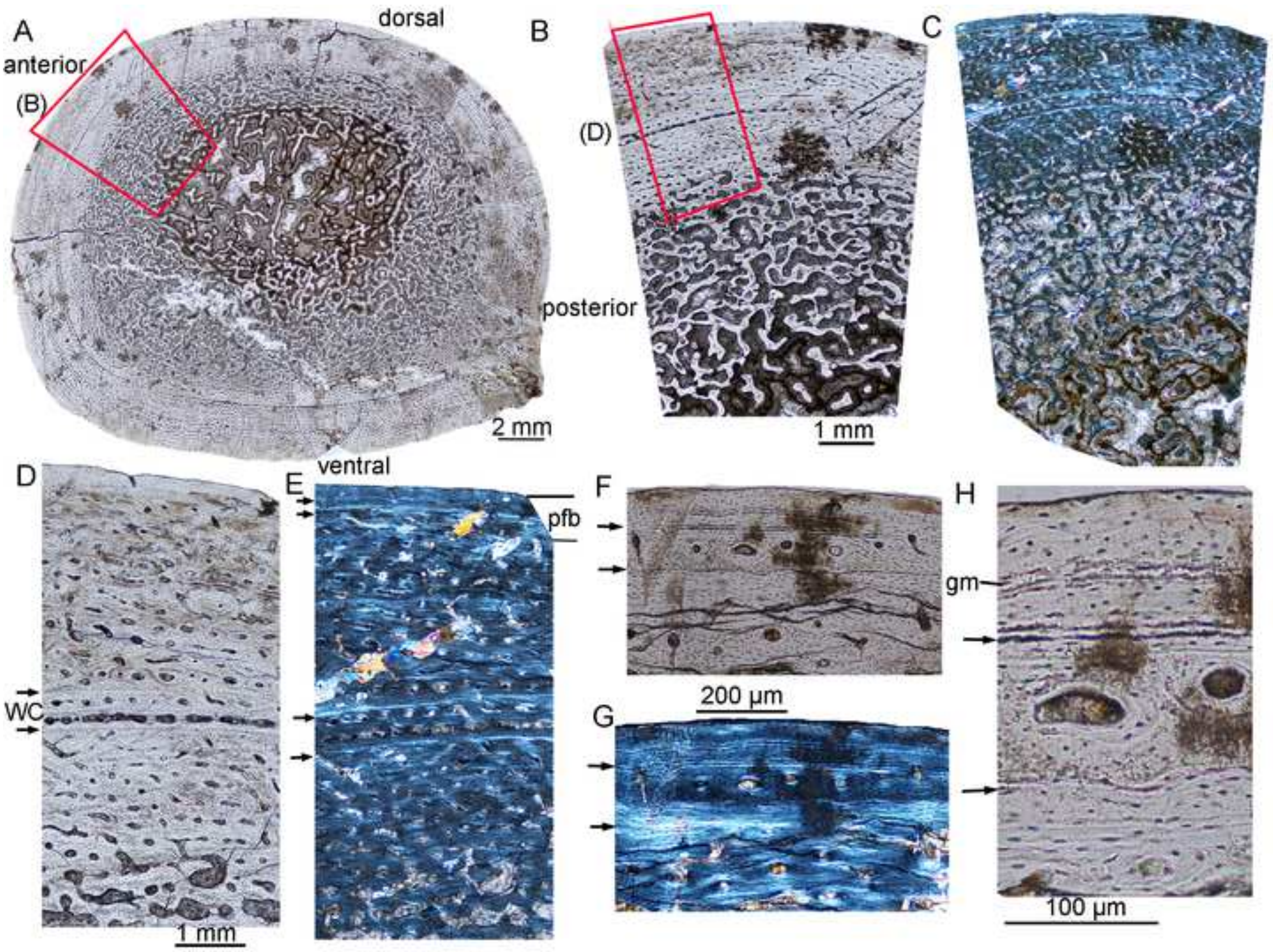

Figure 6 R1 Click here to access/download;Figure;Figure 6 R1.tif

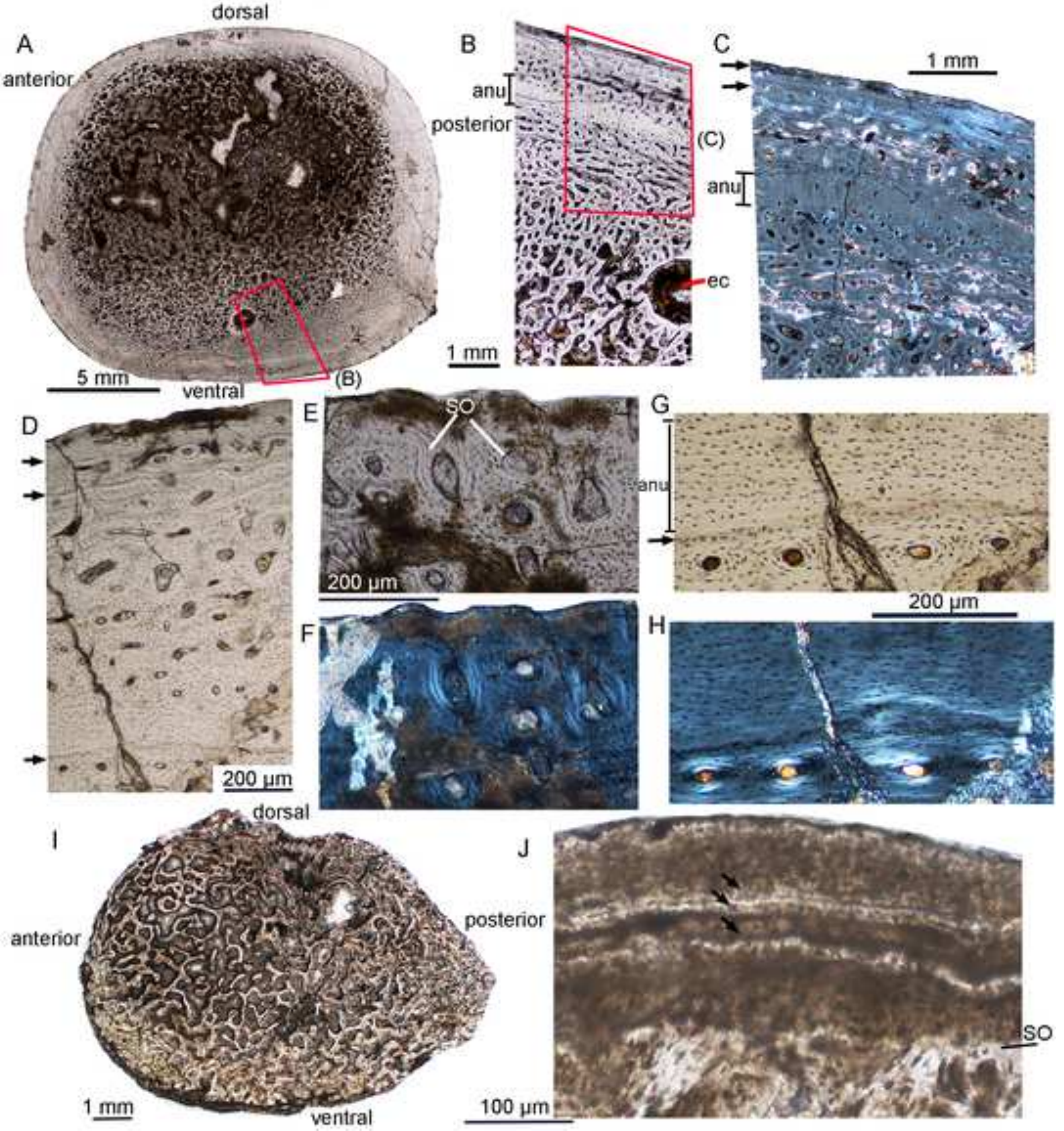

Click here to access/download
Supporting Information
S1 Fig.pdf

Click here to access/download
Supporting Information
S2 Fig.pdf

Click here to access/download
Supporting Information
S3 Fig.pdf

Bone histology of *Lystrosaurus* (Therapsida: Dicynodontia) from the Lower Triassic of North China, and its implication for lifestyle and environments after the end-Permian extinction

Fenglu Han[1*], Qi Zhao[2], Jun Liu[2,3, 4*]

[1] School of Earth Sciences, China University of Geosciences (Wuhan), Wuhan, China

[2] Key Laboratory of Vertebrate Evolution and Human Origins of Chinese Academy of Sciences, Institute of Vertebrate Paleontology and Paleoanthropology, Chinese Academy of Sciences, Beijing, China

3 CAS Center for Excellence in Life and Paleoenvironment, Beijing 100044, China;

4 College of Earth and Planetary Sciences, University of Chinese Academy of Sciences Beijing 100049

* Corresponding author

Email: hanfl@cug.edu.cn (FH); liujun@ivpp.ac.cn (JL)

Abstract

Lystrosaurus represents one of the most successful dicynodonts, a survivor of the end-Permian mass extinction that remained abundant in the Early Triassic, but many aspects of its paleobiology are still controversial. The bone histology of *Lystrosaurus* species from South Africa and India has provided important information on their growth strategy and lifestyle, but until recently no data was available on the bone histology of *Lystrosaurus* from China. Here, we report on the bone microstructure of seven *Lystrosaurus* individuals from the Lower Triassic of Xinjiang, providing the first such data for the Chinese *Lystrosaurus* species. Our samples indicate that the microstructure of *Lystrosaurus* limb bones from China is characterized by fibrolamellar bone tissue similar to those from South Africa and India. Three ontogenetic stages were identified: juvenile, subadult, and adult based on lines of arrested growth (LAGs) and bone tissue changes. Bone histology supports a rapid growth strategy for *Lystrosaurus* during early ontogeny. Unlike Early Triassic *Lystrosaurus* from South Africa, lines of arrested growth are common in our specimens, suggesting interruption in growth in *Lystrosaurus* from China. The differences in bone histology between *Lystrosaurus* from South Africa and China may indicate different environmental conditions in these two regions.

Introduction

The extinction at the end of the Permian was the most influential event in animal history since the Cambrian explosion. About 80% of all marine species and 50-70% of terrestrial families went extinct [1-3]. Recent studies suggest that the Permian-Triassic transition occurred in an increasingly hot environment which was disruptive to life [4-8], although it is still controversial

whether the environment ~~is~~ arid [6, 7] or humid [9]. However, some organisms evolved new survival strategies, such as miniaturization or rapid propagation [10-12].

Lystrosaurus is a medium to large-sized dicynodont therapsid with a strongly anteroventrally downturned snout. It represents one of the most successful vertebrates that survived the end-Permian mass extinction, showing a cosmopolitan distribution during the Early Triassic [13-17]. However, the survival strategies of *Lystrosaurus* during the extinction are still controversial [18, 19]. The lifestyle of *Lystrosaurus* was ~~supposed to be~~ aquatic or semi-aquatic, which could have helped it to escape the harsh terrestrial environment [20, 21], and was supported by histological studies [22, 23]. There is also evidence that *Lystrosaurus* was a burrow maker [24, 25], and burrowing can help an organism survive harsh environmental conditions, as temperature and humidity are generally more stable underground than on the surface [26, 27]. Also, underground burrows can provide better protection for the young, which helps propagate the species [28].

Interestingly, the body size of *Lystrosaurus* from South Africa is smaller in the Early Triassic than in the late Permian [18], suggesting miniaturization. Furthermore, LAGs (lines of arrested growth) are more common in Permian *Lystrosaurus*, which was interpreted as evidence that the Permian species ~~has~~ a more extended ontogeny ~~that~~ extending over several years [19]. This contrasts with the prevalence of small individuals with few or no LAGs in the Triassic species, which was part of the evidence suggesting breeding ~~young~~ to compensate for dying at an early age [19].

Bone histology can provide important information on the life history of *Lystrosaurus*, which may bear on its survival during the end-Permian mass extinction. Previous studies have focused on the *Lystrosaurus* record from South Africa and India [22, 23, 29, 30]. However,

Lystrosaurus seems to have been cosmopolitan in distribution [14], and is also very abundant in the Juggar and Turpan basins of Xinjiang, China. In China, seven *Lystrosaurus* species have been named since 1935, but the validity of most of these need re-evaluation [31, 32], and it is possible that only two species (*L. hedini* and *L. youngi*) are valid [33]. Here, we present data on the bone histology of *Lystrosaurus* from the Lower Triassic Turpan Basin, Xinjiang Province, China for the first time, using this data to comment on the survival strategy of this taxon.

Material and methods

Seven specimens of *Lystrosaurus* from the collections of the Institute of Vertebrate Paleontology and Paleoanthropology (IVPP) were selected for histological analysis. All materials come from the Lower Triassic Jiucaiyuan Formation of the Taodonggou area (Taoshuyuan), Turpan Basin, Xinjiang, China [34, 35]. The age of the Jiucaiyuan Formation in this area is middle-upper Induan based on previous chronostratigraphy [34, 36]. Identification of these specimens to species level is currently not possible because the Chinese *Lystrosaurus* taxonomy is currently based on skull characters.

Sections were sampled from the mid-diaphysis of limb bones including the tibia, femur, humerus, ulna, and radius (S1 Fig). However, IVPP V26546 was sampled near the distal end (the distance between the sampled position and the distal end is about 5 cm) of the femur due to lack of mid-region. Limb diaphyseal sections were chosen because they are the primary centers of ossification and should preserve the most complete record of growth, and also minimize the potential of secondary remodeling overwriting the growth record (S1 Fig; Table 1) [37, 38]. Rib sections in some individuals were also sampled for comparison. All these materials were

photographed and measured before sectioning (S1 Fig; Table 1). The preparation of the histological sections was carried out at the IVPP. The selected bones were processed using the Exakt-Cutting-Grinding System [39]. Sections were embedded in polyester resin, cut by EXAKT-300CP, and were ground to a thickness of about 50 μm with an EXAKT 400CS grinding machine. The polished sections were then observed under transmitted and polarized light. The thin sections were observed using a Leica DM2700P microscope and photographed using a Leica DMC4500 camera. The whole cross-sections are large and bone microstructure ~~is varied~~ in different areas around the cortex, and we have labeled all the sampled directions for all the figures ~~that are easy for~~ comparison. Nomenclature and definitions of structures follow Francillon-Viellot and Chinsamy-Turan [40, 41]. 'LAGs' are growth mark~~ed~~ lines that can be traced around the full circumference The annuli correspond to periods of slow growth by the presence of parallel-fibered bone and flattened osteons [40].

Table 1 Skeletal elements of the *Lystrosaurus* specimens examined for histological sampling from the Early Triassic of Xinjiang, China.

Specimen number	Element	Humeral length (cm)	Ulna length	Radial length (cm)	Femoral length (cm)	Tibial length (cm)	Length of fibulae (cm)	Growth stages
IVPP V26543	Tibia	9.9[e]	8.3[e]	7.4[e]	12.5	9.32	9.0	juvenile
IVPP V26544	Humerus, Tibia, Fibula	10.1	7.8[e]	7.0[e]	11.8	9.17	9.0	juvenile
IVPP V26542	Femur	10.3	8.3[e]	6.52	12.5[e]	9.8[e]	9.5[e]	subadult
IVPP V26545	Fibula, Radius	15.0	11.0	10.8[e]	18[e]	14.2[e]	13.8[e]	subadult
IVPP V26546	Femur	12.0	9.7[e]	8.7[e]	14.6[e]	12.5	11.5	adult
IVPP	Femur	12.4 [e]	10[e]	9[e]	15.1	12.1	11.3	adult

V26547								
IVPP V26548	Rib	15.6 [e]	12.6[e]	11.3[e]	19.0	14.0	13.5	adult
IVPP RV35012	None	11.5	9.3	8.3	14.0	10.9	10.6	unknown

[e] denotes estimated values based on the comparison of the ratios between elements of the *Lystrosaurus* IVPP RV350[illegible] The estimated values in each element are all compared to the femur, whereas there is no femur preserved, the estimated values are compared to the humerus. "Growth stages" were based on bone histological information.

The cortical thickness was calculated as the ratio of the cortex to the cross-section[illegible] diameter of the bone [22]. However, they usually vary in different parts of the whole cross-section~~s~~, and we have provided a range of cortical thickness in each section (Table 2). The measurement positions of the maximum and minimum of the cortical thickness in all sections ~~were~~ shown in S2 Fig. Cortical porosity was measured using the software ImageJ 1.52a (https://imagej.nih.gov/ij/). It was difficult to obtain the cortical porosity of a whole cross-section for each specimen because of partiall~~y~~ breakage or poor preservation in some areas of these sections. Therefore, we subsampled ten areas from the inner to the outermost cortex in all sections (except IVPP V26546 ~~was selected~~ 12 areas) to calculate average vascular porosity. The sampled areas were chosen from the inner region to the periphery on different parts of the whole cross-sections (S3 Fig), so they could reflect the value of cortical poro[illegible] However, some bone sections show unclear bone histology in some regions, which may affect the results. Cortical porosities of those poorly preserved bone sections were labeled ~~to be~~ "estimated values". High-resolution images were acquired using a Leica DMC4500 camera. All the images were converted to 8-bit files, and the brightness/contrast was

adjusted to clarify the structure. After that, we used “threshold” tools in ImageJ to select all the vascular canals. Finally, the cortical porosity was determined using “analyze particles” tools in ImageJ.

Results

The bone histology of *Lystrosaurus* from North China shows a predominance of fibrolamellar bone tissue and a high cortical porosity (Figs 1-6), as in *Lystrosaurus* from South Africa and India [22] The primary osteons are predominantly longitudinal, forming laminar or plexiform bone. Radially oriented primary osteons are also present in some parts of the cortex. Secondary remodeling is common in the inner and mid-cortical bone in subadult and adult stages, resulting in enlarged channels and thin periosteal bone. The medullary cavity is small and filled with bony trabeculae. LAGs are common in the outer cortex of larger individuals.

All these elements could be divided into juvenile, subadult and adult stages based on bone tissue type and the presence of LAGs (**Table 2**). They are generally similar to the growth stages of *Lystrosaurus* from South Africa and India, but also have some features that differ from the latter.

Table 2. Bone histological information of *Lystrosaurus* from China in each specimen

Specimen number	Skeletal elements	Cortical thickness /porosity (%)	Bone tissue type	Secondary reconstruction	Growth rings	Medullary cavity
IVPP V26543	Tibia	24-37/29	plexiform and reticular	inner enlarged channels	0	a small free cavity
IVPP V26544	Humerus	21-45/32	plexiform	a few secondary osteons	0	a small free cavity

IVPP V26544	Tibia	19-30/27	laminar and reticular	a few secondary osteons	0	full of trabeculae
IVPP V26544	Fibula	31-40/22	laminar	a few secondary osteons	0	a large free cavity
IVPP V26542	Femur	16-41/38[e]	laminar and plexiform	extensive secondary reconstruction	1	a free medullary cavity
IVPP V26545	Fibula	17-35/—	laminar	a few secondary osteons	1	full of trabeculae
IVPP V26545	Radius	22-30/20	laminar	a few secondary osteons	1	full of trabeculae
IVPP V26546	femur	9-24/24	laminar	extensive secondary reconstruction	3	full of trabeculae
IVPP V26547	Femur	18-34/22	laminar	extensive secondary reconstruction	4	full of trabeculae
IVPP V26548	rib	/	laminar	extensive secondary reconstruction	>3	full of trabeculae

[e] denotes estimated values.

Juvenile

The juvenile stage was represented by two samples: IVPP V 26543 and IVPP V 26544. All the cortices are composed of fibrolamellar tissue, with a high number of vascular canals, abundant globular osteocyte lacunae, and no LAGs, suggesting a rapid, constant growth rate. The mid-diaphyseal tibia of IVPP V 26543 is elliptical in cross-section, with a diameter of 13.5 (ML, mediolateral width)×12.0 (AP, anteroposterior width) mm and a cortical thickness ranging from 24-32% (Fig 1; Table 2). The free medullary cavity is small, with a diameter of about 4 mm. The entire

cortex is comprised of woven fibered bone matrix and dense vascular canals (Fig 1). The cortex has a high cortical porosity (about 29%, Table 2). The canals are predominantly longitudinal. A few irregularly shaped canals are shown in the inner and mid cortex (Fig 1B). Radial anastomoses are shown in the medial and anterolateral regions of the cortex, and the medial region has a concave periosteal surface (Fig 1A, C). The anastomoses join two or three adjacent canals in the outer cortex (Fig 1D) and link four to five canals forming long canals in the mid and inner cortex (Fig 1B). Most of the vascular channels show thin centripetal osteonal deposits forming primary osteons (Fig 1E, F). The primary osteons mainly show laminar and plexiform patterns in the outer and deeper cortex, and a row of wide-open canals are shown near the periphery (periosteal surface) in some areas (Fig 1D). Osteocyte density is consistently high throughout the cortex. Erosionally enlarged resorption cavities are present in the inner cortex (Fig 1B, C). There are no secondary osteons, LAGs, or endosteal bone. The medullary region contains cancellous bone.

Fig 1. Bone microstructures in juvenile *Lystrosaurus* IVPP V26543. A, whole cross-section of the tibia; **B**, partial cross-section showing fibrolamellar bone; **C**, partial cross-section showing radial vascular canals; **D**, the outer cortex under normal light; **E**, enlargement of the outer cortex showing primary osteons under normal light; **F**, bone tissue of E under polarized light. **Abbreviation**: **ec**, enlarge resorption cavity; **rc**, radial canals; **wc**, wide-open canals

In IVPP V26544, the tibia, humerus, and fibula were all sectioned (Fig 2). The cross-section of the tibia is elliptical in outline, with a diameter of 12.5 (ML) ×10.0 (AP) mm (Fig 2A). The cortical thickness is ranging from 19% to 30% (Table 2). There is no free medullary cavity. The

cortex mainly consists of fibrolamellar bone tissues (Fig 2B), but parallel-fibered bone tissue is present in the medial region of the cortex articulated with the fibula (Fig 2C). Vascular canals are predominantly longitudinal. There are a few anastomoses that link two to five canals forming long straight and irregular canals. Most of the vascular canals are primary osteons forming a laminar pattern (Fig 2B). A thin layer (1 mm) of secondary osteons is present in the inner region of the compact bone (Fig 2D). The cortex has a high porosity of [illegible] (Fig 2A, Table 2), but the diameter of vascular canals is slightly reduced near the periphery. Enlarged vascular canals are present in the inner cortex due to secondary remodeling. No LAG/annulus was detected in the cortex. [illegible] medullary region is full of trabeculae.

Fig 2. Bone microstructures in juvenile *Lystrosaurus* IVPP V26544. (A-D) tibia. **A**, mid-diaphyseal cross-section of the tibia. **B**, bone microstructure of the tibia; **C**, partial cross-section of the tibia showing the parallel-fibered bone under polarized light; **D**, enlargement inner region of B under crossed plane-polarized light showing secondary osteons; (E-J) humerus. **E**, bone microstructure of the humerus under normal light; **F**, bone microstructure of the humerus under polarized light; **G**, the outermost cortex under normal light showing a growth mark (arrow); **H**, the same region as G under polarized light showing parallel-fibered bone; **I**, the inner cortex showing secondary osteons under normal light; **J**, the same region as H under polarized light; (K-O) fibula. **K**, bone microstructure of the fibula under normal light; **L**, bone microstructure of the fibula under polarized light showing parallel-fibered bone; **M**, enlargement of L showing the organized osteocyte lacunae; **N**, the outermost cortex showing secondary osteons under normal li[illegible] **O**, the innermost region showing endosteal bone under polarized light. **Abbreviation**: **end**, endosteal bone; **so**,

secondary osteons.

The humerus is subtriangular in cross-section with a midshaft diameter of 18.5 (AP) ×15.5 (DV, dorsoventral width) mm and has a greater cortical thickness than the tibia (Fig 2E, F and S3 Fig). It has a thick compact bone (at least 7 mm) with a cortical thickness ranging from 21% to 45% (Table 2), and the free medullary cavity is extremely small. The cortex is comprised of fibrolamellar bone tissue, but a thin layer of parallel-fibered bone tissue is present near the periphery (Fig 2F-H). The bone porosity has a value of about 32% (Table 2), which is higher than the tibia. The primary osteons are predominantly plexiform and are longitudinally-oriented in the outer cortex. They are strongly reduced in size in the outer cortex. Parallel-fibered bone tissue and growth marks are present in the outermost region of the cortex (Fig 2G, H), suggesting slower growth rates when the individual died, but the osteocyte lacunae are still abundant. Many secondary osteons are seen in the inner cortex (Fig 2I-J). A thin layer of lamellar endosteal bone is deposited along the edges of the trabeculae in the medullary region (Fig 2F).

The cross-section of the fibula is oval in outline (midshaft diameter 6.5×4 mm, S3 Fig) with a midshaft diameter of 6.3 (AP) × 3.8 (ML) mm and a cortical thickness ranging from 31% to 40% (Table 2). The medullary cavity is small with a diameter of about 2 mm. The cortex mainly consists of fibrolamellar bone tissue, but parallel-fibered bone tissue is shown in the posterior cortex (Fig 2K, L). The cortex is less vascularized than the tibia and humerus, resulting in a lower bone porosity (22%, Table 2). Osteocyte lacunae are abundant but are flattened and organized in parallel-fibered bone (Fig 2M). The primary osteons are predominantly longitudinal and gradually decrease in size towards the periphery. Secondary osteons are mainly in the inner region, but there are also a

few ~~ones~~ near the periphery (Fig 2N). A thin layer of endosteal bone is deposited in the innermost cortex (Fig 2O).

Subadult

The subadult stage was represented by IVPP V26542 and IVPP V26545. The cross-section of the femur of IVPP V26542 is elliptical with a diameter of 15.0 (AP) ×12.5 (DV) mm and a cortical thickness ranging from 16% to 41% (Fig 3). The estimated bone porosity of the whole thin section is about 38%. The medullary cavity is relatively large (the diameter is about 5 mm) compared to those of juvenile individuals. The thickness of the cortex differs around the bone, and the dorsal cortex is much thinner than the ventral cortex (Fig. 3). Furthermore, the thickened ventral cortex has a higher porosity (about 40%) than that of the narrow dorsal cortex (about 17%). The cortex mainly consists of fibrolamellar bone and is highly vascularized. The primary osteons show a laminar pattern that is mainly longitudinally oriented, but radially-oriented channels are found in a narrow channel near the anteroventral cortex (Fig 3A). The number and diameter of the vascular canals are significantly reduced near the periphery. The outer cortex consists of parallel-fibered bone matrix, and a LAG is present near the periphery (Fig 3C, D), suggesting reduced growth rates before it died. The secondary osteons are restricted ~~in~~ the innermost cortex (Fig 3E, F). Endosteal bone is present along the trabeculae in the medullary cavity (Fig 3G).

Fig 3. Bone microstructures of the femur in subadult *Lystrosaurus* IVPP V26542. A, mid-diaphyseal transverse section of the femur; **B**, enlargement of A showing highly vascularized cortical porosity; **C**, enlargement of the outer cortex showing the LAG (arrow) under normal light;

D, the same region as C, showing a LAG and parallel-fibered bone under polarized light; **E**, the inner cortex showing secondary osteons under normal light; **F**, the same region as E under polarized light; **G**, the medullary region showing endosteal bone. **Abbreviation**: **end**, endosteal bone; **rc**, radial canals; **so**, secondary osteons.

The cross-sections of the fibula, radius, and rib of IVPP V26545 were sampled for observation. The cross-section of the fibula is subtriangular in outline with a diameter of 13.0 (AP) × 9.8 (ML) mm and a cortical thickness ranging from 17% to 35% (Fig 4A, B; Table 2). The cortex consists of fibrolamellar bone. Most of the vascular canals are longitudinally arranged circumferentially around the bone, forming primary osteons. They are abundant in the inner region with a cortical porosity of about 35% (measured in the lateral cortex), but the number and size decrease from the mid-cortex (cortical porosity about 22%) to the periphery (cortical porosity about 10%). Secondary osteons are restricted to the inner cortex (Fig 4B), which consists of compact coarse cancellous bone (CCCB). A distinct LAG comprised of several growth marks is present near the mid-region of the cortex (Fig 4B). The medullary region is filled with trabeculae (Fig 4A).

Fig 4. Bone microstructure in subadult *Lystrosaurus* IVPP V26545. A, mid-diaphyseal transverse section of the fibula under normal light. The arrow denotes a LAG. **B**, partial outer cortex of the fibula showing the LAG consists of several growth marks (arrows). **C**, partial outer cortex of the radius under normal light. **D**, mid-cortex showing the LAG consists of several growth marks under polarized light (arrows); **E**, the same region as (D) under normal light. **F**, the whole cross-section of rib under normal light; **G,** enlargement of the outer cortex of the rib. The black arrow

indicates LAGs. **Abbreviation**: **irc**, irregular canals; **so**, secondary osteons.

The radius is more robust than the fibula, with a diameter of 17.5 (ML) × 14.9 (AP) mm and a cortical thickness ranging from 22% to 30% (Fig 4C and S3 Fig, Table 2). The cortex mainly consists of woven fibered bone and has a cortical porosity of about 20% (Table 2). The vascular canals are primarily longitudinal but there are also some irregular canals in the mid-cortex (Fig. 4C). Secondary osteons and erosional cavities are present in the inner cortex. A clear LAG, as well as growth marks are present at the mid-region as in the fibula (Fig 4D, E). Periosteally from the LAG the density of vascular canals is strongly reduced but are still abundant (Fig 4C). The medullary cavity is large with a diameter of about one-third that of the whole cross-section.

The cross-section of the rib is teardrop-shaped, with a diameter of about 11.0 (AP) ×6.5 (ML) mm (Fig 4F, G). The compact bone is very thin (the average thickness is about 0.6 mm) due to a highly remodeled inner and mid-cortex. The medullary cavity is filled with trabeculae. The cortex consists of parallel-fibered bone tissues with only a few vascular canals (Fig 4G). The osteocyte lacunae are still abundant and disorganized. A clear LAG is present, as in the fibula and radius.

Adult

The bone tissue of the femora of IVPP V26546 and IVPP V26547 shows adult tissues. In IVPP V26547, the midshaft of the femoral cortex is elliptical in outline with a diameter of 23.5 (AP) ×19.5 (DV) mm (Fig 5). The cortical thickness ~~is ranging~~ from 18% to 34%, and the cortical porosity is about 22% throughout (Table 2), and the inner region is about 38% but is strongly reduced in the

outer cortex (about 18.5%). There is no free medullary cavity. The cortex is composed of fibrolamellar bone throughout, and the outer cortex consists of parallel-fibered bone matrix with a thickness of about 1 mm (Fig 5E). Primary osteons are predominantly longitudinal and abundant throughout the cortex except for the region near the mid-cortex LAGs and the outer cortex. Secondary osteons are found in the inner and mid-cortex regions. Two prominent LAGs are present in the mid-cortex (Fig 5D, E). The distance between these two LAGs is narrow (about 0.5 mm), and a thin layer of ~~wide-open~~ canals are present around the whole cross-section (Fig 5A, D). In the outermost cortex, the vascular canals are rare and nearly absent (Fig 5F). Two LAGs as well as growth marks are present near the periphery (Fig 5F-H). The osteocyte lacunae are flattened and organized along the growth lines (Fig 5H). The bone histology suggests that this individual grew very slowly before it died, although it has no external fundamental system (EFS), which is present in modern mammals, some dinosaurs, and some dicynodonts [18, 42, 43].

Fig 5. Bone microstructure of the femur in adult ***Lystrosaurus*** **IVPP V26547. A**, mid diaphyseal cross-section. **B**, partial cross-section under normal light; **C**, the same cortex as B under crossed plane-polarized light. **D**, enlargement of (B) under normal light, showing LAGs and parallel-fibered bone in the outer cortex. **E**. the same cortex as (C) under polarized light, showing LAGs and parallel-fibered bone. **F**, the outermost cortex under normal light showing LAGs and vascular canals; **G**, the same region as (F) showing parallel-fibered bone; **H**, enlargement of F showing LAGs, osteocyte lacunae and primary osteons. The arrows denote LAGs. **Abbreviation**: **gm,** growth marks; **wc,** wide-open canal.

In IVPP V26546, the sampled section near the distal end is elliptical with a diameter of 26.5 (AP) × 23.0 (DV) mm. It has a thin compact bone with a cortical thickness ranging from 9% to 24% and a large medullary region (Fig 6; Table 2). The cortex consists of fibrolamellar bone ~~matrix~~ except for the periphery. The vascular canals are longitudinally-oriented and relatively small in the outer cortex ~~than in~~ the mid and inner cortices (Fig 6B). The average cortical porosity is about 24% (Table 2) and is strongly reduced in the outer cortex (about 10% near the periphery). Secondary osteons are common throughout the cortex and are also shown near the periphery (Fig 6E, F). A large erosional cavity surrounded by endosteal bone was present in the inner region of the ventral cortex (Fig. 6B). A LAG with a narrow avascular layer of annuli is present in the mid-cortex (Fig 6B, C, G, H), and there are only a few vascular canals around. The osteocyte lacunae are flattened and organized within the annuli (Fig 6G, H). At least two LAGs are present near the periphery (Fig 6C, D). The parallel-fibered bone matrix with LAGs and rare vascular canals suggests that it grew slowly before it died.

Fig 6. Bone microstructure in adult *Lystrosaurus* IVPP V26546 and IVPP V26548. (A-H) IVPP V26546. **A**, whole cross-section; **B**, partial transverse section of the femur under normal light; **C**, the outer cortex under polarized light showing parallel-fibered bone and LAGs near the periphery; **D**, enlargement of the outer cortex showing LAGs; **E**, the outermost cortex showing secondary osteons under normal light; **F**, The same region as (E) under polarized light; **G**, enlargement of the ~~annuli region~~ showing the organized osteocyte lacunae; **H**, the same region as G under polarized light; **(I-J)** IVPP V26548. **F**, bone microstructure of the dorsal rib under normal light; **G**, enlargement of the outer region under normal light showing the LAGs. The arrows denote LAGs.

Abbreviation: **anu**, annulus; **ec**, endosteal cavity; **so**, secondary osteons.

In the largest specimen IVPP V26548, only one dorsal rib was sampled (Fig 6I). The cortex is strongly remodeled in the inner and mid-region, and even in the outermost cortex in some region. A thin layer of compact bone is shown in the outermost region. It is nearly avascular, with at least three LAGs near the periphery, which is similar to an EFS. However, these lines did not extend to the outermost region, and there are still several open vascular canals, suggesting this individual was still growing when it died (Fig 6J).

Discussion

The bone tissue of *Lystrosaurus* consists ~~of~~ predominantly fibrolamellar bone with large channels, similar to those from Africa and India [22, 30]. Three growth stages of *Lystrosaurus* described here were identified: a fast-growing juvenile stage, a slower-growing subadult, and an adult that grew very slowly. The fast growth rates may have helped them to adapt to the environment quickly [30]. The late juvenile was characterized by uninterrupted fibrolamellar bone tissue and the subadult was primarily fibrolamellar bone tissue interrupted by LAGs. The adult is nearly fully grown, which is characterized by the presence of parallel-fibered bone and closely spaced LAGs in the outermost cortex. Bone tissue type in *Lystrosaurus* is not only related to the growth stages but also differs between the various limb bones, including the femur, tibia, fibula and radius. The humerus, femur and tibia show a mainly laminar to plexiform vascular arrangement with a high cortical porosity in all stages, whereas the vascular canals of the fibula and radius are mostly longitudinally oriented and reduced in density, resulting in a low cortical porosity. In addition, our

samples indicate that the humerus and tibia have higher cortical porosity than that of other limb bones, but the parallel-fibered bone with growth marks near the periphery appeared earlier in the humerus than in other elements (IVPP V26544).

The adult stage ~~was~~ usually represented by the presence of an EFS [38], which has been identified in some dicynodonts, including *Placerias* [43] and *Kannemeyeria* [44]. There is still no typical EFS detected in *Lystrosaurus*. Ray et al. (2005) found parallel-fibered bone in the outer cortex of a dorsal rib and a thin layer of lamellar bone in a vertebra at the adult stage, but they did not find a typical EFS with closely spaced LAGs near the periphery. In our samples, the parallel-fibered bone in the outer cortex appeared in the dorsal rib and other elements, representing the subadult stage here (Fig 4). Although there is no typical EFS detected, the presence of more LAGs and parallel-fibered bone tissue near the periphery suggests that it grew very slowly before it died.

Botha et al. (2016) found that all Early Triassic *Lystrosaurus* from South Africa have no more than one observed LAG and no parallel-fibered bone in the outermost cortex, which suggests that they did not reach skeletal maturity [19]. However, LAGs seem to be more commonly distributed in *Lystrosaurus* from the Early Triassic of China. Within the seven individuals, t[illegible] have more than one LAG and two have one LAG for each. The presence of at least one LAG in *Lystrosaurus* from China has a humerus ranging from 10.3 to 15.6 cm, and a femur ranging from 12 to 19 cm, which is much larger than the sampled one LAG *Lystrosaurus* from the Early Triassic of Africa (i.e. SAM-PK-K8, SAM-PK-[illegible]1 have a humeral length of about 10 cm) [19, 22, 30]. This suggests that our sampled specimens are larger than the sampled individuals of *Lystrosaurus* from the Early Triassic of Africa.

Bone histology in the four *Lystrosaurus* species from the Lower Triassic of South Africa

is structurally similar [30, 45], which also occurs in our samples. However, IVPP V26545 ~~shows~~ a large humerus (15 cm) but ~~with~~ only one LAG that is supposed to be subadult~~,~~ ~~but~~ all other individuals with ~~the~~ humerus larger than 12 cm have more than two LAGs to be among adult stage (Table 1). In addition, the ratio of humerus ~~length~~ to ulna length is about 1.4, which is larger than that of IVPP RV3501(1.2). This may indicate a different species of *Lystrosaurus*, which needs to ~~clarify~~ in the future.

The presence of LAGs and the large size of *Lystrosaurus* from China suggest that many *Lystrosaurus* reached at least the subadult stage, unlike Triassic *Lystrosaurus* from Africa where subadults are rare [19]. This difference may be caused by differing environmental conditions in these areas[23, 46]. Although broadly connected on the Pangaean supercontinent, the Karoo Basin and North China were geographically distant in the Early Triassic, being located in mid-to-high latitudes of the southern and northern hemispheres, respectively [47]. Although evidence from calcic vertisols indicates some region of Karoo Basin ~~to~~ a wetting trend towards the *Daptocephalus*-*Lystrosaurus* Assemblage-Zone boundary [9], the hot and arid environment of Karoo Basin in the Early Triassic had been widely accepted [6, 7, 45]. These harsh environmental conditions may have resulted in high mortality rates of *Lystrosaurus*, with most individuals dying during the early stage [19]. By comparison, the climate is inferred to have been semiarid-subhumid in the Early Triassic of North China [34, 48, 49], which may indicate better environmental conditions than that in South Africa.

Another important issue is whether the body size of *Lystrosaurus* was reduced in the Early Triassic due to the harsh environment such as arid weather. Body size reduction has been shown in non-mammalian eutheriodont therapsids during the end-Permian mass extinction [11], and a

reduct[illegible]s also observed in *Lystrosaurus* from the Karoo Basin of South Africa, but this may the result of the young stage of the body (indicates by rare LAGs) in the Early Triassic based on bone microstructure [19]. Interestingly, the body size of *Lystrosaurus* from the lower section of the Guodikeng Formation (late Permian), such as *L. youngi* is smaller than the upper section of Jiucaiyuan Formation, contrasting with the condition in South Africa [32]. This may indicate that the known materials of *L. youngi* represent immature individuals, whereas later Chinese *Lystrosaurus* from Early Triassic are at least subadult. However, the bone microstructure of *Lystrosaurus* from the Guodikeng Formation is poorly preserved, and more material needs to be assessed in the future to confirm this hypothesis.

The lifestyle of *Lystrosaurus* has been argued to be aquatic/semiaquatic [22], fully terrestrial, or burrowing [20, 21]. In recent decades, this controversy has concentrated on aquatic or ~~borrowing~~ habits, which could benefit its survival in the hot environments of the Early Triassic. The aquatic/semiaquatic lifestyle was supported by the bone microstructure showing a thick cortex and porous vascular canals [22], and linear discriminant analysis based on the compactness profile and body size parameters in extant animals [50, 51]. The cortical thicknesses of most samples described here are between 20% - 40%, which is similar to *Lystrosaurus* from South Africa [22]. [illegible] the main difference is the sampled dorsal rib has dense erosional ca[illegible]es in the inner and mid cortex and a thin layer of compact outer cortex (Figs. 4, 6), which differs from the thick cortex (27-37%) in *Lystrosaurus* from Africa [22]. In sum, our results also show that *Lystrosaurus* has a thick cortex as in ~~extent~~ aquatic animals, such as *Hippopotamus* [52]. However, the thick cortex is not only shown in aquatic animals but also reported in extant burrowing animals and nonmammalian therapsids [41]. Therefore, thick cortex could not provide confident evidence ~~the~~ aquatic or semiaquatic lifestyle ~~of~~

Lystrosaurus. Moreover, Juvenile *Lystrosaurus* has been found inside burrow casts indicating that they were active burrowers [24, 25].

Whether *Lystrosaurus* had a substantial aquatic component to its lifestyle in addition to burrowing could be elucidated by analyzing stable isotopes in the future. Previous studies suggest that aquatic and semi-aquatic vertebrates have $\delta^{18}O$ values significantly lower than those of coexisting terrestrial animals [53-55]. Oxygen and calcium isotopes all proved spinosaurids to be aquatic or semiaquatic like modern crocodiles [56, 57], and they could also provide more evidence for the lifestyle of *Lystrosaurus*. Furthermore, $\delta^{18}O$ isotopes have been already used in some late Permian ~~dicynodontians~~, and the result shows that *Endothiodon* may have a semi-aquatic lifestyle, whereas *Tropidostoma* was ~~a terrestrial species~~ [58].

Conclusion

Here we have studied the bone histology of *Lystrosaurus* from the Early Triassic of China based on thin sections of seven individuals. The results indicate that *Lystrosaurus* grew rapidly in the early stages as previously shown in *Lystrosaurus* from South Africa and India. However, the presence of LAGs in the largest individual suggests that the growth of *Lystrosaurus* was interrupted in the late stage. LAGs are rare in *Lystrosaurus* from South Africa but they are more abundant in the *Lystrosaurus* from Xinjiang, China. We propose that most *Lystrosaurus* from Xinjiang reached subadult and adult stages, whereas those from South Africa died during their juvenile stage. This distinction may be caused by different environmental conditions, but more samples from the Early Triassic of China are needed to test this hypothesis in the future.

Acknowledgment

We thank Zhang Shukang and Wu Rui for helping to make bone sections, Chu Daoliang for his helpful discussion. We thank Jennifer Botha, Megan Whitney, and Zoe Kulik for their comments on a previous edition of this paper. Megan Whitney also reviewed this version and have provided more constructive and useful comments. We thank the editor Jörg Fröbisch, and the reviewer Christian F. Kammerer, and two other anonymous reviewers for their very helpful comments on this manuscript.

References

1. Maxwell WD. Permian and Early Triassic extinction of non-marine tetrapods. Palaeontology. 1992;35(3):571-83.

2. Erwin DH. The Permo-Triassic extinction. Nature. 1994;367(6460):231-5.

3. Stanley SM. Estimates of the magnitudes of major marine mass extinctions in earth history. Proc Natl Acad Sci. 2016;113(42):E6325-E34. doi: 10.1073/pnas.1613094113

4. Retallack GJ. Permian and Triassic greenhouse crises. Gondwana Res. 2013;24(1):90-103. doi: org/10.1016/j.gr.2012.03.003.

5. Sun Y, Joachimski MM, Wignall PB, Yan C, Chen Y, Jiang H, et al. Lethally hot temperatures during the Early Triassic greenhouse. Science. 2012;338(6105):366-70. doi: 10.1126/science.1224126.

6. Smith RMH, Botha-Brink J. Anatomy of a mass extinction: Sedimentological and taphonomic evidence for drought-induced die-offs at the Permo-Triassic boundary in the main Karoo Basin, South Africa. Palaeogeogr Palaeoclimatol Palaeoecol. 2014;396(3):99–118.

7. MacLeod KG, Quinton PC, Bassett DJ. Warming and increased aridity during the earliest Triassic in the Karoo Basin, South Africa. Geology. 2017;45(6):483-6. doi: 10.1130/G38957.1

8. Bernardi M, Petti FM, Benton MJ. Tetrapod distribution and temperature rise during the Permian-Triassic mass extinction. Proc R Soc Lond B Biol Sci. 2018;285(1870):20172331. doi: doi:10.1098/rspb.2017.2331.

9. Gastaldo RA, Kus K, Tabor N, Neveling J. Calcic Vertisols in the upper Daptocephalus Assemblage Zone, Balfour Formation, Karoo Basin, South Africa: Implications for Late Permian Climate. J Sediment Res. 2020;90(6):609-28. doi: 10.2110/jsr.2020.32

10. Song H, Tong J. Mass extinction and survival during the Permian-Triassic crisis. Earth Sci. 2016;41(6):901-18. (in chinese with English abstract)

11. Huttenlocker AK. Body size reductions in nonmammalian eutheriodont therapsids (Synapsida) during the end-Permian mass extinction. PLoS One. 2014;9(2):e87553. doi: 10.1371/journal.pone.0087553.

12. Chu D, Tong J, Song H, Benton MJ, Song H, Yu J, et al. Lilliput effect in freshwater ostracods during the Permian–Triassic extinction. Palaeogeogr Palaeoclimatol Palaeoecol. 2015;435:38-52. doi: https://doi.org/10.1016/j.palaeo.2015.06.003.

13. Smith RM, Ward PD. Pattern of vertebrate extinctions across an event bed at the Permian-Triassic boundary in the Karoo Basin of South Africa. Geology. 2001;29(12):1147-50. doi: org/10.1130/0091-7613(2001)029<1147:POVEAA>2.0.CO;2.

14. Smith R, Botha J. The recovery of terrestrial vertebrate diversity in the South African Karoo Basin after the end-Permian extinction. C R Palevol. 2005;4(6-7):623-36. doi: org/10.1016/j.crpv.2005.07.005.

15. Colbert EH. The distribution of *Lystrosaurus* in Pangaea and its implications. Geobios. 1982;15:375-83. doi: org/10.1016/S0016-6995(82)80126-5.

16. Cosgriff JW, Hammer WR, Ryan WJ. The Pangaean Reptile, *Lystrosaurus maccaigi*, in the Lower Triassic of Antarctica. J Paleontol. 1982;56(2):371-85.

17. Fröbisch J. Composition and similarity of global anomodont-bearing tetrapod faunas. Earth-Sci Rev. 2009;95(3):119-57. doi: https://doi.org/10.1016/j.earscirev.2009.04.001.

18. Botha J, Smith RMH. *Lystrosaurus* species composition across the Permo-Triassic boundary in the Karoo Basin of South Africa: *Lystrosaurus* across the PTB. Lethaia. 2007;40(2):125-37. doi: org/10.1111/j.1502-3931.2007.00011.x.

19. Botha-Brink J, Codron D, Huttenlocker AK, Angielczyk KD, Ruta M. Breeding young as a survival strategy during earth's greatest mass extinction. Sci Rep. 2016;6(1):24053. doi: org/10.1038/srep24053.

20. King GM, Cluver MA. The aquatic *Lystrosaurus*: An alternative lifestyle. Hist Biol. 1991;4(3-4):323-41. doi: org/10.1080/08912969009386548.

21. King GM. The aquatic *Lystrosaurus*: a palaeontological myth. Hist Biol. 1991;4(3-4):285-321.

22. Ray S, Chinsamy A, Bandyopadhyay S. *Lystrosaurus murrayi* (therapsida, dicynodontia): bone histology, growth and lifestyle adaptations. Palaeontology. 2005;48(6):1169–85. doi: org/10.1111/j.1475-4983.2005.00513.x.

23. Ray S, Mukherjee D, Bandyopadhyay S. Growth patterns of fossil vertebrates as deduced from bone microstructure: case studies from India. J Biosci. 2009;34(5):661-72. doi: org/10.1007/s12038-009-0055-x.

24. Botha-Brink J. Burrowing in *Lystrosaurus*: preadaptation to a postextinction environment? J

Vert Paleontol. 2017;37(5):e1365080. doi: 10.1080/02724634.2017.1365080.

25. Modesto SP, Botha-Brink J. A burrow cast with *Lystrosaurus skeletal* remains from the Lower Triassic of South Africa. Palaios. 2010;25(4):274-81. doi: org/10.2110/palo.2009.p09-077r.

26. Tałanda M, DzięCioł S, Sulej T, NiedźWiedzki G. Vertebrate burrow system from the Upper Triassic of Poland. Palaios. 2011;26(2):99-105. doi: org/10.2110/palo.2010.p10-024r.

27. Hasiotis ST, Wellner RW, Martin AJ, Demko TM. Vertebrate Burrows from Triassic and Jurassic Continental Deposits of North America and Antarctica: Their Paleoenvironmental and Paleoecological Significance. Ichnos. 2004;11(1-2):103-24. doi: org/10.1080/10420940490428760.

28. Özkurt Ö, Yiğit N, Çolak E, Sözen M, Gharkheloo M. Observations on the Ecology, Reproduction and Behavior of *Spermophilus* Bennett, 1835 (Mammalia: Rodentia) in Turkey. Turk J Zool. 2005;29:91-9.

29. Chinsamy A, Rubidge BS. Dicynodont (Therapsida) bone histology: phylogenetic and physiological implications. Palaeontol Afr. 1993;30:97-102.

30. Botha-Brink J, Angielczyk KD. Do extraordinarily high growth rates in Permo-Triassic dicynodonts (Therapsida, Anomodontia) explain their success before and after the end-Permian extinction? Zool J Linn Soc. 2010;160(2):341-65. doi: org/10.1111/j.1096-3642.2009.00601.x.

31. Li J-L. *Lystrosaurus* of China. Vertebr Palasiat. 1988;26(4):241-9. (in Chinese with English abstract)

32. Liu J, Li J, Cheng Z. The *Lystrosaurus* fossils from Xinjiang and their bearing on the terrestrial Permian Triassic boundary. Vertebr Palasiat. 2002;40(4):267-75. (in Chinese with English abstract)

33. Camp J, and Liu J. 2011. The taxonomy and cranial morphology of Chinese *Lystrosaurus*. [Abstract] Supplement to online Journal of Vertebrate Paleontology, 71st Annual Meeting Society

of Vertebrate Paleontology: 82

34. Yang W, Feng Q, Liu Y, Tabor N, Miggins D, Crowley JL, et al. Depositional environments and cyclo- and chronostratigraphy of uppermost Carboniferous–Lower Triassic fluvial–lacustrine deposits, southern Bogda Mountains, NW China — A terrestrial paleoclimatic record of mid-latitude NE Pangea. Global Planet Change. 2010;73(1):15-113. doi: org/10.1016/j.gloplacha.2010.03.008.

35. Thomas SG, Tabor NJ, Yang W, Myers TS, Yang Y, Wang D. Palaeosol stratigraphy across the Permian–Triassic boundary, Bogda Mountains, NW China: Implications for palaeoenvironmental transition through earth's largest mass extinction. Palaeogeogr Palaeoclimatol Palaeoecol. 2011;308(1):41-64. doi: org/10.1016/j.palaeo.2010.10.037.

36. Wan M, Yang W, Wang JJ. A new *Protophyllocladoxylon* wood from the Induan (Lower Triassic) Jiucaiyuan Formation in the Turpan–Hami Basin, southern Bogda Mountains, Rev Palaeobot Palynol. northwestern China. 2019;267:62-72.

37. Horner JR, de Ricqlès A, Padian K. Variation in dinosaur skeletochronology indicators: implications for age assessment and physiology. Paleobiology. 1999;25(3): 295-304. doi: org/10.1017/S0094837300021308.

38. Chinsamy-Turan A. The microstructure of dinosaur bone. Baltimore: Johns Hopkins University Press; 2005. 195 p.

39. Donath K, Rohrer M. Bone Sectioning Using the Exakt System. In: An YH, Martin KL, editors. Handbook of Histology Methods for Bone and Cartilage: Springer; 2003. pp. 243-52.

40. Francillon-Viellot H, de Buffrénil V, Castanet J, Géraudie J, Meunier FJ, Dire JY, et al. Microstructure and mineralization of vertebrate skeletal tissues. In: Carter JG, editor. Skeletal

biomineralization: patterns, processes and evolutionary trends. New York: Van Nostrand Reinhold; 1990. p. 471-530.

41. Chinsamy-Turan A. The microstructure of bones and teeth of nonmammalian therapsids. In: Chinsamy-Turan A, editor. Forerunners of mammals: radiation, histology, biology. Bloomington, Indiana: Indiana University Press; 2012. p. 65-90.

42. Horner JR, De Ricqles A, Padian K. Long bone histology of the hadrosaurid dinosaur *Maiasaura peeblesorum*: Growth dynamics and physiology based on an ontogenetic series of skeletal elements. J Vert Paleontol. 2000;20(1):115-29. doi: org/10.1671/0272-4634(2000)020[0115:LBHOTH]2.0.CO;2.

43. Green JL, Schweitzer MH, Lamm ET. Limb bone histology and growth in *Placerias hesternus* (Therapsida: Anomodontia) from the Upper Triassic of North America. Palaeontology. 2010;53(2): 347-64. doi: org/10.1111/j.1475-4983.2010.00944.x.

44. Ray S, Botha-Brink J, Chinsamy-Turan AJ. Dicynodont growth dynamics and lifestyle adaptations. In: Chinsamy-Turan A, editor. The forerunners of mammals: radiation, histology and biology. Bloomington: Indiana University Press; 2012. p. 121-48.

45. Botha J, Huttenlocker AK, Smith RMH, Prevec R, Viglietti P, Modesto SP. New geochemical and palaeontological data from the Permian-Triassic boundary in the South African Karoo Basin test the synchronicity of terrestrial and marine extinctions. Palaeogeogr Palaeoclimatol Palaeoecol. 2020;540:109467. doi: org/10.1016/j.palaeo.2019.109467.

46. Köhler M, Marín-Moratalla N, Jordana X, Aanes R. Seasonal bone growth and physiology in endotherms shed light on dinosaur physiology. Nature. 2012;487(7407):358-61. doi: 10.1038/nature11264.

47. Muttoni G, Gaetani M, Kent DV, Sciunnach D, Angiolini L, Berra F, et al. Opening of the Neo-Tethys Ocean and the Pangea B to Pangea A transformation during the Permian. GeoArabia. 2009;14(4):17-48.

48. Liu D, Zhang C, Yang D, Pan Z, Kong X, Huang Z, et al. Petrography and geochemistry of the Lopingian (Upper Permian)-Lower Triassic strata in the southern Junggar and Turpan basins, NW China: implications for weathering, provenance, and palaeogeography. Int Geol Rev. 2019;61(8):1016-36. doi: org/10.1080/00206814.2018.1490932.

49. Tabor NJ, Sidor CA, Smith RMH, Nesbitt SJ, Angielczyk KD. Paleosols of the Permian-Triassic: proxies for rainfall, climate change and major changes in terrestrial tetrapod diversity. J Vert Paleontol. 2017;37(sup1):240-53. doi: org/10.1080/02724634.2017.1415211.

50. Germain D, Laurin M. Microanatomy of the radius and lifestyle in amniotes (Vertebrata, Tetrapoda). Zool Scr. 2005;34(4):335-50. doi: org/10.1111/j.1463-6409.2005.00198.x.

51. Canoville A, Laurin M. Evolution of humeral microanatomy and lifestyle in amniotes, and some comments on palaeobiological inferences. Biol J Linn Soc. 2010;100(2):384-406. doi: org/10.1111/j.1095-8312.2010.01431.x.

52. Wall WP. The Correlation between High Limb-Bone Density and Aquatic Habits in Recent Mammals. J Paleontol. 1983;57(2):197-207.

53. Bocherens H, Koch PL, Mariotti A, Geraads D, Jaeger J-J. Isotopic biogeochemistry (^{13}C, ^{18}O) of mammalian enamel from African Pleistocene hominid sites. Palaios. 1996;11(4):306-18. doi: org/10.2307/3515241

54. Fricke HC, Rogers RR. Multiple taxon–multiple locality approach to providing oxygen isotope evidence for warm-blooded theropod dinosaurs. Geology. 2000;28(9):799-802. doi:

org/10.1130/0091-7613(2000)28<799:MTLATP>2.0.CO;2

55. Clementz MT, Holroyd PA, Koch PL. Identifying aquatic habits of herbivorous mammals through stable isotope analysis. Palaios. 2008;23(9):574-85. doi: org/10.2110/palo.2007.p07-054r

56. Hassler A, Martin J, Amiot R, Tacail T, Godet FA, Allain R, et al. Calcium isotopes offer clues on resource partitioning among Cretaceous predatory dinosaurs. Proc R Soc Lond B Biol Sci. 2018;285(1876):20180197. doi: org/10.1098/rspb.2018.0197.

57. Amiot R, Buffetaut E, Lécuyer C, Wang X, Boudad L, Ding Z, et al. Oxygen isotope evidence for semi-aquatic habits among spinosaurid theropods. Geology. 2010;38(2):139-42. doi: org/10.1130/G30402.1.

58. Rey K, Day MO, Amiot R, Fourel F, Luyt J, Lécuyer C, et al. Stable isotopes (δ18O and δ13C) give new perspective on the ecology and diet of Endothiodon bathystoma (Therapsida, Dicynodontia) from the late Permian of the South African Karoo Basin. Palaeogeogr Palaeoclimatol Palaeoecol. 2020;556:109882. doi: org/10.1016/j.palaeo.2020.109882.

Supporting information

S1 Fig. The accurate sampled positions of all specimens in this study. **A**, IVPP V26543, arrow denotes the sampled position of the tibia; (**B, C)** IVPP V26544. **B**, the sampled positions of the tibia and fibula; C, the sampled position of the humerus; **D**, IVPP V26542, the distal end of the femur that sampled; (**E-G)** IVPP V26545. **E**, the sampled position of the radius; F, the sampled position of the fibula; **G**, the sampled position of the rib; **H**, IVPP V26547, arrow denotes the sampled position of the femur; **I**, IVPP V26546, arrow denotes the sampled position of the femur; **J**, IVPP V26548, arrow denotes the sampled position of the rib.

S2 Fig. Measurements of the cortical thickness in all thin sections. A, IVPP V26543, tibia; **B**, IVPP V26544 tibia; **C**, IVPP V26544, humerus; **D**, IVPP V26544, fibula; **E**, IVPP V26542, femur; **F**, IVPP V26545, fibula; **G**, IVPP V26545, radius; **H**, IVPP V26546, femur; **I**, IVPP V26547, femur. The yellow lines denote measurements of the cortical thickness. The red lines denote measurements of cross-sectional diameters, and the green circles denote outlines of the medullary cavities.

S3 Fig. Bone microstructure and the sampled areas for calculating cortical porosity in all thin sections.

Dear Editor and Reviewers:

Thank you for your letter and for the reviewers' comments concerning our manuscript entitled "Bone histology of *Lystrosaurus* (Therapsida: Dicynodontia) from the Lower Triassic of North China, and its implication for lifestyle and environments after the end-Permian extinction" (ID: PONE-D-20-17684). Those comments are all valuable and very helpful for revising and improving our paper, as well as important for highlighting the significance of our work. We have studied the comments carefully and made a number of corrections to address them, which we hope to meet with approval. The revised portions of the MS were marked by red colors. The main corrections to the paper and the responses to the editor and reviewer's comments are as following:

Editor comments:

Please pay detailed attention to the comments made by ALL reviewers but specifically to the comments and criticism of Reviewer 4. For this manuscript to be acceptable for publication in Plos One, it is essential that you provide additional comparative data with regards to South African *Lystrosaurus* specimens as well as reconsider the interpretation of your own data, specifically with respect to the evidence for an external fundamental system (EFS), which is rather unconvincing based on the currently provided data.

REPLY: We have modified our MS carefully according to all reviewers' comments. We have also added more comparisons between Chinese *Lystrosaurus* and South African *Lystrosaurus*, including both bone microstructure and body size. In addition, We have deleted the statement of the possible presence of EFS in *Lystrosaurus* based on current evidence, and we just mentioned that "there is still no typical EFS found in *Lystrosaurus*".

Reviewer1

introduction line 4: It might be good to note that there is some debate about this, at least for the extinction in the Karoo Basin, such as Gastaldo et al. (2020; Journal of Sedimentary Research) and references cited therein

REPLY: We have added the debate and related references in the MS.

page 3 line 2: instead of curve, to would recommend using downturned, since the shape is more angular than curved in most *Lystrosaurus* species.

REPLY: Corrected.

page 3 line 3: The report of *Lystrosaurus* in Zambia is incorrect (See Angielczyk et al. 2014; Early Evolutionary History of the Synapsida book), so I recommend not citing the King and Jenkins paper. Frobisch (2009; Earth-Science Reviews) would be a more

comprehensive paper to cite here (although, it still includes the incorrect Zambian record).

REPLY: We have changed the citation as suggested.

page 3 line 6: It might be worth noting that the semi-aquatic ecology of *Lystrosaurus* has been disputed including using bone histology data (e.g., King 1991; King and Cluver 1991, both Historical Biology; Botha-Brink and Angielczyk 2010, ZJLS).

REPLY: We have added more citations here.

page 3, paragraph 2: A couple comments here. 1) remove 'the' before minaturization. 2) I suggest a little re-phrasing of the results from the Botha-Brink et al. (2016) paper. That paper did indicate that LAGs were more common in Permian specimens, which was interpreted as evidence that the Permian species had a more extended ontogeny that extended over several years. This contrasts with the prevalence of small individuals with few or no LAGs in the Triassic species, which was part of the evidence suggesting breeding young (the other part of the evidence were the demographic simulations that suggested that breeding young could be an effective survival strategy when life expectancy was low).

REPLY: Modified as suggested.

material and methods line 6: I'm not sure if the specimens you sectioned had associated skulls. If they do not, I recommend saying that ID to species level is currently not possible because Chinese Lystrosaurus taxonomy (and Lystrosaurus taxonomy in general) is currently based on skull characters. If they have skulls, you might say that you didn't try to ID them to species level given the uncertainties about Chinese Lystrosaurus species-level taxonomy that you mentioned above.

REPLY: Thank you for your suggestion. Our specimens only include postcranial materials. Modified as suggested.

material and methods line 8: Morphology refers to macroscopic features, whereas histology refers to microscopic tissue structure. So you should say: "...South Africa is quite similar, although more LAGs..." You should also make this change in the next sentence when you make your predictions about the Chinese species.

REPLY: Corrected. Furthermore, we moved this part to the discussion as suggested by another reviewer.

material and methods line 10: Identical histology might be too strong. Similar is probably a better prediction, assuming there aren't differences due to environmental conditions (as you note).

REPLY: Modified as suggested.

Materials and methods, paragraph 2, line 3: complete instead of completed; also at the end of this sentence, change bones to bone. It would be good to note whether and how the specimens were documented before sectioning (e.g., e.g., if photographs or measurments were taken, if molds/casts were made, etc.).

REPLY: We have added this information in paragraph 2.

Table 1: How did you assign your specimens to growth stages? Is this something that was done a priori, and if so what was it based on (Size? Degree of ossification and development of muscle scars and articular surfaces? Something else?). You should include this information in the methods section.

REPLY: Growth stages were identified based on bone histological information, and we have added this information in the footnotes of Table 1.

Page 6, paragraph 1: This paragraph uses a mixture of present tense and past tense. Please revise it so it consistently uses past tense.

REPLY: Corrected.

Page 6, line 2-3: You should state why it was difficult to measure cortical porosity for a whole cross section. Was it because of breakage, differential preservation of the bone tissue in different areas, something else? Also, It might be good to elaborate a bit on how you chose areas to measure when that was necessary, maybe the average number of areas you needed to use (in addition to the maximum you report), and whether using different numbers of areas had any effect on the results.

REPLY: We have added this information and modified the sentence as "It was difficult to obtain the cortical porosity of a whole cross-section for each specimen because of partially breakage or poor preservation in some areas of these sections."

Cortical porosity is usually reduced from the inner region to outside, but they are usually (not always) similar on all sides (medial, lateral, anterior, posterior). We subsampled ten areas from the inner to the outermost cortex in all sections (except IVPP V26546 was selected 12 areas) to calculate average vascular porosity. The sampled areas were chosen from the inner region to the periphery on different parts of the whole cross-sections, so they could reflect the value of cortical porosity. However, some bone sections show unclear bone histology in some regions, which may affect the results. Cortical porosities of those poorly preserved bone sections were labeled to be "estimated values". We have provided the figures showing the sampled area (S3 Fig).

Fig. 1: I recommend reorganizing this figure slightly so that the overview of the

specimen showing the focal areas is in panel A (similar to Fig. 2).

REPLY: Modified as suggested.

juvenile line 2: change to: All the corticies are composed of a woven-fibered bone matrix, with a…

REPLY: Corrected. We have modified this sentence as “All the cortices are composed of fibrolamellar tissue, with a high number of vascular canals, abundant globular osteocyte lacunae, and no LAGs, suggesting a rapid, constant growth rate.”

juvenile, line 5: when you mention cortical thickness and porosity values here and elsewhere in the text, you should call out Table 1. It would also be good if you can be more consistent in mentioning those values for the different elements in the text.

REPLY: We have added this information throughout the manuscript.

Fig. 2 and Fig. 2 caption: H and I should be switched in the figure in the caption given that you discuss what is currently I (but should be H) first. Also, panel O is a little dark. Can you adjust the brightness a bit on the image?

REPLY: Modified as suggested.

Fig. 3: Please add boxes to the overview image in panel A to show where the higher magnification images are located.

REPLY: Added.

Page 13, line 3: I think you mean: “The number and diameters of the vascular canals in the narrow region...”

REPLY: Thank you for pointing out. Corrected.

Fig. 5 : Please add boxes to the overview image in panel A to show where the higher magnification images are located.

REPLY: Added.

Page 16, line 2: An EFS was reported in the dicynodont Placerias by Green et al. (2010; Palaeontology).

REPLY: We have added more references here.

Fig. 6 : Please add boxes to the overview image in panel A to show where the higher magnification images are located.

REPLY: We have added a box in panel A, showing the position of B, and also a box in panel B, showing the position of C.

page 18, first full paragraph, line 7: Bone histology has been looked in a pretty wide range of dicynodonts now, and an EFS has only been observed in a couple of instances. Therefore, it seems odd to suggest that it may be present in Lystrosaurus, especially when you didn't find any evidence of one in any of your specimens.

REPLY: We have modified this paragraph and conclusion here. The modified statement is "There is still no typical EFS detected in *Lystrosaurus*."

Page 19, line 9: As noted above, some research has suggested wetter conditions in the Karoo at this time. It would be good to note that possibility here as well.

REPLY: we have added new references on wetter conditions.

Page 19, last line: It might be good to qualify this a bit by stating that most collected Early Triassic specimens are at least subadults. My sense is that *Lystrosaurus* hasn't been documented in a comprehensive enough way to have an unbiased sense of the relative abundances of different size classes.

REPLY: Modified as suggested.

Page 20, first full paragraph: The other thing that's important to note here is that the bone microstructure of *Lystrosaurus* doesn't seem to differ all that much from other dicynodonts (e.g., Botha-Brink and Angielczyk 2010). So either that would imply that most dicynodonts were semi-aquatic or that *Lystrosaurus* was terrestrial like other dicynodonts (with dicynodonts in general showing some fairly consistent differences in microstructure to modern terrestrial tetrapods).

REPLY: Our samples support *Lystrosaurus* has a thick cortex, as in *Lystrosaurus* of Africa. But thick cortex is not only seen in aquatic animals but also shown in extant burrowing animals. Therefore, thick cortex could not provide confident evidence of the aquatic or semiaquatic lifestyle of *Lystrosaurus*, so we did not discuss the lifestyle of other dicynodonts. We have provided more discussion on *Lystrosaurus* lifestyle and how to solve this problem.

Page 20, first full paragraph, next to last sentence: It would be good to cite Rey et al. (2020; Palaeo3) here since it is a study that uses stable isotope data to make inferences about habitat preferences of a diynodont.

REPLY: Thank you for the information. Cited.

Reviewer 2.

There are some critical flaws that I think could only be addressed with an adequate comparative dataset of Permian *Lystrosaurus* specimens, and it's unclear to me whether the research team has adequately surveyed Permo-Triassic therapsid histology at all. Based on the abundant material of Permo-Triassic *Lystrosaurus* that I've thin-sectioned and seen firsthand, the South African material is nearly identical to the Chinese specimens. This study is hindered by a lack of any comparative specimens, and would further benefit from a more quantitative scientific approach.

REPLY: Thank you for your suggestions. We have added more comparison to *Lystrosaurus* from South Africa on both bone histology and body sizes. We admit that our samples are limited, but they do show some interesting and clear features that unlike those of *Lystrosaurus* from South Africa, and we will provide more data on bone histology and other parts of Chinese *Lystrosaurus* in the future.

Reviewer #3: The frequent presence of LAGs in the Chinese specimens is interesting and well worth publishing. I have made a number of copy edits and comments on some unclear parts of the manuscript in the attached file, which should be addressed before this contribution can be accepted.

REPLY: Thank you for your comments and detailed corrections for the grammar in the MS. We have checked the whole text and corrected all these things. Here are our replies to the main comments.

Page 4 Line 72. “Therefore, here we suppose that *Lystrosaurus* from the Taodonggou area of Xinjiang China should have identical morphology on bone histology even in different species. However, they may have distinct bone histological information from other places due to different environmental conditions”
Comment: This statement is unclear. Why should they have identical morphology?

REPLY: we have changed the word “identical” to “similar”. And moved this part to the discussion section. Here we mean that the previous work on Africa *Lystrosaurus* shows similar bone microstructure in different species, and Chinese *Lystrosaurus* may also have similar bone microstructure even in different species.

Page 6 Line 98. “Therefore, we subsampled areas (as many as ten) from the inner to the outermost cortex to calculate average vascular porosity.”
Comment: What is the minimum?

REPLY: We subsampled ten areas in all the sections. We have provided the sampled

areas on photographs in the new supplementary files (S3 Fig).

Page 11. Line 206 The number and diameter of the vascular canals in the narrow region is significantly reduced near the periphery.
COMMENT: This does not make sense to me.

REPLY: Thank you for pointing out. We have modified this sentence to "The number and diameter of the vascular canals are significantly reduced near the periphery."

Page 12. Line 210 "Trabeculae? In the trabecular region?"
REPLY: Yes. We have corrected this word.

Page 19 Recent papers by Kevin Rey on South African oxygen isotope work in dicynodonts would be worth citing and possibly discussing here.
REPLY: Thank you for your useful information. We have added this new reference here.

Reviewer #4:
This study describes the bone histology of seven Early Triassic *Lystrosaurus* from Xinjiang, China. The authors describe an ontogenetic series of rapid growth, with some periods of interrupted growth, and eventually a slowed, adult-stage growth. The authors compare these findings to those from the bone histology of South African *Lystrosaurus* and detail that although signals of arrested growth are rarely reported from South Africa, they were found in their sample. This suggests that perhaps the samples from South Africa are still in the juvenile/subadult stage. The authors suggest that this finding could be explained by environmental conditions that are thought to have been more extreme in South Africa than in China during the Early Triassic resulting in a greater die-off of individuals before skeletal maturity in South Africa. The authors also discuss their findings as it relates to the lifestyle of *Lystrosaurus*, refuting previous hypotheses that the genus was aquatic/semiaquatic.

I have reviewed a previous version of this manuscript and found the grammar and organization to be much improved. There were still some line edits of which I have added to my marked version of the manuscript. I have also added comments that are both specific and general to this document, but in addition, I have a few general comments about the manuscript. I think the figures are incredibly well presented and it is important this data is published. However, I think there are very major revisions in the discussion points/conclusions as well as some methodology that requires attention prior to publication.

Generally, I think the authors need to make a more compelling argument that their sample represents an ontogenetic series, independent of the histology. Table 1 I believe is supposed to address this, but the organization is difficult for the reader to follow, especially when I can't compare a single element across the sample. To address this I think the authors, using more complete specimens and previously established ratios

between elements, could come up with estimates of a single element for each individual in their sample. Alternatively, a single figure that images all of the samples at the same scale would help the reader confirm the authors' designations of ontogenetic stages. I believe there is a supplementary figure, however, this figure was not included in my review document so I am unable to say if it would help to understand the relative sizes of these individuals. This is the only reason why I answered that not all data was available. In such a figure, I would also include images of the largest South African and Indian Lystrosaurus elements so that the reader can see just how much larger the Chinese specimens are.

REPLY: Thank you for your suggestions. Sorry for unmodified this part last time. The postcranial skeleton of *Lystrosaurus* was not well studied, and we did not know the variation between different species and ontogeny, so we did not estimate values previously. We have rechecked more *Lystrosaurus* materials and provided estimated values based on element ratios of one complete skeleton (IVPP RV35012), please see the footnotes of Table 1 for detail information.

In addition, sorry that we forgot to upload figure S1. In this figure, we have provided the accurate sampled positions of each element.

The methodology still requires significant revision/clarity. I would review previous histological papers that measure cortical thickness. I think for cortical thickness, presenting a range is more important that an average, especially in some specimens that are asymmetrically arranged. Cortical porosity also needs to be addressed. The description as it stands does not seem systematic and completely subjective. Subsampling vascularity is ok, but the locations (medially, laterally, posterior, anterior, endosteally, mid-cortex, periosteally) need to be systematically sampled similarly across the entire sample. Without this, it is difficult to review the values reported.

REPLY: We have provided ranges for all cortical thickness and labeled the measurement positions in S2 Fig.

We have added more descriptions of how to measure cortical porosity. We choose ten areas that cover all the cortex. The sampled areas were from the inner region to the periphery on different parts of the whole cross-sections, and it indicates the average cortical porosity of the whole cross-section. Some bone sections show unclear bone histology in some regions, which may affect the results. Cortical porosities of those poorly preserved bone sections were labeled to be "estimated values". We have also provided the figures showing the areas that we choose. Please see S3 Fig.

In the descriptions differences in cortical porosity need to be explicitly stated. There are many instances where the percentage for one region (e.g. endosteal) of the cortex is reported and then the authors state there is a reduction in vascularity towards the periosteal surface, but no percentage is reported. Consistency is important in

interpreting the descriptions.

REPLY: We have added more percentages to make it clear.

It is important that the authors describe the anatomical context for the images (i.e. medial, lateral sides etc.). This can be done in the image itself (e.g. compass-style arrows) or in the figure caption.

REPLY: We checked all the specimens again and have added the directions for all the figures.

Finally, I do not think the data here is sufficient to refute the hypothesis that Lystrosaurus was aquatic. I agree entirely that it is unlikely *Lystrosaurus* was not aquatic, however, I don't believe the data as currently presented in this manuscript supports this. In fact, the thick cortical walls appear very similar to aquatic taxa, which is why previous studies have suggested an aquatic lifestyle. However, there are studies that demonstrate this is not always a reliable proxy for lifestyle (e.g. Houssaye et al. 2016). I would recommend one of two courses of action. Either remove the discussion of the aquatic lifestyle from this manuscript OR discuss how cortical thickness does not always accurately predict habitat.

REPLY: Sorry for the confusion. We did not refute the hypothesis that *Lystrosaurus* was aquatic. We prefer to keep this paragraph and have added more discussion here. Most of our samples support *Lystrosaurus* has a thick cortex, as in *Lystrosaurus* of Africa. But thick cortex is not only seen in aquatic animals but also shown in extant burrowing animals. Therefore, thick cortex could not provide confident evidence of the aquatic or semiaquatic lifestyle of *Lystrosaurus*. The lifestyle of *Lystrosaurus* is still controversial, but we provide more data for this issue.

Other comments in the text

Page 4 Line 9. Comment: This is an important discussion point, but should not be in the materials and methods section. Perhaps move to the discussion when reviewing the variation.

REPLY: We have moved this paragraph to the discussion part.

Page 5 Line 5. Comment: This is a confusing sentence. I think to simplify and to set up the descriptions nicely just describe the histological criteria used to assign a growth line (especially annuli, since you use that term later) vs. LAG.

REPLY: We have modified this sentence as "LAGs are growth marked lines that can be traced around the full circumference. The annuli correspond to periods of slow growth by the presence of parallel-fibered bone and flattened osteons".

Page 5 Table 1. Comment: I think this table is really confusing. It's hard to compare the sizes between specimens since there are different elements. See overall notes for suggestions.

REPLY: We have added the estimated values as suggested.

Page 5 Table 1. Comment: If you are going to estimate values you have to describe how those estimations were made.

REPLY: We have added an explanation of how to estimate values in the footnotes.

Page 7 line 7. Comment: This is very confusing and I'm not convinced it properly measures cortical thickness. Generally, I don't actually think the "diameter" measurements given in each of the description sections are properly stated either. I would suggest reporting mediolateral and anteroposterior widths (which is basically what I think the "diameter" measurements are?). I believe given the variability in cortical thickness around the bone, reporting the average is not helpful. Instead, maybe report a max and min %?

REPLY: We have modified the MS as suggested.

P10 L4 comment: Label these in the figure.

REPLY: Labeled as suggested.

P10L5 There are no secondary osteons.
Comment: “It looks like there is in fact a massive one in 1C”

REPLY: we assigned it to be an enlarged resorption cavity rather than secondary osteons for its extremely large size. No Haversian system was presented around the erosion cavity.

P10 L6. This has already been described earlier in the paragraph

REPLY: we have deleted it.

P10 L11.
I feel that wide-open is too casual of a term. Instead I would go with something like "enlarged canals". You could also just highlight the band of expanded vascularity with a bracket like: |-----|

REPLY: “enlarged canals” usually indicate secondary remodeling in our manuscript. But here it should be the primary bone. Therefore, we just described its shape here. We have added a bracket as suggested.

P11 Line 16 The humerus is subtriangular in cross-section (midshaft diameter: 21×25 mm) and more robust than the tibia.
Comment: I don't know what this means. In what way is it more robust? Greater cortical thickness?

REPLY: “subtriangular” means triangular but less angled. We have shown more figures in supplementary files (S3 Fig). “robust” means the humerus bone has a larger cross-section. Modified as suggested.

Page 12 Line 9
Where within the cortex are these tissues found? How are they distributed throughout the cortex?

REPLY: We have modified this sentence as “The cortex mainly consists of fibrolamellar bone tissue, but parallel-fibered bone tissue is shown in the posterior cortex”.

Page 12 subadult line 3. “The medullary cavity is large (the diameter is about 5 mm)” comment: but not relative to the rest of the cross section...it may be large compared to the juvenile...but this is relative. It appears quite small to me in the figure.

REPLY: We have modified this sentence to “The medullary cavity is relatively large (the diameter is about 5 mm) compared to those of juvenile individuals”

P13. Line 2. What side of the bone is this on? It's a cool structure so some anatomical context would be appreciated.

REPLY: Added as suggested.

P14 Line 1. Also include mid-cortex and endosteal values. See general notes for more on this.

REPLY: We have added these values in the revised MS.

P14 Fig. 4 Where are D and E from in C? See general notes for further comments on this.

REPLY: we have added a box in C to show the position of D and E.

P14. Line 16. The vascular canals are primarily longitudinal but there are also many irregular canals
comments: Where? Label them in figure.

REPLY: We have added the labels in figure 4C.

P14 Line 18. Label these in C as well so there is context for D and E

REPLY: Added.

P15. Comments: The distinction between growth marks and LAGs should be clearly established in the methods section

REPLY: We have added their differences in the methods section. Growth marks and LAGs both form lines, but growth marks could not be traced on the whole circumference cortex bone.

P16 figure 5. **comment**: Indicate from where in A. See general notes for more details

REPLY: We have added a box in A showing the position of B.

P16 figure 5 wide-open canals.

REPLY: enlarged canals usually referred to secondary remodeling. Here we just describe its shape. It should represent primary bone.

P16 paragraph 3 line 3. Compared to what?

REPLY: We have modified this sentence to make it more clear.” The vascular canals are longitudinally-oriented and relatively small in the outer cortex than in the mid and inner cortices”.

P16 paragraph 3 line 5 Stay consistent in reporting percentages and report for all regions.

REPLY: We have added more percentages of cortical porosity in different parts of the cortex.

P16 paragraph 3 line 6 Also mention the LARGE one to the bottom of Fig. 6A.

REPLY: The large cavity to the bottom is a large erosional cavity surrounded by endosteal bone rather than secondary osteon because its size is much larger than other secondary osteons (See fig. S8). We have added the description of this large cavity.

P17 line 1.
Is this section truly from the mid-diaphysis? It looks to me like it's slightly towards the proximal or distal end which is fine, but do report it if that's the case. If it is in fact truly from the mid-diaphysis, the medullary cavity is much larger than in the other adult

specimen and this difference should be discussed.

REPLY: Thank you for pointing out. The femur of IVPP V26546 that sampled only preserved the distal part. We rechecked this specimen and found that you are correct. The measurement of the preserved region is 5 cm, and the estimated whole length is about 14.6 cm. So this section was sampled near the distal region (See figure S1). We have added this information in the method and description part of the paper.

P19 Line 6.
Cite studies such as Köhler et al. 2012 that show LAGs are deposited even in fast growing animals in stressful enivornmental conditions.

REPLY: Cited as suggested.

P19 line13:
There are two discussion points missing from here I think. First, wouldn't the LAGs in the mid-cortex mean that Chinese *Lystrosaurus* was experiencing some kind of stressful season? Of anything, there is more indicators of stress in specimens from China than those from South Africa. I think if there is an argument to be made about the environment being more stressful in South Africa, you need more clarity on the size differences. If you can show that the South African specimens were smaller than your subadult specimens with LAGs then it would get around this issue. Second, I would move the section where species differences in bone tissue is discussed here and expand on differences that may be related to species-level differences.

REPLY: we have added more comparison on body size between Africa and Chinese *Lystrosaurus*, and pointed out that our individuals are larger than sampled Africa *Lystrosaurus* from Early Triassic. We have also added species differences discussion here, although only a little could be clarified here.

Page 19. Paragraph 2. Line5
Do you mean to say something more like, "an artifact of sampling only young individuals from South Africa"? This is not clearly written as is.

REPLY: Here we mean that *Lystrosaurus* from Early Triassic may die at a young age (indicated by fewer LAGs), so they are smaller than those from late Permian (should be at least subadult based on more LAGs). We have modified this sentence to “but this may the result of the young stage (indicates by rare LAGs) of the body in the Early Triassic based on bone microstructure”.

Page 19. Paragraph 2 line 6.
This formation has not been introduced. Describe it's age here for audience reference.

REPLY: Added as suggested.

Page 20 line 10.
Do you mean in the mid-cortex? This means something else in reference to the gross anatomy so I would avoid using it.

REPLY: We have modified this sentence to "---in the inner and mid cortex"

Page 20. Line 11. This jump is not logically laid out. You state a thick cortex and abundant canals suggests aquatic lifestyle. Then you state the cortical thickness (which I think needs clarification or remeasuring) but do not state whether that is thick or thin compared to other animals. You state that the ribs are different. And then you state that because of this, you can't say it's aquatic/semiaquatic. See general comments for further discussion.

REPLY: we have deleted this sentence and modified this paragraph properly to make it more clear. Most of our samples support *Lystrosaurus* has a thick cortex, as in *Lystrosaurus* of Africa. But thick cortex is not only seen in aquatic animals but also shown in extant burrowing animals. Therefore, thick cortex could not provide confident evidence of the aquatic or semiaquatic lifestyle of *Lystrosaurus*. The lifestyle of *Lystrosaurus* could not be certain right now, but we provided more methods to solve this problem.

PAGE 28
S1 Fig. I did not get this figure in my review document.

REPLY: Sorry. We have unloaded this figure in the revised version.

Figure 1. The guides in B to show were zoomed in photos are from are really helpful and necessary. I would suggest adding similar boxes to the rest of the figures for clarity.

REPLY: We have added boxes in other figures as suggested.

I would add anatomical directions (i.e. medial, lateral, posterior, anterior) in either the figure or the caption (e.g. medial to right, lateral to left, etc.). I would do this for all of the histology figures.

REPLY: Thank you for your suggestions. We have added anatomical directions in all the figures.

Sincerely,
Fenglu Han
