# Supplementary material for: Preliminary bone histological analysis of Lystrosaurus (Therapsida: Dicynodontia) from the Lower Triassic of North China, and its implication for lifestyle and environments after the end-Permian extinction
Source: PLoS One. 2021 Mar 18;16(3):e0248681. doi: 10.1371/journal.pone.0248681 (PMC7971864; doi:10.1371/journal.pone.0248681)
Supplement: Attachment — Submitted filename: PONE-D-20-17684_R2_reviewed.pdf [file pone.0248681.s012.pdf]

PLOS ONE

Bone histology of Lystrosaurus (Therapsida: Dicynodontia) from the Lower Triassic of North China, and its implication for lifestyle and environments after the end-Permian extinction

--Manuscript Draft--

**Manuscript Number:**	PONE-D-20-17684R2
**Article Type:**	Research Article
**Full Title:**	Bone histology of Lystrosaurus (Therapsida: Dicynodontia) from the Lower Triassic of North China, and its implication for lifestyle and environments after the end-Permian extinction
**Short Title:**	Bone histology of Lystrosaurus from North China
**Corresponding Author:**	Fenglu Han, Ph.D<br>China University of Geosciences (Wuhan)<br>Wuhan, CHINA
**Keywords:**	bone histology; Lystrosaurus; Early Triassic; China
**Abstract:**	Lystrosaurus represents one of the most successful dicynodonts, a survivor of the end-Permian mass extinction that remained abundant in the Early Triassic, but many aspects of its paleobiology are still controversial. The bone histology of Lystrosaurus species from South Africa and India has provided important information on their growth strategy and lifestyle, but until recently no data was available on the bone histology of Lystrosaurus from China. Here, we report on the bone microstructure of seven Lystrosaurus individuals from the Lower Triassic of Xinjiang, providing the first such data for the Chinese Lystrosaurus species. Our samples indicate that the microstructure of Lystrosaurus limb bones from China is characterized by fibrolamellar bone tissue similar to those from South Africa and India. Three ontogenetic stages were identified: juvenile, early subadult, and late subadult based on lines of arrested growth (LAGs) and bone tissue changes. Bone histology supports a rapid growth strategy for Lystrosaurus during early ontogeny. Unlike Early Triassic Lystrosaurus from South Africa, lines of arrested growth are common in our specimens, suggesting interruption in growth in Lystrosaurus from China. The differences in bone histology between Lystrosaurus from South Africa and China may indicate different environmental conditions in these two regions.
**Order of Authors:**	Fenglu Han, Ph.D
	Qi Zhao
	Jun Liu
**Response to Reviewers:**	Jan. 6th, 2021<br>Dear Editor and Reviewers:<br><br>Thank you for your letter and for the reviewers' comments concerning our manuscript entitled "Bone histology of Lystrosaurus (Therapsida: Dicynodontia) from the Lower Triassic of North China, and its implication for lifestyle and environments after the end-Permian extinction" (ID: PONE-D-20-17684). Those comments are all valuable and very helpful for revising and improving our paper, as well as important for highlighting the significance of our work. We have studied the comments carefully and made a number of corrections to address them, which we hope to meet with approval. The revised portions of the MS were marked by red colors. The main corrections to the paper and the responses to the editor and reviewer's comments are as following:<br><br>Reviewer #4: I appreciate all of the work put into this revision and find it greatly improved. I believe the paper now clearly demonstrates methodology and provides sufficient details to warrant publication. Particularly, S2 and S3 figs. Are well presented figures! I find it very helpful and thorough. There are only a few minor line edits that I found that only related to grammar and clarity. I have attached these comments in a marked version of the PDF.

Powered by Editorial Manager® and ProduXion Manager® from Aries Systems Corporation

REPLY: Thank you for all your comments and corrections. All the minor comments in the pdf file have been corrected. In addition, the revised version has also been modified a lot based on another reviewer's comments. For example, we have deleted some values of cortical thickness and cortical porosity that were thought to be objective, but we still keep S2 and S3 Figs that can show bone microstructure more clear. We also have added new comparisons between Chinese and South Africa Lystrosaurus based on the new published paper by Botha et al. (2020). We hope all these modifications are comfortable for you.

An additional comment refers to S2 fig. The title of the figure says "accurate". I'm not sure what that is in reference to. But it would be better to have the limbs similarly oriented and the view explicitly stated (e.g. anterior).

REPLY: I think you mean S1 Fig. We just pointed the positions that we sampled in the figure. We have removed the word "accurate" and added limb orientation as suggested.

Reviewer #5: This study describes the bone histology of seven Lystrosaurus individuals from the Lower Triassic of North China. The data have potential to provide interesting and new information on the growth patterns of Lystrosaurus as no studies have yet to focus on this taxon from China, potentially allowing for comparisons with Lystrosaurus from South Africa and Antarctica. However, given that the individuals could not be identified to species level (and thus there could be interspecific variation) I would call this study, at most, a preliminary work. They have very interesting conclusions suggesting that the Lystrosaurus from China are larger than those from the Triassic of South Africa and they lived longer (i.e. there are more LAGs in the Chinese specimens compared to the Triassic species from South Africa). This is the most interesting result of the study. However, I have some concerns relating to how much can be concluded from the data and these concerns should be addressed before the manuscript is ready for publication. My concerns are as follows.

1. Only three out of the seven individuals have more than one LAG – and the third element is a rib, which looks like it has a double LAG, not a triple LAG and certainly not an EFS. Double LAGs indicate one season (laid down in one year), so I would not call what you see in the rib as representing multiple seasons. That leaves the two femora – so only two individuals have three or four LAGs. I'm not convinced this is a large enough sample size to indicate that Chinese Triassic Lystrosaurus were growing for longer periods compared to South African Triassic species. At most this is a preliminary observation and more work needs to be done to see if you can replicate the data.

REPLY: We agree that our work is just a preliminary observation and much work needs to be done in the future, but we do not think the third element only has a double LAG that represents one year. The third element is a rib, which has three LAGs that were identified previously. We agree that only two prominent LAGs are shown in Fig. 6J, and the third outermost LAG is weak. At least, there are two LAGs present. We supposed it to be two LAGs rather than a double LAG, mainly based on the following evidence: these two LAGs are isolated, vary out of sync, and never combined; the related femur has more than 2 LAGs and a wide parallel-fibered bone at the periphery, which was sampled by another researcher and will be published elsewhere so that could not use in this paper, unfortunately. Furthermore, the LAGs are more closed to each other in the thin cortex (such as ribs, fibula, etc) than in the robust cortex (such as humerus, femur and tibia). For example, see the paper Han et al (2020, the bone histology of Jeholosaurus). The distinct between LAGs is much narrower in the fibula than that in the tibia. Finally, this specimen (sampled rib) is the largest individual that we sampled and it is reasonable that it has more LAGs than others.

2. The data used to compare the size of Chinese Lystrosaurus with South African Lystrosaurus is outdated. Sam-pk-k8 and Sam-pk-8991 do not represent fully grown specimens or the largest known specimens. You can look at Botha 2020 (Botha, J. 2020. The paleobiology and paleoecology of South African Lystrosaurus. PeerJ 8:e10408. DOI: doi.org/10.7717/peerj.10408.). Granted, this publication came out while this study was under review and thus the authors have not had the opportunity to compare the data – but now is the time to do so. There is skull data for the maximum

Powered by Editorial Manager® and ProduXion Manager® from Aries Systems Corporation

sizes of each South African species as well as size data for the limb bones used – i.e. the authors compare the size of the humerus, this can be done with both Triassic South African Lystrosaurus species to see how much larger the Chinese species is – if they really are notably larger and this can be confirmed, then it is a very interesting result, but this needs to be more adequately confirmed.

REPLY: Thank you for your new information. We have added more comparison of the known humeral length with a LAG between Chinese Lystrosaurus and South Africa from Early Triassic, we found that their size has no significant difference (S4 Fig). But Chinese Lystrosaurus still has a relatively large proportion with LAGs compared to South Africa Lystrosaurus. The humeral length larger than 10 cm is the most common size based on our observation. Both the body size and bone microstructure support that many Lystrosaurus have reached at least the subadult stage, unlike Triassic Lystrosaurus from Africa where subadults are rare. Of Course, we need more work to clarify this conclusion.

3. I disagree with the last ontogenetic stage being labelled adult. An adult will have a significant amount of slower growing tissues (parallel-fibered or lamellar) and/or an EFS – neither of which is present in the largest individuals. There is the beginning of parallel-fibered bone, so what the authors have captured here is the beginning of slower growth which would make these individuals subadults. It also cannot be confirmed if what is seen in the rib is an overall decreased growth or just parallel-fibered bone associated with a double LAG after which growth may have increased again. The two femora are undoubtedly older than the other individuals, but they are not adult. You could perhaps divide the stages into juvenile, early subadult and late subadult – although even saying late subadult is a push because there is so little parallel-fibered bone.

REPLY: We have changed the word “adult” to “late subadult” stage as suggested.

4. I have a problem with the methodology in calculating the cortical thickness of the bones. The problem with most dicynodonts is that they do not have a sharp transition from a clear open medullary cavity to a compact cortex. There is generally, and this is seen in Lystrosaurus, a gradual transition zone and there are often trabeculae within the medullary cavity. So if you are measuring the compact bone manually as the authors have done here, where do you objectively draw the line between compact bone and medullary cavity? It is very difficult to standardize and objectively measure cortical thickness in this way. This is why the program Bone Profiler is better than a manual procedure because the computer measures so many transects all around the bone. I don’t think any of the cortical thickness measurements are useful if they are done manually (unless you have sharp transition zones, which you don’t in Lystrosaurus). These calculations cannot be compared with other Lystrosaurus specimens or with other taxa. However, using Bone Profiler to calculate cortical thickness (which is generally K in the program) is a lot of work as the cross-sections needs to be changed into black and white images, which can only be done manually by colouring in the bone black and the spaces white – this is extremely time consuming. I thus, suggest that the entire cortical thickness section be deleted. The values cannot be reliably used and they don’t say much about the lifestyle of Lystrosaurus anyway. If the authors wish to keep the cortical thickness in the paper then they need to use Bone Profiler to obtain their values.

REPLY: Thank you for your suggestions. In fact, we tried to use the software bone profiler after your suggestion in your first review. But we found many problems and we gave up finally. First, we did not find a valid link that could download this software. The official link provides a new version that analyses on the website but was also not available for analysis right now. In addition, I do not quite understand the principle of the software. All the bone has the same color, then how to separate compact bone and cancellous bone in the program? Girondot and Laurin (2003, p460, secondary paragraph) mentioned that K was the ratio between the internal and external diameter of the bone, so it should not represent the cortical thickness that we mentioned here.

Finally, we agree that a gradual transition from compact bone to medullary cavity has happened in Lystrosaurus, but not all specimens are difficult to separate. There is still a clear change in some specimens, such as the femur IVPP V26542. This situation is

Powered by Editorial Manager® and ProduXion Manager® from Aries Systems Corporation

also mentioned by Ray et al (2005). So in the revised version, we keep the cortical thickness values that we can discern the transition clearly and have omitted other values with a vague transition between compact bone and medullary region. Furthermore, we have also omitted cortical porosity values with vague transitions.

5. There is also a problem with the methodology for calculating cortical porosity. Fig S3 shows where the fields of view were taken – A, B and C are ok, but in D Field Of View 8, 9 and 10 are taken from the inner regions of the cortex, some of which includes resorption cavities – these cannot be compared with primary vasculature – so 8, 9 and 10 would give you inflated values compared to the others. Even in FOV 1-4 the FOV includes enlarged cavities and not just primary vasculature – this again inflates the values compared to 5-7. FOV is generally taken from the mid-cortex only and all 10 FOVs are taken from the “same” place giving you an average over 10 FOV. One can do what was done in A, B and C because each FOV is the same. E is also a problem because some FOVs are from the outer cortex, some from the middle and some from the inner regions – so you’re not standardizing where you’re taken the FOV from. Yes, you’re getting an average of the bone, but you’re doing it in a different way each time. F and particularly G I’m worried about as the inner regions of the FOV are resorption cavities which you cannot include in primary vasculature – so here, the entire bone’s values are inflated, same with H – in fact in H, there is even medullary cavity, so was this included as space or was it ignored? If the latter then your FOV is smaller depending on where the FOV was taken, so you’re not comparing same-sized FOVs. This is going to produce unreliable results.

REPLY: We agree that there are still some problems with measuring cortical porosity. The best way is to measure the whole compact bone. However, there are at least two problems: As we mentioned above, there is no sharp transition between compact bone and medullary cavity in some specimens, and the areas that we sampled may be objective. Therefore, in the revised version, we only keep the cortical porosity values with clear tissue change. Secondly, some bone sections do not show clear bone tissue in some parts due to bad preservation, such as Fig S3 E. It has a clear medullary cavity, but not allowed us to standard the acute position. It just provides a reference, that is why we mentioned that it was an estimated value in the method. Nevertheless, we have omitted this value according to the reviewer’s comments.

6. It would also be good to include Whitney, M.R. and Sidor, C.A. 2020. Evidence of torpor in the tusks of Lystrosaurus from the Early Triassic of Antarctica. Communications Biology 3:471. DOI: 10.1038/s42003-020-01207-6. Although they do not deal with limb bones (they use teeth) they do talk about different environments affecting Lystrosaurus. This publication may have come out while this study was still in review – but it would be good to include it now for a discussion on differing environments affecting Lystrosaurus growth.

REPLY: We have added this reference and mentioned that environments can affect tusk and bone microstructure of Lystrosaurus. But we did not discuss a lot because it was difficult for comparison between teeth and bone microstructure.

Comments in the pdf

P5, line 10
Comment: you need to explain the difference between a LAG and an annulus. i.e. a LAG is a cement line and indicates a temporary, but complete cessation in growth whereas an annulus consists of lamellar or parallel-fibered bone and indicates a temporary decrease in growth rate

REPLY: We have added this information.

Table 1. Comment: fix this column so that the s is not on another line

REPLY: Modified as suggested.

P6, line 2
why was this specimen chosen? is it the largest known in China? is it the most complete? does it have a skull? can you identify it to species level? You say that the

ontogenetic stage is unknown - but is it small or big? are sutures fused? are their muscle scars? etc you must be able to estimate the ontogenetic stage of this specimen. Are you estimating all these other values based on a juvnenile or an adult?

REPLY: we have added more information on this specimen in the method. It is the holotype of L. hedini and represents the most complete specimen of Lystrosaurus (Young 1935). It has a skull length of 173 mm and includes most of the postcranial material. It is probably a subadult individual based on open neurocentral sutures and fused sacral vertebrae. It has a humeral length of 11.5 cm, which is more suitable to assign it to be within the subadult stage compared to others.

P6, paragraph 1
Comment: This is an outdated method for calculating cortical thickness and is problematic in an animal such as Lystrosaurus because there is no clear strict transition between cortical bone and medullary cavity. The broad transition zone in this region makes measurements calculating cortical thickness quite subjective. I recommend using Bone Profiler for this method. These measurements will then be more useful in that they can be compared with other studies.

REPLY: We have already given a detailed explanation above. We have omitted controversial data but keep some measurements that have a clear transition. As we provided the accurate position that we measured (Fig. S2). The reader can choose whether to use it or not.

P6, paragraph 1, Line 11
This method differs from that of previous studies - which take 10 fields of view from the mid-cortex all around the cross-section. It is unclear if you are doing a transect from the inner to outer cortex and getting the average, or if you are taking inner versus outer fields of view randomly all over the cross-section - if the latter then this method is not being very consistent. You need to standardize this method more.

REPLY: We have given a detailed response above. In addition, we are doing an average cortical porosity from the inner to outer cortex rather than the mid cortex. As we know that cortical porosity is different from the inner to outer cortex, so it also has a problem to just choose mid-cortex, because cortical porosity is gradually changed from the inner to outer cortex. How do we define the mid cortex is a problem and objective.

P7, paragraph 2.
Comment: There is a new reference which will help you compare your results to the SA Lystrosaurus, you need to include this:
Botha, J. 2020. The paleobiology and paleoecology of South African Lystrosaurus. PeerJ 8:e10408. DOI: doi.org/10.7717/peerj.10408.

REPLY: Thank you for your information. We have added this reference and more comparison in the discussion part.

P7, table 2
Comment: separate into two separate columns

REPLY: modified as suggested.

P7, table 2
Comment: so would these values mark the highest being the inner cortex and the lowest value being the outer cortex?

REPLY: Sorry for the misunderstanding. Providing the variation of cortical thickness was suggested by Megan Whitney. It is the ratio of compact bone (one side) to the diameter of the whole cross-section. For example, see Fig S2E. The dorsal cortex is thin and the ventral cortex is relatively thick, so the cortical thickness ranges from 16-41%. Please see the method part for detailed information.

P8, table 2
extensive secondary reconstruction would imply dense Haversian bone, or that the

reconstruction has reached the outer cortex - is this the case for your elements here? It doesn't look like it from the figures

REPLY: Thank you for pointing it out. We have modified this sentence as “a few secondary osteons”

P8, Juvenile, line 5.
Comment: where would you denote the edge of the medullary cavity here with such a gradual transition zone? You need to explain how you got these measurements and how you ensured that they were not subjective, but standardized

REPLY: We have added more explanation on how to identify compact bone and medullary cavity. The compact bone tissues are connected tightly without bone trabecula and large resorbed cavity, whereas the medullary cavity either empty (we call it free medullary cavity) or filled with trabecula, which is usually isolated or loosely connected. However, sometimes, the trabecula is abundant and show a similar connection as in compact bone, and could not be separated clearly.

P9 line 2. Comment: in circumferential rows in parts?

REPLY: Yes, added.

P9, line 11.
not shown in Fig 1B - label or take out in the text here

REPLY: The word “Fig. 1B” has been taken out here.

P9, figure 1. I know what you mean here by wide-open canals - but this isn't a very good term. Just label them large primary canals because that's what they are.

REPLY: Thank you for your suggestions. Modified.

P9, last line.
Comment: then how did you calculate cortical thickness?
P10 line 8. Comment: then how accurate is the cortical thickness measurement?

REPLY: We have provided more explanation on defining medullary cavity and compact bone in the method, See our explanation above.

P10, Fig 2 caption N.
Comment: I'm not convinced those are secondary osteons - I cannot see a cement line or How ship's lacunae - you need a better close up of this

REPLY: We have changed to a close up new picture to show secondary osteon here.

P11 paragraph 2 line 11.
Comment: according to your definition then - these growth marks cannot be seen around the whole circumference of the bone? So are they really indicative of a change in growth rate?

REPLY: These growth marks usually associated with parallel-fibered bone in the outermost region, so we consider it to indicate a slow growth rate.

P12 Subadult. Line 11.
Comment: This is not shown from Fig 3A and B - I would not call those channels "significantly reduced"

REPLY: we have removed the word “significantly”.

P12 Subadult. Line 12. can this LAG be followed around the whole circumference?

REPLY: This LAG is weak and not clear in some parts under normal light, and it is also not clear under polarized light, so we revised it to be a growth mark in the revised MS. Therefore, we remove this individual to be within a juvenile stage.

Powered by Editorial Manager® and ProduXion Manager® from Aries Systems Corporation

P13. Line 1. cannot see the LAG here
REPLY: We have removed the word “a LAG” here.

P13 paragraph 2 Line 13
It looks like a double LAG - don't say several otherwise it sounds like an EFS.

REPLY: We did not say several LAGs. There is only one prominent LAG and we wrote “a distinct LAG comprised of several growth marks”. It does show more than two growth marks (weak growth lines) but not LAGs. We remarked the arrow positions to show growth marks that more clear.

P13 Figure 4 caption. Line 3.
Comment: I'm not convinced - looks like two not three. And does the multiple number stay consistent around the whole cortex or does it become one LAG at some point?

REPLY: Again, we did not say it has three LAGs, instead, it is just one LAG. But we can see that it contains several growth marks. It becomes narrower but still a distinct LAG in other regions.

P13 Figure 4 caption. Line 5.
Comment: this looks like a single LAG here

REPLY: Yes. We agree. It is a single LAG containing several growth marks (or weak growth lines).

P14 line 1. Comment: looks like a double LAG here too

REPLY: Yes. We agree and have modified in the revised MS.
P14 line 1. this isn't a normal term - they are either resorption canals or canals that are just starting to form - look to see which one

REPLY: Yes. Here we only describe its shape that was suggested by another reviewer. They probably represent resorption canals.

P14 paragraph 2 line 6
Comment: so what are these? how far do they go around the cortex? do they run with the LAG or are they separate?

REPLY: Here we mean that a clear LAG was formed by several growth marks (weak growth lines that vary throughout the whole cortex), but we rethink parallel-fibered bone is more suitable. We have modified this sentence as “A clear LAG, as well as parallel-fibered bone, are present at the mid-region as in the fibula”.

P14 paragraph 2 line 7
Comment: cannot still be abundant but also strongly reduced - rephrase - I wouldn't call the canals strongly reduced. Perhaps there is a slight decrease in vascular canal size towards the periphery

REPLY: We have changed the word “strongly” to “gradually”.

P14 adult stage.
Comment: how do you define adult tissues?

REPLY: Adult stage was usually defined by the presence of EFS or lamellar/parallel-fibered bone tissue at the periphery. Here, we found parallel-fibered bone at the periphery. However, we agree that the layer of parallel fibered bone was still narrow. We have changed this stage to “late subadult” as suggested.

P15 Line 1
Comment: as with my comments above for every element - how then do you decide where the edge of the compact cortex is?

Powered by Editorial Manager® and ProduXion Manager® from Aries Systems Corporation

REPLY: cortical thickness and porosity have been removed in this specimen.

P15 paragraph 1 last line.
Comment: you've added in Placerias - but an EFS has been found in Kannemeyeria as well - see Botha-Brink and Angielczyk 2010 - those are the only two so you may as well name them seen as they are dicynodonts

REPLY: We have added this reference.

P15 Fig 5 caption.
Comment: I am not convinced this is an adult - at most, with such a small region of parallel-fibered bone at the periphery we are seeing a first transition to slower forming bone tissues - so at most this is a subadult. An adult would have a much larger region or parallel-fibered bone or lamellar bone or an EFS

REPLY: We have changed the stage “adult” to “late subadult”.

P15 Last line.
Comment: change this term
REPLY: Modified.

P16 Fig. 6. Comment: looks like a double LAG not triple
REPLY: This should be at least two LAGs rather than a double LAG. These two LAGs have more than three LAGs and a wide parallel fibered bone have been founded in the associated femur. But it has already been sampled by another research and will be published elsewhere.

P17. Line 5 I would say two
REPLY: Modified as suggested.

P17. Line 5.
Comment: no - not enough to indicate an EFS - looks like a double LAG. This is not an EFS - as you decide it's not one from your next comment - rather just take out the phrase "similar to an EFS"
REPLY: We have deleted this sentence.

P17 Discussion. Line 4.
Comment: There is not enough evidence to indicate this third stage - the rib cannot be used for this and the two femora look like subadults
REPLY: We have modified the “adult” to “late subadult” and modified the text to make it more clear.

P17 Discussion. Line 6.
no - you don't know that - no EFS - a double LAG is not enough to indicate general slow growth - definite subadult, but I wouldn't call these femora and rib adults

REPLY: We have changed the “adult” to “late subadult” stage.

P17 Discussion. Line 6.
Comment: what about the humerus?

REPLY: We have added the word“the humerus”.

P18 Paragraph 3 line 4.
Comment: only three out of seven have more than 1 LAG - and the rib looks like a double LAG - i.e. indicating one season, the rib is not a good one to include in stating that more than 1 or 2 LAGs are present. That just leaves the two femora that have 3 or 4 LAGs - this is not a high sample size

REPLY: As we mentioned above, the rib in Figure 6 has at least two LAGs, and this individual also sampled the femur which has more than 2 LAGs and wide parallel-

	fibered bone tissue at the periphery. But it has been sampled by another researcher and will be published elsewhere.<br><br>P18 Paragraph 3 line 8<br>these are not the largest Triassic Lystrosaurus - compare your elements with Botha 2020<br>P18 Paragraph 3 line 9<br>confirm this by comparing with the largest Triassic humeri in Botha 2020<br><br>REPLY: We have added a new comparison and S4 Figure according to this new reference, and we found that there is no significant size difference between Chinese and South Africa Lystrosaurus, but Chinese Lystrosaurus tends to have more LAGs, which may be affected by different species or environment. This did not affect our conclusion that Chinese Lystrosaurus have reached at least the subadult stage based on the presence of LAGs and relatively large body size (see revised MS for detailed information).<br><br>P19. Line 3.<br>Comment: you don't show this in the table - in the table it's just the two femora that have more than two LAGs. There is only one juvenile humerus in your tables<br><br>REPLY: We have modified this sentence as “All the other individuals with humeral length larger than 12 cm (including estimated value) have more than two LAGs to be among late subadult stage (Table 1)”. The two femora IVPP V26546 and IVPP V26547 have more than two LAGs. IVPP V26546 also preserves a humerus (12 cm), although we did not sample it. IVPP V26547 and IVPP V26548 do not preserve the humeri, but the estimated humeral length is larger than 12 cm. We have changed the “Elements” to “Sampled elements” to make it more clear.<br><br>P20 Line 1<br>Comment: yes but this has been shown to be false in Botha 2020 - the size reduction is just because they didn't live long enough to attain large sizes, not because of miniaturization.<br><br>REPLY: We have added this new reference.<br><br>P20 paragraph 2 line 7<br>Comment: cannot start a sentence with and<br><br>REPLY: Modified.<br><br>P20 paragraph 2, line 8<br>Comment: not necessarily - it depends on where you sampled your rib. Cortical thickness in ribs differ along the length of the shaft and you haven't said where your sample was taken from - so it's very difficult to compare ribs unless you know for sure they were sampled at the same position along the shaft<br><br>REPLY: We have deleted this sentence.<br><br>Sincerely,<br>Fenglu Han
**Additional Information:**	
**Question**	**Response**
**Financial Disclosure**<br><br>Enter a financial disclosure statement that describes the sources of funding for the work included in this submission. Review the submission guidelines for detailed requirements. View published research articles from *PLOS ONE* for specific	This study was supported by Strategic Priority Research Program of Chinese Academy of Sciences (XDB26000000) to JL, the National Natural Science Foundation of China (www.nsfc.gov.cn; 41530104, 41661134047) to FH and JL respectively. The funders had no role in study design, data collection and analysis, decision to publish, or preparation of the manuscript.

<table>
<tr><td>examples.

This statement is required for submission and will appear in the published article if the submission is accepted. Please make sure it is accurate.

Unfunded studies
Enter: The author(s) received no specific funding for this work.

Funded studies
Enter a statement with the following details:
• Initials of the authors who received each award
• Grant numbers awarded to each author
• The full name of each funder
• URL of each funder website
• Did the sponsors or funders play any role in the study design, data collection and analysis, decision to publish, or preparation of the manuscript?
• NO - Include this sentence at the end of your statement: The funders had no role in study design, data collection and analysis, decision to publish, or preparation of the manuscript.
• YES - Specify the role(s) played.

* typeset</td><td></td></tr>
<tr><td>Competing Interests

Use the instructions below to enter a competing interest statement for this submission. On behalf of all authors, disclose any competing interests that could be perceived to bias this work—acknowledging all financial support and any other relevant financial or non-financial competing interests.

This statement will appear in the published article if the submission is accepted. Please make sure it is accurate. View published research articles from PLOS ONE for specific examples.</td><td>The authors have declared that no competing interests exist.</td></tr>
</table>

**NO authors have competing interests**<br><br>Enter: *The authors have declared that no competing interests exist.*<br><br>**Authors with competing interests**<br><br>Enter competing interest details beginning with this statement:<br><br>*I have read the journal's policy and the authors of this manuscript have the following competing interests: [insert competing interests here]*<br><br>* typeset	
**Ethics Statement**<br><br>Enter an ethics statement for this submission. This statement is required if the study involved:<br><br>• Human participants<br>• Human specimens or tissue<br>• Vertebrate animals or cephalopods<br>• Vertebrate embryos or tissues<br>• Field research<br><br>Write "N/A" if the submission does not require an ethics statement.<br><br>General guidance is provided below. Consult the submission guidelines for detailed instructions. **Make sure that all information entered here is included in the Methods section of the manuscript.**	N/A

Powered by Editorial Manager® and ProduXion Manager® from Aries Systems Corporation

**Format for specific study types**<br><br>**Human Subject Research (involving human participants and/or tissue)**<br>• Give the name of the institutional review board or ethics committee that approved the study<br>• Include the approval number and/or a statement indicating approval of this research<br>• Indicate the form of consent obtained (written/oral) or the reason that consent was not obtained (e.g. the data were analyzed anonymously)<br><br>**Animal Research (involving vertebrate animals, embryos or tissues)**<br>• Provide the name of the Institutional Animal Care and Use Committee (IACUC) or other relevant ethics board that reviewed the study protocol, and indicate whether they approved this research or granted a formal waiver of ethical approval<br>• Include an approval number if one was obtained<br>• If the study involved *non-human primates*, add *additional details* about animal welfare and steps taken to ameliorate suffering<br>• If anesthesia, euthanasia, or any kind of animal sacrifice is part of the study, include briefly which substances and/or methods were applied<br><br>**Field Research**<br><br>Include the following details if this study involves the collection of plant, animal, or other materials from a natural setting:<br>• Field permit number<br>• Name of the institution or relevant body that granted permission	
**Data Availability**<br><br>Authors are required to make all data underlying the findings described fully available, without restriction, and from the time of publication. PLOS allows rare exceptions to address legal and ethical concerns. See the PLOS Data Policy and FAQ for detailed information.	Yes - all data are fully available without restriction

Powered by Editorial Manager® and ProduXion Manager® from Aries Systems Corporation

A Data Availability Statement describing where the data can be found is required at submission. Your answers to this question constitute the Data Availability Statement and **will be published in the article**, if accepted.<br><br>**Important:** Stating 'data available on request from the author' is not sufficient. If your data are only available upon request, select 'No' for the first question and explain your exceptional situation in the text box.<br><br>Do the authors confirm that all data underlying the findings described in their manuscript are fully available without restriction?	
**Describe where the data may be found in full sentences. If you are copying our sample text, replace any instances of XXX with the appropriate details.**<br><br>• If the data are **held or will be held in a public repository**, include URLs, accession numbers or DOIs. If this information will only be available after acceptance, indicate this by ticking the box below. For example: *All XXX files are available from the XXX database (accession number(s) XXX, XXX.*).<br>• If the data are all contained **within the manuscript and/or Supporting Information files**, enter the following: *All relevant data are within the manuscript and its Supporting Information files.*<br>• If neither of these applies but you are able to provide **details of access elsewhere**, with or without limitations, please do so. For example:<br><br>*Data cannot be shared publicly because of [XXX]. Data are available from the XXX Institutional Data Access / Ethics Committee (contact via XXX) for researchers who meet the criteria for access to confidential data.*<br><br>*The data underlying the results presented in the study are available from (include the name of the third party*	All relevant data are within the manuscript and its Supporting Information files.

and contact information or URL).<br>• This text is appropriate if the data are owned by a third party and authors do not have permission to share the data.<br><br>* typeset	
Additional data availability information:	

Manuscript

Click here to access/download;Manuscript;Han et al manuscript R2.docx

Bone histology of *Lystrosaurus* (Therapsida: Dicynodontia) from the Lower Triassic of North China, and its implication for lifestyle and environments after the end-Permian extinction

Fenglu Han[1*], Qi Zhao[2], Jun Liu[2,3, 4*]

[1] School of Earth Sciences, China University of Geosciences (Wuhan), Wuhan, China

[2] Key Laboratory of Vertebrate Evolution and Human Origins of Chinese Academy of Sciences, Institute of Vertebrate Paleontology and Paleoanthropology, Chinese Academy of Sciences, Beijing, China

3 CAS Center for Excellence in Life and Paleoenvironment, Beijing 100044, China;

4 College of Earth and Planetary Sciences, University of Chinese Academy of Sciences Beijing 100049

* Corresponding authors

Email: hanfl@cug.edu.cn (FH); liujun@ivpp.ac.cn (JL)

Abstract

Lystrosaurus represents one of the most successful dicynodonts, a survivor of the end-Permian mass extinction that remained abundant in the Early Triassic, but many aspects of its paleobiology are still controversial. The bone histology of *Lystrosaurus* species from South Africa and India has provided important information on their growth strategy and lifestyle, but until recently no data was available on the bone histology of *Lystrosaurus* from China. Here, we report on the bone microstructure of seven *Lystrosaurus* individuals from the Lower Triassic of Xinjiang, providing the first such data for the Chinese *Lystrosaurus* species. Our samples indicate that the microstructure of *Lystrosaurus* limb bones from China is characterized by fibrolamellar bone tissue similar to those from South Africa and India. Three ontogenetic stages were identified: juvenile, early subadult, and late subadult based on lines of arrested growth (LAGs) and bone tissue changes. Bone histology supports a rapid growth strategy for *Lystrosaurus* during early ontogeny. Unlike Early Triassic *Lystrosaurus* from South Africa, lines of arrested growth are common in our specimens, suggesting interruption in growth in *Lystrosaurus* from China. The differences in bone histology between *Lystrosaurus* from South Africa and China may indicate different environmental conditions in these two regions.

Introduction

The extinction at the end of the Permian was the most influential event in animal history since the Cambrian explosion. About 80% of all marine species and 50-70% of terrestrial families went extinct [1-3]. Recent studies suggest that the Permian-Triassic transition occurred in an increasingly hot environment which was disruptive to life [4-8], although it is still controversial

whether the environment was arid [6, 7] or humid [9]. However, some organisms evolved new survival strategies, such as miniaturization or rapid propagation [10-12].

Lystrosaurus is a medium to large-sized dicynodont therapsid with a strongly anteroventrally downturned snout. It represents one of the most successful vertebrates that survived the end-Permian mass extinction, showing a cosmopolitan distribution during the Early Triassic [13-17]. However, the survival strategies of *Lystrosaurus* during the extinction are still controversial [18, 19]. The lifestyle of *Lystrosaurus* was proposed as being aquatic or semi-aquatic, which could have helped it to escape the harsh terrestrial environment [20, 21], and was supported by histological studies [22, 2[illegible]here is also evidence that *Lystrosaurus* was a burrow maker [24, 25], and burrowing can help an organism survive harsh environmental conditions, as temperature and humidity are generally more stable underground than on the surface [26, 27]. Also, underground burrows can provide better protection for the young, which helps propagate the species [28].

Interestingly, the body size of *Lystrosaurus* from South Africa is smaller in the Early Triassic than in the late Permian [18], suggesting miniaturization. Furthermore, LAGs (lines of arrested growth) are more common in Permian *Lystrosaurus*, which was interpreted as evidence that the Permian species had a more extended ontogeny extended over several years [19]. This contrasts with the prevalence of small individuals with few or no LAGs in the Triassic species, which was part of the evidence suggesting early breeding to compensate for dying at an early age [19].

Bone histology can provide important information on the life history of *Lystrosaurus*, which may bear on its survival during the end-Permian mass extinction. Previous studies have focused on the *Lystrosaurus* record from South Africa and India [22, 23, 29, 30]. However, *Lystrosaurus* seems to have been cosmopolitan in distribution [14], and is also very abundant in the

Juggar and Turpan basins of Xinjiang, China. In China, seven *Lystrosaurus* species have been named since 1935, but the validity of most of these needs re-evaluation [31, 32], and it is possible that only two species (*L. hedini* and *L. youngi*) are valid [33]. Here, we present data on the bone histology of *Lystrosaurus* from the Lower Triassic Turpan Basin, Xinjiang Province, China for the first time, using this data to comment on the survival strategy of this taxon.

Material and methods

Seven specimens of *Lystrosaurus* from the collections of the Institute of Vertebrate Paleontology and Paleoanthropology (IVPP) were selected for histological analysis. All materials come from the Lower Triassic Jiucaiyuan Formation of the Taodonggou area (Taoshuyuan), Turpan Basin, Xinjiang, China [34, 35]. The age of the Jiucaiyuan Formation in this area is middle-upper Induan based on previous chronostratigraphy [34, 36]. Identification of these specimens to species level is currently not possible because the Chinese *Lystrosaurus* taxonomy is currently based on skull characters.

Sections were sampled from the mid-diaphysis of limb bones including the tibia, femur, humerus, ulna, and radius (S1 Fig). However, IVPP V26546 was sampled near the distal end (the distance between the sampled position and the distal end is about 5 cm) of the femur due to the lack of a mid-region. Limb diaphyseal sections were chosen because they are the primary centers of ossification and should preserve the most complete record of growth, and also minimize the potential of secondary remodeling overwriting the growth record (S1 Fig; Table 1) [37, 38]. Rib sections in some individuals were also sampled for comparison. All these materials were photographed and measured before sectioning (S1 Fig; Table 1). However, the sampled specimens

preserved different parts of limb bones making the comparison of body size difficult. Therefore, we estimated values of missing limb bones based on comparing with a complete skeleton of *Lystrosaurus hedini* (IVPP RV35012) [39]. It has a skull length of 173 mm and includes most of the postcranial material. It is probably a subadult individual based on open neurocentral sutures and fused sacral vertebrae. Comparison of the limb bone length (Table 1) with other individuals also ascribes it to be within a subadult stage.

The preparation of the histological sections was carried out at the IVPP. The selected bones were processed using the Exakt-Cutting-Grinding System [40]. Sections were embedded in polyester resin, cut by EXAKT-300CP, and were ground to a thickness of about 50 μm with an EXAKT 400CS grinding machine. The polished sections were then observed under transmitted and polarized light. The thin sections were observed using a Leica DM2700P microscope and photographed using a Leica DMC4500 camera. The whole cross-sections are large and bone microstructure varies in different areas around the cortex, and we have labeled all the sampled directions for all the figures for easy comparison. Nomenclature and definitions of structures follow Francillon-Viellot and Chinsamy-Turan [41, 42]. ‘LAGs’ are growth mark lines that can be traced around the full circumference. A LAG is a cement line and indicates a temporary but complete cessation in growth whereas the annuli correspond to periods of slow growth by the presence of parallel-fibered bone and flattened osteons [41].

Table 1 Skeletal elements of the *Lystrosaurus* specimens examined for histological sampling from the Early Triassic of Xinjiang, China.

Specimen number	Sampled elements	Humeral length (cm)	Ulna length	Radial length (cm)	Femoral length (cm)	Tibial length (cm)	Length of fibulae (cm)	Growth stages

IVPP V26543	Tibia	9.9[e]	8.3[e]	7.4[e]	12.5	9.32	9.0	Juvenile
IVPP V26544	Humerus, Tibia, Fibula	10.1	7.8[e]	7.0[e]	11.8	9.17	9.0	Juvenile
IVPP V26542	Femur	10.3	8.3[e]	6.52	12.5[e]	9.8[e]	9.5[e]	Juvenile
IVPP V26545	Fibula, Radius	15.0	11.0	10.8[e]	18[e]	14.2[e]	13.8[e]	Early subadult
IVPP V26546	Femur	12.0	9.7[e]	8.7[e]	14.6[e]	12.5	11.5	Late subadult
IVPP V26547	Femur	12.4 [e]	10[e]	9[e]	15.1	12.1	11.3	Late subadult
IVPP V26548	Rib	15.6 [e]	12.6[e]	11.3[e]	19.0	14.0	13.5	Late subadult
IVPP RV35012	None	11.5	9.3	8.3	14.0	10.9	10.6	subadult

[e] denotes estimated values based on the comparison of the ratios between elements of the *Lystrosaurus* IVPP RV35012. The estimated values in each element are all compared to the femur, whereas there is no femur preserved, the estimated values are compared to the humerus. "Growth stages" of all individuals except IVPP RV35012 were identified based on bone microstructure.

The cortical thickness was calculated as the ratio of the cortex (one side) to the cross-sectional diameter of the bone [22]. The cortex can be identified by bone tissue connected tightly forming net and has no bone trabecula and large resorbed cavity, whereas the medullary cavity either empty (free medullary cavity) or filled with trabeculae, which are usually isolated or loosely connected. However, sometimes, the trabeculae are abundant and show a similar connection as in compact bone, and could not be separated clearly in this situation, and then the cortical thickness could not be provided. In addition, cortical thickness usually varies in different parts of the whole cross-section, and we have provided a range of cortical thickness measurements in each section

(Table 2). The measurement positions of the maximum and minimum of the cortical thickness in available sections are shown in S2 Fig. Cortical porosity was measured using the software ImageJ 1.52a (https://imagej.nih.gov/ij/). It was difficult to obtain the cortical porosity of a whole cross-section for each specimen because of partial breakage or poor preservation in some areas of these sections. Therefore, we subsampled ten areas from the inner to the outermost cortex in all sections to calculate the average vascular porosity. The sampled areas were chosen from the inner region to the periphery on different parts of the whole cross-sections (S3 Fig), so they could reflect the value of cortical porosity. However, some bone sections show unclear bone histology in some regions, which may affect the results. Cortical porosities of those bone sections that were poorly preserved or with an unclear transition between compact bone and medullary cavity are not provided. High-resolution images were acquired using a Leica DMC4500 camera. All the images were converted to 8-bit files, and the brightness/contrast was adjusted to clarify the structure. After that, we used "threshold" tools in ImageJ to select all the vascular canals. Finally, the cortical porosity was determined using "analyze particles" tools in ImageJ.

Results

The bone histology of *Lystrosaurus* from North China shows a predominance of fibrolamellar bone tissue and a high cortical porosity (Figs 1-6), as in *Lystrosaurus* from South Africa and India [22, 43]. The primary osteons are predominantly longitudinal, forming laminar or plexiform bone. Radially oriented primary osteons are also present in some parts of the cortex. Secondary remodeling is common in the inner and mid-cortical bone in the subadult stage, resulting in enlarged channels and thin periosteal bone. The medullary cavity is small and filled with bony

trabeculae. LAGs are common in the outer cortex of larger individuals.

All these elements could be divided into juvenile, early subadult and late subadult stages based on bone tissue type and the presence of LAGs (Table 2). They are generally similar to the growth stages of *Lystrosaurus* from South Africa and India, but also have some features that differ from the latter.

Table 2. Bone histological information of *Lystrosaurus* from China in each specimen

Specimen number	Skeletal elements	Cortical thickness (%)	Cortical porosity (%)	Bone tissue type	Secondary reconstruction	Growth rings	Medullary cavity
IVPP V26543	Tibia	24-32	29	plexiform and reticular	inner enlarged channels	0	a small free cavity
IVPP V26544	Humerus	21-45	32	plexiform	a few secondary osteons	0	a small free cavity
IVPP V26544	Tibia	19-30	27	laminar and reticular	a few secondary osteons	0	full of trabeculae
IVPP V26544	Fibula	/	/	laminar	a few secondary osteons	0	a large free cavity
IVPP V26542	Femur	16-41	/	laminar and plexiform	a few secondary osteons	0	a free medullary cavity
IVPP V26545	Fibula	/	/	laminar	a few secondary osteons	1	full of trabeculae
IVPP V26545	Radius	/	/	laminar	a few secondary osteons	1	full of trabeculae
IVPP V26546	femur	/	/	laminar	extensive secondary reconstruction	3	full of trabeculae

IVPP V26547	Femur	/	/	laminar	extensive secondary reconstruction	4	full of trabeculae
IVPP V26548	rib	/	/	laminar	extensive secondary reconstruction	>2	full of trabeculae

[e] denotes estimated values.

Juvenile

The juvenile stage was represented by three samples: IVPP V 26543, VPP V 26544 and IVPP V26542. All the cortices are composed of fibrolamellar tissue, with a high number of vascular canals, abundant globular osteocyte lacunae, and no LAGs, suggesting a rapid, constant growth rate. The mid-diaphyseal tibia of IVPP V 26543 is elliptical in cross-section, with a diameter of 13.5 (ML, mediolateral width)×12.0 (AP, anteroposterior width) mm and a cortical thickness ranging from 24-32% (Fig 1; Table 2). The free medullary cavity is small, with a diameter of about 4 mm. The entire cortex is comprised of fibrolamellar bone and dense vascular canals (Fig 1). The cortex has a high cortical porosity (about 29%, Table 2). The canals are predominantly longitudinal and arrange in circumferential rows in parts. A few irregularly shaped canals are shown in the inner and mid cortex (Fig 1B). Radial anastomoses are shown in the medial and anterolateral regions of the cortex, and the medial region has a concave periosteal surface (Fig 1A, C). The anastomoses join two or three adjacent canals in the outer cortex (Fig 1D) and link four to five canals forming long canals in the mid and inner cortex (Fig 1B). Most of the vascular channels show thin centripetal osteonal deposits forming primary osteons (Fig 1E, F). The primary osteons mainly show laminar and plexiform patterns in the outer and deeper cortex, and a row of large primary canals are shown near the periphery (periosteal surface) in some areas (Fig 1D). Osteocyte density is consistently high

throughout the cortex. Erosionally enlarged resorption cavities are present in the inner cortex (Fig 1C). There are no secondary osteons, LAGs, or endosteal bone. The medullary region contains cancellous bone.

Fig 1. Bone microstructure in juvenile *Lystrosaurus* IVPP V26543. A, whole cross-section of the tibia; **B**, partial cross-section showing fibrolamellar bone; **C**, partial cross-section showing radial vascular canals; **D**, the outer cortex under normal light; **E**, enlargement of the outer cortex showing primary osteons under normal light; **F**, bone tissue of E under polarized light. **Abbreviation**: **ec**, enlarged resorption cavity; **rc**, radial canals; **lpc**, large primary canals

In IVPP V26544, the tibia, humerus, and fibula were all sectioned (Fig 2). The cross-section of the tibia is elliptical in outline, with a diameter of 12.5 (ML) ×10.0 (AP) mm (Fig 2A). The cortical thickness ranges from 19% to 30% (Table 2). There is no free medullary cavity. The cortex mainly consists of fibrolamellar bone tissues (Fig 2B), but parallel-fibered bone tissue is present in the medial region of the cortex that articulates with the fibula (Fig 2C). Vascular canals are predominantly longitudinal. There are a few anastomoses that link two to five canals forming long straight and irregular canals. Most of the vascular canals are primary osteons forming a laminar pattern (Fig 2B). A thin layer (1 mm) of secondary osteons is present in the inner region of the compact bone (Fig 2D). The cortex has a high porosity of 27% (Fig 2A, Table 2), but the diameter of vascular canals is slightly reduced near the periphery. Enlarged vascular canals are present in the inner cortex due to secondary remodeling. No LAG/annulus was detected in the cortex. The medullary region is full of trabeculae.

Fig 2. Bone microstructure in juvenile *Lystrosaurus* IVPP V26544. (A-D) tibia. **A**, mid-diaphyseal cross-section of the tibia. **B**, bone microstructure of the tibia; **C**, partial cross-section of the tibia showing the parallel-fibered bone under polarized light; **D**, enlargement of the inner region of B under crossed plane-polarized light showing secondary osteons; (E-J) humerus. **E**, bone microstructure of the humerus under normal light; **F**, bone microstructure of the humerus under polarized light; **G**, the outermost cortex under normal light showing a growth mark (arro[illegible] **H**, the same region as G under polarized light showing parallel-fibered bone; **I**, the inner cortex showing secondary osteons under normal light; **J**, the same region as H under polarized light; (K-O) fibula. **K**, bone microstructure of the fibula under normal light; **L**, bone microstructure of the fibula under polarized light showing parallel-fibered bone; **M**, enlargement of L showing the organized osteocyte lacunae; **N**, the outermost cortex showing secondary osteons under normal light; **O**, the innermost region showing endosteal bone under polarized light. **Abbreviation**: **end**, endosteal bone; **so**, secondary osteons.

The humerus is subtriangular in cross-section with a midshaft diameter of 18.5 (AP) ×15.5 (DV, dorsoventral width) mm and has a greater cortical thickness than the tibia (Fig 2E, F and S3 Fig). It has a thick compact bone (at least 7 mm) with a cortical thickness ranging from 21% to 45% (Table 2), and the free medullary cavity is extremely small. The cortex is comprised of fibrolamellar bone tissue, but a thin layer of parallel-fibered bone tissue is present near the periphery (Fig 2F-H). The bone porosity has a value of about 32% (Table 2), which is higher than the tibia. The primary osteons are predominantly plexiform and are longitudinally-oriented in the outer cortex. They are

strongly reduced in size in the outer cortex. Parallel-fibered bone tissue and growth marks are present in the outermost region of the cortex (Fig 2G, H), suggesting slower growth rates when the individual died, but the osteocyte lacunae are still abundant. Many secondary osteons are seen in the inner cortex (Fig 2I-J). A thin layer of lamellar endosteal bone is deposited along the edges of the trabeculae in the medullary region (Fig 2F).

The cross-section of the fibula is oval in outline (midshaft diameter 6.5×4 mm, S3 Fig) with a midshaft diameter of 6.3 (AP) × 3.8 (ML) mm. The medullary cavity is small with a diameter of about 2 mm. The cortex mainly consists of fibrolamellar bone tissue, but parallel-fibered bone tissue is shown in the posterior cortex (Fig 2K, L). The cortex is less vascularized than the tibia and humerus. Osteocyte lacunae are abundant but are flattened and organized in parallel-fibered bone (Fig 2M). The primary osteons are predominantly longitudinal and gradually decrease in size towards the periphery. Secondary osteons are mainly in the inner region, but there are also a few near the periphery (Fig 2N). A thin layer of endosteal bone is deposited in the innermost cortex (Fig 2O).

The cross-section of the femur of IVPP V26542 is elliptical with a diameter of 15.0 (AP) ×12.5 (DV) mm and a cortical thickness ranging from 16% to 41% (Fig 3). The estimated bone porosity of the whole thin section is about 38%. The medullary cavity is relatively large (the diameter is about 5 mm) compared to those of juvenile individuals. The thickness of the cortex differs around the bone, and the dorsal cortex is much thinner than the ventral cortex (Fig. 3A). Furthermore, the thickened ventral cortex has a higher porosity (about 40%) than that of the narrow dorsal cortex (about 17%). The cortex mainly consists of fibrolamellar bone and is highly vascularized. The primary osteons show a laminar pattern that is mainly longitudinally oriented, but

radially-oriented channels are found in a narrow channel near the anteroventral cortex (Fig 3A). The number and diameter of the vascular canals are reduced near the periphery. The outer cortex consists of a thin layer of parallel-fibered bone matrix and a growth mark (Fig 3C, D), suggesting slightly reduced growth rates before it died. The secondary osteons are restricted to the innermost cortex (Fig 3E, F). Endosteal bone is present along the trabeculae in the medullary cavity (Fig 3G).

Fig 3. Bone microstructure of the femur in juvenile *Lystrosaurus* IVPP V26542. A, mid-diaphyseal transverse section of the femur; **B**, enlargement of A showing highly vascularized cortical porosity; **C**, enlargement of the outer cortex showing one growth mark (arrow) under normal light; **D**, the same region as C, showing parallel-fibered bone under polarized light; **E**, the inner cortex showing secondary osteons under normal light; **F**, the same region as E under polarized light; **G**, the medullary region showing endosteal bone. **Abbreviation**: **end**, endosteal bone; **rc**, radial canals; **so**, secondary osteons.

Early subadult

The early subadult stage was represented by IVPP V26545. The cross-sections of the fibula, radius, and rib of IVPP V26545 were sampled for observation (Fig. 4). The cross-section of the fibula is subtriangular in outline with a diameter of 13.0 (AP) × 9.8 (ML) mm. The cortex consists of fibrolamellar bone. Most of the vascular canals are longitudinally arranged circumferentially around the bone, forming primary osteons. They are abundant in the inner region with a cortical porosity of about 35% (measured in the lateral cortex), but the number and size decrease from the mid-cortex (cortical porosity about 22%) to the periphery (cortical porosity about

10%). Secondary osteons are restricted to the inner cortex (Fig 4B), which consists of compact coarse cancellous bone (CCCB). A distinct LAG comprised of several growth marks is present near the mid-region of the cortex (Fig 4B). The medullary region is filled with trabeculae (Fig 4A).

Fig 4. Bone microstructure in early subadult *Lystrosaurus* IVPP V26545. A, mid-diaphyseal transverse section of the fibula under normal light. The arrow denotes a LAG. **B**, partial outer cortex of the fibula showing the LAG consists of several growth marks (arrows). **C**, partial outer cortex of the radius under normal light. **D**, mid-cortex showing the LAG consists of several growth marks under polarized light (arrows); **E**, the same region as (D) under normal light. **F**, the whole cross-section of the rib under normal light; **G,** enlargement of the outer cortex of the rib. The black arrow indicates LAGs. **Abbreviation**: **dl**, double LAG; **irc**, irregular canals; **so**, secondary osteons.

The radius is more robust than the fibula, with a diameter of 17.5 (ML) × 14.9 (AP) mm. The cortex mainly consists of woven fibered bone. The vascular canals are primarily longitudinal but there are also some irregular canals in the mid-cortex (Fig. 4C). Secondary osteons and erosional cavities are present in the inner cortex. A clear LAG, as well as a thin layer of parallel-fibered bone, are present at the mid-region as in the fibula (Fig 4D, E). Periosteally from the LAG the density of vascular canals is gradually reduced but are still abundant (Fig 4C). The medullary cavity is large with a diameter of about one-third that of the whole cross-section.

The cross-section of the rib is teardrop-shaped, with a diameter of about 11.0 (AP) ×6.5 (ML) mm (Fig 4F, G). The compact bone is very thin (the average thickness is about 0.6 mm) due to a highly remodeled inner and mid-cortex. The medullary cavity is filled with trabeculae. The

cortex consists of parallel-fibered bone tissues with only a few vascular canals (Fig 4G). The osteocyte lacunae are still abundant and disorganized. A double LAG is present in the outer cortex suggesting one year as the condition in the fibula and radius.

Late subadult

The bone tissue of the femora of IVPP V26546 and IVPP V26547 shows late subadult tissues. In IVPP V26547, the midshaft of the femoral cortex is elliptical in outline with a diameter of 23.5 (AP) ×19.5 (DV) mm (Fig 5). There is no free medullary cavity. The cortex is composed of fibrolamellar bone throughout, and the outer cortex consists of parallel-fibered bone matrix with a thickness of about 1 mm (Fig 5E). Primary osteons are predominantly longitudinal and abundant throughout the cortex except for the region near the mid-cortex LAGs and the outer cortex. Secondary osteons are found in the inner and mid-cortex regions. Two prominent LAGs are present in the mid-cortex (Fig 5D, E). The distance between these two LAGs is narrow (about 0.5 mm), and a thin layer of large primary canals are present around the whole cross-section (Fig 5A, D). In the outermost cortex, the vascular canals are rare and nearly absent (Fig 5F). Two LAGs, as well as growth marks (weak growth lines), are present near the periphery (Fig 5F-H). The osteocyte lacunae are flattened and organized along the growth lines (Fig 5H). The bone histology suggests that this individual grew very slowly before it died, but there is no external fundamental system (EFS), which is present in modern mammals, some dinosaurs, and some dicynodonts [30, 38, 44-4[illegible]

Fig 5. Bone microstructure of the femur in late subadult *Lystrosaurus* IVPP V26547. A, mid diaphyseal cross-section. **B**, partial cross-section under normal light; **C**, the same cortex as B under

crossed plane-polarized light. **D**, enlargement of (B) under normal light, showing LAGs and parallel-fibered bone in the outer cortex. **E**. the same cortex as (C) under polarized light, showing LAGs and parallel-fibered bone. **F**, the outermost cortex under normal light showing LAGs and vascular canals; **G**, the same region as (F) showing parallel-fibered bone; **H**, enlargement of F showing LAGs, osteocyte lacunae and primary osteons. The arrows denote LAGs. **Abbreviation**: **gm,** growth marks; **lpc,** large primary canals

In IVPP V26546, the sampled section near the distal end of the femur is elliptical with a diameter of 26.5 (AP) × 23.0 (DV) mm. It has a thin compact bone (Fig 6). The cortex consists of fibrolamellar bone except for the periphery. The vascular canals are longitudinally-oriented and relatively small in the outer cortex compared to the mid and inner cortices (Fig 6B). Secondary osteons are common throughout the cortex and are also shown near the periphery (Fig 6E, F). A large erosional cavity surrounded by endosteal bone was present in the inner region of the ventral cortex (Fig. 6B). A LAG with a narrow avascular layer of annuli is present in the mid-cortex (Fig 6B, C, G, H), and there are only a few vascular canals around. The osteocyte lacunae are flattened and organized within the annuli (Fig 6G, H). At least two LAGs are present near the periphery (Fig 6C, D). The parallel-fibered bone matrix with LAGs and rare vascular canals suggests that it grew slowly before it died.

Fig 6. Bone microstructure in late subadult *Lystrosaurus* IVPP V26546 and IVPP V26548. (A-H) IVPP V26546. **A**, whole cross-section; **B**, partial transverse section of the femur under normal light; **C**, the outer cortex under polarized light showing parallel-fibered bone and LAGs near the

periphery; **D**, enlargement of the outer cortex showing LAGs; **E**, the outermost cortex showing secondary osteons under normal light; **F**, the same region as (E) under polarized light; **G**, enlargement of the annulus showing the organized osteocyte lacunae; **H**, the same region as G under polarized light; (**I-J**) IVPP V26548. **I**, bone microstructure of the dorsal rib under normal light; **J**, enlargement of the outer region under normal light showing the LAGs. The arrows denote LAGs. **Abbreviation**: **anu**, annulus; **ec**, endosteal cavity; **so**, secondary osteons.

In the largest specimen IVPP V26548, only one dorsal rib was sampled (Fig 6I). The cortex is strongly remodeled in the inner and mid-region, and even in the outermost cortex in some regions. A thin layer of compact bone is shown in the outermost region. It is nearly avascular, with at least two LAGs near the periphery. However, these lines did not extend to the outermost region, and there are still some open vascular canals, suggesting this individual was still growing when it died (Fig 6J).

Discussion

The bone tissue of *Lystrosaurus* consists predominantly of fibrolamellar bone with large channels, similar to those from Africa and India [22, 30]. Three growth stages of *Lystrosaurus* described here were identified: a fast-growing juvenile stage, a slower-growing early subadult, and a late subadult that grew more slowly. The fast growth rates may have helped them to adapt to the environment quickly [30]. The late juvenile was characterized by uninterrupted fibrolamellar bone tissue and the subadult was primarily fibrolamellar bone tissue interrupted by LAGs. The late subadult is characterized by the presence of parallel-fibered bone and LAGs in the outermost cortex.

Bone tissue type in *Lystrosaurus* is not only related to the growth stages but also differs between the various limb bones, including the humerus, femur, tibia, fibula and radius. The humerus, femur and tibia show a mainly laminar to plexiform vascular arrangement with a high cortical porosity in all stages, whereas the vascular canals of the fibula and radius are mostly longitudinally oriented and reduced in density, resulting in a low cortical porosity. In addition, our samples indicate that the humerus and tibia have higher cortical porosity than that of other limb bones, but the parallel-fibered bone with growth marks near the periphery appeared earlier in the humerus than in other elements (IVPP V26544).

Skeletal maturity is usually represented by the presence of an EFS [38], which has been identified in some dicynodonts, including *Placerias* [45] and *Kannemeyeria* [46]. To date, no typical EFS has been detected in *Lystrosaurus*. Ray et al. (2005) found parallel-fibered bone in the outer cortex of a dorsal rib and a thin layer of lamellar bone in a vertebra at the adult stage, but they did not find a typical EFS with closely spaced LAGs near the periphery. In our samples, the parallel-fibered bone in the outer cortex appeared in the dorsal rib and other elements, representing the subadult stage here (Fig 4). Although there is no typical EFS detected, the presence of more LAGs and parallel-fibered bone tissue near the periphery suggests that it grew ~~very~~ slowly before it died.

Botha et al. (2016) found that all Early Triassic *Lystrosaurus* from South Africa have no more than one observed LAG and no parallel-fibered bone in the outermost cortex, which suggests that most individuals did not reach skeletal maturity [19]. A new study on bone microstructure of South African *Lystrosaurus* provided more evidence that the two Early Triassic species were not fully grown. Only three of 12 individuals in *L. declivis* and three of 20 individuals in *L. murrayi* have one LAG in sampled specimens [43]. However, LAGs seem to be more commonly distributed

in *Lystrosaurus* from the Early Triassic of China. Within the seven individuals, three have more than one LAG and one has one LAG. ~~The presence of at~~ least one LAG ~~in *Lystrosaurus* from China has~~ humeri ranging from 12 to 15.6 cm~~,~~ and femora ranging from 12 to 19 cm. This size variation is the most common in Triassic *Lystrosaurus* based on our observation. Comparison of the known humeral length with a LAG between Chinese ~~*Lystrosaurus*~~ and South Africa from Early Triassic [43], ~~we found~~ that their size ~~has no significant~~ differen~~ce~~ (S4 Fig). However, Chinese *Lystrosaurus* tends to have more LAGs, which may be affected by different species or environmental ~~condition~~.

Bone histology in the four *Lystrosaurus* species from the Lower Triassic of South Africa is structurally similar [30, 47], ~~which also occurs in our samples~~. However, IVPP V26545 ~~has a large humerus (15 cm) but has only one LAG that is supposed to be within the early subadult.~~ All the other individuals with humeral lengths larger than 12 cm (including estimated value) have more than two LAGs ~~to be among the late subadult stage~~ (Table 1). In addition, the ratio of the humerus to ulna length is about 1.4, which is larger than that of IVPP RV35012 (1.2). This may indicate a different species of *Lystrosaurus*, which needs to be clarified in the future.

The presence of LAGs and relatively large body s[illegible]of *Lystrosaurus* from China suggest that many *Lystrosaurus* have reached at least the subadult stage, unlike Triassic *Lystrosaurus* from Africa where subadults are rare [19]. This difference may be caused by differing environmental conditions in these areas [23, 48]. A new study found evidence of ~~pronged~~ and repeated metabolic stress in the tusk microstructure of *Lystrosaurus* from the Early Triassic of Antarctica that was thought to be extreme photoperiod seasonality [49]. During the Early Triassic [illegible]ated close to S60º, ~~while~~ the Juggar Basin ~~lied~~ near N35º [50, 51]. It indicates that the former had stronger seasonality that the latter. Although evidence from calcic vertisols indicates that some regions of the Karoo

Basin exhibited a wetting trend towards the *Daptocephalus*-*Lystrosaurus* Assemblage-Zone boundary [9], the hot and arid environment of the Karoo Basin in the Early Triassic has been widely accepted [6, 7, 47]. These harsh environmental conditions may have resulted in high mortality rates of *Lystrosaurus*, with most individuals dying during ~~the~~ early stage [19]. By comparison, the climate is inferred to have been semiarid-subhumid in the Early Triassic of North China [34, 52, 53], which may indicate better environmental conditions than that in South Africa.

Another important issue is whether the body size of *Lystrosaurus* was reduced in the Early Triassic due to the harsh environment such as arid weather. Body size reduction has been shown in non-mammalian eutheriodont therapsids during the end-Permian mass extinction [11], and a reduction is also observed in *Lystrosaurus* from the Karoo Basin of South Africa, but this may be the result of the young stage of the body (indicated by rare LAGs) in the Early Triassic based on bone microstructure [19, 43]. Interestingly, the body size of *Lystrosaurus* from the lower section of the Guodikeng Formation (late Permian), such as *L. youngi* is smaller than the upper section of the Jiucaiyuan Formation, contrasting with the condition in South Africa [32]. This may indicate that the known material of *L. youngi* represents immature individuals, whereas later Chinese *Lystrosaurus* from the Early Triassic are at least subadult. However, the bone microstructure of *Lystrosaurus* from the Guodikeng Formation is poorly preserved, and more material needs to be assessed in the future to confirm this hypothesis.

The lifestyle of *Lystrosaurus* has been argued to be aquatic/semiaquatic [22], fully terrestrial, or burrowing [20, 21]. In recent decades, this controversy has concentrated on aquatic or burrowing habits, which ~~could benefit~~ its survival in the hot environment of the Early Triassic. The aquatic/semiaquatic lifestyle was supported by the bone microstructure showing a thick cortex and

porous vascular canals [22], and linear discriminant analysis based on the compactness profile and body size parameters in extant animals [54, 55]. The available cortical thicknesses of samples described here are between 19% - 41%, which is similar to *Lystrosaurus* from South Africa [22]. In sum, our results also show that *Lystrosaurus* has a thick cortex as in extant aquatic animals, such as *Hippopotamus* [56]. However, the thick cortex is not only shown in aquatic animals but also reported in extant burrowing animals and nonmammalian therapsids [42]. Therefore, a thick cortex could not provide confident evidence of an aquatic or semiaquatic lifestyle for *Lystrosaurus*. Moreover, Juvenile *Lystrosaurus* has been found inside burrow casts indicating that they were active burrowers [24, 25].

Whether *Lystrosaurus* had a substantial aquatic component to its lifestyle in addition to burrowing could be elucidated by analyzing stable isotopes in the future. Previous studies suggest that aquatic and semi-aquatic vertebrates have $\delta^{18}O$ values significantly lower than those of coexisting terrestrial animals [57-59]. Oxygen and calcium isotopes all proved spinosaurids to be aquatic or semiaquatic like modern crocodiles [60, 61], and they could also provide more evidence for the lifestyle of *Lystrosaurus*. Furthermore, $\delta^{18}O$ isotopes have been already used in some late Permian dicynodonts, and the result shows that *Endothiodon* may have had a semi-aquatic lifestyle, whereas *Tropidostoma* was more terrestrial [62].

Conclusion

Here we have studied the bone histology of *Lystrosaurus* from the Early Triassic of China based on thin sections of seven individuals. The results indicate that *Lystrosaurus* grew rapidly in the early stages as previously shown in *Lystrosaurus* from South Africa and India. However, the

presence of LAGs in the largest individual suggests that the growth of *Lystrosaurus* was interrupted in the late stage. LAGs are rare in Triassic *Lystrosaurus* from South Africa but they are more abundant in the *Lystrosaurus* from Xinjiang, China. We propose that most *Lystrosaurus* from Xinjiang had reached the subadult stage, whereas those from South Africa died during their juvenile stage. This distinction may be caused by different environmental conditions, but more samples from the Early Triassic of China are needed to test this hypothesis in the future.

Acknowledgment

We thank Zhang Shukang and Wu Rui for helping to make bone sections, Tong Jinnan and Chu Daoliang for their helpful discussion. We thank Jennifer Botha, Megan Whitney, and Zoe Kulik for their comments on a previous edition of this paper. Megan Whitney and Jennifer Botha also reviewed this version and have provided more constructive and useful comments. We thank the editor Jörg Fröbisch, and the reviewer Christian F. Kammerer and two other anonymous reviewers for their very helpful comments on this manuscript.

References

1. Maxwell WD. Permian and Early Triassic extinction of non-marine tetrapods. Palaeontology. 1992;35(3):571-83.

2. Erwin DH. The Permo-Triassic extinction. Nature. 1994;367(6460):231-5.

3. Stanley SM. Estimates of the magnitudes of major marine mass extinctions in earth history. Proc Natl Acad Sci. 2016;113(42):E6325-E34. doi: 10.1073/pnas.1613094113

4. Retallack GJ. Permian and Triassic greenhouse crises. Gondwana Res. 2013;24(1):90-103. doi:

10.1016/j.gr.2012.03.003.

5. Sun Y, Joachimski MM, Wignall PB, Yan C, Chen Y, Jiang H, et al. Lethally hot temperatures during the Early Triassic greenhouse. Science. 2012;338(6105):366-70. doi: 10.1126/science.1224126.

6. Smith RMH, Botha-Brink J. Anatomy of a mass extinction: Sedimentological and taphonomic evidence for drought-induced die-offs at the Permo-Triassic boundary in the main Karoo Basin, South Africa. Palaeogeogr Palaeoclimatol Palaeoecol. 2014;396(3):99–118.

7. MacLeod KG, Quinton PC, Bassett DJ. Warming and increased aridity during the earliest Triassic in the Karoo Basin, South Africa. Geology. 2017;45(6):483-6. doi: 10.1130/G38957.1

8. Bernardi M, Petti FM, Benton MJ. Tetrapod distribution and temperature rise during the Permian-Triassic mass extinction. Proc R Soc Lond B Biol Sci. 2018;285(1870):20172331. doi: 10.1098/rspb.2017.2331.

9. Gastaldo RA, Kus K, Tabor N, Neveling J. Calcic Vertisols in the upper Daptocephalus Assemblage Zone, Balfour Formation, Karoo Basin, South Africa: Implications for Late Permian Climate. J Sediment Res. 2020;90(6):609-28. doi: 10.2110/jsr.2020.32

10. Song H, Tong J. Mass extinction and survival during the Permian-Triassic crisis. Earth Sci. 2016;41(6):901-18. (in chinese with English abstract)

11. Huttenlocker AK. Body size reductions in nonmammalian eutheriodont therapsids (Synapsida) during the end-Permian mass extinction. PLoS One. 2014;9(2):e87553. doi: 10.1371/journal.pone.0087553.

12. Chu D, Tong J, Song H, Benton MJ, Song H, Yu J, et al. Lilliput effect in freshwater ostracods during the Permian–Triassic extinction. Palaeogeogr Palaeoclimatol Palaeoecol. 2015;435:38-52.

doi: 10.1016/j.palaeo.2015.06.003.

13. Smith RM, Ward PD. Pattern of vertebrate extinctions across an event bed at the Permian-Triassic boundary in the Karoo Basin of South Africa. Geology. 2001;29(12):1147-50. doi: org/10.1130/0091-7613(2001)029<1147:POVEAA>2.0.CO;2.

14. Smith R, Botha J. The recovery of terrestrial vertebrate diversity in the South African Karoo Basin after the end-Permian extinction. C R Palevol. 2005;4(6-7):623-36. doi: 10.1016/j.crpv.2005.07.005.

15. Colbert EH. The distribution of *Lystrosaurus* in Pangaea and its implications. Geobios. 1982;15:375-83. doi: 10.1016/S0016-6995(82)80126-5.

16. Cosgriff JW, Hammer WR, Ryan WJ. The Pangaean Reptile, *Lystrosaurus maccaigi*, in the Lower Triassic of Antarctica. J Paleontol. 1982;56(2):371-85.

17. Fröbisch J. Composition and similarity of global anomodont-bearing tetrapod faunas. Earth-Sci Rev. 2009;95(3):119-57. doi: 10.1016/j.earscirev.2009.04.001.

18. Botha J, Smith RMH. *Lystrosaurus* species composition across the Permo-Triassic boundary in the Karoo Basin of South Africa: *Lystrosaurus* across the PTB. Lethaia. 2007;40(2):125-37. doi: 10.1111/j.1502-3931.2007.00011.x.

19. Botha-Brink J, Codron D, Huttenlocker AK, Angielczyk KD, Ruta M. Breeding young as a survival strategy during earth’s greatest mass extinction. Sci Rep. 2016;6(1):24053. doi: 10.1038/srep24053.

20. King GM, Cluver MA. The aquatic *Lystrosaurus*: An alternative lifestyle. Hist Biol. 1991;4(3-4):323-41. doi: 10.1080/08912969009386548.

21. King GM. The aquatic *Lystrosaurus*: a palaeontological myth. Hist Biol. 1991;4(3-4):285-321.

22. Ray S, Chinsamy A, Bandyopadhyay S. *Lystrosaurus murrayi* (therapsida, dicynodontia): bone histology, growth and lifestyle adaptations. Palaeontology. 2005;48(6):1169–85. doi: org/10.1111/j.1475-4983.2005.00513.x.

23. Ray S, Mukherjee D, Bandyopadhyay S. Growth patterns of fossil vertebrates as deduced from bone microstructure: case studies from India. J Biosci. 2009;34(5):661-72. doi: 10.1007/s12038-009-0055-x.

24. Botha-Brink J. Burrowing in *Lystrosaurus*: preadaptation to a postextinction environment? J Vert Paleontol. 2017;37(5):e1365080. doi: 10.1080/02724634.2017.1365080.

25. Modesto SP, Botha-Brink J. A burrow cast with *Lystrosaurus skeletal* remains from the Lower Triassic of South Africa. Palaios. 2010;25(4):274-81. doi: 10.2110/palo.2009.p09-077r.

26. Tałanda M, DzięCioł S, Sulej T, NiedźWiedzki G. Vertebrate burrow system from the Upper Triassic of Poland. Palaios. 2011;26(2):99-105. doi: 10.2110/palo.2010.p10-024r.

27. Hasiotis ST, Wellner RW, Martin AJ, Demko TM. Vertebrate Burrows from Triassic and Jurassic Continental Deposits of North America and Antarctica: Their Paleoenvironmental and Paleoecological Significance. Ichnos. 2004;11(1-2):103-24. doi: 10.1080/10420940490428760.

28. Özkurt Ö, Yiğit N, Çolak E, Sözen M, Gharkheloo M. Observations on the Ecology, Reproduction and Behavior of *Spermophilus* Bennett, 1835 (Mammalia: Rodentia) in Turkey. Turk J Zool. 2005;29:91-9.

29. Chinsamy A, Rubidge BS. Dicynodont (Therapsida) bone histology: phylogenetic and physiological implications. Palaeontol Afr. 1993;30:97-102.

30. Botha-Brink J, Angielczyk KD. Do extraordinarily high growth rates in Permo-Triassic dicynodonts (Therapsida, Anomodontia) explain their success before and after the end-Permian

extinction? Zool J Linn Soc. 2010;160(2):341-65. doi: 10.1111/j.1096-3642.2009.00601.x.

31. Li J-L. *Lystrosaurus* of China. Vertebr Palasiat. 1988;26(4):241-9. (in Chinese with English abstract)

32. Liu J, Li J, Cheng Z. The *Lystrosaurus* fossils from Xinjiang and their bearing on the terrestrial Permian Triassic boundary. Vertebr Palasiat. 2002;40(4):267-75. (in Chinese with English abstract)

33. Camp J, and Liu J. 2011. The taxonomy and cranial morphology of Chinese *Lystrosaurus*. [Abstract] Supplement to online Journal of Vertebrate Paleontology, 71st Annual Meeting Society of Vertebrate Paleontology: 82

34. Yang W, Feng Q, Liu Y, Tabor N, Miggins D, Crowley JL, et al. Depositional environments and cyclo- and chronostratigraphy of uppermost Carboniferous–Lower Triassic fluvial–lacustrine deposits, southern Bogda Mountains, NW China — A terrestrial paleoclimatic record of mid-latitude NE Pangea. Global Planet Change. 2010;73(1):15-113. doi: 10.1016/j.gloplacha.2010.03.008.

35. Thomas SG, Tabor NJ, Yang W, Myers TS, Yang Y, Wang D. Palaeosol stratigraphy across the Permian–Triassic boundary, Bogda Mountains, NW China: Implications for palaeoenvironmental transition through earth's largest mass extinction. Palaeogeogr Palaeoclimatol Palaeoecol. 2011;308(1):41-64. doi: 10.1016/j.palaeo.2010.10.037.

36. Wan M, Yang W, Wang JJ. A new *Protophyllocladoxylon* wood from the Induan (Lower Triassic) Jiucaiyuan Formation in the Turpan–Hami Basin, southern Bogda Mountains, Rev Palaeobot Palynol. northwestern China. 2019;267:62-72.

37. Horner JR, de Ricqlès A, Padian K. Variation in dinosaur skeletochronology indicators: implications for age assessment and physiology. Paleobiology. 1999;25(3): 295-304. doi:

10.1017/S0094837300021308.

38. Chinsamy-Turan A. The microstructure of dinosaur bone. Baltimore: Johns Hopkins University Press; 2005. 195 p.

39. Young C. On Two Skeletons of Dicynodontia from Sinkiang. Bulletin of the Geological Society of China. 1935;14(4):483-517.

40. Donath K, Rohrer M. Bone Sectioning Using the Exakt System. In: An YH, Martin KL, editors. Handbook of Histology Methods for Bone and Cartilage: Springer; 2003. pp. 243-52.

41. Francillon-Viellot H, de Buffrénil V, Castanet J, Géraudie J, Meunier FJ, Dire JY, et al. Microstructure and mineralization of vertebrate skeletal tissues. In: Carter JG, editor. Skeletal biomineralization: patterns, processes and evolutionary trends. New York: Van Nostrand Reinhold; 1990. p. 471-530.

42. Chinsamy-Turan A. The microstructure of bones and teeth of nonmammalian therapsids. In: Chinsamy-Turan A, editor. Forerunners of mammals: radiation, histology, biology. Bloomington, Indiana: Indiana University Press; 2012. p. 65-90.

43. Botha J. The paleobiology and paleoecology of South African Lystrosaurus. PeerJ. 2020;8:e10408. doi: 10.7717/peerj.10408.

44. Horner JR, De Ricqles A, Padian K. Long bone histology of the hadrosaurid dinosaur *Maiasaura peeblesorum*: Growth dynamics and physiology based on an ontogenetic series of skeletal elements. J Vert Paleontol. 2000;20(1):115-29. doi: 10.1671/0272-4634(2000)020[0115:LBHOTH]2.0.CO;2.

45. Green JL, Schweitzer MH, Lamm ET. Limb bone histology and growth in *Placerias hesternus* (Therapsida: Anomodontia) from the Upper Triassic of North America. Palaeontology. 2010;53(2):

347-64. doi: 10.1111/j.1475-4983.2010.00944.x.

46. Ray S, Botha-Brink J, Chinsamy-Turan AJ. Dicynodont growth dynamics and lifestyle adaptations. In: Chinsamy-Turan A, editor. The forerunners of mammals: radiation, histology and biology. Bloomington: Indiana University Press; 2012. p. 121-48.

47. Botha J, Huttenlocker AK, Smith RMH, Prevec R, Viglietti P, Modesto SP. New geochemical and palaeontological data from the Permian-Triassic boundary in the South African Karoo Basin test the synchronicity of terrestrial and marine extinctions. Palaeogeogr Palaeoclimatol Palaeoecol. 2020;540:109467. doi: 10.1016/j.palaeo.2019.109467.

48. Köhler M, Marín-Moratalla N, Jordana X, Aanes R. Seasonal bone growth and physiology in endotherms shed light on dinosaur physiology. Nature. 2012;487(7407):358-61. doi: 10.1038/nature11264.

49. Whitney MR, Sidor CA. Evidence of torpor in the tusks of *Lystrosaurus* from the Early Triassic of Antarctica. Communications Biology. 2020;3(1):471. doi: 10.1038/s42003-020-01207-6.

50. Torsvik TH, Cocks LRM. Earth History and Palaeogeography. Cambridge: Cambridge University Press; 2016.

51. Li Y-A, Jin X-C, Sun D-J, Cheng Z-W, Pang Q-Q, Li P-X. Paleomagnetic properties of non-marine Permo-Triassic transitional succession of the Dalongkou Section, Jimsar, Xinjiang. Geological Review. 2003;49(5):525-36.

52. Liu D, Zhang C, Yang D, Pan Z, Kong X, Huang Z, et al. Petrography and geochemistry of the Lopingian (Upper Permian)-Lower Triassic strata in the southern Junggar and Turpan basins, NW China: implications for weathering, provenance, and palaeogeography. Int Geol Rev. 2019;61(8):1016-36. doi: 10.1080/00206814.2018.1490932.

53. Tabor NJ, Sidor CA, Smith RMH, Nesbitt SJ, Angielczyk KD. Paleosols of the Permian-Triassic: proxies for rainfall, climate change and major changes in terrestrial tetrapod diversity. J Vert Paleontol. 2017;37(sup1):240-53. doi: 10.1080/02724634.2017.1415211.

54. Germain D, Laurin M. Microanatomy of the radius and lifestyle in amniotes (Vertebrata, Tetrapoda). Zool Scr. 2005;34(4):335-50. doi: 10.1111/j.1463-6409.2005.00198.x.

55. Canoville A, Laurin M. Evolution of humeral microanatomy and lifestyle in amniotes, and some comments on palaeobiological inferences. Biol J Linn Soc. 2010;100(2):384-406. doi: 10.1111/j.1095-8312.2010.01431.x.

56. Wall WP. The Correlation between High Limb-Bone Density and Aquatic Habits in Recent Mammals. J Paleontol. 1983;57(2):197-207.

57. Bocherens H, Koch PL, Mariotti A, Geraads D, Jaeger J-J. Isotopic biogeochemistry (^{13}C, ^{18}O) of mammalian enamel from African Pleistocene hominid sites. Palaios. 1996;11(4):306-18. doi: 10.2307/3515241

58. Fricke HC, Rogers RR. Multiple taxon–multiple locality approach to providing oxygen isotope evidence for warm-blooded theropod dinosaurs. Geology. 2000;28(9):799-802. doi: 10.1130/0091-7613(2000)28<799:MTLATP>2.0.CO;2

59. Clementz MT, Holroyd PA, Koch PL. Identifying aquatic habits of herbivorous mammals through stable isotope analysis. Palaios. 2008;23(9):574-85. doi: 10.2110/palo.2007.p07-054r

60. Hassler A, Martin J, Amiot R, Tacail T, Godet FA, Allain R, et al. Calcium isotopes offer clues on resource partitioning among Cretaceous predatory dinosaurs. Proc R Soc Lond B Biol Sci. 2018;285(1876):20180197. doi: 10.1098/rspb.2018.0197.

61. Amiot R, Buffetaut E, Lécuyer C, Wang X, Boudad L, Ding Z, et al. Oxygen isotope evidence

for semi-aquatic habits among spinosaurid theropods. Geology. 2010;38(2):139-42. doi: 10.1130/G30402.1.

62. Rey K, Day MO, Amiot R, Fourel F, Luyt J, Lécuyer C, et al. Stable isotopes (δ18O and δ13C) give new perspective on the ecology and diet of Endothiodon bathystoma (Therapsida, Dicynodontia) from the late Permian of the South African Karoo Basin. Palaeogeogr Palaeoclimatol Palaeoecol. 2020;556:109882. doi: 10.1016/j.palaeo.2020.109882.

Supporting information

S1 Fig. The sampled positions of all specimens in this study. A, IVPP V26543, arrow denotes the sampled position of the right tibia in lateral view; (**B, C)** IVPP V26544. **B**, the sampled positions of the right tibia and fibula in anterior view; C, the sampled position of the left humerus in ventral view; **D**, IVPP V26542, the distal end of the femur in ventral view; (**E-G)** IVPP V26545. **E**, the sampled position of the left radius (and ulna) in anterior view; **F**, the sampled position of the fibula; **G**, the sampled position of the rib; **H**, IVPP V26547, arrow denotes the sampled position of the femur in dorsal view; **I**, IVPP V26546, arrow denotes the sampled position of the femur in ventral view; **J**, IVPP V26548, arrow denotes the sampled position of the rib.

S2 Fig. Measurements of the cortical thickness in all thin sections. A, IVPP V26543, tibia; **B**, IVPP V26544 tibia; **C**, IVPP V26544, humerus; **D**, IVPP V26544, fibula; **E**, IVPP V26542, femur; **F**, IVPP V26545, fibula; **G**, IVPP V26545, radius; **H**, IVPP V26546, femur; **I**, IVPP V26547, femur. The yellow lines denote measurements of the cortical thickness. The red lines denote measurements of cross-sectional diameters, and the green circles denote outlines of the medullary cavities.

S3 Fig. Bone microstructure and the sampled areas for calculating cortical porosity in all thin sections.

S4 Fig. Comparison of the humeral length between Chinese and South Africa *Lystrosaurus*

Figure 1

Click here to access/download;Figure;Figure 1 R2.tif

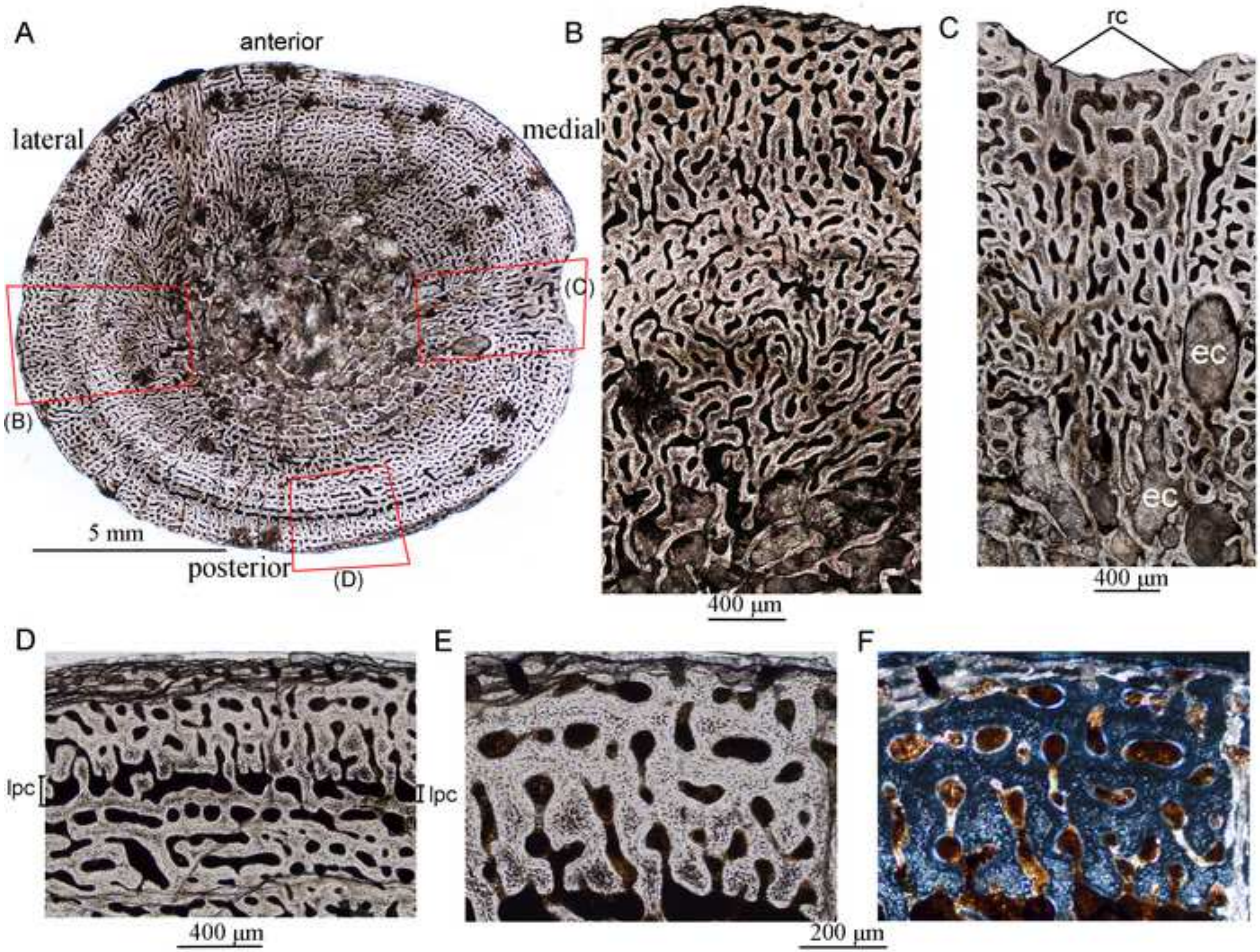

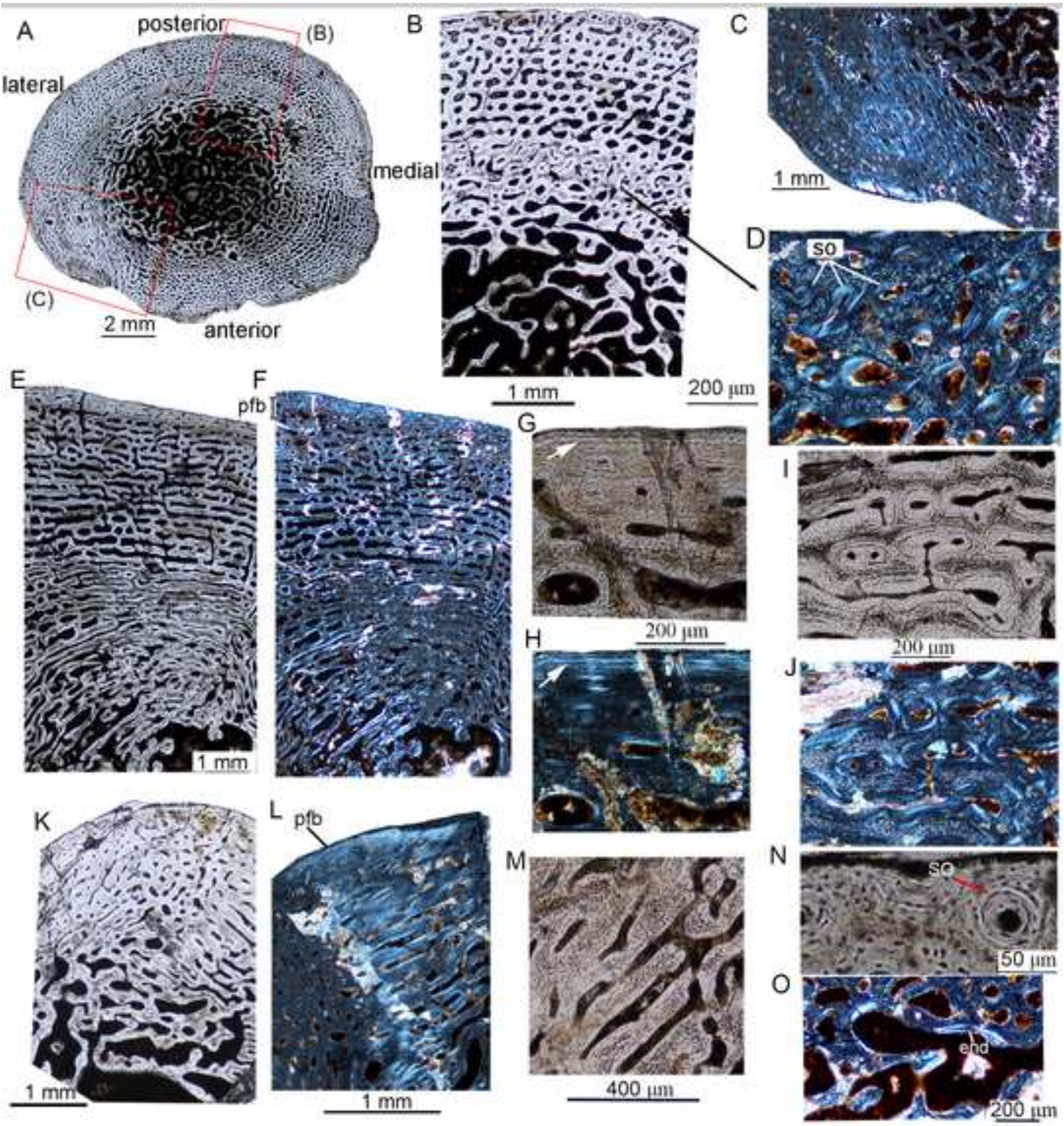

A
posterior
(B)
lateral
medial
(C)
2 mm
anterior
B
1 mm
200 µm
C
1 mm
D
so
E
1 mm
F
pfb
G
200 µm
H
I
200 µm
J
K
1 mm
L
pfb
1 mm
M
400 µm
N
so
50 µm
O
end
200 µm

Figure 3

Click here to access/download;Figure;Figure 3 R2.tif

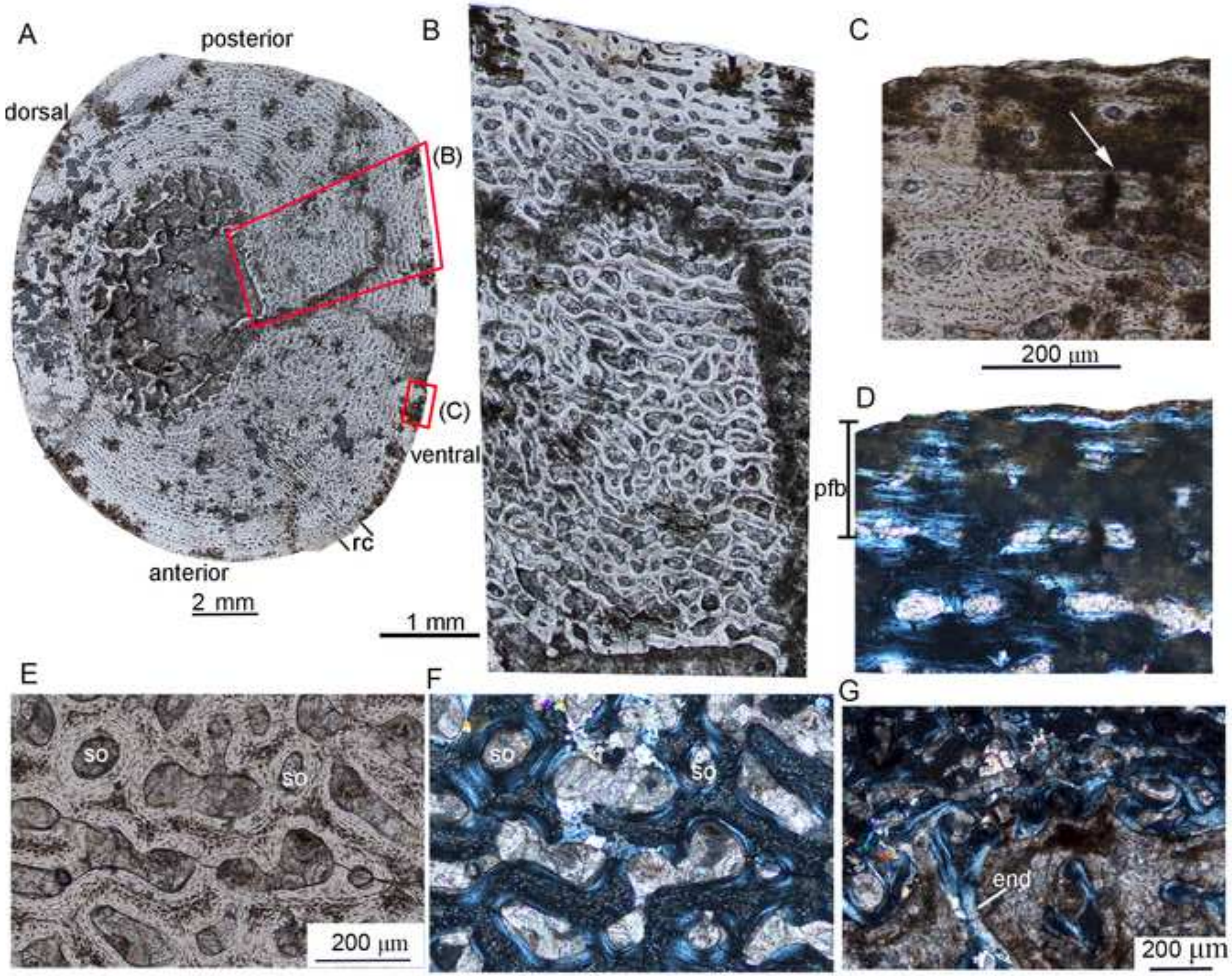

Click here to access/download;Figure;Figure 4 R2.tif

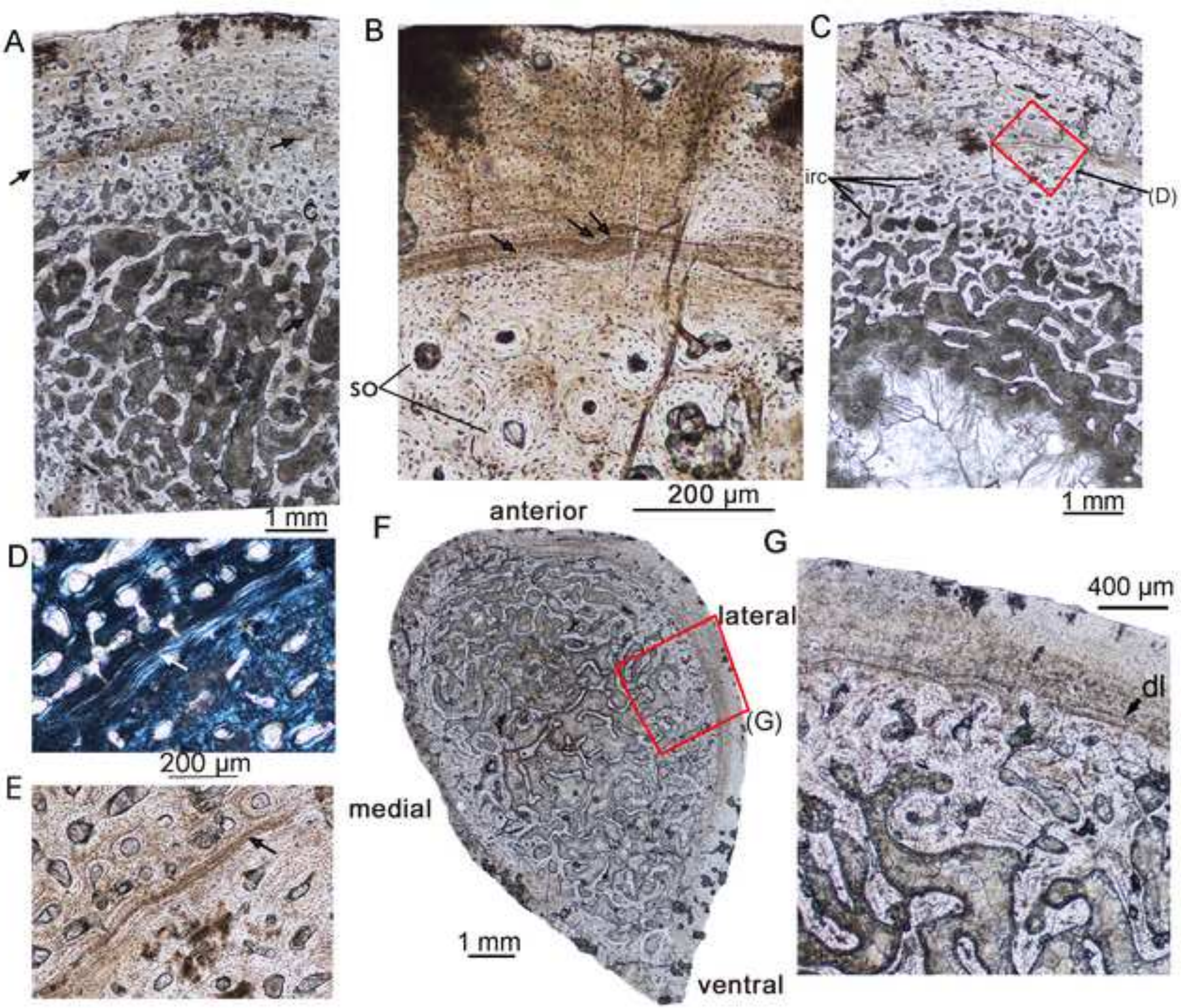

Figure 5

Click here to access/download;Figure;Figure 5 R2.tif

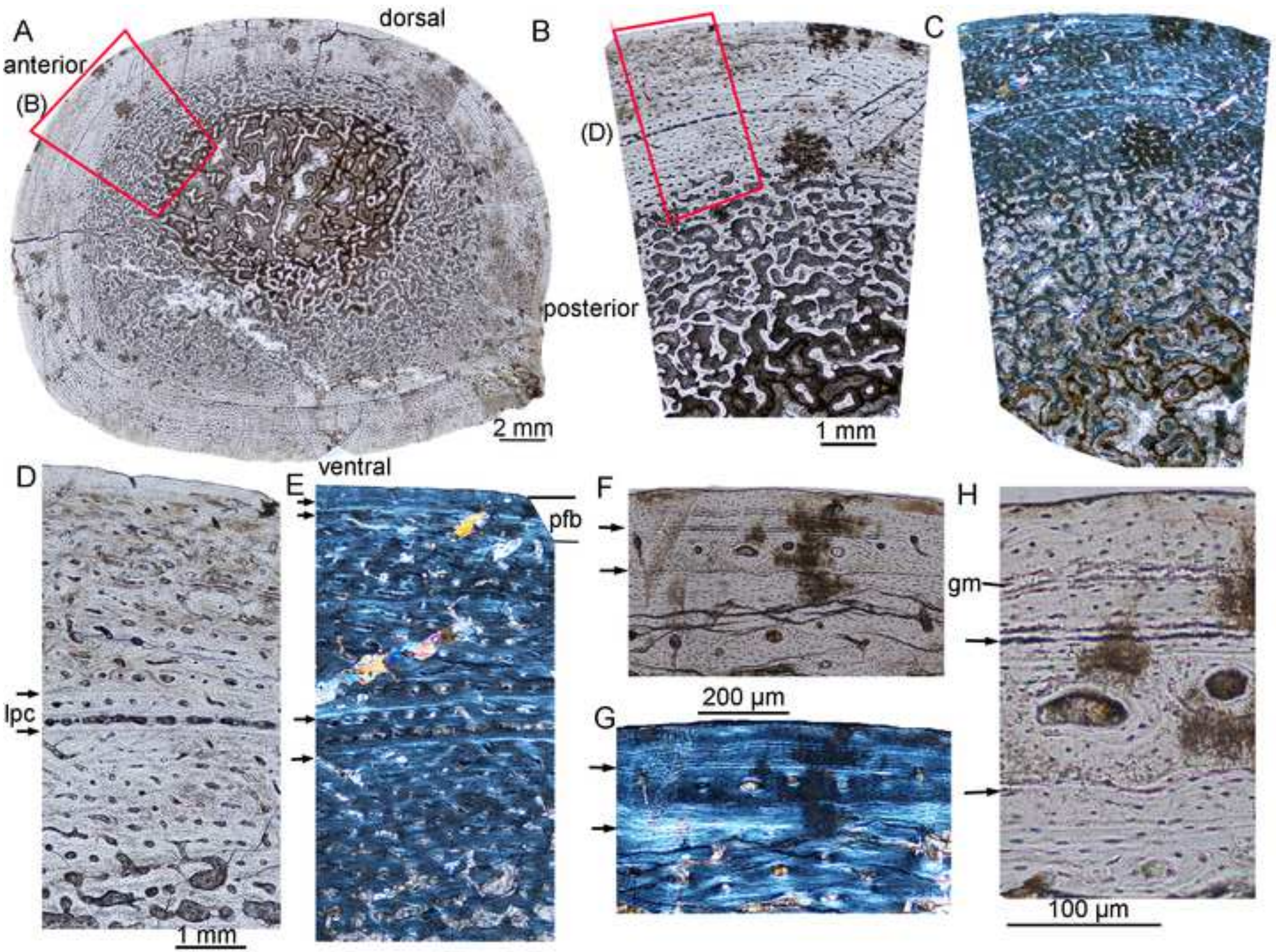

Figure 6 Click here to access/download;Figure;Figure 6 R2.tif

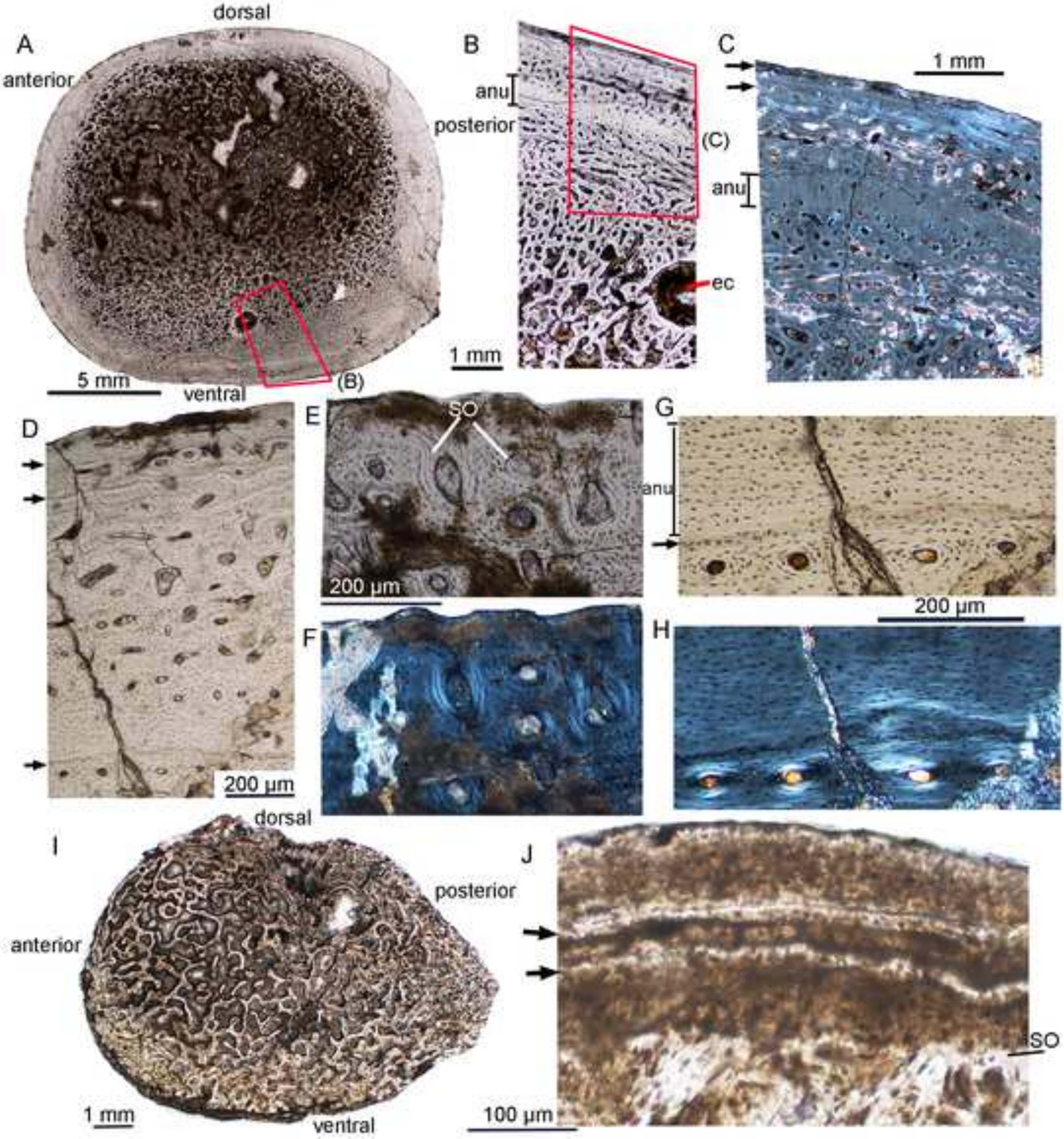

Click here to access/download
Supporting Information
S1 Fig.pdf

Click here to access/download
Supporting Information
S2 Fig.pdf

Click here to access/download
Supporting Information
S3 Fig.pdf

Click here to access/download
Supporting Information
S4 Fig.pdf

Bone histology of *Lystrosaurus* (Therapsida: Dicynodontia) from the Lower Triassic of North China, and its implication for lifestyle and environments after the end-Permian extinction

Fenglu Han[1*], Qi Zhao[2], Jun Liu[2,3, 4*]

[1] School of Earth Sciences, China University of Geosciences (Wuhan), Wuhan, China

[2] Key Laboratory of Vertebrate Evolution and Human Origins of Chinese Academy of Sciences, Institute of Vertebrate Paleontology and Paleoanthropology, Chinese Academy of Sciences, Beijing, China

3 CAS Center for Excellence in Life and Paleoenvironment, Beijing 100044, China;

4 College of Earth and Planetary Sciences, University of Chinese Academy of Sciences Beijing 100049

* Corresponding authors

Email: hanfl@cug.edu.cn (FH); liujun@ivpp.ac.cn (JL)

Abstract

Lystrosaurus represents one of the most successful dicynodonts, a survivor of the end-Permian mass extinction that remained abundant in the Early Triassic, but many aspects of its paleobiology are still controversial. The bone histology of *Lystrosaurus* species from South Africa and India has provided important information on their growth strategy and lifestyle, but until recently no data was available on the bone histology of *Lystrosaurus* from China. Here, we report on the bone microstructure of seven *Lystrosaurus* individuals from the Lower Triassic of Xinjiang, providing the first such data for the Chinese *Lystrosaurus* species. Our samples indicate that the microstructure of *Lystrosaurus* limb bones from China is characterized by fibrolamellar bone tissue similar to those from South Africa and India. Three ontogenetic stages were identified: juvenile, early subadult, and late subadult based on lines of arrested growth (LAGs) and bone tissue changes. Bone histology supports a rapid growth strategy for *Lystrosaurus* during early ontogeny. Unlike Early Triassic *Lystrosaurus* from South Africa, lines of arrested growth are common in our specimens, suggesting interruption in growth in *Lystrosaurus* from China. The differences in bone histology between *Lystrosaurus* from South Africa and China may indicate different environmental conditions in these two regions.

Introduction

The extinction at the end of the Permian was the most influential event in animal history since the Cambrian explosion. About 80% of all marine species and 50-70% of terrestrial families went extinct [1-3]. Recent studies suggest that the Permian-Triassic transition occurred in an increasingly hot environment which was disruptive to life [4-8], although it is still controversial

whether the environment was arid [6, 7] or humid [9]. However, some organisms evolved new survival strategies, such as miniaturization or rapid propagation [10-12].

Lystrosaurus is a medium to large-sized dicynodont therapsid with a strongly anteroventrally downturned snout. It represents one of the most successful vertebrates that survived the end-Permian mass extinction, showing a cosmopolitan distribution during the Early Triassic [13-17]. However, the survival strategies of *Lystrosaurus* during the extinction are still controversial [18, 19]. The lifestyle of *Lystrosaurus* was proposed as being aquatic or semi-aquatic, which could have helped it to escape the harsh terrestrial environment [20, 21], and was supported by histological studies [22, 23]. There is also evidence that *Lystrosaurus* was a burrow maker [24, 25], and burrowing can help an organism survive harsh environmental conditions, as temperature and humidity are generally more stable underground than on the surface [26, 27]. Also, underground burrows can provide better protection for the young, which helps propagate the species [28].

Interestingly, the body size of *Lystrosaurus* from South Africa is smaller in the Early Triassic than in the late Permian [18], suggesting miniaturization. Furthermore, LAGs (lines of arrested growth) are more common in Permian *Lystrosaurus*, which was interpreted as evidence that the Permian species had a more extended ontogeny extended over several years [19]. This contrasts with the prevalence of small individuals with few or no LAGs in the Triassic species, which was part of the evidence suggesting early breeding to compensate for dying at an early age [19].

Bone histology can provide important information on the life history of *Lystrosaurus*, which may bear on its survival during the end-Permian mass extinction. Previous studies have focused on the *Lystrosaurus* record from South Africa and India [22, 23, 29, 30]. However, *Lystrosaurus* seems to have been cosmopolitan in distribution [14], and is also very abundant in the

Juggar and Turpan basins of Xinjiang, China. In China, seven *Lystrosaurus* species have been named since 1935, but the validity of most of these needs re-evaluation [31, 32], and it is possible that only two species (*L. hedini* and *L. youngi*) are valid [33]. Here, we present data on the bone histology of *Lystrosaurus* from the Lower Triassic Turpan Basin, Xinjiang Province, China for the first time, using this data to comment on the survival strategy of this taxon.

Material and methods

Seven specimens of *Lystrosaurus* from the collections of the Institute of Vertebrate Paleontology and Paleoanthropology (IVPP) were selected for histological analysis. All materials come from the Lower Triassic Jiucaiyuan Formation of the Taodonggou area (Taoshuyuan), Turpan Basin, Xinjiang, China [34, 35]. The age of the Jiucaiyuan Formation in this area is middle-upper Induan based on previous chronostratigraphy [34, 36]. Identification of these specimens to species level is currently not possible because the Chinese *Lystrosaurus* taxonomy is currently based on skull characters.

Sections were sampled from the mid-diaphysis of limb bones including the tibia, femur, humerus, ulna, and radius (S1 Fig). However, IVPP V26546 was sampled near the distal end (the distance between the sampled position and the distal end is about 5 cm) of the femur due to the lack of a mid-region. Limb diaphyseal sections were chosen because they are the primary centers of ossification and should preserve the most complete record of growth, and also minimize the potential of secondary remodeling overwriting the growth record (S1 Fig; Table 1) [37, 38]. Rib sections in some individuals were also sampled for comparison. All these materials were photographed and measured before sectioning (S1 Fig; Table 1). However, the sampled specimens

preserved different parts of limb bones making the comparison of body size difficult. Therefore, we estimated values of missing limb bones based on comparing with a complete skeleton of *Lystrosaurus hedini* (IVPP RV35012) [39]. It has a skull length of 173 mm and includes most of the postcranial material. It is probably a subadult individual based on open neurocentral sutures and fused sacral vertebrae. Comparison of the limb bone length (Table 1) with other individuals also ascribes it to be within a subadult stage.

The preparation of the histological sections was carried out at the IVPP. The selected bones were processed using the Exakt-Cutting-Grinding System [40]. Sections were embedded in polyester resin, cut by EXAKT-300CP, and were ground to a thickness of about 50 μm with an EXAKT 400CS grinding machine. The polished sections were then observed under transmitted and polarized light. The thin sections were observed using a Leica DM2700P microscope and photographed using a Leica DMC4500 camera. The whole cross-sections are large and bone microstructure varies in different areas around the cortex, and we have labeled all the sampled directions for all the figures for easy comparison. Nomenclature and definitions of structures follow Francillon-Viellot and Chinsamy-Turan [41, 42]. 'LAGs' are growth mark lines that can be traced around the full circumference. A LAG is a cement line and indicates a temporary but complete cessation in growth whereas the annuli correspond to periods of slow growth by the presence of parallel-fibered bone and flattened osteons [41].

Table 1 Skeletal elements of the *Lystrosaurus* specimens examined for histological sampling from the Early Triassic of Xinjiang, China.

Specimen number	Sampled elements	Humeral length (cm)	Ulna length	Radial length (cm)	Femoral length (cm)	Tibial length (cm)	Length of fibulae (cm)	Growth stages

IVPP V26543	Tibia	9.9^e	8.3^e	7.4^e	12.5	9.32	9.0	Juvenile
IVPP V26544	Humerus, Tibia, Fibula	10.1	7.8^e	7.0^e	11.8	9.17	9.0	Juvenile
IVPP V26542	Femur	10.3	8.3^e	6.52	12.5^e	9.8^e	9.5^e	Juvenile
IVPP V26545	Fibula, Radius	15.0	11.0	10.8^e	18^e	14.2^e	13.8^e	Early subadult
IVPP V26546	Femur	12.0	9.7^e	8.7^e	14.6^e	12.5	11.5	Late subadult
IVPP V26547	Femur	12.4 ^{e}	10^e	9^e	15.1	12.1	11.3	Late subadult
IVPP V26548	Rib	15.6 ^{e}	12.6^e	11.3^e	19.0	14.0	13.5	Late subadult
IVPP RV35012	None	11.5	9.3	8.3	14.0	10.9	10.6	subadult

^{e} denotes estimated values based on the comparison of the ratios between elements of the *Lystrosaurus* IVPP RV35012. The estimated values in each element are all compared to the femur, whereas there is no femur preserved, the estimated values are compared to the humerus. “Growth stages” of all individuals except IVPP RV35012 were identified based on bone microstructure.

The cortical thickness was calculated as the ratio of the cortex (one side) to the cross-sectional diameter of the bone [22]. The cortex can be identified by bone tissue connected tightly forming net and has no bone trabecula and large resorbed cavity, whereas the medullary cavity either empty (free medullary cavity) or filled with trabeculae, which are usually isolated or loosely connected. However, sometimes, the trabeculae are abundant and show a similar connection as in compact bone, and could not be separated clearly in this situation, and then the cortical thickness could not be provided. In addition, cortical thickness usually varies in different parts of the whole cross-section, and we have provided a range of cortical thickness measurements in each section

(Table 2). The measurement positions of the maximum and minimum of the cortical thickness in available sections are shown in S2 Fig. Cortical porosity was measured using the software ImageJ 1.52a (https://imagej.nih.gov/ij/). It was difficult to obtain the cortical porosity of a whole cross-section for each specimen because of partial breakage or poor preservation in some areas of these sections. Therefore, we subsampled ten areas from the inner to the outermost cortex in all sections to calculate the average vascular porosity. The sampled areas were chosen from the inner region to the periphery on different parts of the whole cross-sections (S3 Fig), so they could reflect the value of cortical porosity. However, some bone sections show unclear bone histology in some regions, which may affect the results. Cortical porosities of those bone sections that were poorly preserved or with an unclear transition between compact bone and medullary cavity are not provided. High-resolution images were acquired using a Leica DMC4500 camera. All the images were converted to 8-bit files, and the brightness/contrast was adjusted to clarify the structure. After that, we used "threshold" tools in ImageJ to select all the vascular canals. Finally, the cortical porosity was determined using "analyze particles" tools in ImageJ.

Results

The bone histology of *Lystrosaurus* from North China shows a predominance of fibrolamellar bone tissue and a high cortical porosity (Figs 1-6), as in *Lystrosaurus* from South Africa and India [22, 43]. The primary osteons are predominantly longitudinal, forming laminar or plexiform bone. Radially oriented primary osteons are also present in some parts of the cortex. Secondary remodeling is common in the inner and mid-cortical bone in the subadult stage, resulting in enlarged channels and thin periosteal bone. The medullary cavity is small and filled with bony

trabeculae. LAGs are common in the outer cortex of larger individuals.

All these elements could be divided into juvenile, early subadult and late subadult stages based on bone tissue type and the presence of LAGs (Table 2). They are generally similar to the growth stages of *Lystrosaurus* from South Africa and India, but also have some features that differ from the latter.

Table 2. Bone histological information of *Lystrosaurus* from China in each specimen

Specimen number	Skeletal elements	Cortical thickness (%)	Cortical porosity (%)	Bone tissue type	Secondary reconstruction	Growth rings	Medullary cavity
IVPP V26543	Tibia	24-32	29	plexiform and reticular	inner enlarged channels	0	a small free cavity
IVPP V26544	Humerus	21-45	32	plexiform	a few secondary osteons	0	a small free cavity
IVPP V26544	Tibia	19-30	27	laminar and reticular	a few secondary osteons	0	full of trabeculae
IVPP V26544	Fibula	/	/	laminar	a few secondary osteons	0	a large free cavity
IVPP V26542	Femur	16-41	/	laminar and plexiform	a few secondary osteons	0	a free medullary cavity
IVPP V26545	Fibula	/	/	laminar	a few secondary osteons	1	full of trabeculae
IVPP V26545	Radius	/	/	laminar	a few secondary osteons	1	full of trabeculae
IVPP V26546	femur	/	/	laminar	extensive secondary reconstruction	3	full of trabeculae

IVPP V26547	Femur	/	/	laminar	extensive secondary reconstruction	4	full of trabeculae
IVPP V26548	rib	/	/	laminar	extensive secondary reconstruction	>2	full of trabeculae

[e] denotes estimated values.

Juvenile

The juvenile stage was represented by three samples: IVPP V 26543, VPP V 26544 and IVPP V26542. All the cortices are composed of fibrolamellar tissue, with a high number of vascular canals, abundant globular osteocyte lacunae, and no LAGs, suggesting a rapid, constant growth rate. The mid-diaphyseal tibia of IVPP V 26543 is elliptical in cross-section, with a diameter of 13.5 (ML, mediolateral width)×12.0 (AP, anteroposterior width) mm and a cortical thickness ranging from 24-32% (Fig 1; Table 2). The free medullary cavity is small, with a diameter of about 4 mm. The entire cortex is comprised of fibrolamellar bone and dense vascular canals (Fig 1). The cortex has a high cortical porosity (about 29%, Table 2). The canals are predominantly longitudinal and arrange in circumferential rows in parts. A few irregularly shaped canals are shown in the inner and mid cortex (Fig 1B). Radial anastomoses are shown in the medial and anterolateral regions of the cortex, and the medial region has a concave periosteal surface (Fig 1A, C). The anastomoses join two or three adjacent canals in the outer cortex (Fig 1D) and link four to five canals forming long canals in the mid and inner cortex (Fig 1B). Most of the vascular channels show thin centripetal osteonal deposits forming primary osteons (Fig 1E, F). The primary osteons mainly show laminar and plexiform patterns in the outer and deeper cortex, and a row of large primary canals are shown near the periphery (periosteal surface) in some areas (Fig 1D). Osteocyte density is consistently high

throughout the cortex. Erosionally enlarged resorption cavities are present in the inner cortex (Fig 1C). There are no secondary osteons, LAGs, or endosteal bone. The medullary region contains cancellous bone.

Fig 1. Bone microstructure in juvenile *Lystrosaurus* IVPP V26543. A, whole cross-section of the tibia; **B**, partial cross-section showing fibrolamellar bone; **C**, partial cross-section showing radial vascular canals; **D**, the outer cortex under normal light; **E**, enlargement of the outer cortex showing primary osteons under normal light; **F**, bone tissue of E under polarized light. **Abbreviation**: **ec**, enlarged resorption cavity; **rc**, radial canals; **lpc**, large primary canals

In IVPP V26544, the tibia, humerus, and fibula were all sectioned (Fig 2). The cross-section of the tibia is elliptical in outline, with a diameter of 12.5 (ML) ×10.0 (AP) mm (Fig 2A). The cortical thickness ranges from 19% to 30% (Table 2). There is no free medullary cavity. The cortex mainly consists of fibrolamellar bone tissues (Fig 2B), but parallel-fibered bone tissue is present in the medial region of the cortex that articulates with the fibula (Fig 2C). Vascular canals are predominantly longitudinal. There are a few anastomoses that link two to five canals forming long straight and irregular canals. Most of the vascular canals are primary osteons forming a laminar pattern (Fig 2B). A thin layer (1 mm) of secondary osteons is present in the inner region of the compact bone (Fig 2D). The cortex has a high porosity of 27% (Fig 2A, Table 2), but the diameter of vascular canals is slightly reduced near the periphery. Enlarged vascular canals are present in the inner cortex due to secondary remodeling. No LAG/annulus was detected in the cortex. The medullary region is full of trabeculae.

Fig 2. Bone microstructure in juvenile *Lystrosaurus* IVPP V26544. (A-D) tibia. **A**, mid-diaphyseal cross-section of the tibia. **B**, bone microstructure of the tibia; **C**, partial cross-section of the tibia showing the parallel-fibered bone under polarized light; **D**, enlargement of the inner region of B under crossed plane-polarized light showing secondary osteons; (E-J) humerus. **E**, bone microstructure of the humerus under normal light; **F**, bone microstructure of the humerus under polarized light; **G**, the outermost cortex under normal light showing a growth mark (arrow); **H**, the same region as G under polarized light showing parallel-fibered bone; **I**, the inner cortex showing secondary osteons under normal light; **J**, the same region as H under polarized light; (K-O) fibula. **K**, bone microstructure of the fibula under normal light; **L**, bone microstructure of the fibula under polarized light showing parallel-fibered bone; **M**, enlargement of L showing the organized osteocyte lacunae; **N**, the outermost cortex showing secondary osteons under normal light; **O**, the innermost region showing endosteal bone under polarized light. **Abbreviation**: **end**, endosteal bone; **so**, secondary osteons.

The humerus is subtriangular in cross-section with a midshaft diameter of 18.5 (AP) ×15.5 (DV, dorsoventral width) mm and has a greater cortical thickness than the tibia (Fig 2E, F and S3 Fig). It has a thick compact bone (at least 7 mm) with a cortical thickness ranging from 21% to 45% (Table 2), and the free medullary cavity is extremely small. The cortex is comprised of fibrolamellar bone tissue, but a thin layer of parallel-fibered bone tissue is present near the periphery (Fig 2F-H). The bone porosity has a value of about 32% (Table 2), which is higher than the tibia. The primary osteons are predominantly plexiform and are longitudinally-oriented in the outer cortex. They are

strongly reduced in size in the outer cortex. Parallel-fibered bone tissue and growth marks are present in the outermost region of the cortex (Fig 2G, H), suggesting slower growth rates when the individual died, but the osteocyte lacunae are still abundant. Many secondary osteons are seen in the inner cortex (Fig 2I-J). A thin layer of lamellar endosteal bone is deposited along the edges of the trabeculae in the medullary region (Fig 2F).

The cross-section of the fibula is oval in outline (midshaft diameter 6.5×4 mm, S3 Fig) with a midshaft diameter of 6.3 (AP) × 3.8 (ML) mm. The medullary cavity is small with a diameter of about 2 mm. The cortex mainly consists of fibrolamellar bone tissue, but parallel-fibered bone tissue is shown in the posterior cortex (Fig 2K, L). The cortex is less vascularized than the tibia and humerus. Osteocyte lacunae are abundant but are flattened and organized in parallel-fibered bone (Fig 2M). The primary osteons are predominantly longitudinal and gradually decrease in size towards the periphery. Secondary osteons are mainly in the inner region, but there are also a few near the periphery (Fig 2N). A thin layer of endosteal bone is deposited in the innermost cortex (Fig 2O).

The cross-section of the femur of IVPP V26542 is elliptical with a diameter of 15.0 (AP) ×12.5 (DV) mm and a cortical thickness ranging from 16% to 41% (Fig 3). The estimated bone porosity of the whole thin section is about 38%. The medullary cavity is relatively large (the diameter is about 5 mm) compared to those of juvenile individuals. The thickness of the cortex differs around the bone, and the dorsal cortex is much thinner than the ventral cortex (Fig. 3A). Furthermore, the thickened ventral cortex has a higher porosity (about 40%) than that of the narrow dorsal cortex (about 17%). The cortex mainly consists of fibrolamellar bone and is highly vascularized. The primary osteons show a laminar pattern that is mainly longitudinally oriented, but

radially-oriented channels are found in a narrow channel near the anteroventral cortex (Fig 3A). The number and diameter of the vascular canals are reduced near the periphery. The outer cortex consists of a thin layer of parallel-fibered bone matrix and a growth mark (Fig 3C, D), suggesting slightly reduced growth rates before it died. The secondary osteons are restricted to the innermost cortex (Fig 3E, F). Endosteal bone is present along the trabeculae in the medullary cavity (Fig 3G).

Fig 3. Bone microstructure of the femur in juvenile *Lystrosaurus* IVPP V26542. A, mid-diaphyseal transverse section of the femur; **B**, enlargement of A showing highly vascularized cortical porosity; **C**, enlargement of the outer cortex showing one growth mark (arrow) under normal light; **D**, the same region as C, showing parallel-fibered bone under polarized light; **E**, the inner cortex showing secondary osteons under normal light; **F**, the same region as E under polarized light; **G**, the medullary region showing endosteal bone. **Abbreviation**: **end**, endosteal bone; **rc**, radial canals; **so**, secondary osteons.

Early subadult

The early subadult stage was represented by IVPP V26545. The cross-sections of the fibula, radius, and rib of IVPP V26545 were sampled for observation (Fig. 4). The cross-section of the fibula is subtriangular in outline with a diameter of 13.0 (AP) × 9.8 (ML) mm. The cortex consists of fibrolamellar bone. Most of the vascular canals are longitudinally arranged circumferentially around the bone, forming primary osteons. They are abundant in the inner region with a cortical porosity of about 35% (measured in the lateral cortex), but the number and size decrease from the mid-cortex (cortical porosity about 22%) to the periphery (cortical porosity about

10%). Secondary osteons are restricted to the inner cortex (Fig 4B), which consists of compact coarse cancellous bone (CCCB). A distinct LAG comprised of several growth marks is present near the mid-region of the cortex (Fig 4B). The medullary region is filled with trabeculae (Fig 4A).

Fig 4. Bone microstructure in early subadult *Lystrosaurus* IVPP V26545. A, mid-diaphyseal transverse section of the fibula under normal light. The arrow denotes a LAG. **B**, partial outer cortex of the fibula showing the LAG consists of several growth marks (arrows). **C**, partial outer cortex of the radius under normal light. **D**, mid-cortex showing the LAG consists of several growth marks under polarized light (arrows); **E**, the same region as (D) under normal light. **F**, the whole cross-section of the rib under normal light; **G,** enlargement of the outer cortex of the rib. The black arrow indicates LAGs. **Abbreviation**: **dl**, double LAG; **irc**, irregular canals; **so**, secondary osteons.

The radius is more robust than the fibula, with a diameter of 17.5 (ML) × 14.9 (AP) mm. The cortex mainly consists of woven fibered bone. The vascular canals are primarily longitudinal but there are also some irregular canals in the mid-cortex (Fig. 4C). Secondary osteons and erosional cavities are present in the inner cortex. A clear LAG, as well as a thin layer of parallel-fibered bone, are present at the mid-region as in the fibula (Fig 4D, E). Periosteally from the LAG the density of vascular canals is gradually reduced but are still abundant (Fig 4C). The medullary cavity is large with a diameter of about one-third that of the whole cross-section.

The cross-section of the rib is teardrop-shaped, with a diameter of about 11.0 (AP) ×6.5 (ML) mm (Fig 4F, G). The compact bone is very thin (the average thickness is about 0.6 mm) due to a highly remodeled inner and mid-cortex. The medullary cavity is filled with trabeculae. The

cortex consists of parallel-fibered bone tissues with only a few vascular canals (Fig 4G). The osteocyte lacunae are still abundant and disorganized. A double LAG is present in the outer cortex suggesting one year as the condition in the fibula and radius.

Late subadult

The bone tissue of the femora of IVPP V26546 and IVPP V26547 shows late subadult tissues. In IVPP V26547, the midshaft of the femoral cortex is elliptical in outline with a diameter of 23.5 (AP) ×19.5 (DV) mm (Fig 5). There is no free medullary cavity. The cortex is composed of fibrolamellar bone throughout, and the outer cortex consists of parallel-fibered bone matrix with a thickness of about 1 mm (Fig 5E). Primary osteons are predominantly longitudinal and abundant throughout the cortex except for the region near the mid-cortex LAGs and the outer cortex. Secondary osteons are found in the inner and mid-cortex regions. Two prominent LAGs are present in the mid-cortex (Fig 5D, E). The distance between these two LAGs is narrow (about 0.5 mm), and a thin layer of large primary canals are present around the whole cross-section (Fig 5A, D). In the outermost cortex, the vascular canals are rare and nearly absent (Fig 5F). Two LAGs, as well as growth marks (weak growth lines), are present near the periphery (Fig 5F-H). The osteocyte lacunae are flattened and organized along the growth lines (Fig 5H). The bone histology suggests that this individual grew very slowly before it died, but there is no external fundamental system (EFS), which is present in modern mammals, some dinosaurs, and some dicynodonts [30, 38, 44-46].

Fig 5. Bone microstructure of the femur in late subadult *Lystrosaurus* IVPP V26547. A, mid diaphyseal cross-section. **B**, partial cross-section under normal light; **C**, the same cortex as B under

crossed plane-polarized light. **D**, enlargement of (B) under normal light, showing LAGs and parallel-fibered bone in the outer cortex. **E**. the same cortex as (C) under polarized light, showing LAGs and parallel-fibered bone. **F**, the outermost cortex under normal light showing LAGs and vascular canals; **G**, the same region as (F) showing parallel-fibered bone; **H**, enlargement of F showing LAGs, osteocyte lacunae and primary osteons. The arrows denote LAGs. **Abbreviation**: **gm,** growth marks; **lpc,** large primary canals

In IVPP V26546, the sampled section near the distal end of the femur is elliptical with a diameter of 26.5 (AP) × 23.0 (DV) mm. It has a thin compact bone (Fig 6). The cortex consists of fibrolamellar bone except for the periphery. The vascular canals are longitudinally-oriented and relatively small in the outer cortex compared to the mid and inner cortices (Fig 6B). Secondary osteons are common throughout the cortex and are also shown near the periphery (Fig 6E, F). A large erosional cavity surrounded by endosteal bone was present in the inner region of the ventral cortex (Fig. 6B). A LAG with a narrow avascular layer of annuli is present in the mid-cortex (Fig 6B, C, G, H), and there are only a few vascular canals around. The osteocyte lacunae are flattened and organized within the annuli (Fig 6G, H). At least two LAGs are present near the periphery (Fig 6C, D). The parallel-fibered bone matrix with LAGs and rare vascular canals suggests that it grew slowly before it died.

Fig 6. Bone microstructure in late subadult *Lystrosaurus* IVPP V26546 and IVPP V26548. (A-H) IVPP V26546. **A**, whole cross-section; **B**, partial transverse section of the femur under normal light; **C**, the outer cortex under polarized light showing parallel-fibered bone and LAGs near the

periphery; **D**, enlargement of the outer cortex showing LAGs; **E**, the outermost cortex showing secondary osteons under normal light; **F**, the same region as (E) under polarized light; **G**, enlargement of the annulus showing the organized osteocyte lacunae; **H**, the same region as G under polarized light; (**I-J**) IVPP V26548. **I**, bone microstructure of the dorsal rib under normal light; **J**, enlargement of the outer region under normal light showing the LAGs. The arrows denote LAGs. **Abbreviation**: **anu**, annulus; **ec**, endosteal cavity; **so**, secondary osteons.

In the largest specimen IVPP V26548, only one dorsal rib was sampled (Fig 6I). The cortex is strongly remodeled in the inner and mid-region, and even in the outermost cortex in some regions. A thin layer of compact bone is shown in the outermost region. It is nearly avascular, with at least two LAGs near the periphery. However, these lines did not extend to the outermost region, and there are still some open vascular canals, suggesting this individual was still growing when it died (Fig 6J).

Discussion

The bone tissue of *Lystrosaurus* consists predominantly of fibrolamellar bone with large channels, similar to those from Africa and India [22, 30]. Three growth stages of *Lystrosaurus* described here were identified: a fast-growing juvenile stage, a slower-growing early subadult, and a late subadult that grew more slowly. The fast growth rates may have helped them to adapt to the environment quickly [30]. The late juvenile was characterized by uninterrupted fibrolamellar bone tissue and the subadult was primarily fibrolamellar bone tissue interrupted by LAGs. The late subadult is characterized by the presence of parallel-fibered bone and LAGs in the outermost cortex.

Bone tissue type in *Lystrosaurus* is not only related to the growth stages but also differs between the various limb bones, including the humerus, femur, tibia, fibula and radius. The humerus, femur and tibia show a mainly laminar to plexiform vascular arrangement with a high cortical porosity in all stages, whereas the vascular canals of the fibula and radius are mostly longitudinally oriented and reduced in density, resulting in a low cortical porosity. In addition, our samples indicate that the humerus and tibia have higher cortical porosity than that of other limb bones, but the parallel-fibered bone with growth marks near the periphery appeared earlier in the humerus than in other elements (IVPP V26544).

Skeletal maturity is usually represented by the presence of an EFS [38], which has been identified in some dicynodonts, including *Placerias* [45] and *Kannemeyeria* [46]. To date, no typical EFS has been detected in *Lystrosaurus*. Ray et al. (2005) found parallel-fibered bone in the outer cortex of a dorsal rib and a thin layer of lamellar bone in a vertebra at the adult stage, but they did not find a typical EFS with closely spaced LAGs near the periphery. In our samples, the parallel-fibered bone in the outer cortex appeared in the dorsal rib and other elements, representing the subadult stage here (Fig 4). Although there is no typical EFS detected, the presence of more LAGs and parallel-fibered bone tissue near the periphery suggests that it grew very slowly before it died.

Botha et al. (2016) found that all Early Triassic *Lystrosaurus* from South Africa have no more than one observed LAG and no parallel-fibered bone in the outermost cortex, which suggests that most individuals did not reach skeletal maturity [19]. A new study on bone microstructure of South African *Lystrosaurus* provided more evidence that the two Early Triassic species were not fully grown. Only three of 12 individuals in *L. declivis* and three of 20 individuals in *L. murrayi* have one LAG in sampled specimens [43]. However, LAGs seem to be more commonly distributed

in *Lystrosaurus* from the Early Triassic of China. Within the seven individuals, three have more than one LAG and one has one LAG. The presence of at least one LAG in *Lystrosaurus* from China has humeri ranging from 12 to 15.6 cm, and femora ranging from 12 to 19 cm. This size variation is the most common in Triassic *Lystrosaurus* based on our observation. Comparison of the known humeral length with a LAG between Chinese *Lystrosaurus* and South Africa from Early Triassic [43], we found that their size has no significant difference (S4 Fig). However, Chinese *Lystrosaurus* tends to have more LAGs, which may be affected by different species or environmental condition.

Bone histology in the four *Lystrosaurus* species from the Lower Triassic of South Africa is structurally similar [30, 47], which also occurs in our samples. However, IVPP V26545 has a large humerus (15 cm) but has only one LAG that is supposed to be within the early subadult. All the other individuals with humeral lengths larger than 12 cm (including estimated value) have more than two LAGs to be among the late subadult stage (Table 1). In addition, the ratio of the humerus to ulna length is about 1.4, which is larger than that of IVPP RV35012 (1.2). This may indicate a different species of *Lystrosaurus*, which needs to be clarified in the future.

The presence of LAGs and relatively large body size of *Lystrosaurus* from China suggest that many *Lystrosaurus* have reached at least the subadult stage, unlike Triassic *Lystrosaurus* from Africa where subadults are rare [19]. This difference may be caused by differing environmental conditions in these areas [23, 48]. A new study found evidence of pronged and repeated metabolic stress in the tusk microstructure of *Lystrosaurus* from the Early Triassic of Antarctica that was thought to be extreme photoperiod seasonality [49]. During the Early Triassic, located close to S60º, while the Juggar Basin lied near N35º [50, 51]. It indicates that the former had stronger seasonality that the latter. Although evidence from calcic vertisols indicates that some regions of the Karoo

Basin exhibited a wetting trend towards the *Daptocephalus-Lystrosaurus* Assemblage-Zone boundary [9], the hot and arid environment of the Karoo Basin in the Early Triassic has been widely accepted [6, 7, 47]. These harsh environmental conditions may have resulted in high mortality rates of *Lystrosaurus*, with most individuals dying during the early stage [19]. By comparison, the climate is inferred to have been semiarid-subhumid in the Early Triassic of North China [34, 52, 53], which may indicate better environmental conditions than that in South Africa.

Another important issue is whether the body size of *Lystrosaurus* was reduced in the Early Triassic due to the harsh environment such as arid weather. Body size reduction has been shown in non-mammalian eutheriodont therapsids during the end-Permian mass extinction [11], and a reduction is also observed in *Lystrosaurus* from the Karoo Basin of South Africa, but this may be the result of the young stage of the body (indicated by rare LAGs) in the Early Triassic based on bone microstructure [19, 43]. Interestingly, the body size of *Lystrosaurus* from the lower section of the Guodikeng Formation (late Permian), such as *L. youngi* is smaller than the upper section of the Jiucaiyuan Formation, contrasting with the condition in South Africa [32]. This may indicate that the known material of *L. youngi* represents immature individuals, whereas later Chinese *Lystrosaurus* from the Early Triassic are at least subadult. However, the bone microstructure of *Lystrosaurus* from the Guodikeng Formation is poorly preserved, and more material needs to be assessed in the future to confirm this hypothesis.

The lifestyle of *Lystrosaurus* has been argued to be aquatic/semiaquatic [22], fully terrestrial, or burrowing [20, 21]. In recent decades, this controversy has concentrated on aquatic or burrowing habits, which could benefit its survival in the hot environment of the Early Triassic. The aquatic/semiaquatic lifestyle was supported by the bone microstructure showing a thick cortex and

porous vascular canals [22], and linear discriminant analysis based on the compactness profile and body size parameters in extant animals [54, 55]. The available cortical thicknesses of samples described here are between 19% - 41%, which is similar to *Lystrosaurus* from South Africa [22]. In sum, our results also show that *Lystrosaurus* has a thick cortex as in extant aquatic animals, such as *Hippopotamus* [56]. However, the thick cortex is not only shown in aquatic animals but also reported in extant burrowing animals and nonmammalian therapsids [42]. Therefore, a thick cortex could not provide confident evidence of an aquatic or semiaquatic lifestyle for *Lystrosaurus*. Moreover, Juvenile *Lystrosaurus* has been found inside burrow casts indicating that they were active burrowers [24, 25].

Whether *Lystrosaurus* had a substantial aquatic component to its lifestyle in addition to burrowing could be elucidated by analyzing stable isotopes in the future. Previous studies suggest that aquatic and semi-aquatic vertebrates have $\delta^{18}O$ values significantly lower than those of coexisting terrestrial animals [57-59]. Oxygen and calcium isotopes all proved spinosaurids to be aquatic or semiaquatic like modern crocodiles [60, 61], and they could also provide more evidence for the lifestyle of *Lystrosaurus*. Furthermore, $\delta^{18}O$ isotopes have been already used in some late Permian dicynodonts, and the result shows that *Endothiodon* may have had a semi-aquatic lifestyle, whereas *Tropidostoma* was more terrestrial [62].

Conclusion

Here we have studied the bone histology of *Lystrosaurus* from the Early Triassic of China based on thin sections of seven individuals. The results indicate that *Lystrosaurus* grew rapidly in the early stages as previously shown in *Lystrosaurus* from South Africa and India. However, the

presence of LAGs in the largest individual suggests that the growth of *Lystrosaurus* was interrupted in the late stage. LAGs are rare in Triassic *Lystrosaurus* from South Africa but they are more abundant in the *Lystrosaurus* from Xinjiang, China. We propose that most *Lystrosaurus* from Xinjiang had reached the subadult stage, whereas those from South Africa died during their juvenile stage. This distinction may be caused by different environmental conditions, but more samples from the Early Triassic of China are needed to test this hypothesis in the future.

Acknowledgment

We thank Zhang Shukang and Wu Rui for helping to make bone sections, Tong Jinnan and Chu Daoliang for their helpful discussion. We thank Jennifer Botha, Megan Whitney, and Zoe Kulik for their comments on a previous edition of this paper. Megan Whitney and Jennifer Botha also reviewed this version and have provided more constructive and useful comments. We thank the editor Jörg Fröbisch, and the reviewer Christian F. Kammerer and two other anonymous reviewers for their very helpful comments on this manuscript.

References

1. Maxwell WD. Permian and Early Triassic extinction of non-marine tetrapods. Palaeontology. 1992;35(3):571-83.

2. Erwin DH. The Permo-Triassic extinction. Nature. 1994;367(6460):231-5.

3. Stanley SM. Estimates of the magnitudes of major marine mass extinctions in earth history. Proc Natl Acad Sci. 2016;113(42):E6325-E34. doi: 10.1073/pnas.1613094113

4. Retallack GJ. Permian and Triassic greenhouse crises. Gondwana Res. 2013;24(1):90-103. doi:

10.1016/j.gr.2012.03.003.

5. Sun Y, Joachimski MM, Wignall PB, Yan C, Chen Y, Jiang H, et al. Lethally hot temperatures during the Early Triassic greenhouse. Science. 2012;338(6105):366-70. doi: 10.1126/science.1224126.

6. Smith RMH, Botha-Brink J. Anatomy of a mass extinction: Sedimentological and taphonomic evidence for drought-induced die-offs at the Permo-Triassic boundary in the main Karoo Basin, South Africa. Palaeogeogr Palaeoclimatol Palaeoecol. 2014;396(3):99–118.

7. MacLeod KG, Quinton PC, Bassett DJ. Warming and increased aridity during the earliest Triassic in the Karoo Basin, South Africa. Geology. 2017;45(6):483-6. doi: 10.1130/G38957.1

8. Bernardi M, Petti FM, Benton MJ. Tetrapod distribution and temperature rise during the Permian-Triassic mass extinction. Proc R Soc Lond B Biol Sci. 2018;285(1870):20172331. doi: 10.1098/rspb.2017.2331.

9. Gastaldo RA, Kus K, Tabor N, Neveling J. Calcic Vertisols in the upper Daptocephalus Assemblage Zone, Balfour Formation, Karoo Basin, South Africa: Implications for Late Permian Climate. J Sediment Res. 2020;90(6):609-28. doi: 10.2110/jsr.2020.32

10. Song H, Tong J. Mass extinction and survival during the Permian-Triassic crisis. Earth Sci. 2016;41(6):901-18. (in chinese with English abstract)

11. Huttenlocker AK. Body size reductions in nonmammalian eutheriodont therapsids (Synapsida) during the end-Permian mass extinction. PLoS One. 2014;9(2):e87553. doi: 10.1371/journal.pone.0087553.

12. Chu D, Tong J, Song H, Benton MJ, Song H, Yu J, et al. Lilliput effect in freshwater ostracods during the Permian–Triassic extinction. Palaeogeogr Palaeoclimatol Palaeoecol. 2015;435:38-52.

doi: 10.1016/j.palaeo.2015.06.003.

13. Smith RM, Ward PD. Pattern of vertebrate extinctions across an event bed at the Permian-Triassic boundary in the Karoo Basin of South Africa. Geology. 2001;29(12):1147-50. doi: org/10.1130/0091-7613(2001)029<1147:POVEAA>2.0.CO;2.

14. Smith R, Botha J. The recovery of terrestrial vertebrate diversity in the South African Karoo Basin after the end-Permian extinction. C R Palevol. 2005;4(6-7):623-36. doi: 10.1016/j.crpv.2005.07.005.

15. Colbert EH. The distribution of *Lystrosaurus* in Pangaea and its implications. Geobios. 1982;15:375-83. doi: 10.1016/S0016-6995(82)80126-5.

16. Cosgriff JW, Hammer WR, Ryan WJ. The Pangaean Reptile, *Lystrosaurus maccaigi*, in the Lower Triassic of Antarctica. J Paleontol. 1982;56(2):371-85.

17. Fröbisch J. Composition and similarity of global anomodont-bearing tetrapod faunas. Earth-Sci Rev. 2009;95(3):119-57. doi: 10.1016/j.earscirev.2009.04.001.

18. Botha J, Smith RMH. *Lystrosaurus* species composition across the Permo-Triassic boundary in the Karoo Basin of South Africa: *Lystrosaurus* across the PTB. Lethaia. 2007;40(2):125-37. doi: 10.1111/j.1502-3931.2007.00011.x.

19. Botha-Brink J, Codron D, Huttenlocker AK, Angielczyk KD, Ruta M. Breeding young as a survival strategy during earth’s greatest mass extinction. Sci Rep. 2016;6(1):24053. doi: 10.1038/srep24053.

20. King GM, Cluver MA. The aquatic *Lystrosaurus*: An alternative lifestyle. Hist Biol. 1991;4(3-4):323-41. doi: 10.1080/08912969009386548.

21. King GM. The aquatic *Lystrosaurus*: a palaeontological myth. Hist Biol. 1991;4(3-4):285-321.

22. Ray S, Chinsamy A, Bandyopadhyay S. *Lystrosaurus murrayi* (therapsida, dicynodontia): bone histology, growth and lifestyle adaptations. Palaeontology. 2005;48(6):1169–85. doi: org/10.1111/j.1475-4983.2005.00513.x.

23. Ray S, Mukherjee D, Bandyopadhyay S. Growth patterns of fossil vertebrates as deduced from bone microstructure: case studies from India. J Biosci. 2009;34(5):661-72. doi: 10.1007/s12038-009-0055-x.

24. Botha-Brink J. Burrowing in *Lystrosaurus*: preadaptation to a postextinction environment? J Vert Paleontol. 2017;37(5):e1365080. doi: 10.1080/02724634.2017.1365080.

25. Modesto SP, Botha-Brink J. A burrow cast with *Lystrosaurus skeletal* remains from the Lower Triassic of South Africa. Palaios. 2010;25(4):274-81. doi: 10.2110/palo.2009.p09-077r.

26. Tałanda M, DzięCioł S, Sulej T, NiedźWiedzki G. Vertebrate burrow system from the Upper Triassic of Poland. Palaios. 2011;26(2):99-105. doi: 10.2110/palo.2010.p10-024r.

27. Hasiotis ST, Wellner RW, Martin AJ, Demko TM. Vertebrate Burrows from Triassic and Jurassic Continental Deposits of North America and Antarctica: Their Paleoenvironmental and Paleoecological Significance. Ichnos. 2004;11(1-2):103-24. doi: 10.1080/10420940490428760.

28. Özkurt Ö, Yiğit N, Çolak E, Sözen M, Gharkheloo M. Observations on the Ecology, Reproduction and Behavior of *Spermophilus* Bennett, 1835 (Mammalia: Rodentia) in Turkey. Turk J Zool. 2005;29:91-9.

29. Chinsamy A, Rubidge BS. Dicynodont (Therapsida) bone histology: phylogenetic and physiological implications. Palaeontol Afr. 1993;30:97-102.

30. Botha-Brink J, Angielczyk KD. Do extraordinarily high growth rates in Permo-Triassic dicynodonts (Therapsida, Anomodontia) explain their success before and after the end-Permian

extinction? Zool J Linn Soc. 2010;160(2):341-65. doi: 10.1111/j.1096-3642.2009.00601.x.

31. Li J-L. *Lystrosaurus* of China. Vertebr Palasiat. 1988;26(4):241-9. (in Chinese with English abstract)

32. Liu J, Li J, Cheng Z. The *Lystrosaurus* fossils from Xinjiang and their bearing on the terrestrial Permian Triassic boundary. Vertebr Palasiat. 2002;40(4):267-75. (in Chinese with English abstract)

33. Camp J, and Liu J. 2011. The taxonomy and cranial morphology of Chinese *Lystrosaurus*. [Abstract] Supplement to online Journal of Vertebrate Paleontology, 71st Annual Meeting Society of Vertebrate Paleontology: 82

34. Yang W, Feng Q, Liu Y, Tabor N, Miggins D, Crowley JL, et al. Depositional environments and cyclo- and chronostratigraphy of uppermost Carboniferous–Lower Triassic fluvial–lacustrine deposits, southern Bogda Mountains, NW China — A terrestrial paleoclimatic record of mid-latitude NE Pangea. Global Planet Change. 2010;73(1):15-113. doi: 10.1016/j.gloplacha.2010.03.008.

35. Thomas SG, Tabor NJ, Yang W, Myers TS, Yang Y, Wang D. Palaeosol stratigraphy across the Permian–Triassic boundary, Bogda Mountains, NW China: Implications for palaeoenvironmental transition through earth's largest mass extinction. Palaeogeogr Palaeoclimatol Palaeoecol. 2011;308(1):41-64. doi: 10.1016/j.palaeo.2010.10.037.

36. Wan M, Yang W, Wang JJ. A new *Protophyllocladoxylon* wood from the Induan (Lower Triassic) Jiucaiyuan Formation in the Turpan–Hami Basin, southern Bogda Mountains, Rev Palaeobot Palynol. northwestern China. 2019;267:62-72.

37. Horner JR, de Ricqlès A, Padian K. Variation in dinosaur skeletochronology indicators: implications for age assessment and physiology. Paleobiology. 1999;25(3): 295-304. doi:

10.1017/S0094837300021308.

38. Chinsamy-Turan A. The microstructure of dinosaur bone. Baltimore: Johns Hopkins University Press; 2005. 195 p.

39. Young C. On Two Skeletons of Dicynodontia from Sinkiang. Bulletin of the Geological Society of China. 1935;14(4):483-517.

40. Donath K, Rohrer M. Bone Sectioning Using the Exakt System. In: An YH, Martin KL, editors. Handbook of Histology Methods for Bone and Cartilage: Springer; 2003. pp. 243-52.

41. Francillon-Viellot H, de Buffrénil V, Castanet J, Géraudie J, Meunier FJ, Dire JY, et al. Microstructure and mineralization of vertebrate skeletal tissues. In: Carter JG, editor. Skeletal biomineralization: patterns, processes and evolutionary trends. New York: Van Nostrand Reinhold; 1990. p. 471-530.

42. Chinsamy-Turan A. The microstructure of bones and teeth of nonmammalian therapsids. In: Chinsamy-Turan A, editor. Forerunners of mammals: radiation, histology, biology. Bloomington, Indiana: Indiana University Press; 2012. p. 65-90.

43. Botha J. The paleobiology and paleoecology of South African Lystrosaurus. PeerJ. 2020;8:e10408. doi: 10.7717/peerj.10408.

44. Horner JR, De Ricqles A, Padian K. Long bone histology of the hadrosaurid dinosaur *Maiasaura peeblesorum*: Growth dynamics and physiology based on an ontogenetic series of skeletal elements. J Vert Paleontol. 2000;20(1):115-29. doi: 10.1671/0272-4634(2000)020[0115:LBHOTH]2.0.CO;2.

45. Green JL, Schweitzer MH, Lamm ET. Limb bone histology and growth in *Placerias hesternus* (Therapsida: Anomodontia) from the Upper Triassic of North America. Palaeontology. 2010;53(2):

347-64. doi: 10.1111/j.1475-4983.2010.00944.x.

46. Ray S, Botha-Brink J, Chinsamy-Turan AJ. Dicynodont growth dynamics and lifestyle adaptations. In: Chinsamy-Turan A, editor. The forerunners of mammals: radiation, histology and biology. Bloomington: Indiana University Press; 2012. p. 121-48.

47. Botha J, Huttenlocker AK, Smith RMH, Prevec R, Viglietti P, Modesto SP. New geochemical and palaeontological data from the Permian-Triassic boundary in the South African Karoo Basin test the synchronicity of terrestrial and marine extinctions. Palaeogeogr Palaeoclimatol Palaeoecol. 2020;540:109467. doi: 10.1016/j.palaeo.2019.109467.

48. Köhler M, Marín-Moratalla N, Jordana X, Aanes R. Seasonal bone growth and physiology in endotherms shed light on dinosaur physiology. Nature. 2012;487(7407):358-61. doi: 10.1038/nature11264.

49. Whitney MR, Sidor CA. Evidence of torpor in the tusks of *Lystrosaurus* from the Early Triassic of Antarctica. Communications Biology. 2020;3(1):471. doi: 10.1038/s42003-020-01207-6.

50. Torsvik TH, Cocks LRM. Earth History and Palaeogeography. Cambridge: Cambridge University Press; 2016.

51. Li Y-A, Jin X-C, Sun D-J, Cheng Z-W, Pang Q-Q, Li P-X. Paleomagnetic properties of non-marine Permo-Triassic transitional succession of the Dalongkou Section, Jimsar, Xinjiang. Geological Review. 2003;49(5):525-36.

52. Liu D, Zhang C, Yang D, Pan Z, Kong X, Huang Z, et al. Petrography and geochemistry of the Lopingian (Upper Permian)-Lower Triassic strata in the southern Junggar and Turpan basins, NW China: implications for weathering, provenance, and palaeogeography. Int Geol Rev. 2019;61(8):1016-36. doi: 10.1080/00206814.2018.1490932.

53. Tabor NJ, Sidor CA, Smith RMH, Nesbitt SJ, Angielczyk KD. Paleosols of the Permian-Triassic: proxies for rainfall, climate change and major changes in terrestrial tetrapod diversity. J Vert Paleontol. 2017;37(sup1):240-53. doi: 10.1080/02724634.2017.1415211.

54. Germain D, Laurin M. Microanatomy of the radius and lifestyle in amniotes (Vertebrata, Tetrapoda). Zool Scr. 2005;34(4):335-50. doi: 10.1111/j.1463-6409.2005.00198.x.

55. Canoville A, Laurin M. Evolution of humeral microanatomy and lifestyle in amniotes, and some comments on palaeobiological inferences. Biol J Linn Soc. 2010;100(2):384-406. doi: 10.1111/j.1095-8312.2010.01431.x.

56. Wall WP. The Correlation between High Limb-Bone Density and Aquatic Habits in Recent Mammals. J Paleontol. 1983;57(2):197-207.

57. Bocherens H, Koch PL, Mariotti A, Geraads D, Jaeger J-J. Isotopic biogeochemistry (^{13}C, ^{18}O) of mammalian enamel from African Pleistocene hominid sites. Palaios. 1996;11(4):306-18. doi: 10.2307/3515241

58. Fricke HC, Rogers RR. Multiple taxon–multiple locality approach to providing oxygen isotope evidence for warm-blooded theropod dinosaurs. Geology. 2000;28(9):799-802. doi: 10.1130/0091-7613(2000)28<799:MTLATP>2.0.CO;2

59. Clementz MT, Holroyd PA, Koch PL. Identifying aquatic habits of herbivorous mammals through stable isotope analysis. Palaios. 2008;23(9):574-85. doi: 10.2110/palo.2007.p07-054r

60. Hassler A, Martin J, Amiot R, Tacail T, Godet FA, Allain R, et al. Calcium isotopes offer clues on resource partitioning among Cretaceous predatory dinosaurs. Proc R Soc Lond B Biol Sci. 2018;285(1876):20180197. doi: 10.1098/rspb.2018.0197.

61. Amiot R, Buffetaut E, Lécuyer C, Wang X, Boudad L, Ding Z, et al. Oxygen isotope evidence

for semi-aquatic habits among spinosaurid theropods. Geology. 2010;38(2):139-42. doi: 10.1130/G30402.1.

62. Rey K, Day MO, Amiot R, Fourel F, Luyt J, Lécuyer C, et al. Stable isotopes (δ18O and δ13C) give new perspective on the ecology and diet of Endothiodon bathystoma (Therapsida, Dicynodontia) from the late Permian of the South African Karoo Basin. Palaeogeogr Palaeoclimatol Palaeoecol. 2020;556:109882. doi: 10.1016/j.palaeo.2020.109882.

Supporting information

S1 Fig. The sampled positions of all specimens in this study. A, IVPP V26543, arrow denotes the sampled position of the right tibia in lateral view; **(B, C)** IVPP V26544. **B**, the sampled positions of the right tibia and fibula in anterior view; C, the sampled position of the left humerus in ventral view; **D**, IVPP V26542, the distal end of the femur in ventral view; **(E-G)** IVPP V26545. **E**, the sampled position of the left radius (and ulna) in anterior view; **F**, the sampled position of the fibula; **G**, the sampled position of the rib; **H**, IVPP V26547, arrow denotes the sampled position of the femur in dorsal view; **I**, IVPP V26546, arrow denotes the sampled position of the femur in ventral view; **J**, IVPP V26548, arrow denotes the sampled position of the rib.

S2 Fig. Measurements of the cortical thickness in all thin sections. A, IVPP V26543, tibia; **B**, IVPP V26544 tibia; **C**, IVPP V26544, humerus; **D**, IVPP V26544, fibula; **E**, IVPP V26542, femur; **F**, IVPP V26545, fibula; **G**, IVPP V26545, radius; **H**, IVPP V26546, femur; **I**, IVPP V26547, femur. The yellow lines denote measurements of the cortical thickness. The red lines denote measurements of cross-sectional diameters, and the green circles denote outlines of the medullary cavities.

S3 Fig. Bone microstructure and the sampled areas for calculating cortical porosity in all thin sections.

S4 Fig. Comparison of the humeral length between Chinese and South Africa *Lystrosaurus*

Jan. 6th, 2021

Dear Editor and Reviewers:

Thank you for your letter and for the reviewers' comments concerning our manuscript entitled "Bone histology of *Lystrosaurus* (Therapsida: Dicynodontia) from the Lower Triassic of North China, and its implication for lifestyle and environments after the end-Permian extinction" (ID: PONE-D-20-17684). Those comments are all valuable and very helpful for revising and improving our paper, as well as important for highlighting the significance of our work. We have studied the comments carefully and made a number of corrections to address them, which we hope to meet with approval. The revised portions of the MS were marked by red colors. The main corrections to the paper and the responses to the editor and reviewer's comments are as following:

Reviewer #4: I appreciate all of the work put into this revision and find it greatly improved. I believe the paper now clearly demonstrates methodology and provides sufficient details to warrant publication. Particularly, S2 and S3 figs. Are well presented figures! I find it very helpful and thorough. There are only a few minor line edits that I found that only related to grammar and clarity. I have attached these comments in a marked version of the PDF.

REPLY: Thank you for all your comments and corrections. All the minor comments in the pdf file have been corrected. In addition, the revised version has also been modified a lot based on another reviewer's comments. For example, we have deleted some values of cortical thickness and cortical porosity that were thought to be objective, but we still keep S2 and S3 Figs that can show bone microstructure more clear. We also have added new comparisons between Chinese and South Africa *Lystrosaurus* based on the new published paper by Botha et al. (2020). We hope all these modifications are comfortable for you.

An additional comment refers to S2 fig. The title of the figure says "accurate". I'm not sure what that is in reference to. But it would be better to have the limbs similarly oriented and the view explicitly stated (e.g. anterior).

REPLY: I think you mean S1 Fig. We just pointed the positions that we sampled in the figure. We have removed the word "accurate" and added limb orientation as suggested.

Reviewer #5: This study describes the bone histology of seven *Lystrosaurus* individuals from the Lower Triassic of North China. The data have potential to provide interesting and new information on the growth patterns of *Lystrosaurus* as no studies have yet to focus on this taxon from China, potentially allowing for comparisons with *Lystrosaurus* from South Africa and Antarctica. However, given that the individuals could not be identified to species level (and thus there could be interspecific variation) I would call this study, at most, a preliminary work. They

have very interesting conclusions suggesting that the *Lystrosaurus* from China are larger than those from the Triassic of South Africa and they lived longer (i.e. there are more LAGs in the Chinese specimens compared to the Triassic species from South Africa). This is the most interesting result of the study. However, I have some concerns relating to how much can be concluded from the data and these concerns should be addressed before the manuscript is ready for publication. My concerns are as follows.

1. Only three out of the seven individuals have more than one LAG – and the third element is a rib, which looks like it has a double LAG, not a triple LAG and certainly not an EFS. Double LAGs indicate one season (laid down in one year), so I would not call what you see in the rib as representing multiple seasons. That leaves the two femora – so only two individuals have three or four LAGs. I'm not convinced this is a large enough sample size to indicate that Chinese Triassic *Lystrosaurus* were growing for longer periods compared to South African Triassic species. At most this is a preliminary observation and more work needs to be done to see if you can replicate the data.

REPLY: We agree that our work is just a preliminary observation and much work needs to be done in the future, but we do not think the third element only has a double LAG that represents one year. The third element is a rib, which has three LAGs that were identified previously. We agree that only two prominent LAGs are shown in Fig. 6J, and the third outermost LAG is weak. At least, there are two LAGs present. We supposed it to be two LAGs rather than a double LAG, mainly based on the following evidence: these two LAGs are isolated, vary out of sync, and never combined; the related femur has more than 2 LAGs and a wide parallel-fibered bone at the periphery, which was sampled by another researcher and will be published elsewhere so that could not use in this paper, unfortunately. Furthermore, the LAGs are more closed to each other in the thin cortex (such as ribs, fibula, etc) than in the robust cortex (such as humerus, femur and tibia). For example, see the paper Han et al (2020, the bone histology of *Jeholosaurus*). The distinct between LAGs is much narrower in the fibula than that in the tibia. Finally, this specimen (sampled rib) is the largest individual that we sampled and it is reasonable that it has more LAGs than others.

2. The data used to compare the size of Chinese *Lystrosaurus* with South African *Lystrosaurus* is outdated. Sam-pk-k8 and Sam-pk-8991 do not represent fully grown specimens or the largest known specimens. You can look at Botha 2020 (Botha, J. 2020. The paleobiology and paleoecology of South African Lystrosaurus. PeerJ 8:e10408. DOI: doi.org/10.7717/peerj.10408.). Granted, this publication came out while this study was under review and thus the authors have not had the opportunity to compare the data – but now is the time to do so. There is skull data for the maximum sizes of each South African species as well as size data for the limb bones used – i.e. the authors compare the size of the humerus, this can be done with both Triassic South African *Lystrosaurus* species to see how much larger the Chinese species is – if they really are notably

larger and this can be confirmed, then it is a very interesting result, but this needs to be more adequately confirmed.

REPLY: Thank you for your new information. We have added more comparison of the known humeral length with a LAG between Chinese *Lystrosaurus* and South Africa from Early Triassic, we found that their size has no significant difference (S4 Fig). But Chinese *Lystrosaurus* still has a relatively large proportion with LAGs compared to South Africa *Lystrosaurus*. The humeral length larger than 10 cm is the most common size based on our observation. Both the body size and bone microstructure support that many *Lystrosaurus* have reached at least the subadult stage, unlike Triassic *Lystrosaurus* from Africa where subadults are rare. Of Course, we need more work to clarify this conclusion.

3. I disagree with the last ontogenetic stage being labelled adult. An adult will have a significant amount of slower growing tissues (parallel-fibered or lamellar) and/or an EFS – neither of which is present in the largest individuals. There is the beginning of parallel-fibered bone, so what the authors have captured here is the beginning of slower growth which would make these individuals subadults. It also cannot be confirmed if what is seen in the rib is an overall decreased growth or just parallel-fibered bone associated with a double LAG after which growth may have increased again. The two femora are undoubtedly older than the other individuals, but they are not adult. You could perhaps divide the stages into juvenile, early subadult and late subadult – although even saying late subadult is a push because there is so little parallel-fibered bone.

REPLY: We have changed the word "adult" to "late subadult" stage as suggested.

4. I have a problem with the methodology in calculating the cortical thickness of the bones. The problem with most dicynodonts is that they do not have a sharp transition from a clear open medullary cavity to a compact cortex. There is generally, and this is seen in Lystrosaurus, a gradual transition zone and there are often trabeculae within the medullary cavity. So if you are measuring the compact bone manually as the authors have done here, where do you objectively draw the line between compact bone and medullary cavity? It is very difficult to standardize and objectively measure cortical thickness in this way. This is why the program Bone Profiler is better than a manual procedure because the computer measures so many transects all around the bone. I don't think any of the cortical thickness measurements are useful if they are done manually (unless you have sharp transition zones, which you don't in Lystrosaurus). These calculations cannot be compared with other Lystrosaurus specimens or with other taxa. However, using Bone Profiler to calculate cortical thickness (which is generally K in the program) is a lot of work as the cross-sections needs to be changed into black and white images, which can only be done manually by colouring in the bone black and the spaces white – this is extremely time consuming. I thus, suggest that the entire cortical thickness section be deleted. The values cannot be reliably used and they don't say much about the lifestyle of Lystrosaurus anyway. If the authors wish to keep the cortical thickness in the paper then they need to use Bone Profiler to obtain their values.

REPLY: Thank you for your suggestions. In fact, we tried to use the software bone profiler after your suggestion in your first review. But we found many problems and we gave up finally. First, we did not find a valid link that could download this software. The official link provides a new version that analyses on the website but was also not available for analysis right now. In addition, I do not quite understand the principle of the software. All the bone has the same color, then how to separate compact bone and cancellous bone in the program? Girondot and Laurin (2003, p460, secondary paragraph) mentioned that K was the ratio between the internal and external diameter of the bone, so it should not represent the cortical thickness that we mentioned here.

Finally, we agree that a gradual transition from compact bone to medullary cavity has happened in *Lystrosaurus*, but not all specimens are difficult to separate. There is still a clear change in some specimens, such as the femur IVPP V26542. This situation is also mentioned by Ray et al (2005). So in the revised version, we keep the cortical thickness values that we can discern the transition clearly and have omitted other values with a vague transition between compact bone and medullary region. Furthermore, we have also omitted cortical porosity values with vague transitions.

5. There is also a problem with the methodology for calculating cortical porosity. Fig S3 shows where the fields of view were taken – A, B and C are ok, but in D Field Of View 8, 9 and 10 are taken from the inner regions of the cortex, some of which includes resorption cavities – these cannot be compared with primary vasculature – so 8, 9 and 10 would give you inflated values compared to the others. Even in FOV 1-4 the FOV includes enlarged cavities and not just primary vasculature – this again inflates the values compared to 5-7. FOV is generally taken from the mid-cortex only and all 10 FOVs are taken from the "same" place giving you an average over 10 FOV. One can do what was done in A, B and C because each FOV is the same. E is also a problem because some FOVs are from the outer cortex, some from the middle and some from the inner regions – so you're not standardizing where you're taken the FOV from. Yes, you're getting an average of the bone, but you're doing it in a different way each time. F and particularly G I'm worried about as the inner regions of the FOV are resorption cavities which you cannot include in primary vasculature – so here, the entire bone's values are inflated, same with H – in fact in H, there is even medullary cavity, so was this included as space or was it ignored? If the latter then your FOV is smaller depending on where the FOV was taken, so you're not comparing same-sized FOVs. This is going to produce unreliable results.

REPLY: We agree that there are still some problems with measuring cortical porosity. The best way is to measure the whole compact bone. However, there are at least two problems: As we mentioned above, there is no sharp transition between compact bone and medullary cavity in some specimens, and the areas that we sampled may be objective. Therefore, in the revised version, we only keep the cortical porosity values with clear tissue change. Secondly, some bone sections do not show clear bone tissue in some parts due to bad preservation, such as Fig S3 E. It has a clear

medullary cavity, but not allowed us to standard the acute position. It just provides a reference, that is why we mentioned that it was an estimated value in the method. Nevertheless, we have omitted this value according to the reviewer's comments.

6. It would also be good to include Whitney, M.R. and Sidor, C.A. 2020. Evidence of torpor in the tusks of *Lystrosaurus* from the Early Triassic of Antarctica. Communications Biology 3:471. DOI: 10.1038/s42003-020-01207-6. Although they do not deal with limb bones (they use teeth) they do talk about different environments affecting Lystrosaurus. This publication may have come out while this study was still in review – but it would be good to include it now for a discussion on differing environments affecting *Lystrosaurus* growth.

REPLY: We have added this reference and mentioned that environments can affect tusk and bone microstructure of *Lystrosaurus*. But we did not discuss a lot because it was difficult for comparison between teeth and bone microstructure.

Comments in the pdf

P5, line 10

Comment: you need to explain the difference between a LAG and an annulus. i.e. a LAG is a cement line and indicates a temporary, but complete cessation in growth whereas an annulus consists of lamellar or parallel-fibered bone and indicates a temporary decrease in growth rate

REPLY: We have added this information.

Table 1. Comment: fix this column so that the s is not on another line

REPLY: Modified as suggested.

P6, line 2

why was this specimen chosen? is it the largest known in China? is it the most complete? does it have a skull? can you identify it to species level? You say that the ontogenetic stage is unknown - but is it small or big? are sutures fused? are their muscle scars? etc you must be able to estimate the ontogenetic stage of this specimen. Are you estimating all these other values based on a juvnenile or an adult?

REPLY: we have added more information on this specimen in the method. It is the holotype of *L. hedini* and represents the most complete specimen of *Lystrosaurus* (Young 1935). It has a skull length of 173 mm and includes most of the postcranial material. It is probably a subadult individual based on open neurocentral sutures and fused sacral vertebrae. It has a humeral length of 11.5 cm, which is more suitable to assign it to be within the subadult stage compared to others.

P6, paragraph 1

Comment: This is an outdated method for calculating cortical thickness and is problematic in an animal such as *Lystrosaurus* because there is no clear strict transition between cortical bone and medullary cavity. The broad transition zone in this region makes measurements calculating cortical thickness quite subjective. I recommend using Bone Profiler for this method. These measurements will then be more useful in that they can be compared with other studies.

REPLY: We have already given a detailed explanation above. We have omitted controversial data but keep some measurements that have a clear transition. As we provided the accurate position that we measured (Fig. S2). The reader can choose whether to use it or not.

P6, paragraph 1, Line 11
This method differs from that of previous studies - which take 10 fields of view from the mid-cortex all around the cross-section. It is unclear if you are doing a transect from the inner to outer cortex and getting the average, or if you are taking inner versus outer fields of view randomly all over the cross-section - if the latter then this method is not being very consistent. You need to standardize this method more.

REPLY: We have given a detailed response above. In addition, we are doing an average cortical porosity from the inner to outer cortex rather than the mid cortex. As we know that cortical porosity is different from the inner to outer cortex, so it also has a problem to just choose mid-cortex, because cortical porosity is gradually changed from the inner to outer cortex. How do we define the mid cortex is a problem and objective.

P7, paragraph 2.
Comment: There is a new reference which will help you compare your results to the SA Lystrosaurus, you need to include this:
Botha, J. 2020. The paleobiology and paleoecology of South African *Lystrosaurus*. *PeerJ* 8:e10408. DOI: doi.org/10.7717/peerj.10408.

REPLY: Thank you for your information. We have added this reference and more comparison in the discussion part.

P7, table 2
Comment: separate into two separate columns

REPLY: modified as suggested.

P7, table 2
Comment: so would these values mark the highest being the inner cortex and the lowest value being the outer cortex?

REPLY: Sorry for the misunderstanding. Providing the variation of cortical thickness was suggested by Megan Whitney. It is the ratio of compact bone (one side) to the diameter of the whole cross-section. For example, see Fig S2E. The dorsal cortex is thin and the ventral cortex is relatively thick, so the cortical thickness ranges from 16-41%. Please see the method part for detailed information.

P8, table 2

extensive secondary reconstruction would imply dense Haversian bone, or that the reconstruction has reached the outer cortex - is this the case for your elements here? It doesn't look like it from the figures

REPLY: Thank you for pointing it out. We have modified this sentence as “a few secondary osteons”

P8, Juvenile, line 5.

Comment: where would you denote the edge of the medullary cavity here with such a gradual transition zone? You need to explain how you got these measurements and how you ensured that they were not subjective, but standardized

REPLY: We have added more explanation on how to identify compact bone and medullary cavity. The compact bone tissues are connected tightly without bone trabecula and large resorbed cavity, whereas the medullary cavity either empty (we call it free medullary cavity) or filled with trabecula, which is usually isolated or loosely connected. However, sometimes, the trabecula is abundant and show a similar connection as in compact bone, and could not be separated clearly.

P9 line 2. Comment: in circumferential rows in parts?

REPLY: Yes, added.

P9, line 11.

not shown in Fig 1B - label or take out in the text here

REPLY: The word “Fig. 1B” has been taken out here.

P9, figure 1. I know what you mean here by wide-open canals - but this isn't a very good term. Just label them large primary canals because that's what they are.

REPLY: Thank you for your suggestions. Modified.

P9, last line.

Comment: then how did you calculate cortical thickness?

P10 line 8. Comment: then how accurate is the cortical thickness measurement?

REPLY: We have provided more explanation on defining medullary cavity and compact bone in the method, See our explanation above.

P10, Fig 2 caption N.

Comment: I'm not convinced those are secondary osteons - I cannot see a cement line or How ship's lacunae - you need a better close up of this

REPLY: We have changed to a close up new picture to show secondary osteon here.

P11 paragraph 2 line 11.

Comment: according to your definition then - these growth marks cannot be seen around the whole circumference of the bone? So are they really indicative of a change in growth rate?

REPLY: These growth marks usually associated with parallel-fibered bone in the outermost region, so we consider it to indicate a slow growth rate.

P12 Subadult. Line 11.

Comment: This is not shown from Fig 3A and B - I would not call those channels "significantly reduced"

REPLY: we have removed the word "significantly".

P12 Subadult. Line 12. can this LAG be followed around the whole circumference?

REPLY: This LAG is weak and not clear in some parts under normal light, and it is also not clear under polarized light, so we revised it to be a growth mark in the revised MS. Therefore, we remove this individual to be within a juvenile stage.

P13. Line 1. cannot see the LAG here

REPLY: We have removed the word "a LAG" here.

P13 paragraph 2 Line 13

It looks like a double LAG - don't say several otherwise it sounds like an EFS.

REPLY: We did not say several LAGs. There is only one prominent LAG and we wrote "a distinct LAG comprised of several growth marks". It does show more than two growth marks (weak growth lines) but not LAGs. We remarked the arrow positions to show growth marks that more clear.

P13 Figure 4 caption. Line 3.

Comment: I'm not convinced - looks like two not three. And does the multiple number stay consistent around the whole cortex or does it become one LAG at some point?

REPLY: Again, we did not say it has three LAGs, instead, it is just one LAG. But we can see that it contains several growth marks. It becomes narrower but still a distinct LAG in other regions.

P13 Figure 4 caption. Line 5.

Comment: this looks like a single LAG here

REPLY: Yes. We agree. It is a single LAG containing several growth marks (or weak growth lines).

P14 line 1. Comment: looks like a double LAG here too

REPLY: Yes. We agree and have modified in the revised MS.

P14 line 1. this isn't a normal term - they are either resorption canals or canals that are just starting to form - look to see which one

REPLY: Yes. Here we only describe its shape that was suggested by another reviewer. They probably represent resorption canals.

P14 paragraph 2 line 6

Comment: so what are these? how far do they go around the cortex? do they run with the LAG or are they separate?

REPLY: Here we mean that a clear LAG was formed by several growth marks (weak growth lines that vary throughout the whole cortex), but we rethink parallel-fibered bone is more suitable. We have modified this sentence as "A clear LAG, as well as parallel-fibered bone, are present at the mid-region as in the fibula".

P14 paragraph 2 line 7

Comment: cannot still be abundant but also strongly reduced - rephrase - I wouldn't call the canals strongly reduced. Perhaps there is a slight decrease in vascular canal size towards the periphery

REPLY: We have changed the word "strongly" to "gradually".

P14 adult stage.

Comment: how do you define adult tissues?

REPLY: Adult stage was usually defined by the presence of EFS or lamellar/parallel-fibered bone tissue at the periphery. Here, we found parallel-fibered bone at the periphery. However, we agree that the layer of parallel fibered bone was still narrow. We have changed this stage to "late subadult" as suggested.

P15 Line 1

Comment: as with my comments above for every element - how then do you decide where the edge of the compact cortex is?

REPLY: cortical thickness and porosity have been removed in this specimen.

P15 paragraph 1 last line.

Comment: you've added in *Placerias* - but an EFS has been found in *Kannemeyeria* as well - see Botha-Brink and Angielczyk 2010 - those are the only two so you may as well name them seen as they are dicynodonts

REPLY: We have added this reference.

P15 Fig 5 caption.

Comment: I am not convinced this is an adult - at most, with such a small region of parallel-fibered bone

at the periphery we are seeing a first transition to slower forming bone tissues - so at most this is a subadult. An adult would have a much larger region or parallel-fibered bone or lamellar bone or an EFS

REPLY: We have changed the stage “adult” to “late subadult”.

P15 Last line.
Comment: change this term
REPLY: Modified.

P16 Fig. 6. Comment: looks like a double LAG not triple
REPLY: This should be at least two LAGs rather than a double LAG. These two LAGs have more than three LAGs and a wide parallel fibered bone have been founded in the associated femur. But it has already been sampled by another research and will be published elsewhere.

P17. Line 5 I would say two
REPLY: Modified as suggested.

P17. Line 5.
Comment: no - not enough to indicate an EFS - looks like a double LAG. This is not an EFS - as you decide it's not one from your next comment - rather just take out the phrase "similar to an EFS"
REPLY: We have deleted this sentence.

P17 Discussion. Line 4.
Comment: There is not enough evidence to indicate this third stage - the rib cannot be used for this and the two femora look like subadults
REPLY: We have modified the “adult” to “late subadult” and modified the text to make it more clear.

P17 Discussion. Line 6.
no - you don't know that - no EFS - a double LAG is not enough to indicate general slow growth - definite subadult, but I wouldn't call these femora and rib adults

REPLY: We have changed the “adult” to “late subadult” stage.

P17 Discussion. Line 6.
Comment: what about the humerus?

REPLY: We have added the word“the humerus”.

P18 Paragraph 3 line 4.
Comment: only three out of seven have more than 1 LAG - and the rib looks like a double LAG - i.e. indicating one season, the rib is not a good one to include in stating that more than 1 or 2 LAGs are

present. That just leaves the two femora that have 3 or 4 LAGs - this is not a high sample size

REPLY: As we mentioned above, the rib in Figure 6 has at least two LAGs, and this individual also sampled the femur which has more than 2 LAGs and wide parallel-fibered bone tissue at the periphery. But it has been sampled by another researcher and will be published elsewhere.

P18 Paragraph 3 line 8

these are not the largest Triassic Lystrosaurus - compare your elements with Botha 2020

P18 Paragraph 3 line 9

confirm this by comparing with the largest Triassic humeri in Botha 2020

REPLY: We have added a new comparison and S4 Figure according to this new reference, and we found that there is no significant size difference between Chinese and South Africa *Lystrosaurus*, but Chinese *Lystrosaurus* tends to have more LAGs, which may be affected by different species or environment. This did not affect our conclusion that Chinese *Lystrosaurus* have reached at least the subadult stage based on the presence of LAGs and relatively large body size (see revised MS for detailed information).

P19. Line 3.

Comment: you don't show this in the table - in the table it's just the two femora that have more than two LAGs. There is only one juvenile humerus in your tables

REPLY: We have modified this sentence as “All the other individuals with humeral length larger than 12 cm (including estimated value) have more than two LAGs to be among late subadult stage (Table 1)”. The two femora IVPP V26546 and IVPP V26547 have more than two LAGs. IVPP V26546 also preserves a humerus (12 cm), although we did not sample it. IVPP V26547 and IVPP V26548 do not preserve the humeri, but the estimated humeral length is larger than 12 cm. We have changed the “Elements” to “Sampled elements” to make it more clear.

P20 Line 1

Comment: yes but this has been shown to be false in Botha 2020 - the size reduction is just because they didn't live long enough to attain large sizes, not because of miniaturization.

REPLY: We have added this new reference.

P20 paragraph 2 line 7

Comment: cannot start a sentence with and

REPLY: Modified.

P20 paragraph 2, line 8

Comment: not necessarily - it depends on where you sampled your rib. Cortical thickness in ribs differ along the length of the shaft and you haven't said where your sample was taken from - so it's very difficult to compare ribs unless you know for sure they were sampled at the same position along the shaft

REPLY: We have deleted this sentence.

Sincerely,
Fenglu Han
