# Supplementary material for: Preliminary bone histological analysis of Lystrosaurus (Therapsida: Dicynodontia) from the Lower Triassic of North China, and its implication for lifestyle and environments after the end-Permian extinction
Source: PLoS One. 2021 Mar 18;16(3):e0248681. doi: 10.1371/journal.pone.0248681 (PMC7971864; doi:10.1371/journal.pone.0248681)
Supplement: Attachment — Submitted filename: lystrosaurus histology edits.docx [file pone.0248681.s006.docx]

**Preliminary report on the bone histology of *Lystrosaurus* from the Lower Triassic of Xinjiang, China, and its implication for lifestyle and environments after the end-Permian extinction**

Fenglu Han^1*^, Qi Zhao^2^, Jun Liu^2,3, 4*^

^1^ School of Earth Sciences, China University of Geosciences (Wuhan), Wuhan, China

^2^ Key Laboratory of Vertebrate Evolution and Human Origins of Chinese Academy of Sciences, Institute of Vertebrate Paleontology and Paleoanthropology, Chinese Academy of Sciences, Beijing, China

3 CAS Center for Excellence in Life and Paleoenvironment, Beijing 100044, China;

4 College of Earth and Planetary Sciences, University of Chinese Academy of Sciences Beijing 100049

* Corresponding author

Email: [hanfl@cug.edu.cn](mailto:hanfl@cug.edu.cn) (FH); [liujun@ivpp.ac.cn](mailto:liujun@ivpp.ac.cn) (JL)

**Abstract**

*Lystrosaurus* represents one of the most successful dicynodonts, a survivor of the end-Permian mass extinction that remained abundant in the Early Triassic, but many aspects of its paleobiology are still controversial. The bone histology of *Lystrosaurus* species from South Africa and India has provided important information on their growth strategy and lifestyle, but until recently no data was available on the bone histology of *Lystrosaurus* from China. Here, we report on the bone microstructure of seven *Lystrosaurus* individuals from the Lower Triassic of Xinjiang, providing the first such data for the Chinese *Lystrosaurus* species. Our samples suggest that the microstructure of *Lystrosaurus* limb bones from China is characterized by fibrolamellar bone tissue similar to those from South Africa and India. Three ontogenetic stages were identified: juvenile, subadult, and adult based on lines of arrested growth (LAGs) and bone tissue changes. Bone histology supports a rapid growth strategy for *Lystrosaurus* during early ontogeny. Unlike Early Triassic *Lystrosaurus* from South Africa, lines of arrested growth are common in our specimens, suggesting interruptions in growth in *Lystrosaurus* from China. The differences in bone histology between *Lystrosaurus* from South Africa and China may indicate different environmental conditions in these two regions.

**Introduction**

The extinction at the end of the Permian was the most influential event in animal history since the Cambrian explosion. About 80% of all marine species and 50-70% of terrestrial families went extinct [1-3]. Recent studies suggest that the Permian-Triassic transition occurred in an increasingly hot and arid environment, disruptive to life [4-8]. However, some organisms evolved new survival strategies, such as miniaturization or rapid propagation [9-11].

*Lystrosaurus* is a medium to large-sized dicynodont therapsid with a strongly anteroventrally angled snout. It represents one of the most successful vertebrates that survived the end-Permian mass extinction, showing a cosmopolitan distribution during the Early Triassic [12-16]. However, the survival strategies of *Lystrosaurus* during the extinction are still controversial [17, 18]. Histological study of *Lystrosaurus* from South Africa has been used to support an aquatic or at least semi-aquatic lifestyle for this taxon, which could have helped it to escape the harsh terrestrial environment [19, 20]. There is also evidence that *Lystrosaurus* was a burrow maker [21, 22], and burrowing can help an organism survive harsh environmental conditions, as temperature and humidity are generally more stable underground than on the surface [23, 24]. Also, underground burrows can provide better protection for young, which is helpful for propagating the species [25].

Interestingly, the body size of *Lystrosaurus* from South Africa is smaller in the Early Triassic than in the late Permian [17], suggesting miniaturization. Furthermore, LAGs are more common in Permian *Lystrosaurus*, indicating a subadult or adult stage, whereas they are very rare from Early Triassic *Lystrosaurus*, suggesting that they were breeding young to compensate for dying at an early age [18].

Bone histology can provide important information on the life history of *Lystrosaurus*, which may bear on its survival during the end-Permian mass extinction. Previous studies have focused on the *Lystrosaurus* record from South Africa and India [19, 20, 26, 27]. However, *Lystrosaurus* seems to have been cosmopolitan in distribution [14], and is also very abundant in the Juggar and Turpan basins of Xinjiang, China. In China, seven *Lystrosaurus* species have been named since 1935, but the validity of most of these need re-evaluation [28, 29], and it is possible that only two species (*L. hedini* and *L. youngi*) are valid [30]. Here, we present data on the bone histology of *Lystrosaurus* from the Lower Triassic Turpan Basin, Xinjiang Province, China for the first time, using this data to comment on the survival strategy of this taxon.

**Material and methods**

Seven specimens of *Lystrosaurus* from the collections of the Institute of Vertebrate Paleontology and Paleoanthropology (IVPP) were selected for histological analysis. All these materials come from the Lower Triassic Jiucaiyuan Formation of the Taodonggou area (Taoshuyuan), Turpan Basin, Xinjiang, China [31, 32]. The age of Jiucaiyuan Formation in this area is middle-upper Induan based on previous chronostratigraphy [31, 33]. Identification of these specimens to species level is currently not possible, due to poor preservation or incomplete preparation. Bone histology in the four *Lystrosaurus* species from the Lower Triassic of South Africa is structurally similar [27, 34], although more LAGs are present in South African *Lystrosaurus* from the late Permian, which may indicate differences in environment [18]. Therefore, here we suppose that *Lystrosaurus* from the Taodonggou area of Xinjiang China should have identical morphology on bone histology even in different species. However, they may have distinct bone histological information from other places due to different environmental conditions [20]

Sections were sampled from the mid-diaphysis of limb bones including the tibia, femur, humerus, ulna, and radius. Limb diaphyseal sections were chosen because they are the primary centers of ossification and should preserve the most complete record of growth, and also minimize the potential of secondary remodeling overwriting the growth record (Fig S1; Table 1) [35, 36]. Rib sections in some individuals were also sampled for comparison. The preparation of the histological sections was carried out at the IVPP. The selected bones were processed using the Exakt-Cutting-Grinding System [37]. Sections were embedded in polyester resin, cut by EXAKT-300CP, and were ground to a thickness of about 50 μm with an EXAKT 400CS grinding machine. The polished sections were then observed under the transmitted and polarized light. The thin sections were observed using a Leica DM2700P microscope and photographed using a Leica DMC4500 camera. Nomenclature and definitions of structures follow [38, 39]. ‘Growth marks’ and ‘LAGs’ are both forms of growth lines, but the growth marks are identified to be LAGs only when the marked lines can be traced around the full circumference of the bone [35].

**Table 1 Skeletal elements of the *Lystrosaurus* specimens examined for histological sampling from the Early Triassic of Xinjiang, China.**

| Specimen number | Elements examined | Humeral length  (cm) | Radial length  (cm) | Femoral length  (cm) | Tibial length  (cm) | Length of fibulae  (cm) | Growth stages |
| --- | --- | --- | --- | --- | --- | --- | --- |
| IVPP V26543 | Tibia | —— | —— | 12.5 | 9 | —— | juvenile |
| IVPP V26544 | Humerus, Tibia, Fibula | 11 | 7.5 | —— | 9 | 9 | juvenile |
| IVPP V26542 | Femur | 11 | 7.5 | —— | —— | —— | subadult |
| IVPP V26545 | Fibula, Radius | 15.5 | 11^e^ | —— | —— | 11^e^ | subadult |
| IVPP V26546 | Femur | —— | —— | —— | —— | 12.5 | adult |
| IVPP V26547 | Femur | —— | 12^e^ | 17 | 12 | 12 | adult |
| IVPP V26548 | Rib | —— | 14 | 19 | 14 | 13.5 | adult |

^e^ denote estimated values .

Cortical porosity was measured using the software ImageJ 1.52a (https://imagej.nih.gov/ij/). It was difficult to obtain the cortical porosity of a whole cross-section for each specimen. Therefore, we subsampled areas (as many as ten) from the inner to the outermost cortex to calculate average vascular porosity. High-resolution images were acquired using a Leica DMC4500 camera. All the images were converted to 8-bit files, and the brightness/contrast was adjusted to clarify the structure. After that, we used “threshold” tools in ImageJ to select the vascular canals. Finally, the cortical porosity was determined using “analyze particles” tools in ImageJ. The average cortical thickness was calculated as the ratio of the cortex to the cross-sectional diameter of the bone. The medullary cavity is usually round or elliptical. To accommodate this variation, we initially drew a line representing the maximum observed distance within the medullary cavity. Then, we drew a line perpendicular to it. Along these two lines, we measured the cortical thickness and divided by the whole diameters. This value represents the minimum cortical thickness of each element.

**Results**

The bone histology of *Lystrosaurus* from North China shows a predominance of fibrolamellar bone tissue and a high cortical porosity (Figs 1-6), as in *Lystrosaurus* from South Africa and India [19]. The primary osteons are predominantly longitudinal, forming laminar or plexiform bone. Radially oriented primary osteons are also present in some parts of the cortex. Secondary reconstruction is common in the inner and mid-cortical bone in subadult and adult stages, resulting in enlarged channels and thin periosteal bone. The free medullary cavity is small and filled with bony trabeculae. Lines of arrested growth (LAGs) are common in the outer cortex of larger individuals.

All these elements could be divided into juvenile, subadult and adult stages based on bone tissue type and the presence of LAGs (**Table 2**). They are generally similar to the growth stages of *Lystrosaurus* from South Africa and India, but also have some features that differ from the latter.

**Table 2.** **Bone histological information of *Lystrosaurus* from China in each specimen**

| Specimen number | Skeletal elements | Cortical thickness /porosity (%) | Bone tissue type | Secondary reconstruction | Growth rings | Medullary cavity | Stages |
| --- | --- | --- | --- | --- | --- | --- | --- |
| IVPP V26543 | Tibia | 28/29 | plexiform and reticular | inner enlarged channels | 0 | a small free cavity | juvenile |
| IVPP V26544 | Humerus | 30/32 | plexiform | a few secondary osteons | 0 | a small free cavity | juvenile |
|  | Tibia | 24/27 | laminar and reticular | a few secondary osteons | 0 | full of trabeculae | juvenile |
|  | Fibula | 25/22 | laminar | a few secondary osteons | 0 | a large free cavity | juvenile |
| IVPP V26542 | femur | 27/38 | laminar and plexiform | extensive secondary reconstruction | 1 | a free medullary cavity | subadult |
| IVPP V26545 | fibula | 25/18 | laminar | a few secondary osteons | 1 | full of trabeculae | subadult |
|  | Radius | 26­/20 | laminar | a few secondary osteons | 1 | full of trabeculae | subadult |
| IVPP V26546 | femur | 15/24 | laminar | extensive secondary reconstrution | 3 | full of trabeculae | adult |
| IVPP V26547 | Femur | 25­­/22 | laminar | extensive secondary reconstruction | 4 | full of trabeculae | adult |
| IVPP V26548 | rib | / | laminar | extensive secondary reconstruction | >3 | full of trabeculae | adult |

**Juvenile**

The juvenile stage was represented by two samples: IVPP V 26543 and IVPP V 26544. All the cortices are characterized by a woven-fibered bone matrix, a high number of vascular canals, abundant globular osteocyte lacunae, and no LAGs, suggesting a rapid, constant growth rate. The mid-diaphyseal tibia of IVPP V 26543 is elliptical in cross-section, with a diameter of 12.5×14.3 mm and an average cortical thickness of about 28% (Fig 1). The free medullary cavity is small, with a diameter of about 4 mm. The entire cortex is comprised of woven fibered bone matrix and dense vascular canals (Fig 1). The cortex has a high cortical porosity (about 29%). The canals are predominantly longitudinal. A few irregular canals are shown in the inner and mid cortex (Fig 1A). Radial anastomoses form two narrow regions throughout the cortex, and one region has a concave periosteal surface (Fig 1B, C). The anastomoses join two or three adjacent canals in the outer cortex (Fig 1D) and link four to five canals forming long canals in the mid and inner cortex (Fig 1A). Most of the vascular channels show thin centripetal osteonal deposits forming primary osteons (Fig 1E, F). The primary osteons mainly show laminar and plexiform patterns in the outer and deeper cortex, and a row of wide-open canals are shown near the periphery (periosteal surface) in some areas (Fig 1D). Osteocyte density is consistently high throughout the cortex. Erosionally enlarged resorption cavities are present in the inner cortex (Fig 1A, C). There are no secondary osteons, LAGs, or endosteal bone. The medullary region contains cancellous bone, and a free small cavity is present.

**Fig 1. Bone microstructures in juvenile *Lystrosaurus* IVPP V26543.** **A**, partial cross-section showing fibrolamellar bone; **B**, the whole cross-section of the tibia; **C**, partial cross-section showing radial vascular canals; **D**, the outer cortex under normal light; **E**, enlargement of the outer cortex showing primary osteons under normal light; **F**, bone tissue of E under polarized light. **Abbreviation**: **rc**, radial canals; **wc**, wide-open canals;

In IVPP V26544, the tibia, humerus, and fibula were all sectioned (Fig 2). The cross-section of the tibia is elliptical in outline, with a diameter of 15×12 mm (Fig 2A). The cortical thickness is about 24%. There is no free medullary cavity. The cortex mainly consists of fibrolamellar bone tissues (Fig 2B), but parallel-fibered bone tissue is present in the medial region of the cortex articulated with the fibula (Fig 2C). Vascular canals are predominantly longitudinal. There are a few anastomoses that link two to five canals forming long straight and irregular canals. Most of the vascular canals are primary osteons forming a laminar pattern (Fig 2B). A thin layer (1 mm) of secondary osteons is present in the inner region of the compact bone (Fig 2D). The cortex has a high porosity of 27% (Fig 2A), but the diameter of vascular canals is slightly reduced near the periphery. Enlarged vascular canals are present in the inner cortex due to secondary remodeling. No LAG/annulus was detected in the cortex. The medullary region is full of trabeculae.

**Fig 2. Bone microstructures in juvenile *Lystrosaurus* IVPP V26544.** **A**, mid-diaphyseal cross-section of the tibia. **B**, bone microstructure of the tibia; **C**, partial cross-section of the tibia showing the parallel-fibered bone under polarized light; **D**, the enlargement inner region of B under crossed plane-polarized light showing secondary osteons; **E**, bone microstructure of the humerus under normal light; **F**. bone microstructure of the humerus under polarized light; **G**, the outermost cortex under normal light showing a growth mark (arrow); **I**, the same region as G under polarized light showing parallel-fibered bone; **H**, the inner cortex showing secondary osteons under normal light; **J**, the same region as H under polarized light; **K**, bone microstructure of the fibula under normal light; **L**, bone microstructure of the fibula under polarized light showing parallel-fibered bone; **M**, enlargement of L showing the organized osteocyte lacunae; **N**, the outermost cortex showing secondary osteons under normal light; **O**, innermost region showing endosteal bone under polarized light. **Abbreviation**: **end**, endosteal bone.

The humerus is subtriangular in cross-section (midshaft diameter: 21×25 mm) and more robust than the tibia (Fig 2E, F). It has a thick compact bone (at least 7 mm) with an average cortical thickness of about 30%, and the free medullary cavity is extremely small. The cortex is comprised of fibrolamellar bone tissue, but a thin layer of parallel-fibered bone tissue is present near the periphery (Fig 2F-I). The bone porosity has a value of about 32%, which is higher than the tibia. The primary osteons are predominantly plexiform and are longitudinally-oriented in the outer cortex. They are strongly reduced in size in the outer cortex. Parallel-fibered bone tissue and growth marks are present in the outermost region of the cortex (Fig 2G, I), suggesting slower growth rates when the individual died, but the osteocyte lacunae are still abundant. Many secondary osteons are seen in the inner cortex (Fig 2H-J). A thin layer of lamellar endosteal bone is deposited along the edges of the trabeculae in the medullary region (Fig 2F).

The fibula is much narrower than the tibia. The cross-section is oval in outline (midshaft diameter 6.5×4 mm) with a cortical thickness of about 25%. The medullary cavity is small with a diameter of about 2 mm. The cortex mainly consists of both fibrolamellar and parallel-fibered bone tissue (Fig 2K, L) and is less vascularized than the tibia and humerus, resulting in a lower bone porosity (22%). Osteocyte lacunae are abundant but are flattened and organized in the parallel-fibered bone (Fig 2M). The primary osteons are predominantly longitudinal and gradually decrease in size towards the periphery. Secondary osteons are mainly shown in the inner region, but there are also a few ones near the periphery (Fig 2N). A thin layer of endosteal bone is deposited in the innermost cortex (Fig 2O).

**Subadult**

The subadult stage was represented by IVPP V26542 and IVPP V26545. The cross-section of the femur of IVPP V26542 is elliptical with a diameter of 15×13 mm and an average cortical thickness of about 27% (Fig 3). The medullary cavity is large (the diameter is about 5 mm). The thickness of the cortex differs around the bone. The bone porosity is about 38% and the thickened region of the cortex has a higher porosity than that of the narrow region (Fig 3B). The cortex mainly consists of fibrolamellar bone and is highly vascularized. ­The primary osteons show a laminar pattern that is mainly longitudinally oriented, but radially-oriented channels are also shown in some places (Fig 3A). The number and diameter of the vascular canals include the porosity of the narrow region is significantly reduced near the periphery. The outer cortex consists of parallel-fibered bone matrix, and a LAG is present near the periphery (Fig 3C, D), suggesting reduced growth rates before it died. The secondary osteons are restricted in the innermost cortex (Fig 3E, F). Endosteal bone is present along the trabecular in the medullary cavity (Fig 3G).

**Fig 3. Bone microstructures of the femur in subadult *Lystrosaurus* IVPP V26542.** **A**, mid-diaphyseal transverse section of the femur; **B**, enlargement of A showing highly vascularized cortical porosity; **C**, enlargement of the outer cortex showing the LAG (arrow) under normal light; **D**, the same region as C, showing a LAG and parallel-fibered bone under polarized light; **E**, the inner cortex showing secondary osteons under normal light; **F**, the same region as E under polarized light; **G**, the medullary region showing endosteal bone. **Abbreviation**: **end**, endosteal bone; **so**, secondary osteons;

The cross-sections of the fibula, radius and rib of IVPP V26545 were sampled for observation. The cross-section of the fibula is subtriangular in outline (diameter: 12.4×9 mm) with an average cortical thickness of about 25% (Fig 4A, B). The cortex consists of fibrolamellar bone. Most of the vascular canals are longitudinally arranged circumferentially around the bone, forming primary osteons. They are abundant in the inner region, but the number and size decrease from the mid-region to the periphery, resulting in a low cortical porosity (18%). Secondary osteons are restricted to the inner cortex (Fig 4B), which consists of compact coarse cancellous bone (CCCB). A distinct LAG comprised of several growth marks is present near the mid-region of the cortex (Fig 4B). The medullary region is filled with trabeculae (Fig 4A).

**Fig 4. Bone microstructure in subadult *Lystrosaurus* IVPP V26545.** **A**, mid-diaphyseal transverse section of the fibula under normal light. The arrow denotes a LAG. **B**, partial outer cortex of the fibula showing the LAG consists of several growth marks (arrows). **C**, partial outer cortex of the radius under normal light. **D**, mid-cortex showing the LAG consists of several growth marks under polarized light (arrows); **E**, the same region as (D) under normal light. **F**, the whole cross-section of rib under normal light; **G,** enlargement of the outer cortex of the rib. The black arrow indicates LAGs. **Abbreviation**: **so**, secondary osteons.

The radius is more robust than the fibula, with an average cortical thickness of about 26%. The cross-section of the radius is elliptical with a diameter of about 6×5 mm (Fig 4C). The cortex mainly consists of woven fibered bone and has a cortical porosity of about 20%. The vascular canals are primarily longitudinal but there are also many irregular canals. Most of the vascular canals have formed primary osteons. Secondary osteons and erosional cavities are present in the inner cortex. A clear LAG, as well as growth marks, are present at the mid-region as in the fibula (Fig 4D, E). Outside the LAG to the periphery, the density of vascular canals is strongly reduced but still abundant (Fig 4C). The medullary cavity is large with a diameter of about one third that of the whole cross-section.

The cross-section of the rib is drop-shaped, with a diameter of about 13×7 mm (Fig 4F, G). The compact bone is very thin (the average thickness is about 0.6 mm) due to a highly remodeled inner and mid-cortex. The medullary cavity is filled with trabeculae. The cortex consists of parallel-fibered bone tissues with only a few vascular canals (Fig 4G). The osteocyte lacunae are still abundant and disorganized. A clear LAG is present, as in the fibula and radius.

**Adult**

The bone tissue of the femora of IVPP V26546 and IVPP V26547 shows the adult stage. In IVPP V26547, the midshaft of the femoral cortex is elliptical in outline with a diameter of 25×20 mm (Fig 5). The cortical thickness is about 25%. The cortical porosity is about 22% throughout, and the inner region is about 38% but is strongly reduced in the outer cortex (about 18.5%). There is no free medullary cavity. The cortex is composed of fibrolamellar bone throughout, and the outer cortex consists of parallel-fibered bone matrix with a thickness of about 1 mm (Fig 5). Primary osteons are predominantly longitudinal and abundant throughout the cortex except for the region near the LAGs and the outer cortex. The vascular canals are enlarged due to secondary reconstruction in the inner and mid regions. Two prominent LAGs are present in the mid-cortex (Fig 5D, E). The distance between these two LAGs is narrow (about 0.5 mm), and a thin layer of wide-open canals are present around the whole cross-section (Fig 5A, D). In the outermost region, the vascular canals are rare and nearly absent (Fig 5F), and two LAGs as well as growth marks are present near the periphery (Fig 5F-H). The osteocyte lacunae are flattened and organized along the growth lines (Fig 5H). The bone histology suggests that this individual had grown very slowly before it died, although it has no external fundamental system (EFS), which is present in modern mammals and some dinosaurs [36, 40].

**Fig 5. Bone microstructure of the femur in adult *Lystrosaurus* IVPP V26547.** **A**, mid diaphyseal cross-section. **B**, partial cross-section under normal light; **C**, The same cortex as B under crossed plane-polarized light. **D**, Enlargement of (B) under normal light, showing LAGs and parallel-fibered bone in the outer cortex. **E**. The same cortex as (C) under polarized light, showing LAGs and parallel-fibered bone. **F**, the outermost cortex under normal light showing LAGs and vascular canals; **G**, the same region as (F) showing parallel-fibered bone; **H**, enlargement of F showing LAGs, osteocyte lacunae and primary osteons. The arrows denote LAGs. **Abbreviation**: **wc,** wide canals

In IVPP V26546, the femoral cortex is elliptical in cross-section. It has a thin compact bone with an average cortical thickness of about 15% and a large medullary region (Fig 6A). The cortex consists of fibrolamellar bone matrix except for the periphery. The vascular canals are relatively small and longitudinally-oriented in the outer cortex (Fig 6B). The cortical porosity is about 24% and strongly reduced in the outer cortex. Secondary osteons are common throughout the cortex and are also shown near the periphery (Fig 6E, F). A LAG with a narrow layer of annuli is present in the mid-cortex (Fig 6B, C, G, H), and there are only a few vascular canals around. The osteocyte lacunae are flattened and organized within the annuli (Fig 6G, H). At least two LAGs are present near the periphery (Fig 6C, D). The parallel-fibered bone matrix with LAGs and rare vascular canals suggests that it grew slowly before it died.

**Fig 6.** **Bone microstructure in adult *Lystrosaurus* IVPP V26546 and IVPP V26548.** (A-H) IVPP V26546. **A**, whole cross-section; **B**, partial transverse section of the femur under normal light; **C**, the outer cortex under polarized light showing parallel-fibered bone and LAGs near the periphery; **D**, enlargement of the outer cortex showing LAGs; **E**, the outermost cortex showing secondary osteons under normal light; **F**, The same region as (E) under polarized light; **G**, enlargement of the annuli region showing the organized osteocyte lacunae; **H**, the same region as G under polarized light; (**I-J**) IVPP V26548. **F**, bone microstructure of the dorsal rib under normal light; **G**, enlargement of the outer region under normal light showing the LAGs. The arrows denote LAGs. **Abbreviation**: **anu**, annuli; **so**, secondary osteons.

In the largest specimen IVPP V26548, only one dorsal rib was sampled (Fig 6I). The cortex is strongly remodeled in the inner and mid-region, and even in the outermost cortex in some region. A thin layer of compact bone is shown in the outermost region. It is nearly avascular, with at least three LAGs near the periphery, which is similar to the EFS. However, these lines did not extend to the outermost region, and there are still several vascular canals, suggesting this individual was still growing when it died (Fig 6J).

**Discussion**

The bone tissue of *Lystrosaurus* consists of predominantly fibrolamellar bone with large channels, similar to those from Africa and India [19, 27], and three growth stages were identified: a fast-growing juvenile stage, a slower-growing subadult and an adult that grew very slowly. The fast growth rates may have helped them to adapt to the environment quickly [27]. The late juvenile was characterized by uninterrupted fibrolamellar bone tissue and the subadult was primarily fibrolamellar bone tissue interrupted by LAGs. The adult is nearly fully grown, which is characterized by the presence of parallel-fibered bone and closely spaced LAGs in the outermost cortex. Bone tissue type in *Lystrosaurus* is not only related to the growth stages but also differs between the various limb bones, including the femur, tibia, fibula and radius. The humerus, femur and tibia show a mainly laminar to plexiform vascular arrangement with a high cortical porosity in all stages, whereas the vascular canals of the fibula and radius are mostly longitudinally oriented and reduced in density, resulting in a low cortical porosity. In addition, our samples indicate that the humerus and tibia have higher cortical porosity than that of other limb bones, but the parallel-fibered bone with growth marks near the periphery appeared earlier in humerus than in other elements (IVPP V26544).

Ray et al. (2005) found a parallel-fibered bone in the outer cortex of a dorsal rib and a thin layer of lamellar bone in a vertebra at the adult stage, but they did not find a typical EFS with closely spaced LAGs near the periphery. In our samples, the parallel-fibered bone in the outer cortex appeared in the dorsal rib and other elements, representing the subadult stage here (Fig 4). Although there is no typical EFS detected, the presence of more LAGs and parallel-fibered bone tissue near the periphery suggests that it grew very slowly before it died. Considering that an EFS has been identified in other dicynodonts, such as in *Placerias* [41] and *Kannemeyeria* [42], *Lystrosaurus* may also have had an EFS. Botha et al. (2016) found that all Early Triassic *Lystrosaurus* from South Africa have no more than one observed LAG and no parallel-fibered bone in the outermost cortex, which suggests that they did not reach skeletal maturity [18]. However, LAGs seem to be more commonly distributed in *Lystrosaurus* from the Early Triassic of China. Within the seven individuals, three have more than one LAG and two have one LAG for each. The adult stage of *Lystrosaurus* from China has a femur of about 15 cm and tibia about 12 cm, which is larger than all the samples taken from Ray et al. (2005). The presence of LAGs and the large size of *Lystrosaurus* from China suggest that many *Lystrosaurus* reached the subadult stage, unlike Triassic *Lystrosaurus* from Africa where subadults are rare. This difference may be caused by differing environmental conditions in these areas. Although broadly connected on the Pangaean supercontinent, the Karoo Basin and North China were nonetheless extremely distant the Early Triassic, being located in mid-to-high latitudes of the southern and northern hemisphere, respectively [43]. The Karoo Basin has been inferred to be very arid in Early Triassic based on sedimentological evidence [6, 7, 34]. These harsh environmental conditions may have resulted in high mortality rates of *Lystrosaurus*, with most individuals dying during the early stage [18]. By comparison, the climate is inferred to have been semiarid-subhumid in the Early Triassic of North China [31, 44, 45], which may indicate better environmental conditions than that in South Africa.

Another important issue is whether the body size of *Lystrosaurus* was reduced in Early Triassic due to the harsh environment such as arid weather. Body size reduction has been shown in non-mammalian eutheriodont therapsids during the end-Permian mass extinction [10], and a reduction is also observed in *Lystrosaurus* from the Karoo Basin of South Africa, but this may the result of immaturity of the body based on bone microstructure [18]. Interestingly, the body size of *Lystrosaurus* from the lower section of the Guodikeng Formation, such as *L. youngi*, is smaller than in the upper section of Jiucaiyuan Formation, contrasting with the condition in South Africa [29]. This may indicate that the known materials of *L. youngi* represent immature individuals, whereas later Chinese *Lystrosaurus* from Early Triassic have reached the subadult or adult stage. However, the bone microstructure of *Lystrosaurus* from Guodikeng Formation is poorly preserved, and more material needs to be assessed in the future to confirm this hypothesis.

The lifestyle of *Lystrosaurus* has been argued to be aquatic/semiaquatic [19], fully terrestrial, or burrowing [46, 47]. In recent decades, this controversy has concentrated on aquatic or burrowing habits, which could benefit its survival in the arid environments of the Early Triassic. The aquatic/semiaquatic lifestyle was supported by the bone microstructure showing a thick cortex and porous vascular canals [19], and the linear discriminant analysis based on the compactness profile and body size parameters in extant animals [48, 49]. The cortical thicknesses of all samples are between 15-30% here, which is similar to *Lystrosaurus* from South Africa [19]. However, the dorsal ribs have dense erosional cavities in the medial and mid-cortex and a thin layer of compact outer cortex (Figs. 4, 6), which differs from the thick cortex (27-37%) in *Lystrosaurus* from Africa [19]. Therefore, the bone histology of the Chinese *Lystrosaurus* described here provides weak evidence for an amphibious lifestyle. A thick cortex has been reported in extant burrowing animals and nonmammalian therapsids [39], and juvenile *Lystrosaurus* has been found inside burrow casts indicating that they were active burrowers [21, 22]. Whether *Lystrosaurus* had a substantial aquatic component to its lifestyle in addition to burrowing could be elucidated by analyzing stable isotopes in the future. Previous studies suggest that aquatic and semi-aquatic vertebrates have δ^18^O values significantly lower than those of coexisting terrestrial animals [50-52]. Oxygen and calcium isotopes all proved spinosaurids to be aquatic or semiaquatic like modern crocodiles [53, 54], and they could also provide more evidence for the lifestyle of *Lystrosaurus*.

**Conclusion**

Here we have studied the bone histology of *Lystrosaurus* from the Early Triassic of China based on thin sections of seven individuals. The results indicate that *Lystrosaurus* grew rapidly in the early stages as previously shown in *Lystrosaurus* from South Africa and India. However, the presence of LAGs in the largest individual suggests that the growth of *Lystrosaurus* was interrupted in the late stage. The number of LAGs is rare in *Lystrosaurus* from South Africa but they are more abundant in the *Lystrosaurus* from Xinjiang, China. We propose that most *Lystrosaurus* from Xinjiang reached subadult and adult stages, whereas those from South Africa died during their juvenile stage. This distinction may be caused by different environmental conditions, but more samples from the Early Triassic of China are needed to test this hypothesis in the future.

**Acknowledgment**

We thank Zhang Shukang and Wu Rui for helping to make bone sections, Chu Daoliang for his helpful discussion. We thank Jennifer Botha, Megan Whitney, and Zoe Kulik for their comments on a previous edition of this paper.

**References**

1.	Maxwell WD. Permian and Early Triassic Extinction of Non-Marine Tetrapods. Palaeontology. 1992;35(3):571-83.

2.	Erwin DH. The Permo-Triassic extinction. Nature. 1994;367(6460):231-5.

3.	Stanley SM. Estimates of the magnitudes of major marine mass extinctions in earth history. Proceedings of the National Academy of Sciences. 2016;113(42):E6325-E34. doi: 10.1073/pnas.1613094113

4.	Retallack GJ. Permian and Triassic greenhouse crises. Gondwana Res. 2013;24(1):90-103. doi: org/10.1016/j.gr.2012.03.003.

5.	Sun Y, Joachimski MM, Wignall PB, Yan C, Chen Y, Jiang H, et al. Lethally hot temperatures during the Early Triassic greenhouse. Science. 2012;338(6105):366-70. doi: 10.1126/science.1224126.

6.	Smith RMH, Botha-Brink J. Anatomy of a mass extinction: Sedimentological and taphonomic evidence for drought-induced die-offs at the Permo-Triassic boundary in the main Karoo Basin, South Africa. Palaeogeogr Palaeoclimatol Palaeoecol. 2014;396(3):99–118.

7.	MacLeod KG, Quinton PC, Bassett DJ. Warming and increased aridity during the earliest Triassic in the Karoo Basin, South Africa. Geology. 2017;45(6):483-6. doi: 10.1130/G38957.1

8.	Bernardi M, Petti FM, Benton MJ. Tetrapod distribution and temperature rise during the Permian-Triassic mass extinction. Proceedings of the Royal Society B: Biological Sciences. 2018;285(1870):20172331. doi: doi:10.1098/rspb.2017.2331.

9.	Song H, Tong J. Mass Extinction and Survival during the Permian-Triassic Crisis. Earth Science. 2016;41(6):901-18.

10.	Huttenlocker AK. Body Size Reductions in Nonmammalian Eutheriodont Therapsids (Synapsida) during the End-Permian Mass Extinction. PLoS One. 2014;9(2):e87553. doi: 10.1371/journal.pone.0087553.

11.	Chu D, Tong J, Song H, Benton MJ, Song H, Yu J, et al. Lilliput effect in freshwater ostracods during the Permian–Triassic extinction. Palaeogeography, Palaeoclimatology, Palaeoecology. 2015;435:38-52. doi: <https://doi.org/10.1016/j.palaeo.2015.06.003>.

12.	Smith RM, Ward PD. Pattern of vertebrate extinctions across an event bed at the Permian-Triassic boundary in the Karoo Basin of South Africa. Geology. 2001;29(12):1147-50. doi: doi.org/10.1130/0091-7613(2001)029<1147:POVEAA>2.0.CO;2.

13.	Smith R, Botha J. The recovery of terrestrial vertebrate diversity in the South African Karoo Basin after the end-Permian extinction. C R Palevol. 2005;4(6-7):623-36. doi: doi.org/10.1016/j.crpv.2005.07.005.

14.	Colbert EH. The distribution of Lystros aurus in pangaea and its implications. Geobios. 1982;15:375-83. doi: <https://doi.org/10.1016/S0016-6995(82)80126-5>.

15.	Cosgriff JW, Hammer WR, Ryan WJ. The Pangaean Reptile, Lystrosaurus maccaigi, in the Lower Triassic of Antarctica. J Paleontol. 1982;56(2):371-85.

16.	King GM, Jenkins I. The dicynodont Lystrosaurus from the Upper Permian of Zambia: Evolutionary and stratigraphical implications. Palaeontology. 1997;40(1):149-56.

17.	Botha J, Smith RMH. *Lystrosaurus* species composition across the Permo-Triassic boundary in the Karoo Basin of South Africa: *Lystrosaurus* across the PTB. Lethaia. 2007;40(2):125-37. doi: <https://doi.org/10.1111/j.1502-3931.2007.00011.x>.

18.	Botha-Brink J, Codron D, Huttenlocker AK, Angielczyk KD, Ruta M. Breeding young as a survival strategy during earth’s greatest mass extinction. Sci Rep. 2016;6(1):24053. doi: 10.1038/srep24053.

19.	Ray S, Chinsamy A, Bandyopadhyay S. *Lystrosaurus murrayi* (therapsida, dicynodontia): bone histology, growth and lifestyle adaptations. Palaeontology. 2005;48(6):1169–85. doi: <https://doi.org/10.1111/j.1475-4983.2005.00513.x>.

20.	Ray S, Mukherjee D, Bandyopadhyay S. Growth patterns of fossil vertebrates as deduced from bone microstructure: case studies from India. J Biosci. 2009;34(5):661-72. doi: doi.org/10.1007/s12038-009-0055-x.

21.	Botha-Brink J. Burrowing in *Lystrosaurus*: preadaptation to a postextinction environment? J Vert Paleontol. 2017;37(5):e1365080. doi: 10.1080/02724634.2017.1365080.

22.	Modesto SP, Botha-Brink J. A burrow cast with *Lystrosaurus skeletal* remains from the Lower Triassic of South Africa. Palaios. 2010;25(4):274-81. doi: doi.org/10.2110/palo.2009.p09-077r.

23.	Tałanda M, DzięCioł S, Sulej T, NiedźWiedzki G. Vertebrate burrow system from the Upper Triassic of Poland. Palaios. 2011;26(2):99-105. doi: 10.2110/palo.2010.p10-024r.

24.	Hasiotis ST, Wellner RW, Martin AJ, Demko TM. Vertebrate Burrows from Triassic and Jurassic Continental Deposits of North America and Antarctica: Their Paleoenvironmental and Paleoecological Significance. Ichnos. 2004;11(1-2):103-24. doi: 10.1080/10420940490428760.

25.	Özkurt Ö, Yiğit N, Çolak E, Sözen M, Gharkheloo M. Observations on the Ecology, Reproduction and Behavior of *Spermophilus* Bennett, 1835 (Mammalia: Rodentia) in Turkey. Turk J Zool. 2005;29:91-9.

26.	Chinsamy A, Rubidge BS. Dicynodont (Therapsida) bone histology: phylogenetic and physiological implications. Palaeontol Afr. 1993;30:97-102.

27.	Botha-Brink J, Angielczyk KD. Do extraordinarily high growth rates in Permo-Triassic dicynodonts (Therapsida, Anomodontia) explain their success before and after the end-Permian extinction? Zool J Linn Soc. 2010;160(2):341-65. doi: doi.org/10.1111/j.1096-3642.2009.00601.x.

28.	Li J-L. *Lystrosaurus* of China. Vertebr Palasiat. 1988;26(4):241-9.

29.	Liu J, Li J, Cheng Z. The *Lystrosaurus* fossils from Xinjiang and their bearing on the terrestrial Permian Triassic boundary. Vertebr Palasiat. 2002;40(4):267-75.

30.	Camp J, Liu J, editors. The taxonomy and cranial morphology of Chinese *Lystrosaurus* 71nd Annual Meeting of the Society of Vertebrate Paleontology; 2011 Nov 5; Paris Las Vegas Hotel, Las Vegas, NV.

31.	Yang W, Feng Q, Liu Y, Tabor N, Miggins D, Crowley JL, et al. Depositional environments and cyclo- and chronostratigraphy of uppermost Carboniferous–Lower Triassic fluvial–lacustrine deposits, southern Bogda Mountains, NW China — A terrestrial paleoclimatic record of mid-latitude NE Pangea. Global Planet Change. 2010;73(1):15-113. doi: <https://doi.org/10.1016/j.gloplacha.2010.03.008>.

32.	Thomas SG, Tabor NJ, Yang W, Myers TS, Yang Y, Wang D. Palaeosol stratigraphy across the Permian–Triassic boundary, Bogda Mountains, NW China: Implications for palaeoenvironmental transition through earth's largest mass extinction. Palaeogeography, Palaeoclimatology, Palaeoecology. 2011;308(1):41-64. doi: <https://doi.org/10.1016/j.palaeo.2010.10.037>.

33.	Wan M, Yang W, Wang JJRoP, Palynology. A new Protophyllocladoxylon wood from the Induan (Lower Triassic) Jiucaiyuan Formation in the Turpan–Hami Basin, southern Bogda Mountains, northwestern China. 2019;267:62-72.

34.	Botha J, Huttenlocker AK, Smith RMH, Prevec R, Viglietti P, Modesto SP. New geochemical and palaeontological data from the Permian-Triassic boundary in the South African Karoo Basin test the synchronicity of terrestrial and marine extinctions. Palaeogeography, Palaeoclimatology, Palaeoecology. 2020;540:109467. doi: <https://doi.org/10.1016/j.palaeo.2019.109467>.

35.	Horner JR, de Ricqlès A, Padian K. Variation in dinosaur skeletochronology indicators: implications for age assessment and physiology. Paleobiology. 1999;25(3):295-304. Epub 2016/04/08. doi: 10.1017/S0094837300021308.

36.	Chinsamy-Turan A. The microstructure of dinosaur bone. Baltimore: Johns Hopkins University Press; 2005. 195 p.

37.	Donath K, Rohrer M. Bone Sectioning Using the Exakt System. Handbook of Histology Methods for Bone and Cartilage: Springer; 2003. p. 243-52.

38.	Francillon-Viellot H, de Buffrénil V, Castanet J, Géraudie J, Meunier FJ, Dire JY, et al. Microstructure and mineralization of vertebrate skeletal tissues. In: Carter JG, editor. Skeletal biomineralization: patterns, processes and evolutionary trends. New York: Van Nostrand Reinhold; 1990. p. 471-530.

39.	Chinsamy-Turan A. The microstructure of bones and teeth of nonmammalian therapsids. In: Chinsamy-Turan A, editor. Forerunners of mammals: radiation, histology, biology. Bloomington, Indiana: Indiana University Press; 2012. p. 65-90.

40.	Horner JR, De Ricqles A, Padian K. Long bone histology of the hadrosaurid dinosaur *Maiasaura peeblesorum*: Growth dynamics and physiology based on an ontogenetic series of skeletal elements. J Vert Paleontol. 2000;20(1):115-29. doi: [https://doi.org/10.1671/0272-4634(2000)020[0115:LBHOTH]2.0.CO;2](https://doi.org/10.1671/0272-4634(2000)020%5b0115:LBHOTH%5d2.0.CO;2). PubMed PMID: ISI:000166943200010.

41.	Green JL, Schweitzer MH, Lamm ET. Limb bone histology and growth in *Placerias hesternus* (Therapsida: Anomodontia) from the Upper Triassic of North America. Palaeontology. 2010;53(2):347-64. doi: <https://doi.org/10.1111/j.1475-4983.2010.00944.x>.

42.	Ray S, Botha-Brink J, Chinsamy-Turan AJFoM. Dicynodont growth dynamics and lifestyle adaptations. In: Chinsamy-Turan A, editor. The forerunners of mammals: radiation, histology and biology. Bloomington: Indiana University Press; 2012. p. 121-48.

43.	Muttoni G, Gaetani M, Kent DV, Sciunnach D, Angiolini L, Berra F, et al. Opening of the Neo-Tethys Ocean and the Pangea B to Pangea A transformation during the Permian. GeoArabia. 2009;14(4):17-48.

44.	Liu D, Zhang C, Yang D, Pan Z, Kong X, Huang Z, et al. Petrography and geochemistry of the Lopingian (upper Permian)-Lower Triassic strata in the southern Junggar and Turpan basins, NW China: implications for weathering, provenance, and palaeogeography. Int Geol Rev. 2019;61(8):1016-36. doi: 10.1080/00206814.2018.1490932.

45.	Tabor NJ, Sidor CA, Smith RMH, Nesbitt SJ, Angielczyk KD. Paleosols of the Permian-Triassic: proxies for rainfall, climate change and major changes in terrestrial tetrapod diversity. J Vert Paleontol. 2017;37(sup1):240-53. doi: 10.1080/02724634.2017.1415211.

46.	King GM, Cluver MA. The aquatic *Lystrosaurus*: An alternative lifestyle. Hist Biol. 1991;4(3-4):323-41. doi: doi.org/10.1080/08912969009386548.

47.	King GM. The aquatic *Lystrosaurus*: a palaeontological myth. Hist Biol. 1991;4(3-4):285-321.

48.	Germain D, Laurin M. Microanatomy of the radius and lifestyle in amniotes (Vertebrata, Tetrapoda). Zool Scr. 2005;34(4):335-50. doi: 10.1111/j.1463-6409.2005.00198.x.

49.	Canoville A, Laurin M. Evolution of humeral microanatomy and lifestyle in amniotes, and some comments on palaeobiological inferences. Biol J Linn Soc. 2010;100(2):384-406. doi: 10.1111/j.1095-8312.2010.01431.x.

50.	Bocherens H, Koch PL, Mariotti A, Geraads D, Jaeger J-J. Isotopic biogeochemistry (^13^ C, ^18^ O) of mammalian enamel from African Pleistocene hominid sites. Palaios. 1996;11(4):306-18.

51.	Fricke HC, Rogers RRJG. Multiple taxon–multiple locality approach to providing oxygen isotope evidence for warm-blooded theropod dinosaurs. 2000;28(9):799-802.

52.	Clementz MT, Holroyd PA, Koch PL. Identifying aquatic habits of herbivorous mammals through stable isotope analysis. Palaios. 2008;23(9):574-85.

53.	Hassler A, Martin J, Amiot R, Tacail T, Godet FA, Allain R, et al. Calcium isotopes offer clues on resource partitioning among Cretaceous predatory dinosaurs. Proceedings of the Royal Society B: Biological Sciences. 2018;285(1876):20180197. doi: doi.org/10.1098/rspb.2018.0197.

54.	Amiot R, Buffetaut E, Lécuyer C, Wang X, Boudad L, Ding Z, et al. Oxygen isotope evidence for semi-aquatic habits among spinosaurid theropods. Geology. 2010;38(2):139-42. doi: <https://doi.org/10.1130/G30402.1>.

**Supporting information**

**S1 Fig. The accurate sampled positions of all specimens in this study.** **A**. IVPP V26543, arrow denotes the sampled position of the tibia; **B, C**. IVPP V26544. B, the sampled positions of the tibia and fibula; C, the sampled position of the humerus; **D**, IVPP V26542, the distal end of the femur that sampled; **E-G.** IVPP V26545. E, The sampled position of the radius; F, the sampled position of the fibula; G. The sampled position of the rib; H. IVPP V26547, arrow denotes the sampled position of the femur; **I**, IVPP V26546, arrow denotes the sampled position of the femur; **J**. IVPP V26548, arrow denotes the sampled position of the rib.
