# Supplementary material for: Preliminary bone histological analysis of Lystrosaurus (Therapsida: Dicynodontia) from the Lower Triassic of North China, and its implication for lifestyle and environments after the end-Permian extinction
Source: PLoS One. 2021 Mar 18;16(3):e0248681. doi: 10.1371/journal.pone.0248681 (PMC7971864; doi:10.1371/journal.pone.0248681)
Supplement: Attachment — Submitted filename: Response to Reviewers.docx [file pone.0248681.s008.docx]

Dear Editor and Reviewers:

Thank you for your letter and for the reviewers’ comments concerning our manuscript entitled “Bone histology of *Lystrosaurus* (Therapsida: Dicynodontia) from the Lower Triassic of North China, and its implication for lifestyle and environments after the end-Permian extinction” (ID: PONE-D-20-17684). Those comments are all valuable and very helpful for revising and improving our paper, as well as important for highlighting the significance of our work. We have studied the comments carefully and made a number of corrections to address them, which we hope to meet with approval. The revised portions of the MS were marked by red colors. The main corrections to the paper and the responses to the editor and reviewer’s comments are as following:

**Editor comments:**

Please pay detailed attention to the comments made by ALL reviewers but specifically to the comments and criticism of Reviewer 4. For this manuscript to be acceptable for publication in Plos One, it is essential that you provide additional comparative data with regards to South African *Lystrosaurus* specimens as well as reconsider the interpretation of your own data, specifically with respect to the evidence for an external fundamental system (EFS), which is rather unconvincing based on the currently provided data.

**REPLY:** We have modified our MS carefully according to all reviewers’ comments. We have also added more comparisons between Chinese *Lystrosaurus* and South African *Lystrosaurus*, including both bone microstructure and body size. In addition, We have deleted the statement of the possible presence of EFS in *Lystrosaurus* based on current evidence, and we just mentioned that “there is still no typical EFS found in *Lystrosaurus*”.

**Reviewer1**

introduction line 4: It might be good to note that there is some debate about this, at least for the extinction in the Karoo Basin, such as Gastaldo et al. (2020; Journal of Sedimentary Research) and references cited therein

REPLY: We have added the debate and related references in the MS.

page 3 line 2: instead of curve, to would recommend using downturned, since the shape is more angular than curved in most *Lystrosaurus* species.

REPLY: Corrected.

page 3 line 3: The report of *Lystrosaurus* in Zambia is incorrect (See Angielczyk et al. 2014; Early Evolutionary History of the Synapsida book), so I recommend not citing the King and Jenkins paper. Frobisch (2009; Earth-Science Reviews) would be a more comprehensive paper to cite here (although, it still includes the incorrect Zambian record).

REPLY: We have changed the citation as suggested.

page 3 line 6: It might be worth noting that the semi-aquatic ecology of *Lystrosaurus* has been disputed including using bone histology data (e.g., King 1991; King and Cluver 1991, both Historical Biology; Botha-Brink and Angielczyk 2010, ZJLS).

REPLY: We have added more citations here.

page 3, paragraph 2: A couple comments here. 1) remove ‘the’ before minaturization. 2) I suggest a little re-phrasing of the results from the Botha-Brink et al. (2016) paper. That paper did indicate that LAGs were more common in Permian specimens, which was interpreted as evidence that the Permian species had a more extended ontogeny that extended over several years. This contrasts with the prevalence of small individuals with few or no LAGs in the Triassic species, which was part of the evidence suggesting breeding young (the other part of the evidence were the demographic simulations that suggested that breeding young could be an effective survival strategy when life expectancy was low).

REPLY: Modified as suggested.

material and methods line 6: I’m not sure if the specimens you sectioned had associated skulls. If they do not, I recommend saying that ID to species level is currently not possible because Chinese Lystrosaurus taxonomy (and Lystrosaurus taxonomy in general) is currently based on skull characters. If they have skulls, you might say that you didn’t try to ID them to species level given the uncertainties about Chinese Lystrosaurus species-level taxonomy that you mentioned above.

REPLY: Thank you for your suggestion. Our specimens only include postcranial materials. Modified as suggested.

material and methods line 8: Morphology refers to macroscopic features, whereas histology refers to microscopic tissue structure. So you should say: “...South Africa is quite similar, although more LAGs...” You should also make this change in the next sentence when you make your predictions about the Chinese species.

REPLY: Corrected. Furthermore, we moved this part to the discussion as suggested by another reviewer.

material and methods line 10: Identical histology might be too strong. Similar is probably a better prediction, assuming there aren’t differences due to environmental conditions (as you note).
REPLY: Modified as suggested.

Materials and methods, paragraph 2, line 3: complete instead of completed; also at the end of this sentence, change bones to bone. It would be good to note whether and how the specimens were documented before sectioning (e.g., e.g., if photographs or measurments were taken, if molds/casts were made, etc.).

REPLY: We have added this information in paragraph 2.

Table 1: How did you assign your specimens to growth stages? Is this something that was done a priori, and if so what was it based on (Size? Degree of ossification and development of muscle scars and articular surfaces? Something else?). You should include this information in the methods section.

REPLY: Growth stages were identified based on bone histological information, and we have added this information in the footnotes of Table 1.

Page 6, paragraph 1: This paragraph uses a mixture of present tense and past tense. Please revise it so it consistently uses past tense.

REPLY: Corrected.

Page 6, line 2-3: You should state why it was difficult to measure cortical porosity for a whole cross section. Was it because of breakage, differential preservation of the bone tissue in different areas, something else? Also, It might be good to elaborate a bit on how you chose areas to measure when that was necessary, maybe the average number of areas you needed to use (in addition to the maximum you report), and whether using different numbers of areas had any effect on the results.

REPLY: We have added this information and modified the sentence as “It was difficult to obtain the cortical porosity of a whole cross-section for each specimen because of partially breakage or poor preservation in some areas of these sections.”

Cortical porosity is usually reduced from the inner region to outside, but they are usually (not always) similar on all sides (medial, lateral, anterior, posterior). We subsampled ten areas from the inner to the outermost cortex in all sections (except IVPP V26546 was selected 12 areas) to calculate average vascular porosity. The sampled areas were chosen from the inner region to the periphery on different parts of the whole cross-sections, so they could reflect the value of cortical porosity. However, some bone sections show unclear bone histology in some regions, which may affect the results. Cortical porosities of those poorly preserved bone sections were labeled to be “estimated values”. We have provided the figures showing the sampled area (S3 Fig).

Fig. 1: I recommend reorganizing this figure slightly so that the overview of the specimen showing the focal areas is in panel A (similar to Fig. 2).

REPLY: Modified as suggested.

juvenile line 2: change to: All the corticies are composed of a woven-fibered bone matrix, with a…

REPLY: Corrected. We have modified this sentence as “All the cortices are composed of fibrolamellar tissue, with a high number of vascular canals, abundant globular osteocyte lacunae, and no LAGs, suggesting a rapid, constant growth rate.”

juvenile, line 5: when you mention cortical thickness and porosity values here and elsewhere in the text, you should call out Table 1. It would also be good if you can be more consistent in mentioning those values for the different elements in the text.

REPLY: We have added this information throughout the manuscript.

Fig. 2 and Fig. 2 caption: H and I should be switched in the figure in the caption given that you discuss what is currently I (but should be H) first. Also, panel O is a little dark. Can you adjust the brightness a bit on the image?

REPLY: Modified as suggested.


Fig. 3: Please add boxes to the overview image in panel A to show where the higher magnification images are located.

REPLY: Added.

Page 13, line 3: I think you mean: “The number and diameters of the vascular canals in the narrow region...”

REPLY: Thank you for pointing out. Corrected.

Fig. 5 : Please add boxes to the overview image in panel A to show where the higher magnification images are located.

REPLY: Added.

Page 16, line 2: An EFS was reported in the dicynodont Placerias by Green et al. (2010; Palaeontology).

REPLY: We have added more references here.

Fig. 6 : Please add boxes to the overview image in panel A to show where the higher magnification images are located.

REPLY: We have added a box in panel A, showing the position of B, and also a box in panel B, showing the position of C.

page 18, first full paragraph, line 7: Bone histology has been looked in a pretty wide range of dicynodonts now, and an EFS has only been observed in a couple of instances. Therefore, it seems odd to suggest that it may be present in Lystrosaurus, especially when you didn’t find any evidence of one in any of your specimens.

REPLY: We have modified this paragraph and conclusion here. The modified statement is “There is still no typical EFS detected in *Lystrosaurus*.”

Page 19, line 9: As noted above, some research has suggested wetter conditions in the Karoo at this time. It would be good to note that possibility here as well.

REPLY: we have added new references on wetter conditions.

Page 19, last line: It might be good to qualify this a bit by stating that most collected Early Triassic specimens are at least subadults. My sense is that *Lystrosaurus* hasn’t been documented in a comprehensive enough way to have an unbiased sense of the relative abundances of different size classes.

REPLY: Modified as suggested.

Page 20, first full paragraph: The other thing that’s important to note here is that the bone microstructure of *Lystrosaurus* doesn’t seem to differ all that much from other dicynodonts (e.g., Botha-Brink and Angielczyk 2010). So either that would imply that most dicynodonts were semi-aquatic or that *Lystrosaurus* was terrestrial like other dicynodonts (with dicynodonts in general showing some fairly consistent differences in microstructure to modern terrestrial tetrapods).

REPLY: Our samples support *Lystrosaurus* has a thick cortex, as in *Lystrosaurus* of Africa. But thick cortex is not only seen in aquatic animals but also shown in extant burrowing animals. Therefore, thick cortex could not provide confident evidence of the aquatic or semiaquatic lifestyle of *Lystrosaurus*, so we did not discuss the lifestyle of other dicynodonts. We have provided more discussion on *Lystrosaurus* lifestyle and how to solve this problem.

Page 20, first full paragraph, next to last sentence: It would be good to cite Rey et al. (2020; Palaeo3) here since it is a study that uses stable isotope data to make inferences about habitat preferences of a diynodont.

REPLY: Thank you for the information. Cited.

Reviewer 2.

There are some critical flaws that I think could only be addressed with an adequate comparative dataset of Permian *Lystrosaurus* specimens, and it's unclear to me whether the research team has adequately surveyed Permo-Triassic therapsid histology at all. Based on the abundant material of Permo-Triassic *Lystrosaurus* that I've thin-sectioned and seen firsthand, the South African material is nearly identical to the Chinese specimens. This study is hindered by a lack of any comparative specimens, and would further benefit from a more quantitative scientific approach.

REPLY: Thank you for your suggestions. We have added more comparison to *Lystrosaurus* from South Africa on both bone histology and body sizes. We admit that our samples are limited, but they do show some interesting and clear features that unlike those of *Lystrosaurus* from South Africa, and we will provide more data on bone histology and other parts of Chinese *Lystrosaurus* in the future.

Reviewer #3: The frequent presence of LAGs in the Chinese specimens is interesting and well worth publishing. I have made a number of copy edits and comments on some unclear parts of the manuscript in the attached file, which should be addressed before this contribution can be accepted.

REPLY: Thank you for your comments and detailed corrections for the grammar in the MS. We have checked the whole text and corrected all these things. Here are our replies to the main comments.

Page 4 Line 72. “Therefore, here we suppose that *Lystrosaurus* from the Taodonggou area of Xinjiang China should have identical morphology on bone histology even in different species. However, they may have distinct bone histological information from other places due to different environmental conditions”

**Comment**: This statement is unclear. Why should they have identical morphology?

REPLY: we have changed the word “identical” to “similar”. And moved this part to the discussion section. Here we mean that the previous work on Africa *Lystrosaurus* shows similar bone microstructure in different species, and Chinese *Lystrosaurus* may also have similar bone microstructure even in different species.

Page 6 Line 98. “Therefore, we subsampled areas (as many as ten) from the inner to the outermost cortex to calculate average vascular porosity.”

**Comment**: What is the minimum?

REPLY: We subsampled ten areas in all the sections. We have provided the sampled areas on photographs in the new supplementary files (S3 Fig).

Page 11. Line 206 The number and diameter of the vascular canals in the narrow region is significantly reduced near the periphery.

COMMENT: This does not make sense to me.

REPLY: Thank you for pointing out. We have modified this sentence to “The number and diameter of the vascular canals are significantly reduced near the periphery.”

Page 12. Line 210 “Trabeculae? In the trabecular region?”

REPLY: Yes. We have corrected this word.

Page 19 Recent papers by Kevin Rey on South African oxygen isotope work in dicynodonts would be worth citing and possibly discussing here.

REPLY: Thank you for your useful information. We have added this new reference here.

Reviewer #4:

This study describes the bone histology of seven Early Triassic *Lystrosaurus* from Xinjiang, China. The authors describe an ontogenetic series of rapid growth, with some periods of interrupted growth, and eventually a slowed, adult-stage growth. The authors compare these findings to those from the bone histology of South African *Lystrosaurus* and detail that although signals of arrested growth are rarely reported from South Africa, they were found in their sample. This suggests that perhaps the samples from South Africa are still in the juvenile/subadult stage. The authors suggest that this finding could be explained by environmental conditions that are thought to have been more extreme in South Africa than in China during the Early Triassic resulting in a greater die-off of individuals before skeletal maturity in South Africa. The authors also discuss their findings as it relates to the lifestyle of *Lystrosaurus*, refuting previous hypotheses that the genus was aquatic/semiaquatic.

I have reviewed a previous version of this manuscript and found the grammar and organization to be much improved. There were still some line edits of which I have added to my marked version of the manuscript. I have also added comments that are both specific and general to this document, but in addition, I have a few general comments about the manuscript. I think the figures are incredibly well presented and it is important this data is published. However, I think there are very major revisions in the discussion points/conclusions as well as some methodology that requires attention prior to publication.

Generally, I think the authors need to make a more compelling argument that their sample represents an ontogenetic series, independent of the histology. Table 1 I believe is supposed to address this, but the organization is difficult for the reader to follow, especially when I can’t compare a single element across the sample. To address this I think the authors, using more complete specimens and previously established ratios between elements, could come up with estimates of a single element for each individual in their sample. Alternatively, a single figure that images all of the samples at the same scale would help the reader confirm the authors’ designations of ontogenetic stages. I believe there is a supplementary figure, however, this figure was not included in my review document so I am unable to say if it would help to understand the relative sizes of these individuals. This is the only reason why I answered that not all data was available. In such a figure, I would also include images of the largest South African and Indian Lystrosaurus elements so that the reader can see just how much larger the Chinese specimens are.

REPLY: Thank you for your suggestions. Sorry for unmodified this part last time. The postcranial skeleton of *Lystrosaurus* was not well studied, and we did not know the variation between different species and ontogeny, so we did not estimate values previously. We have rechecked more *Lystrosaurus* materials and provided estimated values based on element ratios of one complete skeleton (IVPP RV35012), please see the footnotes of Table 1 for detail information.

In addition, sorry that we forgot to upload figure S1. In this figure, we have provided the accurate sampled positions of each element.

The methodology still requires significant revision/clarity. I would review previous histological papers that measure cortical thickness. I think for cortical thickness, presenting a range is more important that an average, especially in some specimens that are asymmetrically arranged. Cortical porosity also needs to be addressed. The description as it stands does not seem systematic and completely subjective. Subsampling vascularity is ok, but the locations (medially, laterally, posterior, anterior, endosteally, mid-cortex, periosteally) need to be systematically sampled similarly across the entire sample. Without this, it is difficult to review the values reported.

REPLY: We have provided ranges for all cortical thickness and labeled the measurement positions in S2 Fig.

We have added more descriptions of how to measure cortical porosity. We choose ten areas that cover all the cortex. The sampled areas were from the inner region to the periphery on different parts of the whole cross-sections, and it indicates the average cortical porosity of the whole cross-section. Some bone sections show unclear bone histology in some regions, which may affect the results. Cortical porosities of those poorly preserved bone sections were labeled to be “estimated values”. We have also provided the figures showing the areas that we choose. Please see S3 Fig.

In the descriptions differences in cortical porosity need to be explicitly stated. There are many instances where the percentage for one region (e.g. endosteal) of the cortex is reported and then the authors state there is a reduction in vascularity towards the periosteal surface, but no percentage is reported. Consistency is important in interpreting the descriptions.

REPLY: We have added more percentages to make it clear.

It is important that the authors describe the anatomical context for the images (i.e. medial, lateral sides etc.). This can be done in the image itself (e.g. compass-style arrows) or in the figure caption.

REPLY: We checked all the specimens again and have added the directions for all the figures.

Finally, I do not think the data here is sufficient to refute the hypothesis that Lystrosaurus was aquatic. I agree entirely that it is unlikely *Lystrosaurus* was not aquatic, however, I don’t believe the data as currently presented in this manuscript supports this. In fact, the thick cortical walls appear very similar to aquatic taxa, which is why previous studies have suggested an aquatic lifestyle. However, there are studies that demonstrate this is not always a reliable proxy for lifestyle (e.g. Houssaye et al. 2016). I would recommend one of two courses of action. Either remove the discussion of the aquatic lifestyle from this manuscript OR discuss how cortical thickness does not always accurately predict habitat.

REPLY: Sorry for the confusion. We did not refute the hypothesis that *Lystrosaurus* was aquatic. We prefer to keep this paragraph and have added more discussion here. Most of our samples support *Lystrosaurus* has a thick cortex, as in *Lystrosaurus* of Africa. But thick cortex is not only seen in aquatic animals but also shown in extant burrowing animals. Therefore, thick cortex could not provide confident evidence of the aquatic or semiaquatic lifestyle of *Lystrosaurus*. The lifestyle of *Lystrosaurus* is still controversial, but we provide more data for this issue.

**Other comments in the text**

Page 4 Line 9. Comment: This is an important discussion point, but should not be in the materials and methods section. Perhaps move to the discussion when reviewing the variation.

REPLY: We have moved this paragraph to the discussion part.

Page 5 Line 5. Comment: This is a confusing sentence. I think to simplify and to set up the descriptions nicely just describe the histological criteria used to assign a growth line (especially annuli, since you use that term later) vs. LAG.

REPLY: We have modified this sentence as “LAGs are growth marked lines that can be traced around the full circumference. The annuli correspond to periods of slow growth by the presence of parallel-fibered bone and flattened osteons”.

Page 5 Table 1. Comment: I think this table is really confusing. It's hard to compare the sizes between specimens since there are different elements. See overall notes for suggestions.

REPLY: We have added the estimated values as suggested.

Page 5 Table 1. Comment: If you are going to estimate values you have to describe how those estimations were made.

REPLY: We have added an explanation of how to estimate values in the footnotes.

Page 7 line 7. Comment: This is very confusing and I'm not convinced it properly measures cortical thickness. Generally, I don't actually think the "diameter" measurements given in each of the description sections are properly stated either. I would suggest reporting mediolateral and anteroposterior widths (which is basically what I think the "diameter" measurements are?). I believe given the variability in cortical thickness around the bone, reporting the average is not helpful. Instead, maybe report a max and min %?

REPLY: We have modified the MS as suggested.

P10 L4 comment: Label these in the figure.

REPLY: Labeled as suggested.

P10L5 There are no secondary osteons.

Comment: “It looks like there is in fact a massive one in 1C”

REPLY: we assigned it to be an enlarged resorption cavity rather than secondary osteons for its extremely large size. No Haversian system was presented around the erosion cavity.

P10 L6. This has already been described earlier in the paragraph

REPLY: we have deleted it.

P10 L11.

I feel that wide-open is too casual of a term. Instead I would go with something like "enlarged canals". You could also just highlight the band of expanded vascularity with a bracket like: |-----|

REPLY: “enlarged canals” usually indicate secondary remodeling in our manuscript. But here it should be the primary bone. Therefore, we just described its shape here. We have added a bracket as suggested.

P11 Line 16 The humerus is subtriangular in cross-section (midshaft diameter: 21×25 mm) and more robust than the tibia.

**Comment**: I don't know what this means. In what way is it more robust? Greater cortical thickness?

REPLY: “subtriangular” means triangular but less angled. We have shown more figures in supplementary files (S3 Fig). “robust” means the humerus bone has a larger cross-section. Modified as suggested.

Page 12 Line 9

Where within the cortex are these tissues found? How are they distributed throughout the cortex?

REPLY: We have modified this sentence as “The cortex mainly consists of fibrolamellar bone tissue, but parallel-fibered bone tissue is shown in the posterior cortex”.

Page 12 subadult line 3. “The medullary cavity is large (the diameter is about 5 mm)” comment: but not relative to the rest of the cross section...it may be large compared to the juvenile...but this is relative. It appears quite small to me in the figure.

REPLY: We have modified this sentence to “The medullary cavity is relatively large (the diameter is about 5 mm) compared to those of juvenile individuals”

P13. Line 2. What side of the bone is this on? It's a cool structure so some anatomical context would be appreciated.

REPLY: Added as suggested.

P14 Line 1. Also include mid-cortex and endosteal values. See general notes for more on this.

REPLY: We have added these values in the revised MS.

P14 Fig. 4 Where are D and E from in C? See general notes for further comments on this.

REPLY: we have added a box in C to show the position of D and E.

P14. Line 16. The vascular canals are primarily longitudinal but there are also many irregular canals

comments: Where? Label them in figure.

REPLY: We have added the labels in figure 4C.

P14 Line 18. Label these in C as well so there is context for D and E

REPLY: Added.

P15. Comments: The distinction between growth marks and LAGs should be clearly established in the methods section

REPLY: We have added their differences in the methods section. Growth marks and LAGs both form lines, but growth marks could not be traced on the whole circumference cortex bone.

P16 figure 5. **comment**: Indicate from where in A. See general notes for more details

REPLY: We have added a box in A showing the position of B.

P16 figure 5 wide-open canals.

REPLY: enlarged canals usually referred to secondary remodeling. Here we just describe its shape. It should represent primary bone.

P16 paragraph 3 line 3. Compared to what?

REPLY: We have modified this sentence to make it more clear.” The vascular canals are longitudinally-oriented and relatively small in the outer cortex than in the mid and inner cortices”.

P16 paragraph 3 line 5 Stay consistent in reporting percentages and report for all regions.

REPLY: We have added more percentages of cortical porosity in different parts of the cortex.

P16 paragraph 3 line 6 Also mention the LARGE one to the bottom of Fig. 6A.

REPLY: The large cavity to the bottom is a large erosional cavity surrounded by endosteal bone rather than secondary osteon because its size is much larger than other secondary osteons (See fig. S8). We have added the description of this large cavity.

P17 line 1.

Is this section truly from the mid-diaphysis? It looks to me like it's slightly towards the proximal or distal end which is fine, but do report it if that's the case. If it is in fact truly from the mid-diaphysis, the medullary cavity is much larger than in the other adult specimen and this difference should be discussed.

REPLY: Thank you for pointing out. The femur of IVPP V26546 that sampled only preserved the distal part. We rechecked this specimen and found that you are correct. The measurement of the preserved region is 5 cm, and the estimated whole length is about 14.6 cm. So this section was sampled near the distal region (See figure S1). We have added this information in the method and description part of the paper.

P19 Line 6.

Cite studies such as Köhler et al. 2012 that show LAGs are deposited even in fast growing animals in stressful enivornmental conditions.

REPLY: Cited as suggested.

P19 line13:

There are two discussion points missing from here I think. First, wouldn't the LAGs in the mid-cortex mean that Chinese *Lystrosaurus* was experiencing some kind of stressful season? Of anything, there is more indicators of stress in specimens from China than those from South Africa. I think if there is an argument to be made about the environment being more stressful in South Africa, you need more clarity on the size differences. If you can show that the South African specimens were smaller than your subadult specimens with LAGs then it would get around this issue. Second, I would move the section where species differences in bone tissue is discussed here and expand on differences that may be related to species-level differences.

REPLY: we have added more comparison on body size between Africa and Chinese *Lystrosaurus*, and pointed out that our individuals are larger than sampled Africa *Lystrosaurus* from Early Triassic. We have also added species differences discussion here, although only a little could be clarified here.

Page 19. Paragraph 2. Line5

Do you mean to say something more like, "an artifact of sampling only young individuals from South Africa"? This is not clearly written as is.

REPLY: Here we mean that *Lystrosaurus* from Early Triassic may die at a young age (indicated by fewer LAGs), so they are smaller than those from late Permian (should be at least subadult based on more LAGs). We have modified this sentence to “but this may the result of the young stage (indicates by rare LAGs) of the body in the Early Triassic based on bone microstructure”.

Page 19. Paragraph 2 line 6.

This formation has not been introduced. Describe it's age here for audience reference.

REPLY: Added as suggested.

Page 20 line 10.

Do you mean in the mid-cortex? This means something else in reference to the gross anatomy so I would avoid using it.

REPLY: We have modified this sentence to “---in the inner and mid cortex”

Page 20. Line 11. This jump is not logically laid out. You state a thick cortex and abundant canals suggests aquatic lifestyle. Then you state the cortical thickness (which I think needs clarification or remeasuring) but do not state whether that is thick or thin compared to other animals. You state that the ribs are different. And then you state that because of this, you can't say it's aquatic/semiaquatic. See general comments for further discussion.

REPLY: we have deleted this sentence and modified this paragraph properly to make it more clear. Most of our samples support *Lystrosaurus* has a thick cortex, as in *Lystrosaurus* of Africa. But thick cortex is not only seen in aquatic animals but also shown in extant burrowing animals. Therefore, thick cortex could not provide confident evidence of the aquatic or semiaquatic lifestyle of *Lystrosaurus*. The lifestyle of *Lystrosaurus* could not be certain right now, but we provided more methods to solve this problem.

PAGE 28

S1 Fig. I did not get this figure in my review document.

REPLY: Sorry. We have unloaded this figure in the revised version.

Figure 1. The guides in B to show were zoomed in photos are from are really helpful and necessary. I would suggest adding similar boxes to the rest of the figures for clarity.

REPLY: We have added boxes in other figures as suggested.

I would add anatomical directions (i.e. medial, lateral, posterior, anterior) in either the figure or the caption (e.g. medial to right, lateral to left, etc.). I would do this for all of the histology figures.

REPLY: Thank you for your suggestions. We have added anatomical directions in all the figures.

Sincerely,

Fenglu Han
