# Supplementary material for: Preliminary bone histological analysis of Lystrosaurus (Therapsida: Dicynodontia) from the Lower Triassic of North China, and its implication for lifestyle and environments after the end-Permian extinction
Source: PLoS One. 2021 Mar 18;16(3):e0248681. doi: 10.1371/journal.pone.0248681 (PMC7971864; doi:10.1371/journal.pone.0248681)
Supplement: Attachment — Submitted filename: Response to Reviewers R2.docx [file pone.0248681.s011.docx]

Jan. 6^th^, 2021

**Dear Editor and Reviewers:**

Thank you for your letter and for the reviewers’ comments concerning our manuscript entitled “Bone histology of *Lystrosaurus* (Therapsida: Dicynodontia) from the Lower Triassic of North China, and its implication for lifestyle and environments after the end-Permian extinction” (ID: PONE-D-20-17684). Those comments are all valuable and very helpful for revising and improving our paper, as well as important for highlighting the significance of our work. We have studied the comments carefully and made a number of corrections to address them, which we hope to meet with approval. The revised portions of the MS were marked by red colors. The main corrections to the paper and the responses to the editor and reviewer’s comments are as following:

**Reviewer #4**: I appreciate all of the work put into this revision and find it greatly improved. I believe the paper now clearly demonstrates methodology and provides sufficient details to warrant publication. Particularly, S2 and S3 figs. Are well presented figures! I find it very helpful and thorough. There are only a few minor line edits that I found that only related to grammar and clarity. I have attached these comments in a marked version of the PDF.

**REPLY:** Thank you for all your comments and corrections. All the minor comments in the pdf file have been corrected. In addition, the revised version has also been modified a lot based on another reviewer’s comments. For example, we have deleted some values of cortical thickness and cortical porosity that were thought to be objective, but we still keep S2 and S3 Figs that can show bone microstructure more clear. We also have added new comparisons between Chinese and South Africa *Lystrosaurus* based on the new published paper by Botha et al. (2020). We hope all these modifications are comfortable for you.

An additional comment refers to S2 fig. The title of the figure says "accurate". I'm not sure what that is in reference to. But it would be better to have the limbs similarly oriented and the view explicitly stated (e.g. anterior).

**REPLY:** I think you mean S1 Fig. We just pointed the positions that we sampled in the figure. We have removed the word “accurate” and added limb orientation as suggested.

**Reviewer #5**: This study describes the bone histology of seven *Lystrosaurus* individuals from the Lower Triassic of North China. The data have potential to provide interesting and new information on the growth patterns of *Lystrosauru*s as no studies have yet to focus on this taxon from China, potentially allowing for comparisons with *Lystrosaurus* from South Africa and Antarctica. However, given that the individuals could not be identified to species level (and thus there could be interspecific variation) I would call this study, at most, a preliminary work. They have very interesting conclusions suggesting that the *Lystrosaurus* from China are larger than those from the Triassic of South Africa and they lived longer (i.e. there are more LAGs in the Chinese specimens compared to the Triassic species from South Africa). This is the most interesting result of the study. However, I have some concerns relating to how much can be concluded from the data and these concerns should be addressed before the manuscript is ready for publication. My concerns are as follows.

1. Only three out of the seven individuals have more than one LAG – and the third element is a rib, which looks like it has a double LAG, not a triple LAG and certainly not an EFS. Double LAGs indicate one season (laid down in one year), so I would not call what you see in the rib as representing multiple seasons. That leaves the two femora – so only two individuals have three or four LAGs. I’m not convinced this is a large enough sample size to indicate that Chinese Triassic *Lystrosaurus* were growing for longer periods compared to South African Triassic species. At most this is a preliminary observation and more work needs to be done to see if you can replicate the data.

**REPLY**: We agree that our work is just a preliminary observation and much work needs to be done in the future, but we do not think the third element only has a double LAG that represents one year. The third element is a rib, which has three LAGs that were identified previously. We agree that only two prominent LAGs are shown in Fig. 6J, and the third outermost LAG is weak. At least, there are two LAGs present. We supposed it to be two LAGs rather than a double LAG, mainly based on the following evidence: these two LAGs are isolated, vary out of sync, and never combined; the related femur has more than 2 LAGs and a wide parallel-fibered bone at the periphery, which was sampled by another researcher and will be published elsewhere so that could not use in this paper, unfortunately. Furthermore, the LAGs are more closed to each other in the thin cortex (such as ribs, fibula, etc) than in the robust cortex (such as humerus, femur and tibia). For example, see the paper Han et al (2020, the bone histology of *Jeholosaurus*). The distinct between LAGs is much narrower in the fibula than that in the tibia. Finally, this specimen (sampled rib) is the largest individual that we sampled and it is reasonable that it has more LAGs than others.

2. The data used to compare the size of Chinese *Lystrosaurus* with South African *Lystrosaurus* is outdated. Sam-pk-k8 and Sam-pk-8991 do not represent fully grown specimens or the largest known specimens. You can look at Botha 2020 (Botha, J. 2020. The paleobiology and paleoecology of South African Lystrosaurus. PeerJ 8:e10408. DOI: doi.org/10.7717/peerj.10408.). Granted, this publication came out while this study was under review and thus the authors have not had the opportunity to compare the data – but now is the time to do so. There is skull data for the maximum sizes of each South African species as well as size data for the limb bones used – i.e. the authors compare the size of the humerus, this can be done with both Triassic South African *Lystrosaurus* species to see how much larger the Chinese species is – if they really are notably larger and this can be confirmed, then it is a very interesting result, but this needs to be more adequately confirmed.

**REPLY**: Thank you for your new information. We have added more comparison of the known humeral length with a LAG between Chinese *Lystrosaurus* and South Africa from Early Triassic, we found that their size has no significant difference (S4 Fig). But Chinese *Lystrosaurus* still has a relatively large proportion with LAGs compared to South Africa *Lystrosaurus*. The humeral length larger than 10 cm is the most common size based on our observation. Both the body size and bone microstructure support that many *Lystrosaurus* have reached at least the subadult stage, unlike Triassic *Lystrosaurus* from Africa where subadults are rare. Of Course, we need more work to clarify this conclusion.

3. I disagree with the last ontogenetic stage being labelled adult. An adult will have a significant amount of slower growing tissues (parallel-fibered or lamellar) and/or an EFS – neither of which is present in the largest individuals. There is the beginning of parallel-fibered bone, so what the authors have captured here is the beginning of slower growth which would make these individuals subadults. It also cannot be confirmed if what is seen in the rib is an overall decreased growth or just parallel-fibered bone associated with a double LAG after which growth may have increased again. The two femora are undoubtedly older than the other individuals, but they are not adult. You could perhaps divide the stages into juvenile, early subadult and late subadult – although even saying late subadult is a push because there is so little parallel-fibered bone.

**REPLY**: We have changed the word “adult” to “late subadult” stage as suggested.

4. I have a problem with the methodology in calculating the cortical thickness of the bones. The problem with most dicynodonts is that they do not have a sharp transition from a clear open medullary cavity to a compact cortex. There is generally, and this is seen in Lystrosaurus, a gradual transition zone and there are often trabeculae within the medullary cavity. So if you are measuring the compact bone manually as the authors have done here, where do you objectively draw the line between compact bone and medullary cavity? It is very difficult to standardize and objectively measure cortical thickness in this way. This is why the program Bone Profiler is better than a manual procedure because the computer measures so many transects all around the bone. I don’t think any of the cortical thickness measurements are useful if they are done manually (unless you have sharp transition zones, which you don’t in Lystrosaurus). These calculations cannot be compared with other Lystrosaurus specimens or with other taxa. However, using Bone Profiler to calculate cortical thickness (which is generally K in the program) is a lot of work as the cross-sections needs to be changed into black and white images, which can only be done manually by colouring in the bone black and the spaces white – this is extremely time consuming. I thus, suggest that the entire cortical thickness section be deleted. The values cannot be reliably used and they don’t say much about the lifestyle of Lystrosaurus anyway. If the authors wish to keep the cortical thickness in the paper then they need to use Bone Profiler to obtain their values.

**REPLY**: Thank you for your suggestions. In fact, we tried to use the software bone profiler after your suggestion in your first review. But we found many problems and we gave up finally. First, we did not find a valid link that could download this software. The official link provides a new version that analyses on the website but was also not available for analysis right now. In addition, I do not quite understand the principle of the software. All the bone has the same color, then how to separate compact bone and cancellous bone in the program? Girondot and Laurin (2003, p460, secondary paragraph) mentioned that K was the ratio between the internal and external diameter of the bone, so it should not represent the cortical thickness that we mentioned here.

Finally, we agree that a gradual transition from compact bone to medullary cavity has happened in *Lystrosaurus*, but not all specimens are difficult to separate. There is still a clear change in some specimens, such as the femur IVPP V26542. This situation is also mentioned by Ray et al (2005). So in the revised version, we keep the cortical thickness values that we can discern the transition clearly and have omitted other values with a vague transition between compact bone and medullary region. Furthermore, we have also omitted cortical porosity values with vague transitions.

5. There is also a problem with the methodology for calculating cortical porosity. Fig S3 shows where the fields of view were taken – A, B and C are ok, but in D Field Of View 8, 9 and 10 are taken from the inner regions of the cortex, some of which includes resorption cavities – these cannot be compared with primary vasculature – so 8, 9 and 10 would give you inflated values compared to the others. Even in FOV 1-4 the FOV includes enlarged cavities and not just primary vasculature – this again inflates the values compared to 5-7. FOV is generally taken from the mid-cortex only and all 10 FOVs are taken from the “same” place giving you an average over 10 FOV. One can do what was done in A, B and C because each FOV is the same. E is also a problem because some FOVs are from the outer cortex, some from the middle and some from the inner regions – so you’re not standardizing where you’re taken the FOV from. Yes, you’re getting an average of the bone, but you’re doing it in a different way each time. F and particularly G I’m worried about as the inner regions of the FOV are resorption cavities which you cannot include in primary vasculature – so here, the entire bone’s values are inflated, same with H – in fact in H, there is even medullary cavity, so was this included as space or was it ignored? If the latter then your FOV is smaller depending on where the FOV was taken, so you’re not comparing same-sized FOVs. This is going to produce unreliable results.

**REPLY:** We agree that there are still some problems with measuring cortical porosity. The best way is to measure the whole compact bone. However, there are at least two problems: As we mentioned above, there is no sharp transition between compact bone and medullary cavity in some specimens, and the areas that we sampled may be objective. Therefore, in the revised version, we only keep the cortical porosity values with clear tissue change. Secondly, some bone sections do not show clear bone tissue in some parts due to bad preservation, such as Fig S3 E. It has a clear medullary cavity, but not allowed us to standard the acute position. It just provides a reference, that is why we mentioned that it was an estimated value in the method. Nevertheless, we have omitted this value according to the reviewer’s comments.

6. It would also be good to include Whitney, M.R. and Sidor, C.A. 2020. Evidence of torpor in the tusks of *Lystrosaurus* from the Early Triassic of Antarctica. Communications Biology 3:471. DOI: 10.1038/s42003-020-01207-6. Although they do not deal with limb bones (they use teeth) they do talk about different environments affecting Lystrosaurus. This publication may have come out while this study was still in review – but it would be good to include it now for a discussion on differing environments affecting *Lystrosaurus* growth.

**REPLY**: We have added this reference and mentioned that environments can affect tusk and bone microstructure of *Lystrosaurus*. But we did not discuss a lot because it was difficult for comparison between teeth and bone microstructure.

**Comments in the pdf**

P5, line 10

**Comment:** you need to explain the difference between a LAG and an annulus. i.e. a LAG is a cement line and indicates a temporary, but complete cessation in growth whereas an annulus consists of lamellar or parallel-fibered bone and indicates a temporary decrease in growth rate

**REPLY**: We have added this information.

Table 1. Comment: fix this column so that the s is not on another line

**REPLY**: Modified as suggested.

P6, line 2

why was this specimen chosen? is it the largest known in China? is it the most complete? does it have a skull? can you identify it to species level? You say that the ontogenetic stage is unknown - but is it small or big? are sutures fused? are their muscle scars? etc you must be able to estimate the ontogenetic stage of this specimen. Are you estimating all these other values based on a juvnenile or an adult?

**REPLY:** we have added more information on this specimen in the method. It is the holotype of *L.* *hedini* and represents the most complete specimen of *Lystrosaurus* (Young 1935). It has a skull length of 173 mm and includes most of the postcranial material. It is probably a subadult individual based on open neurocentral sutures and fused sacral vertebrae. It has a humeral length of 11.5 cm, which is more suitable to assign it to be within the subadult stage compared to others.

P6, paragraph 1

Comment: This is an outdated method for calculating cortical thickness and is problematic in an animal such as *Lystrosaurus* because there is no clear strict transition between cortical bone and medullary cavity. The broad transition zone in this region makes measurements calculating cortical thickness quite subjective. I recommend using Bone Profiler for this method. These measurements will then be more useful in that they can be compared with other studies.

**REPLY**: We have already given a detailed explanation above. We have omitted controversial data but keep some measurements that have a clear transition. As we provided the accurate position that we measured (Fig. S2). The reader can choose whether to use it or not.

P6, paragraph 1, Line 11

This method differs from that of previous studies - which take 10 fields of view from the mid-cortex all around the cross-section. It is unclear if you are doing a transect from the inner to outer cortex and getting the average, or if you are taking inner versus outer fields of view randomly all over the cross-section - if the latter then this method is not being very consistent. You need to standardize this method more.

**REPLY**: We have given a detailed response above. In addition, we are doing an average cortical porosity from the inner to outer cortex rather than the mid cortex. As we know that cortical porosity is different from the inner to outer cortex, so it also has a problem to just choose mid-cortex, because cortical porosity is gradually changed from the inner to outer cortex. How do we define the mid cortex is a problem and objective.

P7, paragraph 2.

Comment: There is a new reference which will help you compare your results to the SA Lystrosaurus, you need to include this:

Botha, J. 2020. The paleobiology and paleoecology of South African *Lystrosaurus*. *PeerJ* 8:e10408. DOI: doi.org/10.7717/peerj.10408.

**REPLY**: Thank you for your information. We have added this reference and more comparison in the discussion part.

P7, table 2

Comment: separate into two separate columns

**REPLY**: modified as suggested.

P7, table 2

Comment: so would these values mark the highest being the inner cortex and the lowest value being the outer cortex?

**REPLY**: Sorry for the misunderstanding. Providing the variation of cortical thickness was suggested by Megan Whitney. It is the ratio of compact bone (one side) to the diameter of the whole cross-section. For example, see Fig S2E. The dorsal cortex is thin and the ventral cortex is relatively thick, so the cortical thickness ranges from 16-41%. Please see the method part for detailed information.

P8, table 2

extensive secondary reconstruction would imply dense Haversian bone, or that the reconstruction has reached the outer cortex - is this the case for your elements here? It doesn't look like it from the figures

**REPLY**: Thank you for pointing it out. We have modified this sentence as “a few secondary osteons”

P8, Juvenile, line 5.

Comment: where would you denote the edge of the medullary cavity here with such a gradual transition zone? You need to explain how you got these measurements and how you ensured that they were not subjective, but standardized

**REPLY**: We have added more explanation on how to identify compact bone and medullary cavity. The compact bone tissues are connected tightly without bone trabecula and large resorbed cavity, whereas the medullary cavity either empty (we call it free medullary cavity) or filled with trabecula, which is usually isolated or loosely connected. However, sometimes, the trabecula is abundant and show a similar connection as in compact bone, and could not be separated clearly.

P9 line 2. Comment: in circumferential rows in parts?

**REPLY**: Yes, added.

P9, line 11.

not shown in Fig 1B - label or take out in the text here

**REPLY**: The word “Fig. 1B” has been taken out here.

P9, figure 1. I know what you mean here by wide-open canals - but this isn't a very good term. Just label them large primary canals because that's what they are.

**REPLY**: Thank you for your suggestions. Modified.

P9, last line.

Comment: then how did you calculate cortical thickness?

P10 line 8. Comment: then how accurate is the cortical thickness measurement?

**REPLY**: We have provided more explanation on defining medullary cavity and compact bone in the method, See our explanation above.

P10, Fig 2 caption N.

Comment: I'm not convinced those are secondary osteons - I cannot see a cement line or How ship's lacunae - you need a better close up of this

**REPLY**: We have changed to a close up new picture to show secondary osteon here.

P11 paragraph 2 line 11.

Comment: according to your definition then - these growth marks cannot be seen around the whole circumference of the bone? So are they really indicative of a change in growth rate?

**REPLY**: These growth marks usually associated with parallel-fibered bone in the outermost region, so we consider it to indicate a slow growth rate.

P12 Subadult. Line 11.

Comment: This is not shown from Fig 3A and B - I would not call those channels "significantly reduced"

**REPLY**: we have removed the word “significantly”.

P12 Subadult. Line 12. can this LAG be followed around the whole circumference?

**REPLY**: This LAG is weak and not clear in some parts under normal light, and it is also not clear under polarized light, so we revised it to be a growth mark in the revised MS. Therefore, we remove this individual to be within a juvenile stage.

P13. Line 1. cannot see the LAG here

**REPLY**: We have removed the word “a LAG” here.

P13 paragraph 2 Line 13

It looks like a double LAG - don't say several otherwise it sounds like an EFS.

**REPLY**: We did not say several LAGs. There is only one prominent LAG and we wrote “a distinct LAG comprised of several growth marks”. It does show more than two growth marks (weak growth lines) but not LAGs. We remarked the arrow positions to show growth marks that more clear.

P13 Figure 4 caption. Line 3.

Comment: I'm not convinced - looks like two not three. And does the multiple number stay consistent around the whole cortex or does it become one LAG at some point?

**REPLY**: Again, we did not say it has three LAGs, instead, it is just one LAG. But we can see that it contains several growth marks. It becomes narrower but still a distinct LAG in other regions.

P13 Figure 4 caption. Line 5.

Comment: this looks like a single LAG here

**REPLY**: Yes. We agree. It is a single LAG containing several growth marks (or weak growth lines).

P14 line 1. Comment: looks like a double LAG here too

**REPLY**: Yes. We agree and have modified in the revised MS.

P14 line 1. this isn't a normal term - they are either resorption canals or canals that are just starting to form - look to see which one

**REPLY**: Yes. Here we only describe its shape that was suggested by another reviewer. They probably represent resorption canals.

P14 paragraph 2 line 6

Comment: so what are these? how far do they go around the cortex? do they run with the LAG or are they separate?

**REPLY**: Here we mean that a clear LAG was formed by several growth marks (weak growth lines that vary throughout the whole cortex), but we rethink parallel-fibered bone is more suitable. We have modified this sentence as “A clear LAG, as well as parallel-fibered bone, are present at the mid-region as in the fibula”.

P14 paragraph 2 line 7

Comment: cannot still be abundant but also strongly reduced - rephrase - I wouldn't call the canals strongly reduced. Perhaps there is a slight decrease in vascular canal size towards the periphery

**REPLY**: We have changed the word “strongly” to “gradually”.

P14 adult stage.

Comment: how do you define adult tissues?

**REPLY**: Adult stage was usually defined by the presence of EFS or lamellar/parallel-fibered bone tissue at the periphery. Here, we found parallel-fibered bone at the periphery. However, we agree that the layer of parallel fibered bone was still narrow. We have changed this stage to “late subadult” as suggested.

P15 Line 1

Comment: as with my comments above for every element - how then do you decide where the edge of the compact cortex is?

**REPLY**: cortical thickness and porosity have been removed in this specimen.

P15 paragraph 1 last line.

Comment: you've added in *Placerias* - but an EFS has been found in *Kannemeyeria* as well - see Botha-Brink and Angielczyk 2010 - those are the only two so you may as well name them seen as they are dicynodonts

**REPLY**: We have added this reference.

P15 Fig 5 caption.

Comment: I am not convinced this is an adult - at most, with such a small region of parallel-fibered bone at the periphery we are seeing a first transition to slower forming bone tissues - so at most this is a subadult. An adult would have a much larger region or parallel-fibered bone or lamellar bone or an EFS

**REPLY**: We have changed the stage “adult” to “late subadult”.

P15 Last line.

Comment: change this term

**REPLY**: Modified.

P16 Fig. 6. Comment: looks like a double LAG not triple

**REPLY**: This should be at least two LAGs rather than a double LAG. These two LAGs have more than three LAGs and a wide parallel fibered bone have been founded in the associated femur. But it has already been sampled by another research and will be published elsewhere.

P17. Line 5 I would say two

**REPLY**: Modified as suggested.

P17. Line 5.

Comment: no - not enough to indicate an EFS - looks like a double LAG. This is not an EFS - as you decide it's not one from your next comment - rather just take out the phrase "similar to an EFS"

**REPLY**: We have deleted this sentence.

P17 Discussion. Line 4.

**Comment**: There is not enough evidence to indicate this third stage - the rib cannot be used for this and the two femora look like subadults

**REPLY**: We have modified the “adult” to “late subadult” and modified the text to make it more clear.

P17 Discussion. Line 6.

no - you don't know that - no EFS - a double LAG is not enough to indicate general slow growth - definite subadult, but I wouldn't call these femora and rib adults

**REPLY**: We have changed the “adult” to “late subadult” stage.

P17 Discussion. Line 6.

Comment: what about the humerus?

**REPLY**: We have added the word“the humerus”.

P18 Paragraph 3 line 4.

**Comment**: only three out of seven have more than 1 LAG - and the rib looks like a double LAG - i.e. indicating one season, the rib is not a good one to include in stating that more than 1 or 2 LAGs are present. That just leaves the two femora that have 3 or 4 LAGs - this is not a high sample size

**REPLY**: As we mentioned above, the rib in Figure 6 has at least two LAGs, and this individual also sampled the femur which has more than 2 LAGs and wide parallel-fibered bone tissue at the periphery. But it has been sampled by another researcher and will be published elsewhere.

P18 Paragraph 3 line 8

these are not the largest Triassic Lystrosaurus - compare your elements with Botha 2020

P18 Paragraph 3 line 9

confirm this by comparing with the largest Triassic humeri in Botha 2020

**REPLY:** We have added a new comparison and S4 Figure according to this new reference, and we found that there is no significant size difference between Chinese and South Africa *Lystrosaurus*, but Chinese *Lystrosaurus* tends to have more LAGs, which may be affected by different species or environment. This did not affect our conclusion that Chinese *Lystrosaurus* have reached at least the subadult stage based on the presence of LAGs and relatively large body size (see revised MS for detailed information).

P19. Line 3.

Comment: you don't show this in the table - in the table it's just the two femora that have more than two LAGs. There is only one juvenile humerus in your tables

**REPLY**: We have modified this sentence as “All the other individuals with humeral length larger than 12 cm (including estimated value) have more than two LAGs to be among late subadult stage (Table 1)”. The two femora IVPP V26546 and IVPP V26547 have more than two LAGs. IVPP V26546 also preserves a humerus (12 cm), although we did not sample it. IVPP V26547 and IVPP V26548 do not preserve the humeri, but the estimated humeral length is larger than 12 cm. We have changed the “Elements” to “Sampled elements” to make it more clear.

**P20 Line 1**

**Comment**: yes but this has been shown to be false in Botha 2020 - the size reduction is just because they didn't live long enough to attain large sizes, not because of miniaturization.

**REPLY**: We have added this new reference.

P20 paragraph 2 line 7

Comment: cannot start a sentence with and

**REPLY**: Modified.

P20 paragraph 2, line 8

Comment: not necessarily - it depends on where you sampled your rib. Cortical thickness in ribs differ along the length of the shaft and you haven't said where your sample was taken from - so it's very difficult to compare ribs unless you know for sure they were sampled at the same position along the shaft

**REPLY**: We have deleted this sentence.

Sincerely,

Fenglu Han
