# Supplementary material for: Preliminary bone histological analysis of Lystrosaurus (Therapsida: Dicynodontia) from the Lower Triassic of North China, and its implication for lifestyle and environments after the end-Permian extinction
Source: PLoS One. 2021 Mar 18;16(3):e0248681. doi: 10.1371/journal.pone.0248681 (PMC7971864; doi:10.1371/journal.pone.0248681)
Supplement: Attachment — Submitted filename: Response to reviewers R3.docx [file pone.0248681.s013.docx]

**Dear Editor and Reviewers:**

Thank you for your letter and for the reviewers’ comments concerning our manuscript entitled “Bone histology of *Lystrosaurus* (Therapsida: Dicynodontia) from the Lower Triassic of North China, and its implication for lifestyle and environments after the end-Permian extinction” (ID: PONE-D-20-17684). Those comments are all valuable and very helpful for revising and improving our paper, as well as important for highlighting the significance of our work. We have studied the comments carefully and made a number of corrections to address them, which we hope to meet with approval. The revised portions of the MS were marked by red colors. The main corrections to the paper and the responses to the editor and reviewer’s comments are as following:

**Editor’s comment:** I further strongly recommend that you indicate the preliminary nature of this study in the title of the manuscript by changing the title to something like: "Preliminary bone histological analysis of Lystrosaurus (Therapsida: Dicynodontia) from the Lower Triassic of North China, and its implication for lifestyle and environments after the end-Permian extinction".

REPLY: We have modified the title as suggested.

**Reviewer #5**: I have reviewed a revision of this manuscript and it is greatly improved. The authors have clarified or corrected most issues, but I still have a few queries that perhaps need further clarification.

1. The main issue I have pertains to how the authors define growth marks. Growth marks, growth lines and growth rings all mean the same thing, and include both LAGs and annuli. A LAG is a cement line, and there can be more than one closely spaced LAG, 2 LAGs is referred to as a double LAG, 3 as triple etc. When the LAGs are so closely spaced that there are no vascular canals between them, osteohistologists refer to these growth marks as double or triple LAGs etc – they pertain to one season (or one year), and so are not counted separately (as in a triple LAG would mean a one year old not a three year old). The authors sometimes refer to a LAG containing several growth marks. This is confusing because a LAG is a growth mark and a double or triple LAG is referred to as such, not a single LAG consisting of several growth marks. This needs a bit of clarification in the text (which I note in my comments on the pdf). (e.g. early subadult 26545 fibula is described as have a LAG of several growth marks),

REPLY: Thank you for clarifying the meaning of these words. In our paper, LAGs, growth lines (or rings) are the same things that indicate seasonal change, but growth marks are a little different. We follow Horner etal (1999) and Green etal (2010) in only identifying growth marks as LAGs and annuli if they are traced around the full circumference of the bone. Therefore, one growth mark may not represent one year, unlike one LAG. I agree entirely with your definition of double LAG and triple LAGs. In the new revised version, we have added the definition of growth marks in the method. Moreover, we have removed the statement of “ a LAG containing several growth marks”.

2. The LAG number in juvenile humerus IVPP V26544 is unclear. In the table the authors say 0 growth rings, but in the text they note a LAG. I don’t know if this is because of issue 1 where there needs to be clarification about the definition, or whether the table needs to be changed to show that there is a growth mark in this bone.

REPLY: As we have mentioned above, the growth mark in the text is not a true LAG, for it is unstable and does not go across the whole cross-section of the bone. Here we assume that a growth mark does not represent one year.

3. In the discussion the authors mention the larger body size of the Chinese *Lystrosaurus* compared to the South African species but the graph in S4 shows the Chinese *Lystrosaurus* to be equal to the Triassic species of South Africa, I don’t see how the Chinese *Lystrosaurus* are larger.

REPLY: Sorry for the confusion. In S4, the graph only compares *Lystrosaurus* with LAGs. But most of South Africa *Lystrosaurus* do not have LAGs and have small sizes (this can be checked from the measurement of Lystrosaurus in Botha et al. 2020). Therefore, generally, the Chinese *Lystrosaurus* looks larger because most of them reached subadult here. As this is a little confusing, we have deleted “large body size” in the revised version.

4. The authors suggest that there were better conditions in North China compared to South Africa, but the Chinese specimens have more LAGs and they don’t seem to be any bigger than the SA specimens. If the Chinese specimens have more LAGs wouldn’t this rather suggest that the China environment was more harsh (causing them to react to a more seasonal environment and deposit LAGs), and/or that the Chinese specimens were stunted if they have more LAGs (and thus older) but are not physically larger?

REPLY: This is a very good question. Our conclusion that North China may have a better environmental condition than South Africa is mainly based on most Chinese *Lystrosaurus* has reached subadult stage when they died, whereas most South Africa *Lystrosaurus* are still young when they died. The “high juvenile excess mortality” (Botha et al. 2020) should be caused by an unusual environmental condition, although we still do not know the accurate condition. The presence of LAGs with large size suggests that the Chinese *Lystrosaurus* is at least reach a subadult stage. They have a relatively better condition than South Africa, but it does not mean that they have a good environmental condition. The environment may be harsh, but they could survive to a late stage may be through seasonal reduction in metabolic activity (torpor), like *Lystrosaurus* from Antarctica (Whitney and Sidor 2020). We have added this new statement.

Whether the Chinese *Lystrosaurus* have more LAGs than those from South Africa **with similar sizes** still needs more data to prove. In addition, the presence of LAGs was also affected by different species. For example, I notice that *L. declivis* with one LAG is large than *L. murrayi* (figure S4). But this needs more data to support.

Comments in the PDF

Page 3, line 9. you should add in here, but see Botha 2020

REPLY: Added as suggested.

Page 9. Table 1. Comment: take this out - I don't see an e anywhere

REPLY: Deleted.

Page 11 line 6. but in the table you say 0 growth rings

Page 12 line 1. Comment: as growth mark number is important in this study you need to say how many growth marks are present. You only show one in 2G and H, but you mention growth marks plural - and in the table you say 0 growth rings, I think there is an issue here on your definition of growth marks

REPLY: We have modified “growth marks” to “one growth mark”. As we mentioned above, the growth mark could not trace around the whole cross-section of bone and may not indicate a year (unlike LAG or growth ring), so we wrote 0 growth ring in the table.

Page 13. Fig. 3 line 3. I would call this a LAG

REPLY: Here the picture shows the most prominent line, but it is only clear in this region and not shown in other parts of the bone, so we just say it is a growth mark, which has already been defined in the method part.

Page 14. Line 2. This is where the definition of growth marks is an issue - you show 3 arrows very close together, with no vascular canals between them - each growth mark here is a LAG - so this forms a triple LAG - which is interesting because triple LAGs have not been found in the SA Triassic species - but it needs to be remembered that this still represents only one season

REPLY: Here we prefer not to use the word “a triple LAG” because only one prominent LAG go across the whole cross-section, other lines disappeared soon. In addition, the third rest line is too weak and we have modified as “the presence of two rest lines” here. Therefore, the revised sentence is “A distinct LAG is present near the mid-region of the cortex (Fig 4A), and it separates into two closely spaced rest lines in some regions (Fig 4B).”

Page 14. Fig. 4. Line 3. you need to rephrase - this is a fibula showing a triple LAG

REPLY: We have modified this sentence as “partial outer cortex of the fibula showing two closely spaced rest lines (arrows).”

Page 14. Fig. 4. Line 7. Rephrase to a double LAG

REPLY: Modified as suggested. This is a double LAG as they are very stable and go across the whole cross-section.

Page 16 paragraph 2, line 7.

Comment: if it's "a" avascular layer - then the LAG is accompanied by an annulus - not annuli

REPLY: We have modified this sentence as “A LAG with a narrow avascular layer of an annulus is present in the mid-cortex.

Page 16 paragraph 2, line 9.

this like C shows one annulus - not annuli

REPLY: Corrected.

Page 19, paragraph 2, line 1.

Comment: but your graph shows that the Chinese *Lystrosaurus* is similar in size to the of the Triassic South African sample

REPLY: Right. But the graph only compares *Lystrosaurus* with LAGs. But most of South Africa *Lystrosaurus* do not have LAGs and have small sizes. Therefore, generally, the Chinese *Lystrosaurus* is larger. Here we have deleted “large body size” for this confusion.

All other grammar mistakes that mentioned have been corrected.

Thank you and best regards,

Fenglu Han
